# Supplementary material for: Volunteering and political participation are differentially associated with eudaimonic and social well-being across age groups and European countries
Source: PLoS One. 2023 Feb 3;18(2):e0281354. doi: 10.1371/journal.pone.0281354 (PMC9897590; doi:10.1371/journal.pone.0281354)
Supplement: S1 Table — (PDF) [file pone.0281354.s001.pdf]

S1 Table

Country-Level Variance Estimates for the Main Effects of Voluntary Participation on Eudaimonic and Social Well-Being and for the Interactions With Age

Random effects (σ^2)	Flow experiences	Sense of direction	Sense of competence	Learning new things	Generalized trust	Perceived social support	Loneliness
$S_{nonpolitical\ volunteering}$	0.007** (0.004)	0.025** (0.015)	0.017** (0.009)	0.013** (0.007)	0.006** (0.004)	0.005** (0.003)	0.001* (0.001)
$S_{nonpolitical\ volunteering*15-30}$	0.005** (0.003)	0.030** (0.019)	0.009** (0.006)	0.009** (0.006)	0.005** (0.004)	0.006** (0.005)	0.002** (0.002)
$S_{nonpolitical\ volunteering*31-60}$	0.003** (0.002)	0.012** (0.008)	0.005** (0.003)	0.008** (0.005)	0.003** (0.003)	0.006** (0.004)	0.002* (0.001)
$S_{political\ participation}$	0.420** (0.241)	1.338** (0.766)	0.669** (0.373)	1.117** (0.603)	0.300** (0.186)	0.270** (0.201)	0.121** (0.078)
$S_{political\ participation*15-30}$	0.512** (0.318)	0.967** (0.683)	0.768** (0.475)	1.111** (0.651)	0.382** (0.299)	0.602** (0.407)	0.165** (0.116)
$S_{political\ participation*31-60}$	0.337** (0.212)	0.870** (0.568)	0.505** (0.300)	0.588** (0.368)	0.146** (0.125)	0.336** (0.240)	0.198** (0.124)

Note. N = 54,673. Variance estimates for regression slopes (S) with standard errors in parentheses, which stem from the models with all individual-level control variables, are shown. Reference group for age: 61+. The main effects of age were also random (i.e., allowed to vary across countries; estimates not shown). Significance levels are based on asymmetric Bayesian credibility intervals.

* $p < .05$. ** $p < .01$.
