# Supplementary material for: Volunteering and political participation are differentially associated with eudaimonic and social well-being across age groups and European countries
Source: PLoS One. 2023 Feb 3;18(2):e0281354. doi: 10.1371/journal.pone.0281354 (PMC9897590; doi:10.1371/journal.pone.0281354)
Supplement: S2 Table — (PDF) [file pone.0281354.s002.pdf]

S2 Table

Cross-Level Three-Way Interactions Between Contextual Variables, Voluntary Participation, and Age for Eudaimonic Well-Being

Country-level variables	Outcome	$S_{nonpolitical\ volunteering}$	$S_{political\ participation}$	$S_{15\text{-}30}$[a]	$S_{31\text{-}60}$[a]	$S_{nonpolitical\ volunteering*15\text{-}30}$	$S_{nonpolitical\ volunteering*31\text{-}60}$	$S_{political\ participation*15\text{-}30}$	$S_{political\ participation*31\text{-}60}$
Flow experiences									
Intercept	0.00[b]	0.07**		-0.14*	-0.11*	-0.03	-0.03		
		(0.02)		(0.05)	(0.05)	(0.02)	(0.01)		
Life expectancy at	0.10*	-0.02*		-0.08*	-0.05	0.00	0.01		
age 65	(0.04)	(0.01)		(0.03)	(0.03)	(0.01)	(0.01)		
Intercept	0.00[b]		0.23	-0.14*	-0.11*			-0.23	-0.15
			(0.15)	(0.05)	(0.05)			(0.17)	(0.15)
Life expectancy at	0.10*		-0.10	-0.08*	-0.05			0.13	0.08
age 65	(0.04)		(0.09)	(0.03)	(0.03)			(0.10)	(0.09)
Intercept	0.00[b]	0.07**		-0.14*	-0.10*	-0.04*	-0.03*		
		(0.02)		(0.06)	(0.05)	(0.02)	(0.02)		
Youth	0.00	0.00		0.01	0.01	0.00	0.00		
unemployment rate	(0.01)	(0.00)		(0.01)	(0.01)	(0.00)	(0.00)		
Intercept	0.00[b]		0.20	-0.14*	-0.10*			-0.23	-0.13
			(0.15)	(0.06)	(0.05)			(0.17)	(0.15)
Youth	0.00		0.01	0.01	0.01			-0.01	0.00
unemployment rate	(0.01)		(0.02)	(0.01)	(0.01)			(0.02)	(0.02)
Sense of direction									
Intercept	7.15**	0.07		-0.35*	-0.31*	-0.01	-0.02		
	(0.15)	(0.03)		(0.16)	(0.12)	(0.04)	(0.03)		

Life expectancy at	0.06	-0.03		0.00	0.00	0.00	0.02		
age 65	(0.09)	(0.02)		(0.09)	(0.07)	(0.02)	(0.02)		
Intercept	7.14**		0.18	-0.34*	-0.30*			-0.39	-0.05
	(0.15)		(0.28)	(0.15)	(0.11)			(0.28)	(0.25)
Life expectancy at	0.06		-0.01	0.00	0.00			0.07	-0.02
age 65	(0.09)		(0.16)	(0.09)	(0.07)			(0.17)	(0.15)
Intercept	7.15**	0.07		-0.33*	-0.31**	-0.01	-0.01		
	(0.14)	(0.03)		(0.15)	(0.11)	(0.04)	(0.03)		
Youth	0.00	0.00		-0.01	-0.01	0.00	0.00		
unemployment rate	(0.02)	(0.00)		(0.02)	(0.01)	(0.01)	(0.00)		
Intercept	7.14**		0.17	-0.32*	-0.30**			-0.40	-0.03
	(0.14)		(0.26)	(0.15)	(0.11)			(0.27)	(0.23)
Youth	0.00		-0.01	-0.01	-0.01			-0.01	0.02
unemployment rate	(0.02)		(0.03)	(0.02)	(0.01)			(0.03)	(0.03)
					Sense of competence				
Intercept	0.00[b]	0.09**		-0.29**	-0.20**	-0.04	-0.03		
		(0.03)		(0.07)	(0.06)	(0.02)	(0.02)		
Effective	0.03	-0.01		0.03	0.03	0.01	0.00		
retirement age	(0.05)	(0.01)		(0.03)	(0.03)	(0.01)	(0.01)		
Intercept	0.00[b]		0.22	-0.29**	-0.20**			-0.20	-0.12
			(0.19)	(0.07)	(0.06)			(0.22)	(0.18)
Effective	0.03		-0.05	0.03	0.03			0.03	0.03
retirement age	(0.05)		(0.08)	(0.03)	(0.03)			(0.10)	(0.08)
Intercept	0.00[b]		0.24	-0.27**	-0.19**			-0.21	-0.12
			(0.17)	(0.07)	(0.06)			(0.20)	(0.17)

Mean age	0.08		-0.17	-0.05	-0.04			0.19	0.08
MPs/cabinet	(0.06)		(0.10)	(0.04)	(0.04)			(0.12)	(0.10)
Intercept	0.00[b]	0.09**		-0.27**	-0.19**	-0.03	-0.03*		
		(0.03)		(0.07)	(0.06)	(0.02)	(0.02)		
Youth	-0.01	0.00		0.01	0.00	0.00	0.00		
unemployment rate	(0.01)	(0.00)		(0.01)	(0.01)	(0.00)	(0.00)		
Intercept	0.00[b]		0.24	-0.27**	-0.19**			-0.21	-0.12
			(0.19)	(0.07)	(0.06)			(0.22)	(0.17)
Youth	-0.01		0.01	0.01	0.00			-0.01	-0.01
unemployment rate	(0.01)		(0.02)	(0.01)	(0.01)			(0.02)	(0.02)
					Learning new things				
Intercept	3.87**	0.10**		0.42**	0.22**	-0.05*	-0.05*		
	(0.16)	(0.03)		(0.13)	(0.08)	(0.03)	(0.02)		
Effective	0.05	-0.01		-0.02	0.01	0.01	0.00		
retirement age	(0.07)	(0.01)		(0.06)	(0.04)	(0.01)	(0.01)		
Intercept	3.86**		0.69**	0.43**	0.22**			-0.68*	-0.41*
	(0.16)		(0.24)	(0.13)	(0.08)			(0.26)	(0.20)
Effective	0.05		-0.01	-0.01	0.01			-0.01	-0.04
retirement age	(0.07)		(0.10)	(0.05)	(0.04)			(0.11)	(0.08)
Intercept	3.86**		0.66**	0.44**	0.23**			-0.64**	-0.40*
	(0.12)		(0.22)	(0.10)	(0.07)			(0.23)	(0.18)
Mean age	0.20*		-0.16	-0.15*	-0.09*			0.21	0.10
MPs/cabinet	(0.08)		(0.13)	(0.06)	(0.04)			(0.14)	(0.11)
Intercept	3.86**	0.10**		0.44**	0.23**	-0.06*	-0.05*		
	(0.15)	(0.02)		(0.12)	(0.08)	(0.02)	(0.02)		

Youth	0.00	0.00		0.00	0.00	0.00	0.00		
unemployment rate	(0.02)	(0.00)		(0.01)	(0.01)	(0.00)	(0.00)		
Intercept	3.86**		0.66**	0.44**	0.23**			-0.64**	-0.40*
	(0.15)		(0.22)	(0.12)	(0.08)			(0.24)	(0.19)
Youth	-0.01		-0.02	0.00	0.00			0.01	0.00
unemployment rate	(0.02)		(0.03)	(0.01)	(0.01)			(0.03)	(0.02)

Note. N = 53,378–54,673. Reduced sample size in some models was due to the exclusion of countries with missing data on specific country-level indicators. Each pair of rows (intercept + predictor) represents a separate regression model with one cross-level three-way interaction tested. Cells show unstandardized linear regression coefficients with standard errors in parentheses. Effects were adjusted for the full set of control variables at the individual level. Flow experiences and sense of competence were modeled as latent variables. S = random slope.

[a] Reference category: age 61+.

[b] The intercept of a latent variable is fixed at zero.

* $p < .05$. ** $p < .01$.
