# Supplementary material for: Volunteering and political participation are differentially associated with eudaimonic and social well-being across age groups and European countries
Source: PLoS One. 2023 Feb 3;18(2):e0281354. doi: 10.1371/journal.pone.0281354 (PMC9897590; doi:10.1371/journal.pone.0281354)
Supplement: S3 Table — (PDF) [file pone.0281354.s003.pdf]

S3 Table

Cross-Level Three-Way Interactions Between Contextual Variables, Voluntary Participation, and Age for Social Well-Being

Country-level variables	Outcome	$S_{\text{nonpolitical volunteering}}$	$S_{\text{political participation}}$	$S_{15\text{-}30}$[a]	$S_{31\text{-}60}$[a]	$S_{\text{nonpolitical volunteering*15-30}}$	$S_{\text{nonpolitical volunteering*31-60}}$	$S_{\text{political participation*15-30}}$	$S_{\text{political participation*31-60}}$
				Generalized trust					
Intercept	0.00[b]		0.01	-0.23**	-0.19**			-0.16	-0.03
			(0.13)	(0.06)	(0.04)			(0.17)	(0.11)
Mean age	0.07		0.04	-0.01	0.00			-0.01	0.02
MPs/cabinet	(0.11)		(0.08)	(0.04)	(0.02)			(0.10)	(0.07)
Intercept	0.00[b]	0.00		-0.24**	-0.19**	0.03	0.03		
		(0.02)		(0.06)	(0.04)	(0.02)	(0.02)		
Youth	-0.03*	0.00		0.01	0.00	0.00	0.00		
unemployment rate	(0.02)	(0.00)		(0.01)	(0.00)	(0.00)	(0.00)		
Intercept	0.00[b]		0.01	-0.23**	-0.19**			-0.16	-0.02
			(0.13)	(0.06)	(0.04)			(0.17)	(0.11)
Youth	-0.03*		0.00	0.01	0.00			0.01	0.00
unemployment rate	(0.02)		(0.01)	(0.01)	(0.00)			(0.02)	(0.01)
				Perceived social support					
Intercept	0.00[b]	-0.01		-0.25**	-0.30**	0.04	0.02		
		(0.02)		(0.06)	(0.05)	(0.02)	(0.02)		
Life expectancy at	0.13*	-0.01		-0.03	-0.02	0.00	0.00		
age 65	(0.05)	(0.01)		(0.03)	(0.03)	(0.01)	(0.01)		
Intercept	0.00[b]		-0.33*	-0.25**	-0.30**			0.15	0.24
			(0.14)	(0.06)	(0.04)			(0.20)	(0.15)

Life expectancy at	0.13*		-0.01	-0.03	-0.03			0.13	0.05
age 65	(0.05)		(0.08)	(0.03)	(0.02)			(0.12)	(0.09)
Intercept	0.00[b]		-0.33*	-0.25**	-0.31**			0.14	0.27*
			(0.13)	(0.05)	(0.04)			(0.18)	(0.14)
Mean age	0.02		-0.07	-0.03	-0.02			0.15	0.09
MPs/cabinet	(0.06)		(0.08)	(0.03)	(0.02)			(0.11)	(0.08)
Intercept	0.00[b]	0.00		-0.25**	-0.31**	0.04	0.01		
		(0.02)		(0.06)	(0.04)	(0.02)	(0.02)		
Youth	-0.01	0.00		0.00	0.00	0.00	0.00		
unemployment rate	(0.01)	(0.00)		(0.01)	(0.00)	(0.00)	(0.00)		
Intercept	0.00[b]		-0.35**	-0.24**	-0.30**			0.16	0.30*
			(0.12)	(0.05)	(0.04)			(0.18)	(0.14)
Youth	-0.01		-0.02	0.00	0.00			0.01	0.02
unemployment rate	(0.01)		(0.01)	(0.01)	(0.00)			(0.02)	(0.02)
					Loneliness				
Intercept	1.66**	-0.01		-0.01	0.06**	0.01	0.01		
	(0.04)	(0.01)		(0.03)	(0.02)	(0.01)	(0.01)		
Life expectancy at	-0.09**	0.01		0.03*	0.03*	-0.01	-0.01		
age 65	(0.02)	(0.01)		(0.02)	(0.01)	(0.01)	(0.01)		
Intercept	1.66**		0.01	-0.01	0.06**			0.11	0.10
	(0.04)		(0.08)	(0.03)	(0.02)			(0.11)	(0.10)
Life expectancy at	-0.09**		-0.02	0.03*	0.03*			0.03	-0.02
age 65	(0.02)		(0.05)	(0.02)	(0.01)			(0.06)	(0.06)
Intercept	1.67**		0.01	-0.01	0.06**			0.11	0.09
	(0.06)		(0.08)	(0.03)	(0.02)			(0.10)	(0.10)

Mean age	-0.03		0.02	0.02	0.01			-0.04	-0.04
MPs/cabinet	(0.03)		(0.05)	(0.02)	(0.01)			(0.06)	(0.06)
Intercept	1.67**	-0.01		-0.01	0.06**	0.02	0.01		
	(0.05)	(0.01)		(0.03)	(0.02)	(0.01)	(0.01)		
Youth	0.01	0.00		-0.01	0.00	0.00	0.00		
unemployment rate	(0.01)	(0.00)		(0.00)	(0.00)	(0.00)	(0.00)		
Intercept	1.67**		0.02	-0.01	0.06**			0.11	0.09
	(0.05)		(0.08)	(0.03)	(0.02)			(0.10)	(0.10)
Youth	0.01		0.00	0.00	0.00			0.00	0.00
unemployment rate	(0.01)		(0.01)	(0.00)	(0.00)			(0.01)	(0.01)

Note. N = 53,378–54,673. Reduced sample size in some models was due to the exclusion of countries with missing data on specific country-level indicators. Each pair of rows (intercept + predictor) represents a separate regression model with one three-way interaction tested. Cells show unstandardized linear regression coefficients with standard errors in parentheses. Effects were adjusted for the full set of control variables at the individual level. Generalized trust and perceived social support were modeled as latent variables. S = random slope.

[a] Reference category: age 61+.

[b] The intercept of a latent variable is fixed at zero.

* $p < .05$. ** $p < .01$.
