# Supplementary figures and images for: Volunteering and political participation are differentially associated with eudaimonic and social well-being across age groups and European countries
Source: PLoS One. 2023 Feb 3;18(2):e0281354. doi: 10.1371/journal.pone.0281354 (PMC9897590; doi:10.1371/journal.pone.0281354)

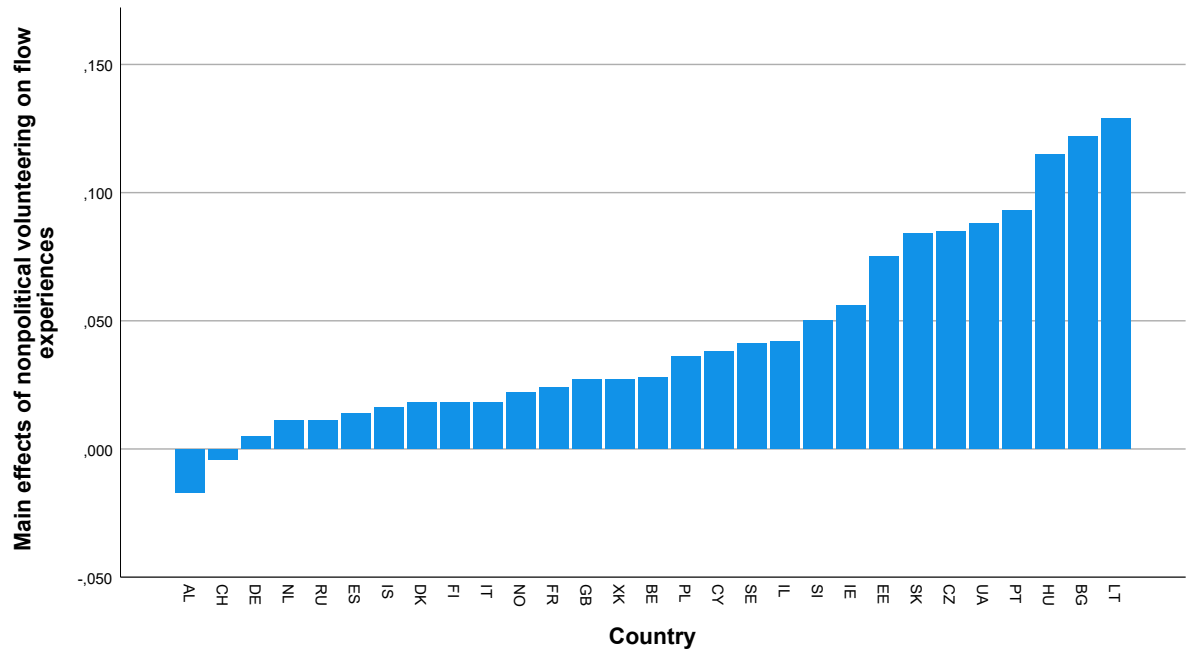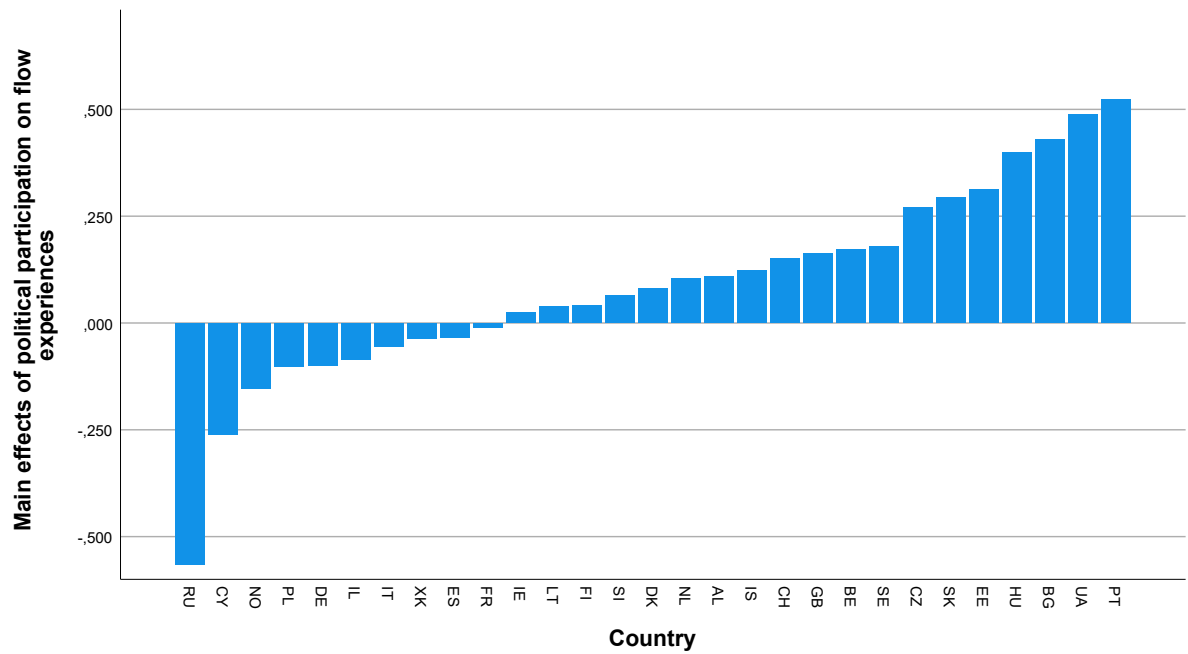

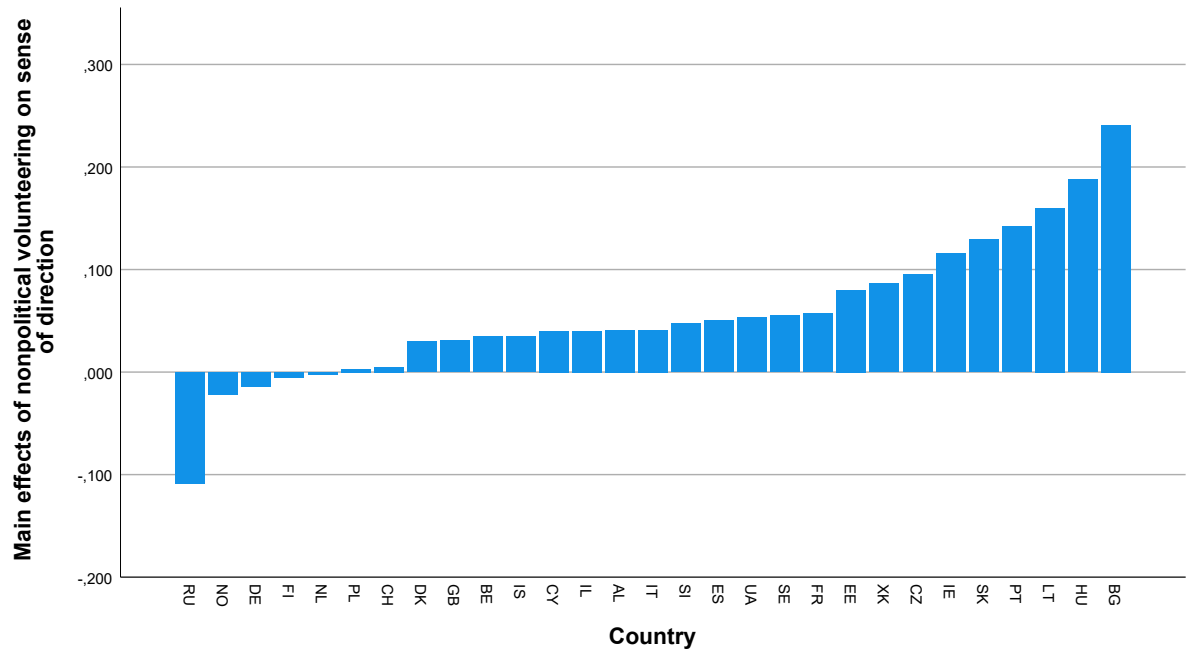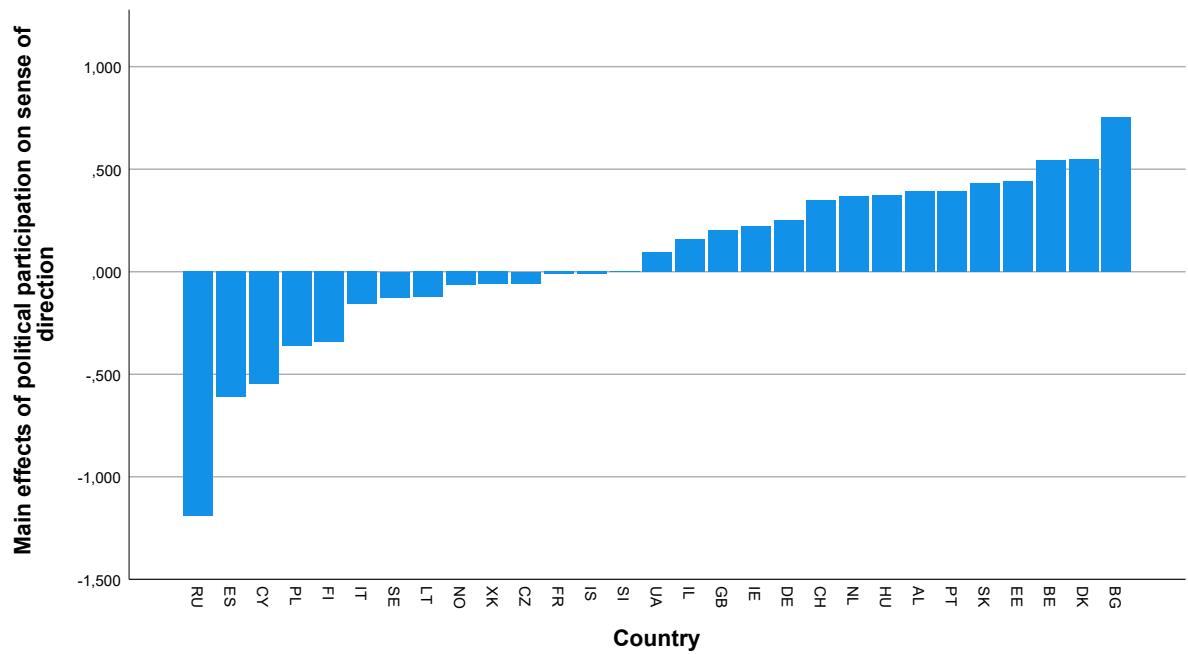

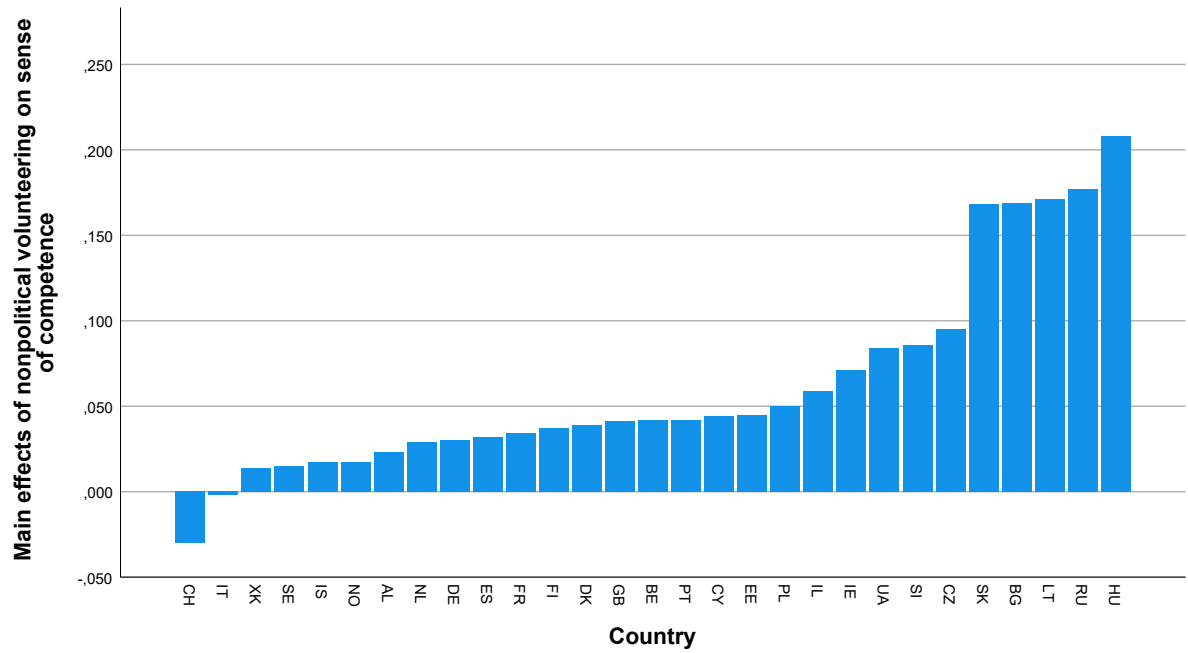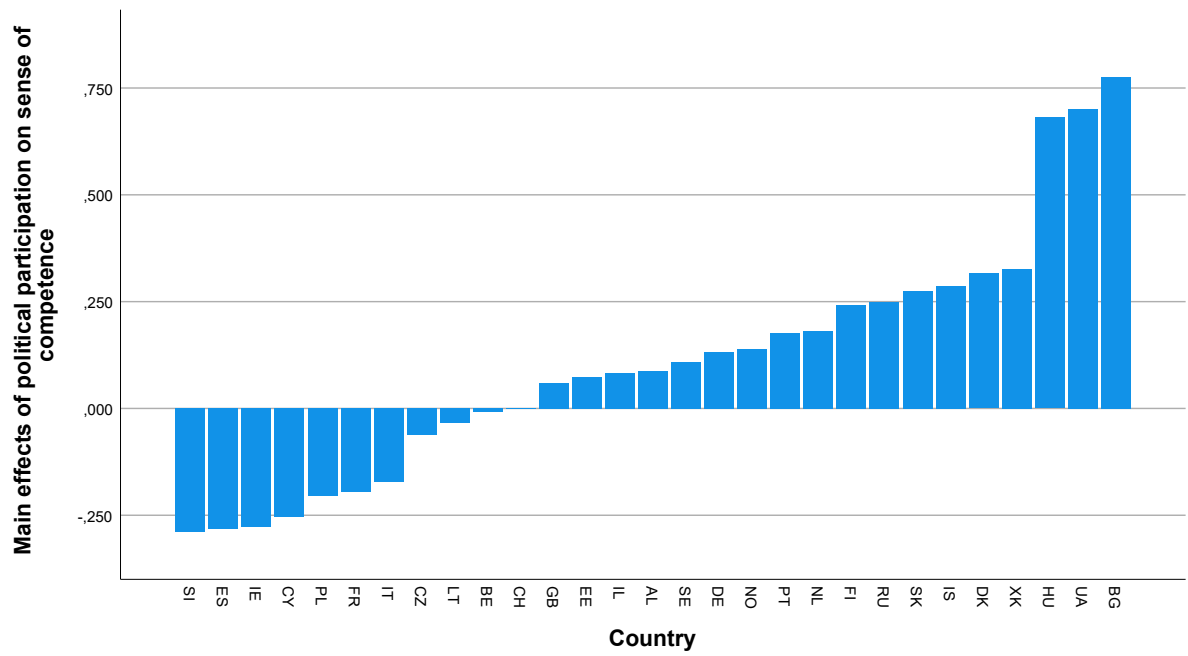

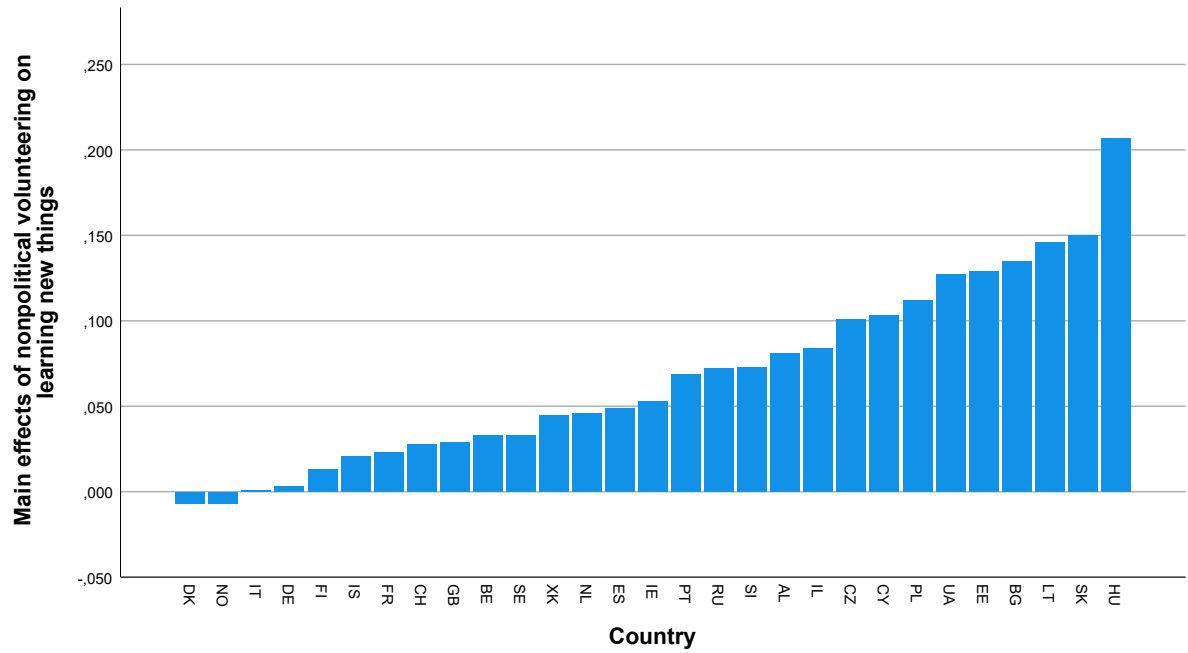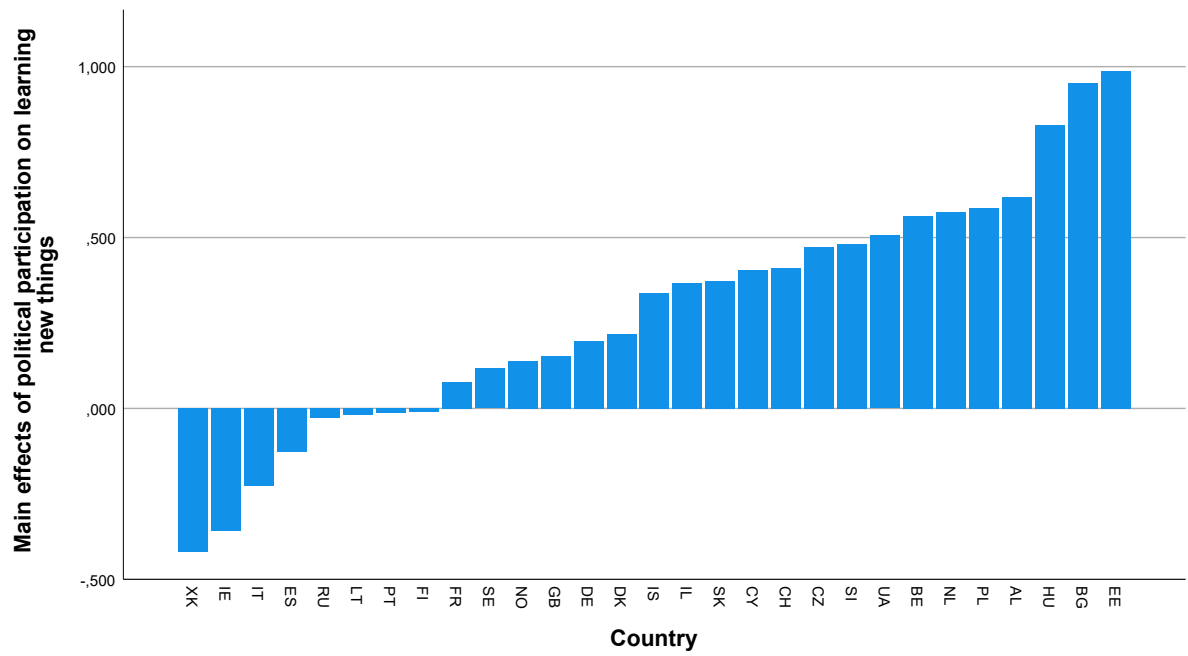

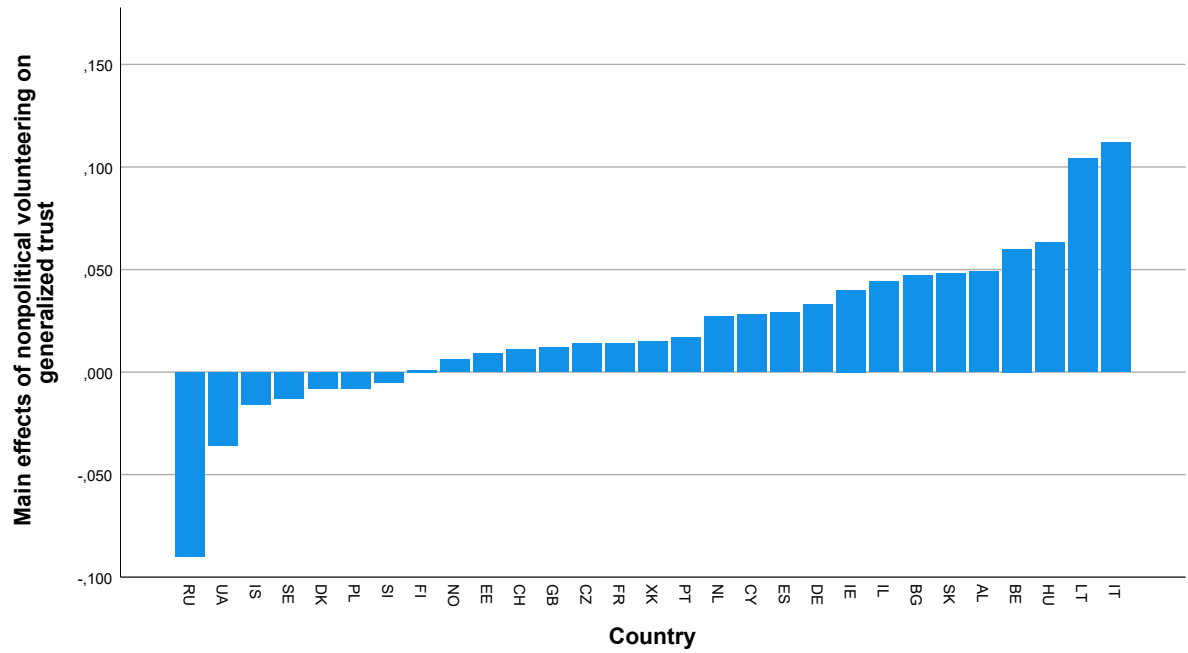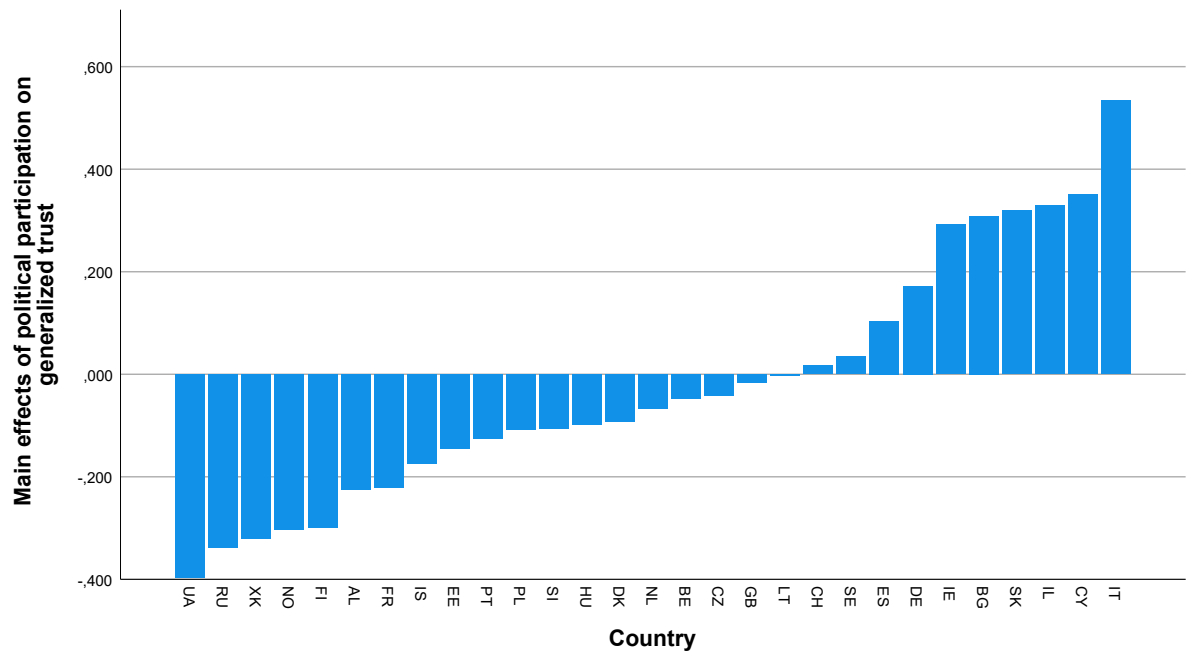

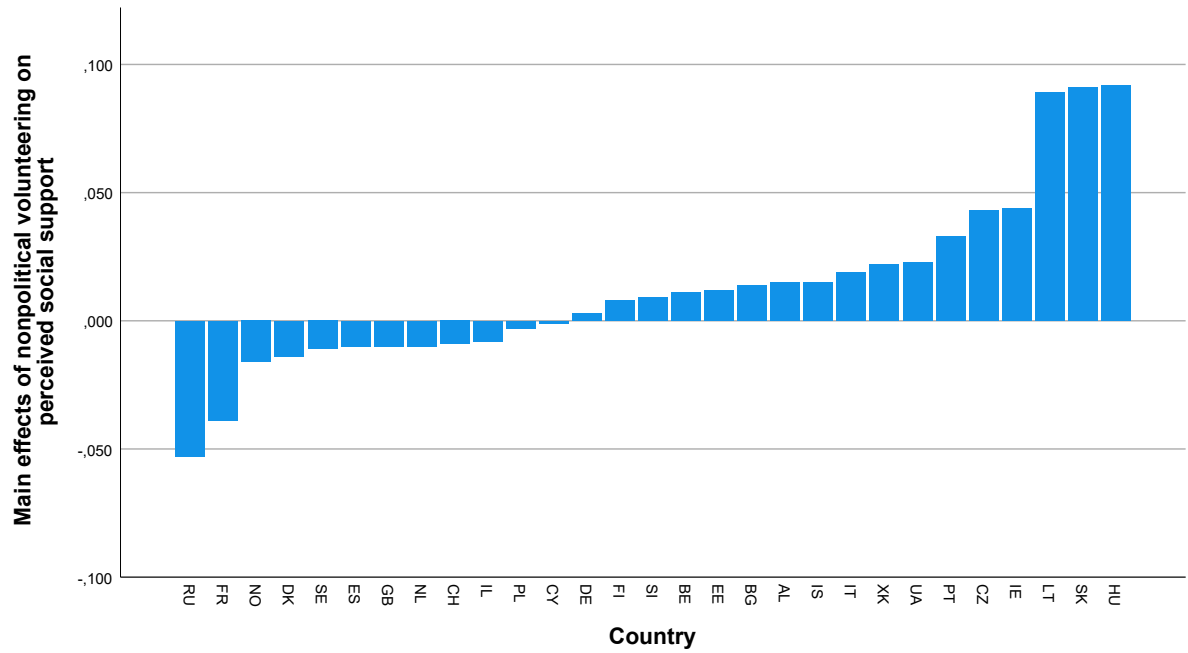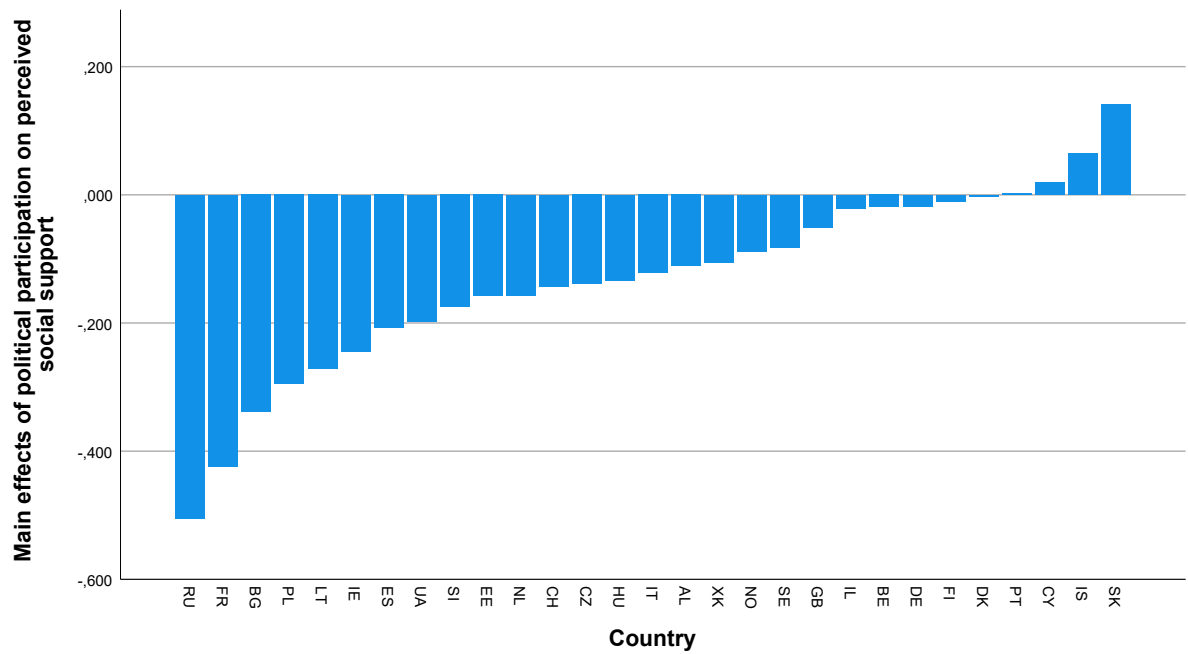

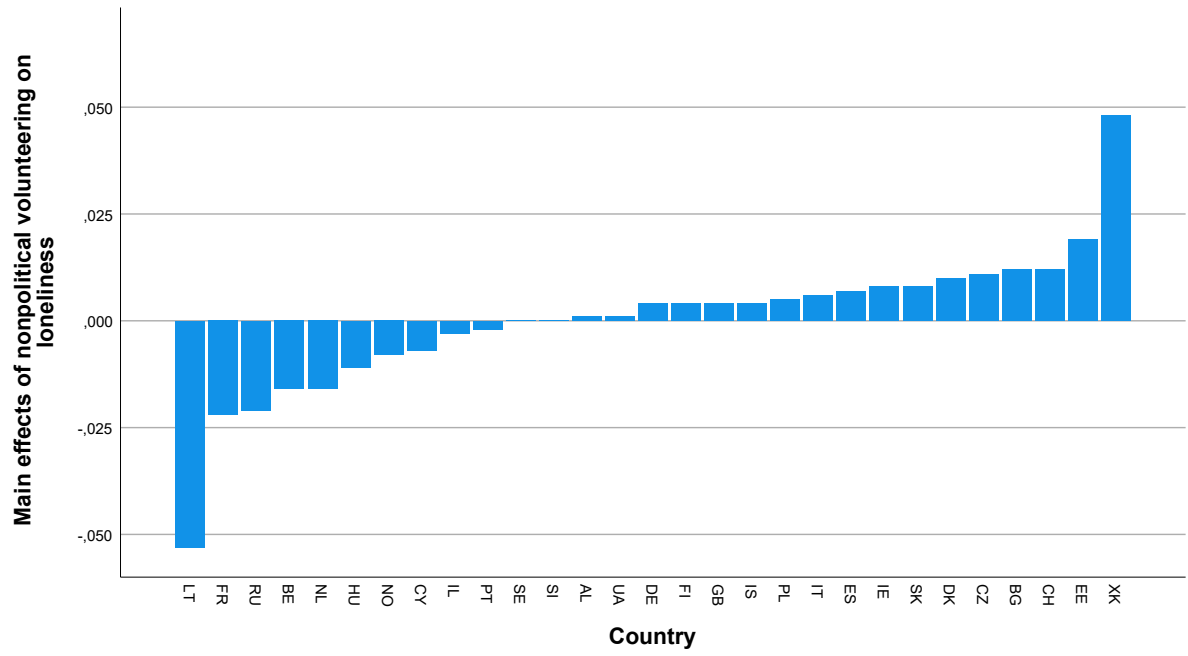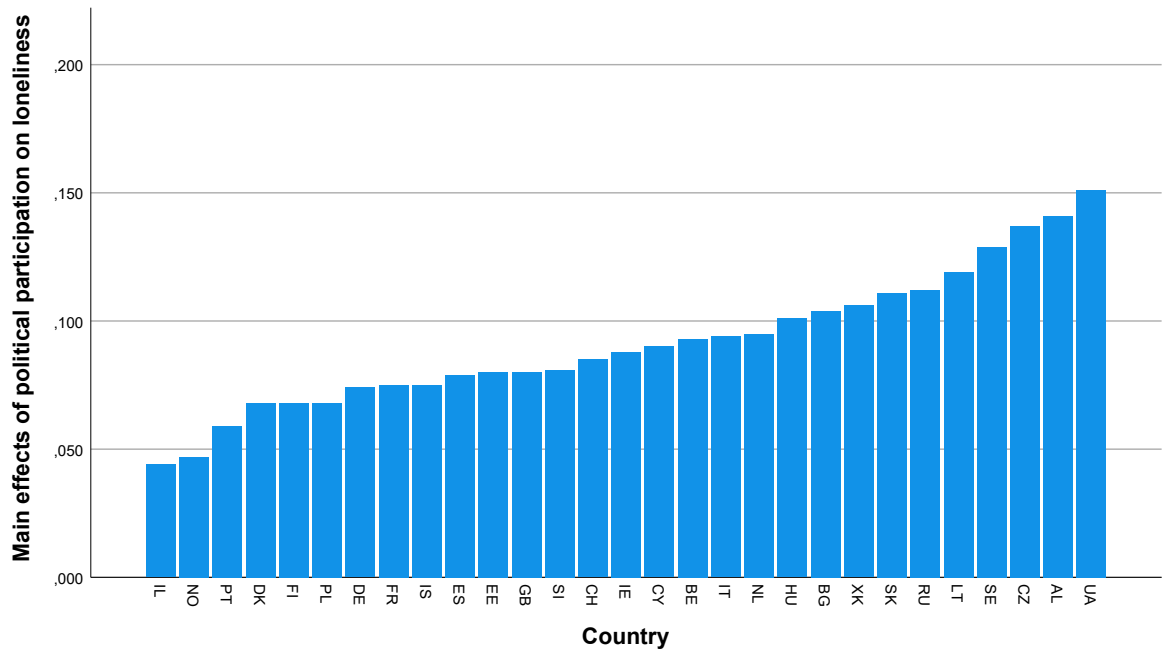

Supplement: S1 Fig — (PDF) [file pone.0281354.s004.pdf]

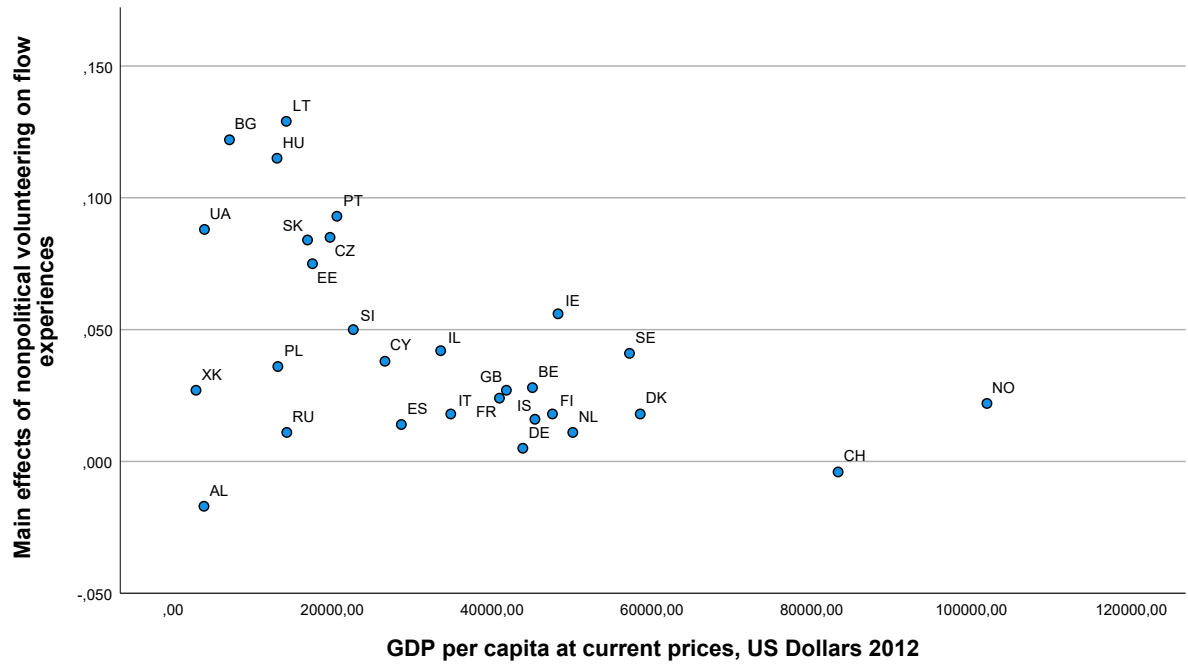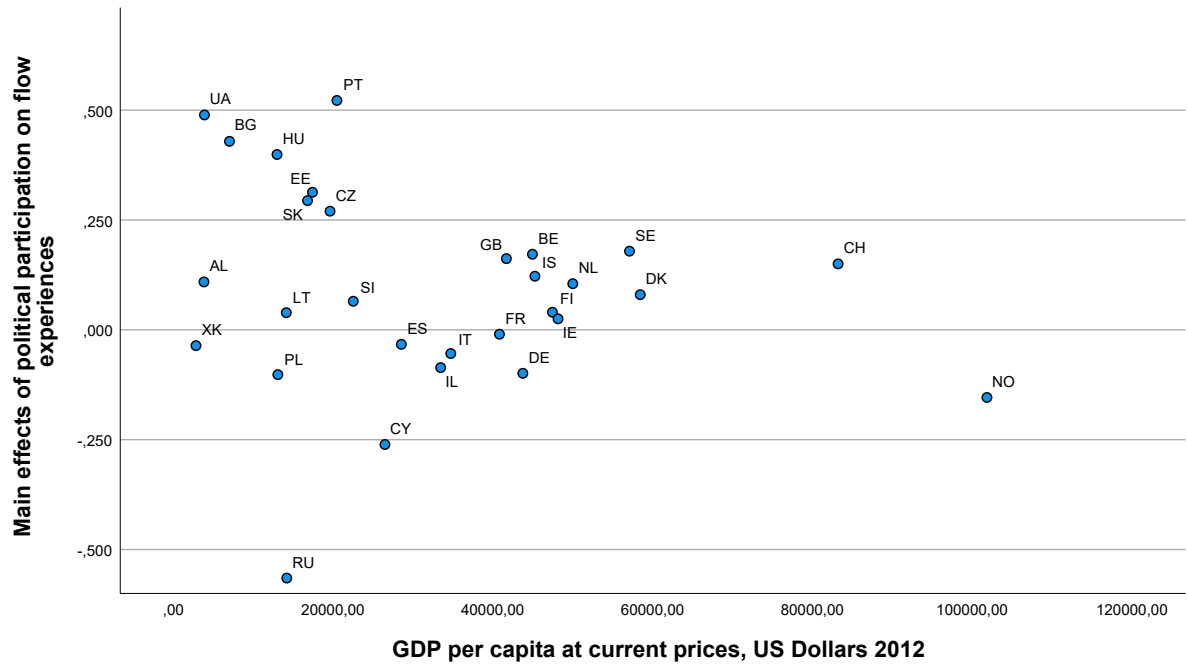

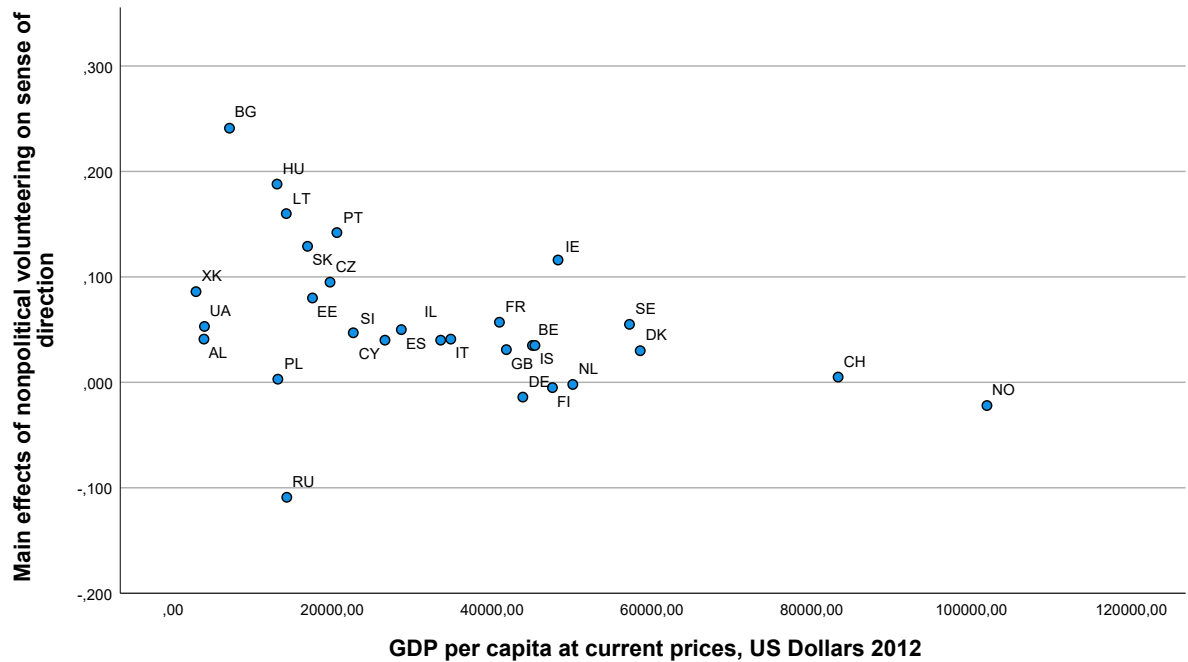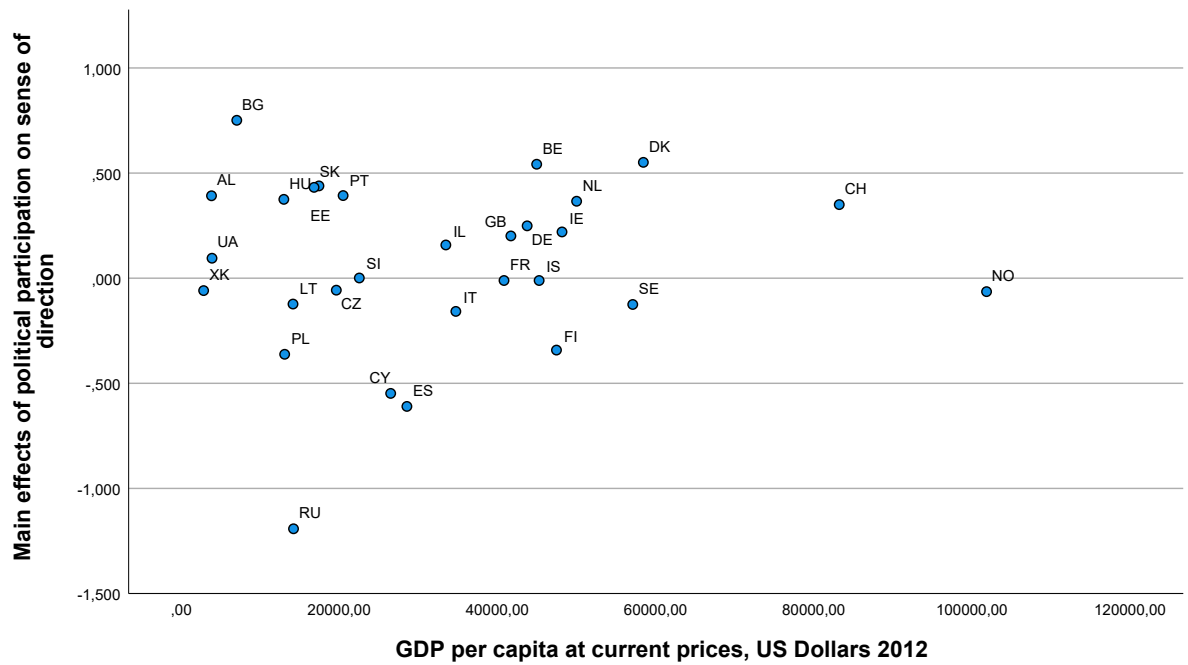

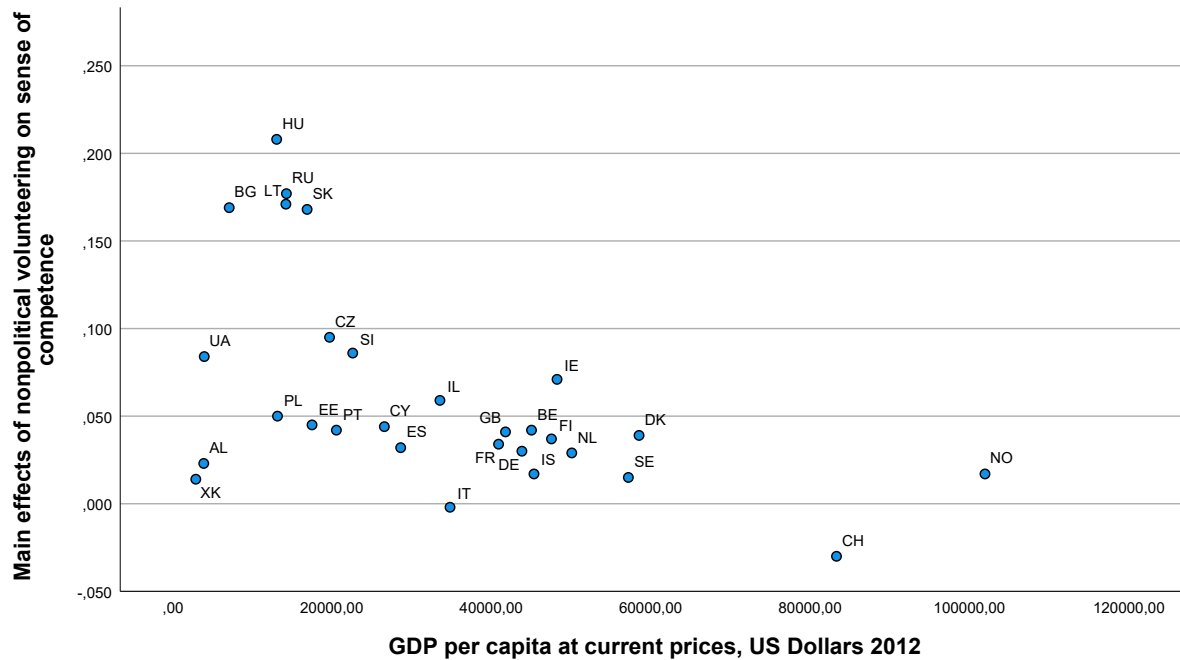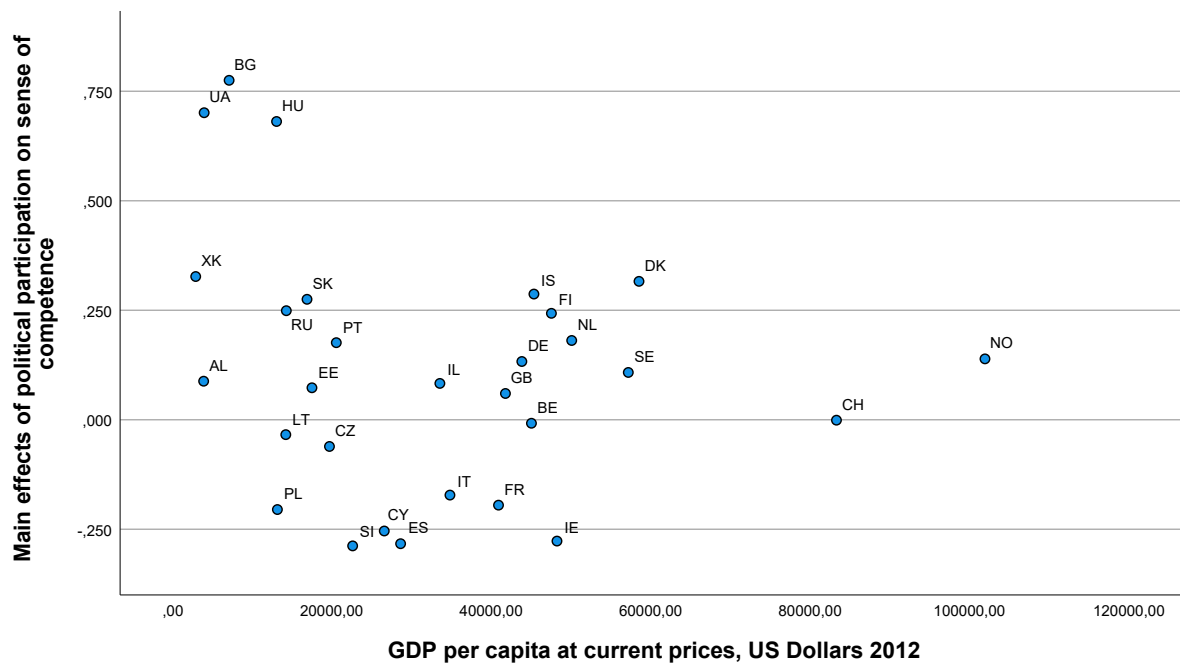

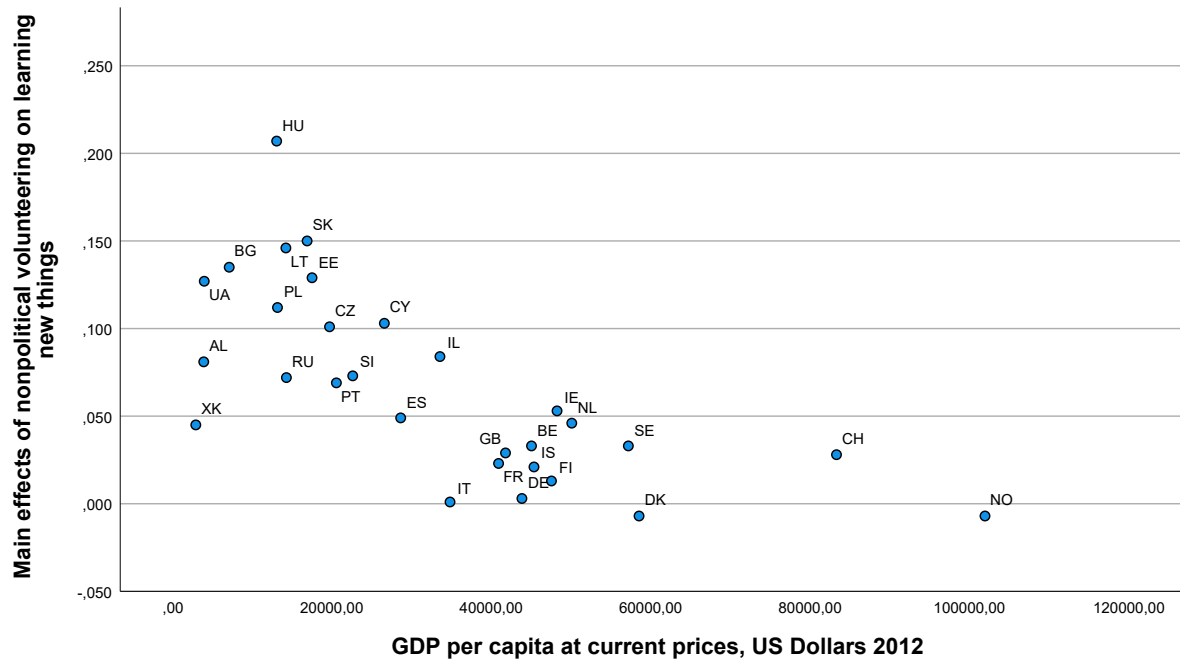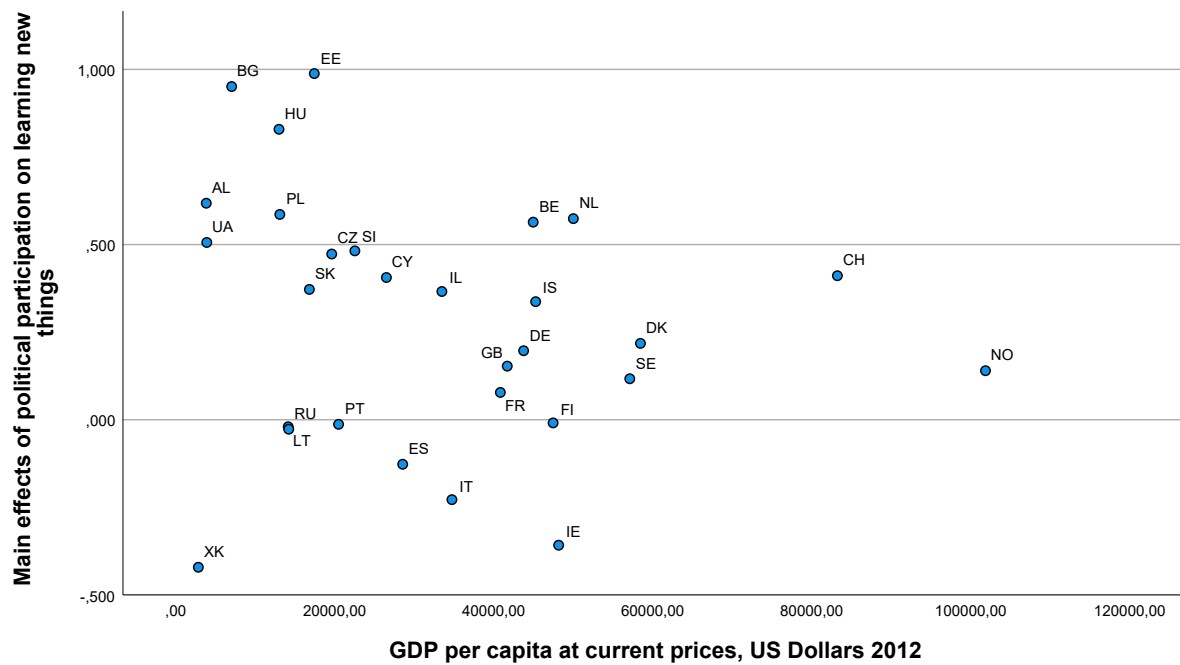

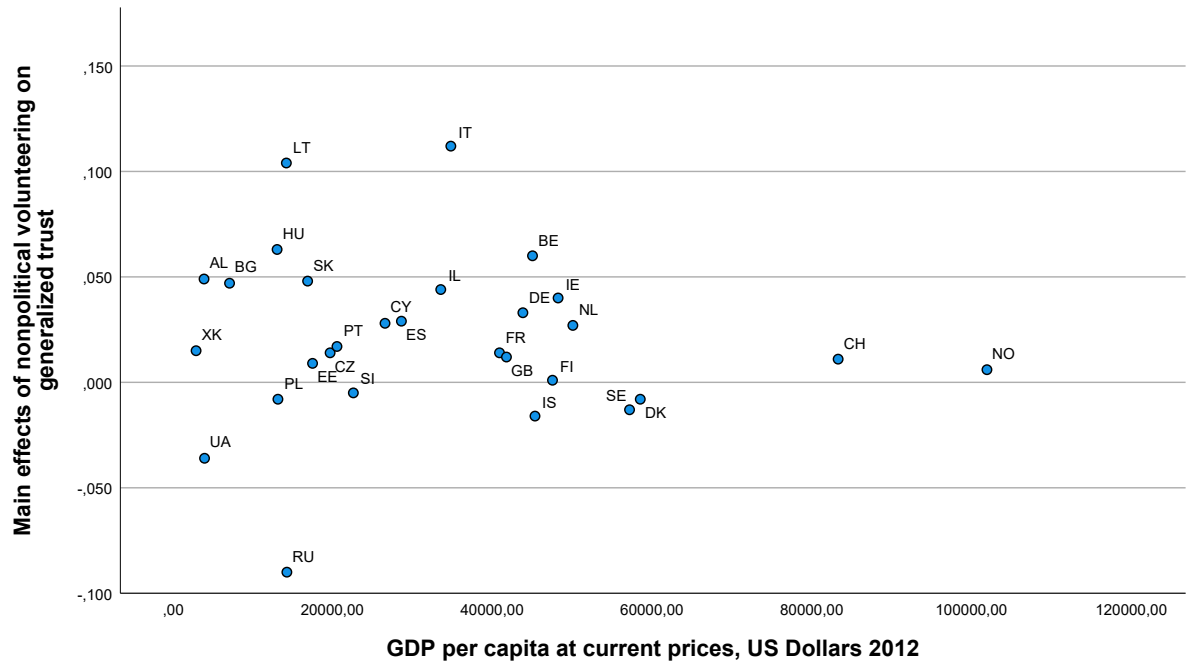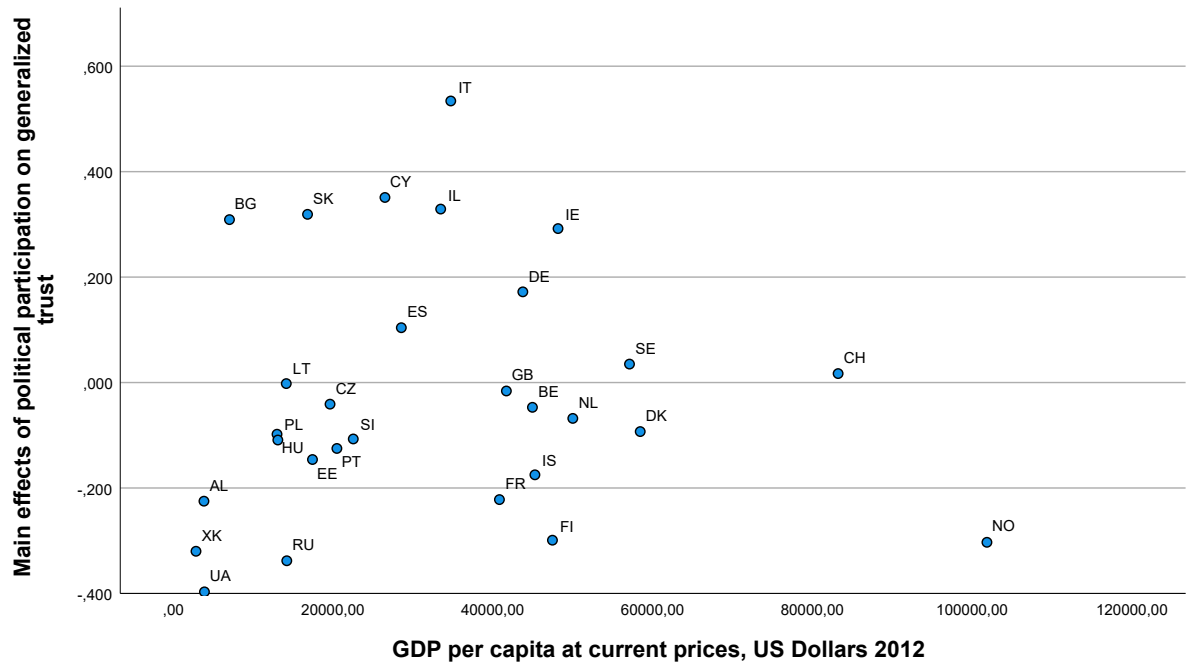

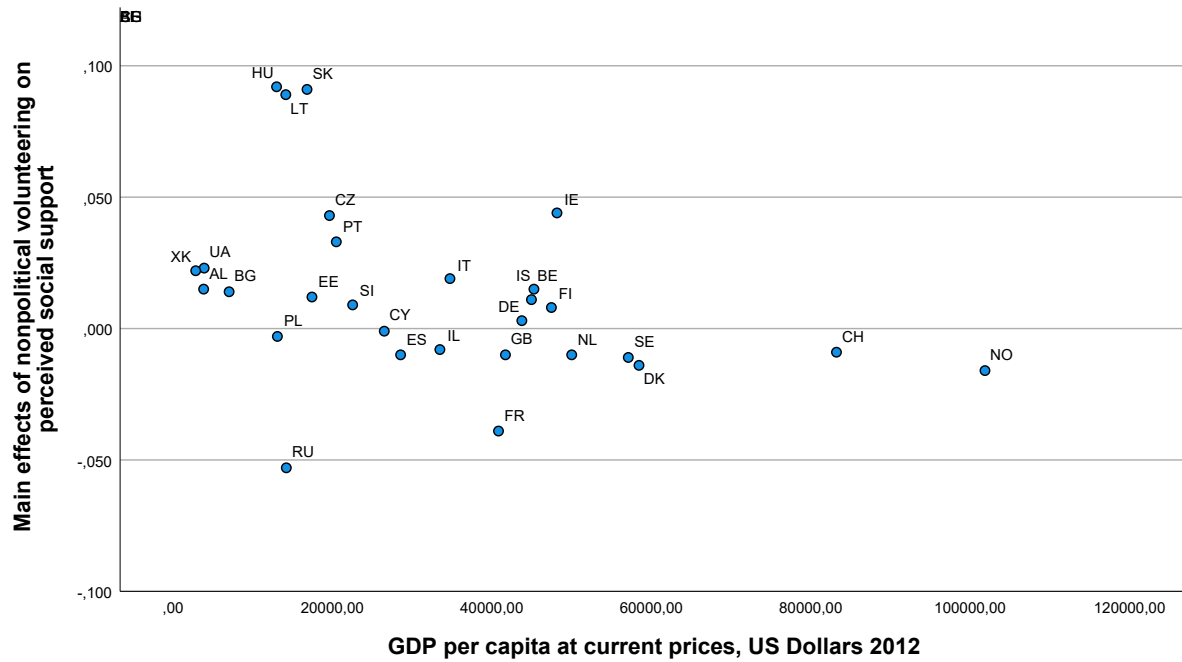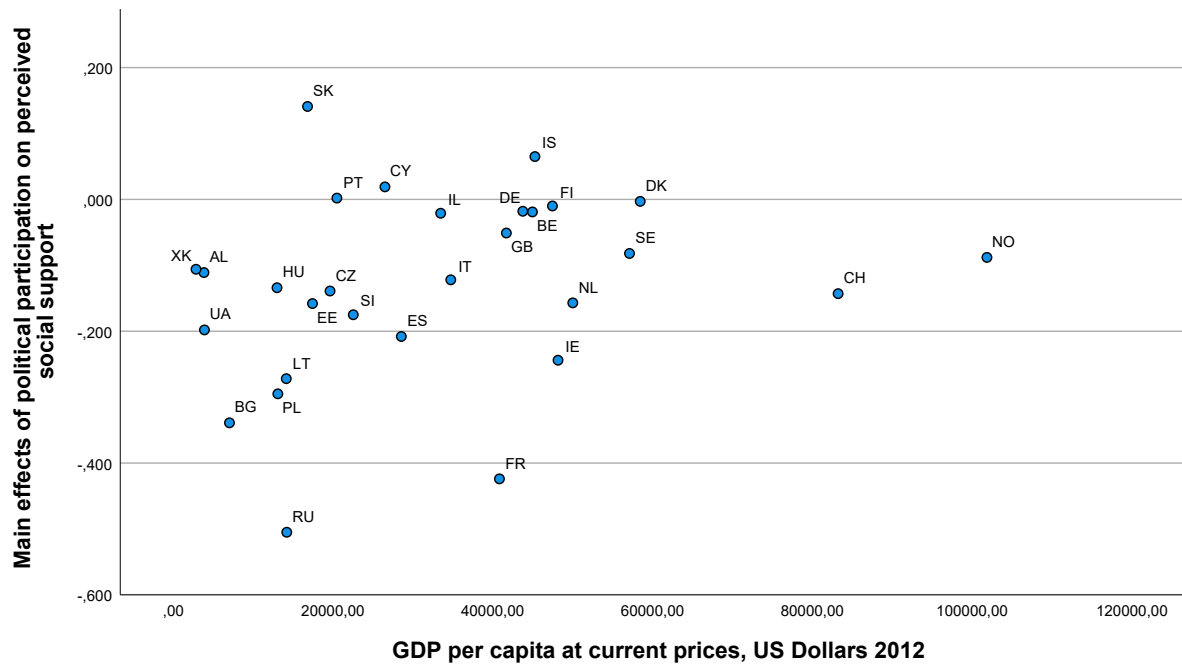

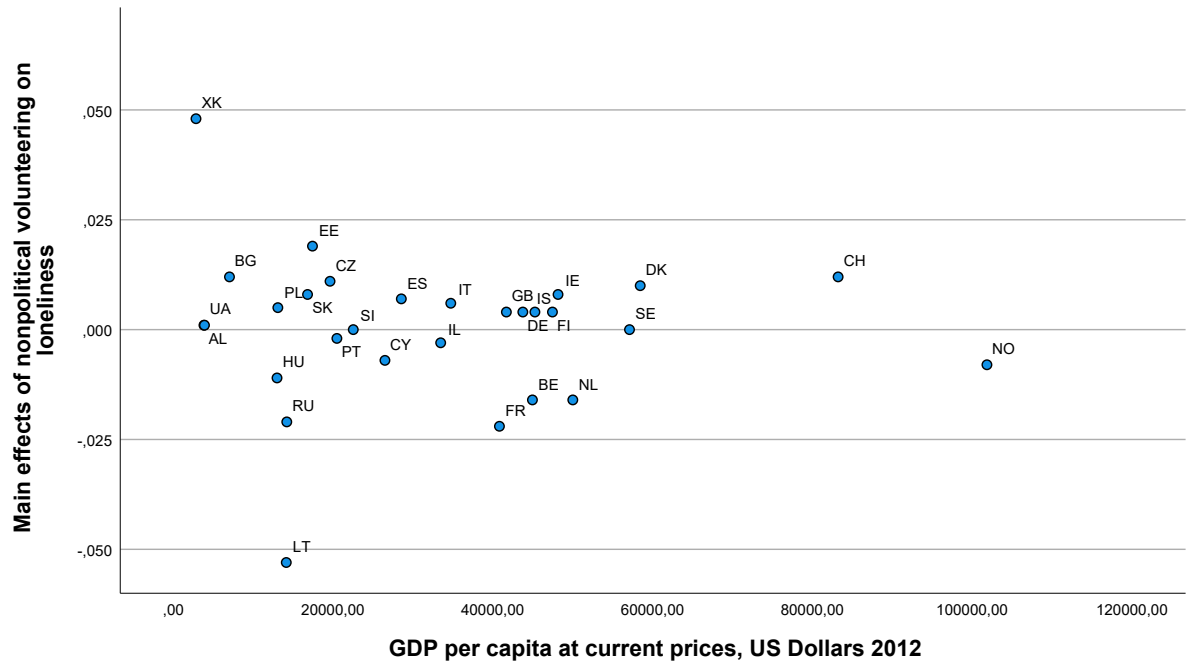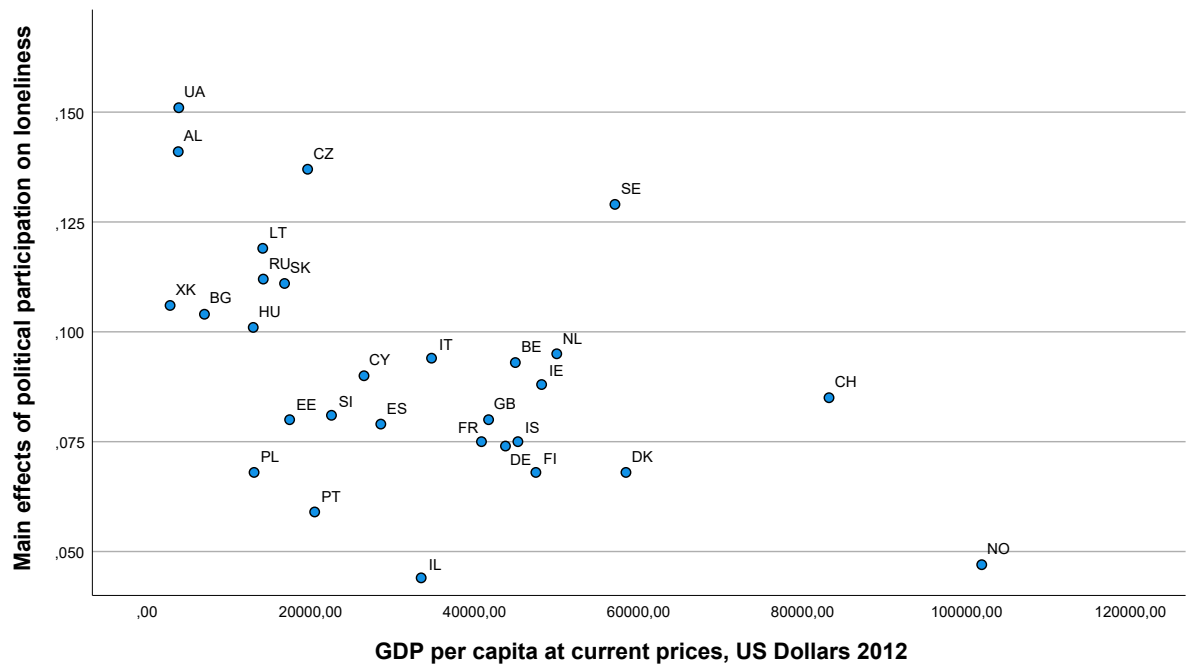

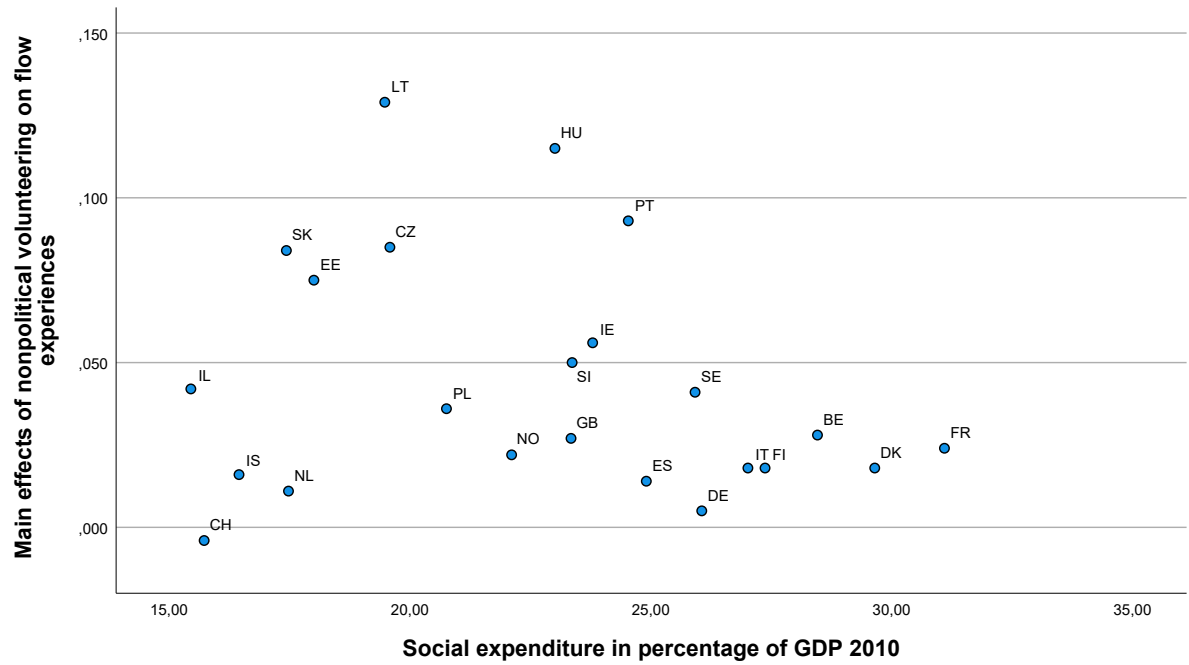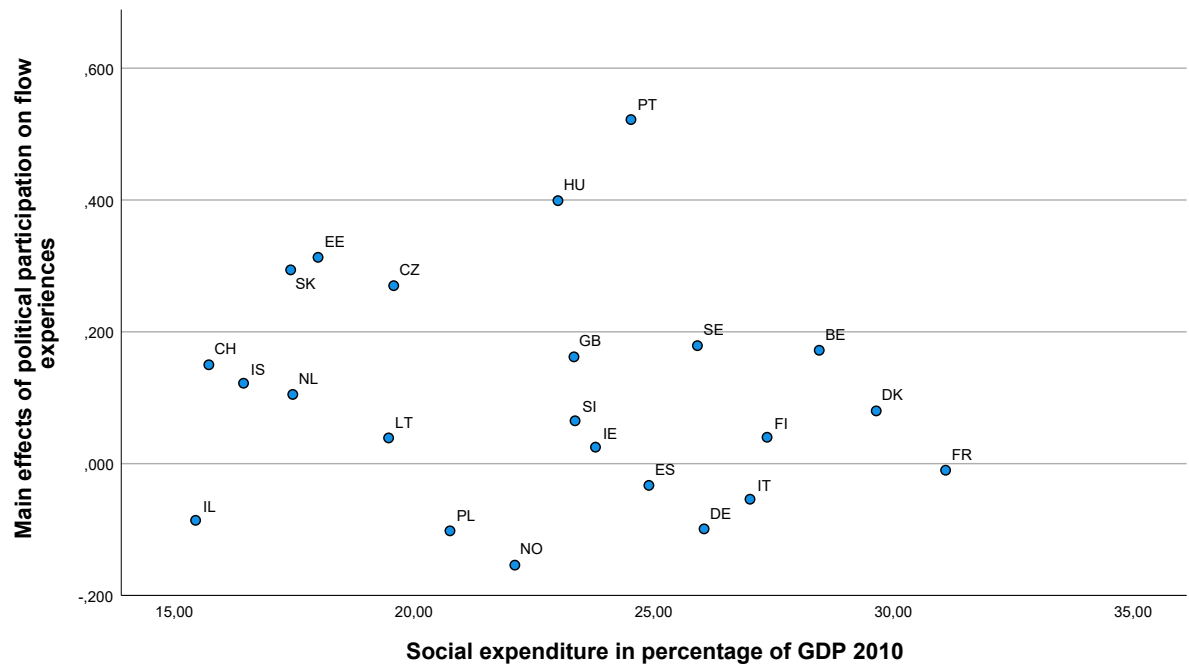

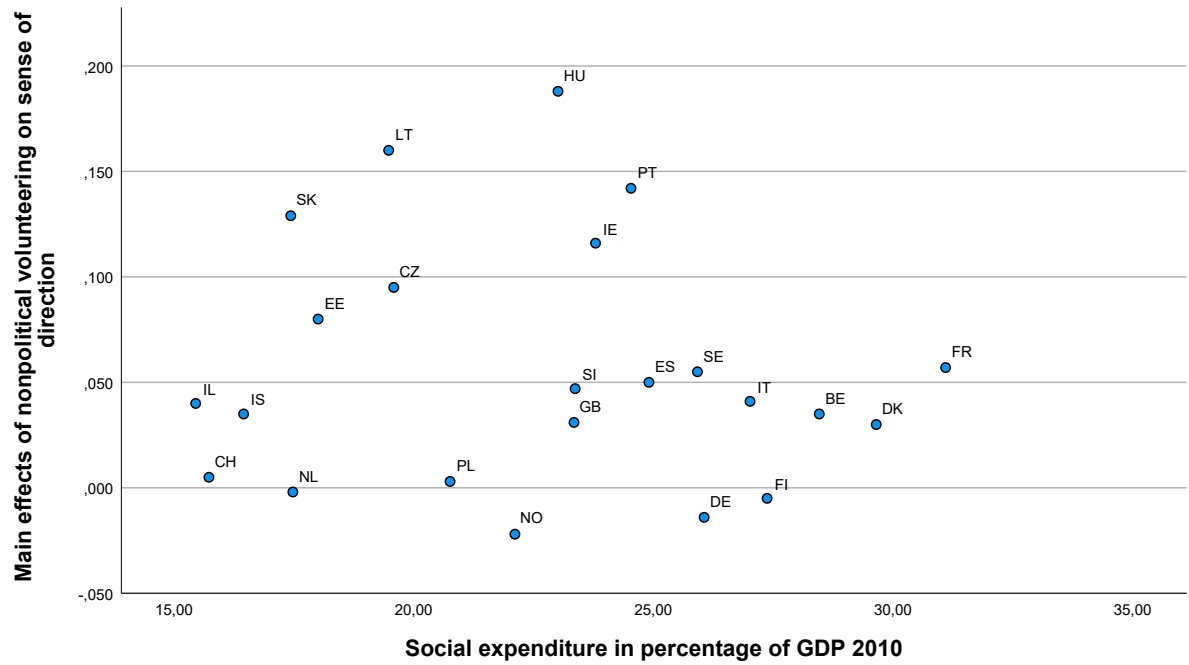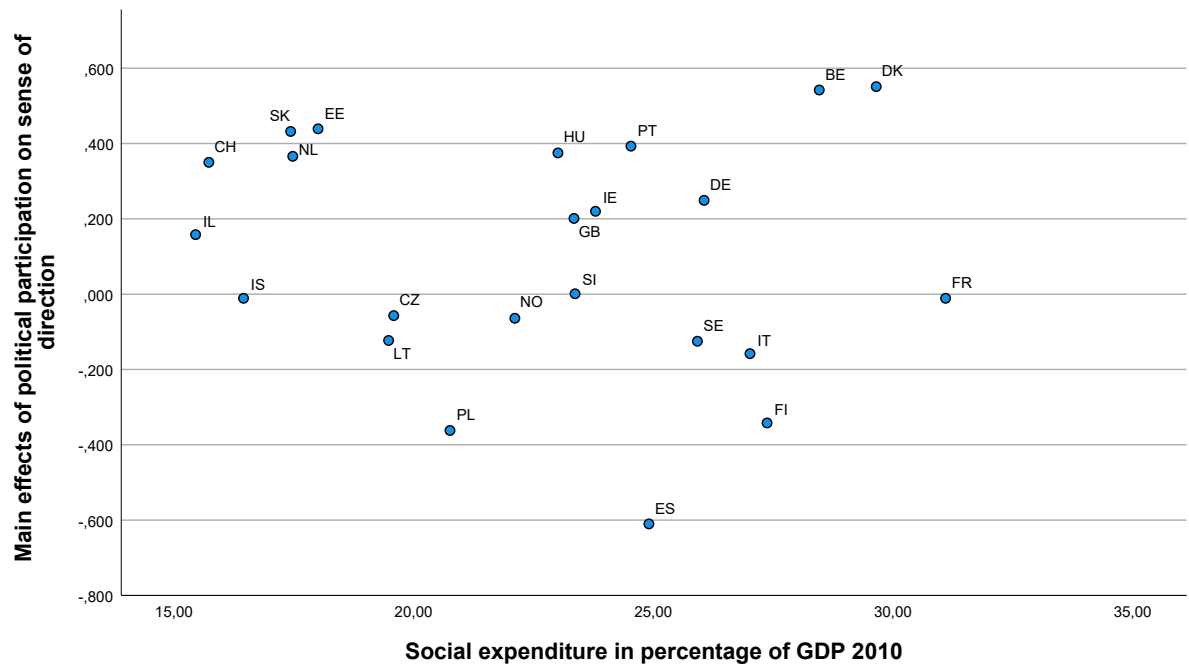

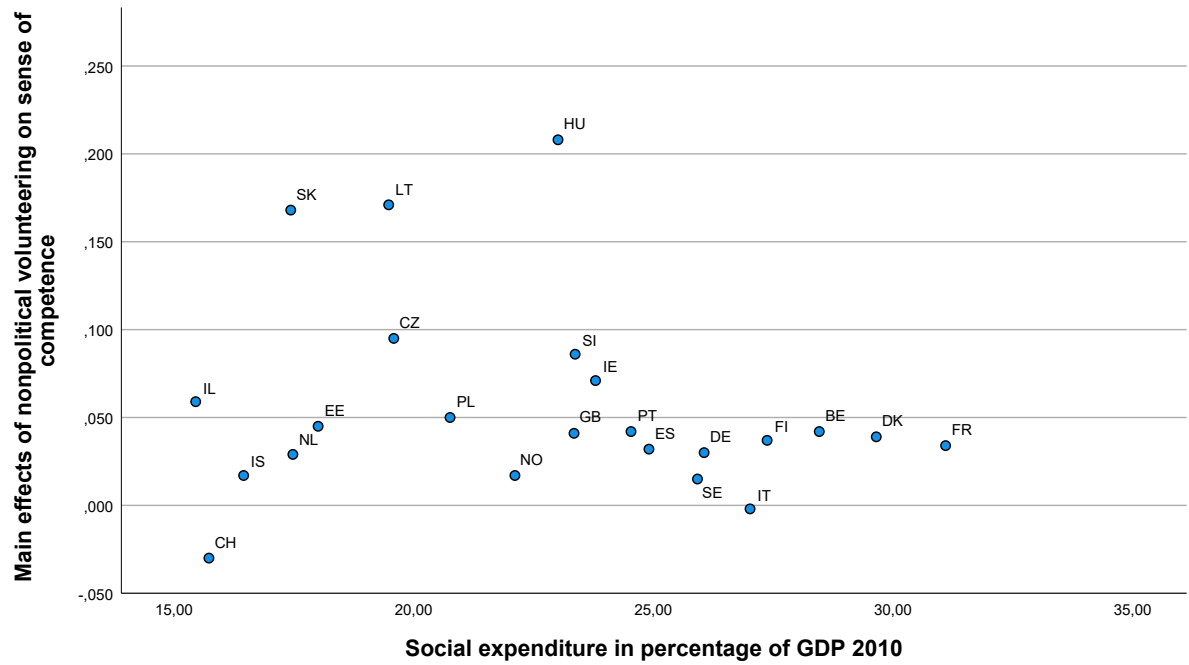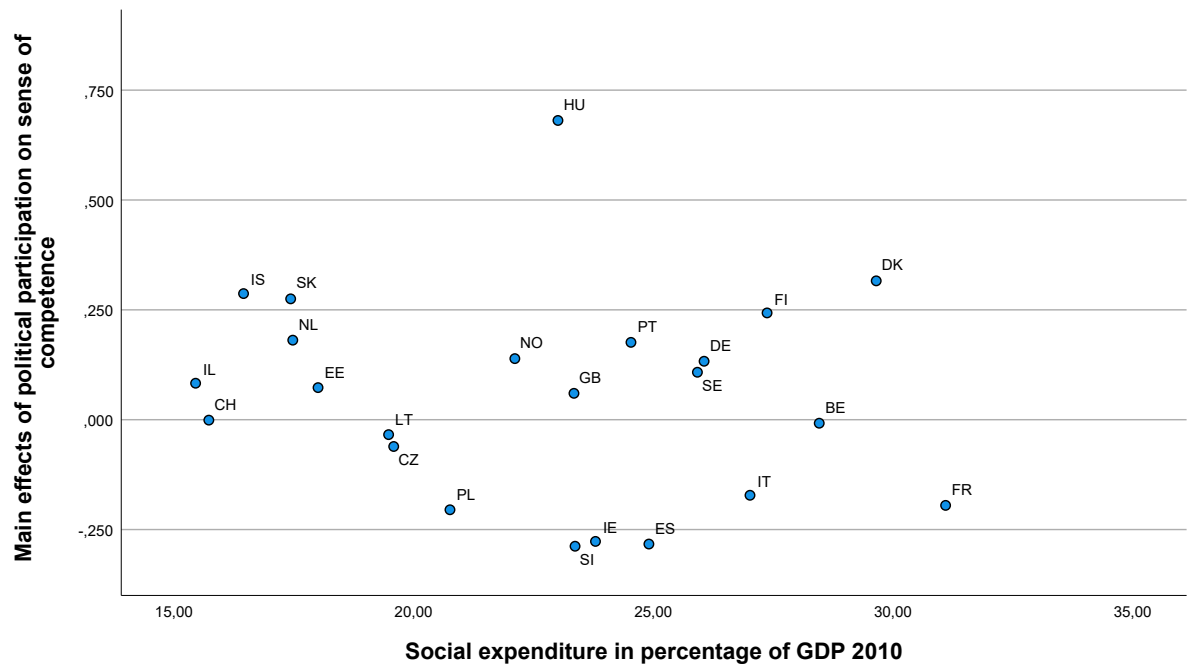

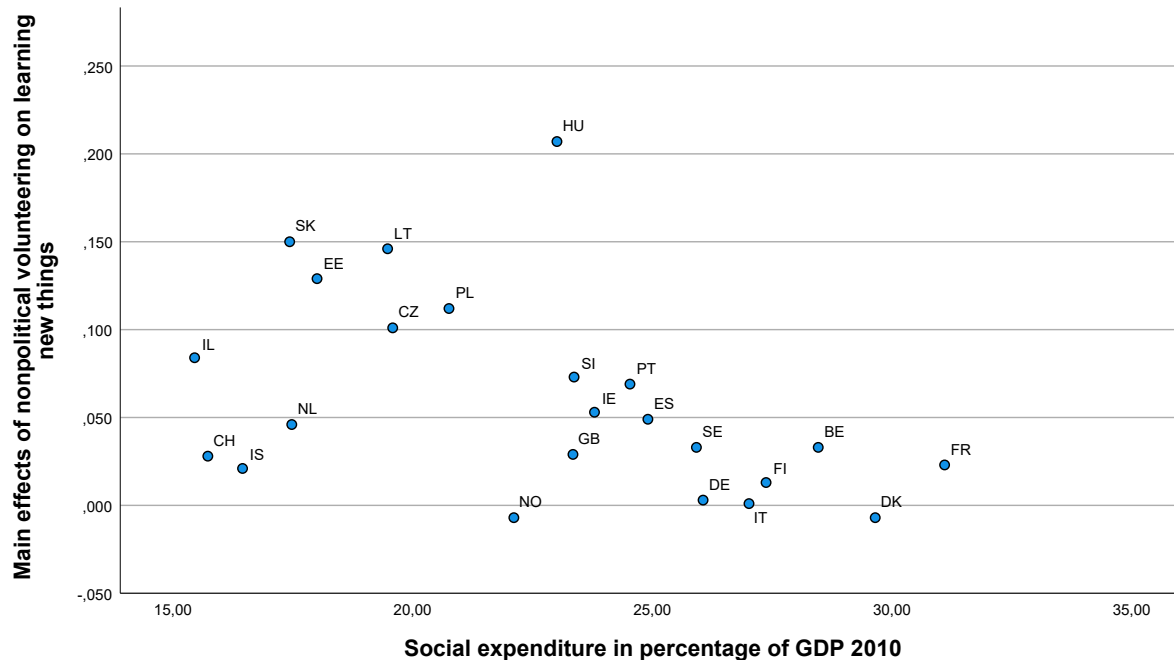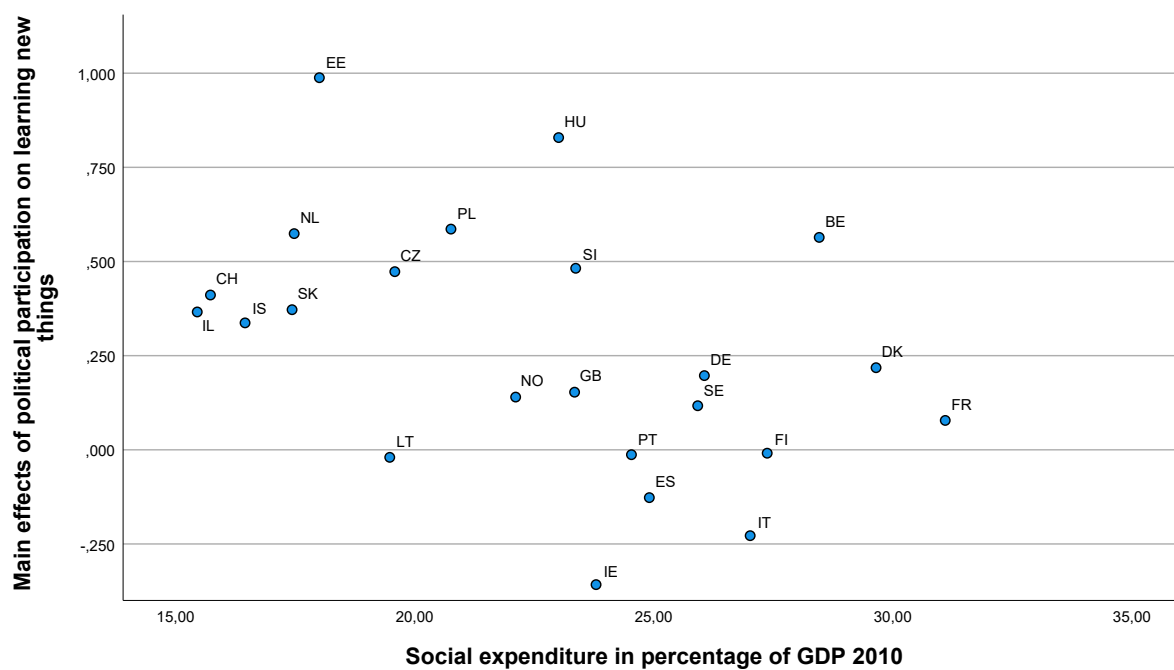

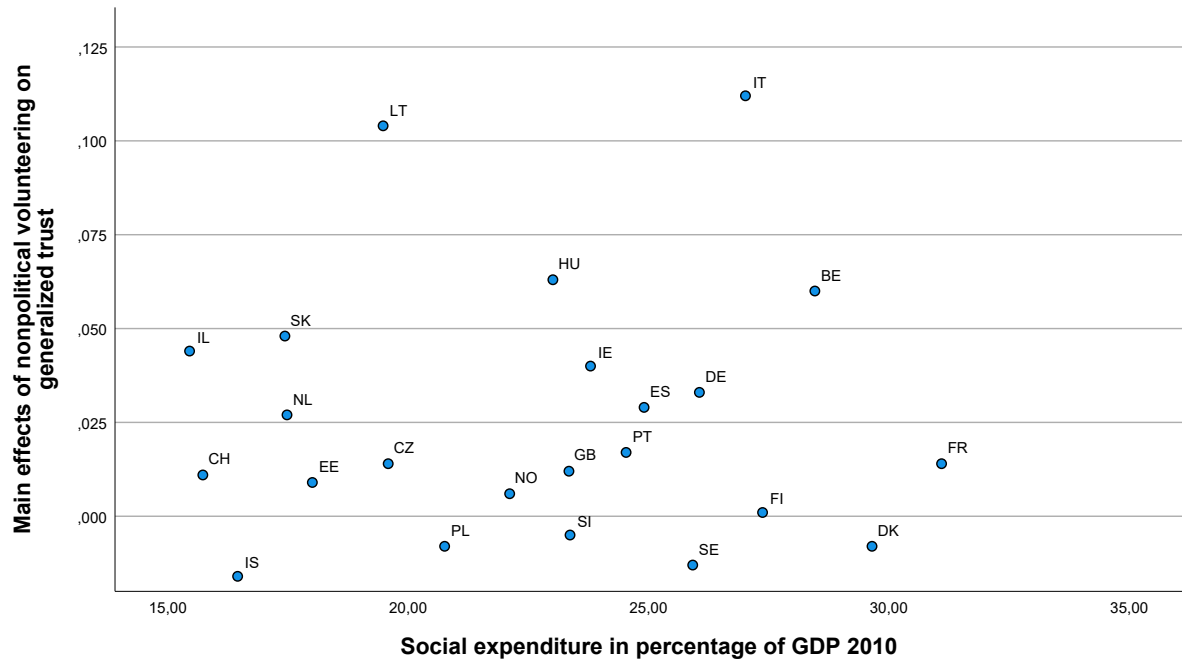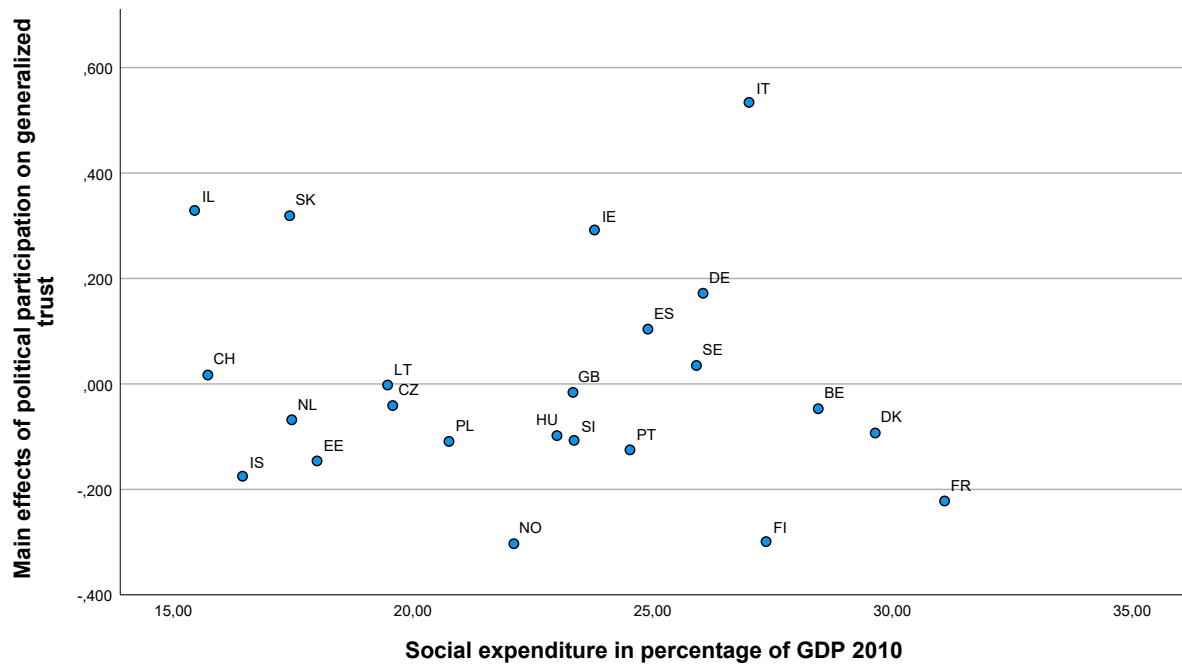

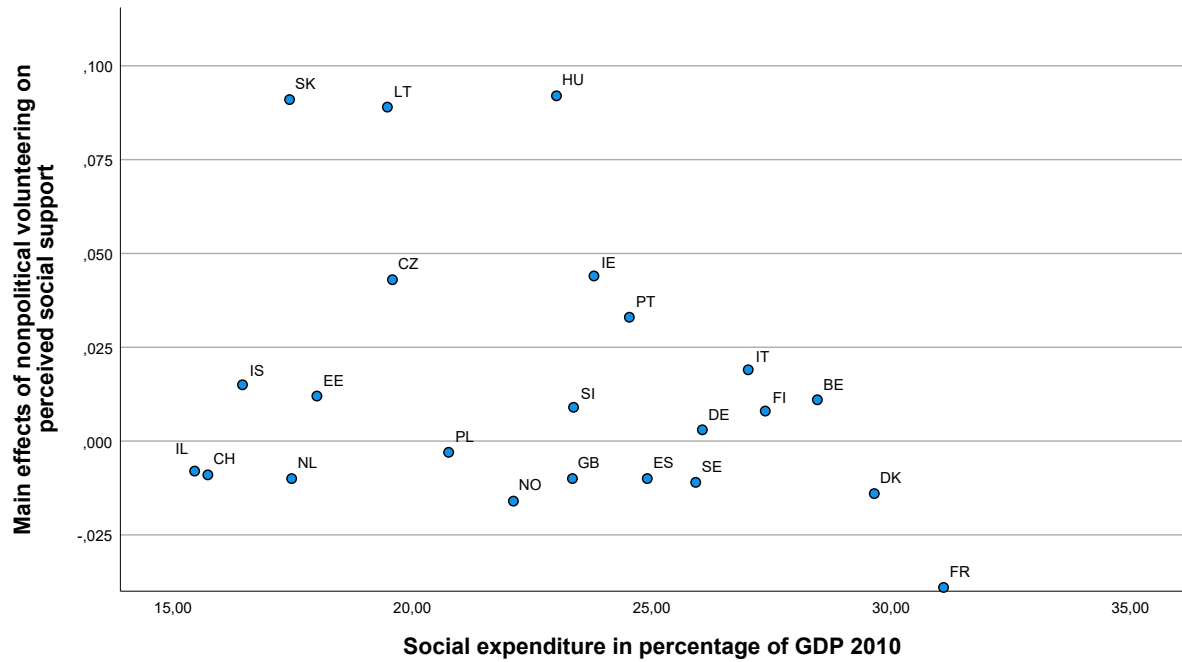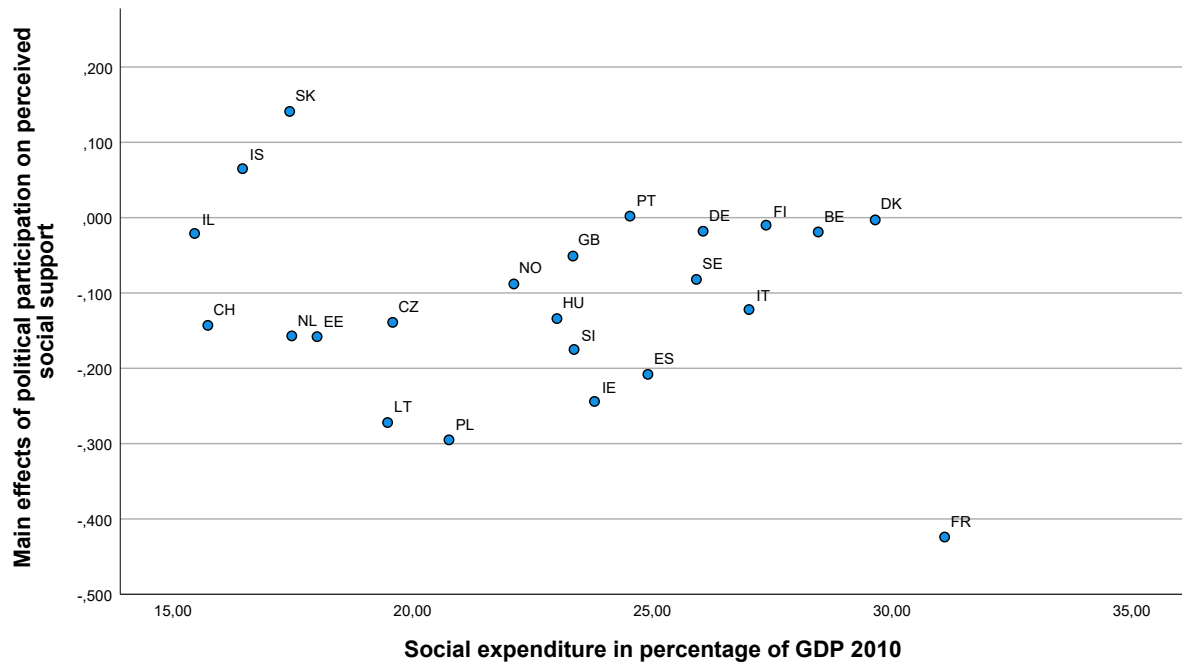

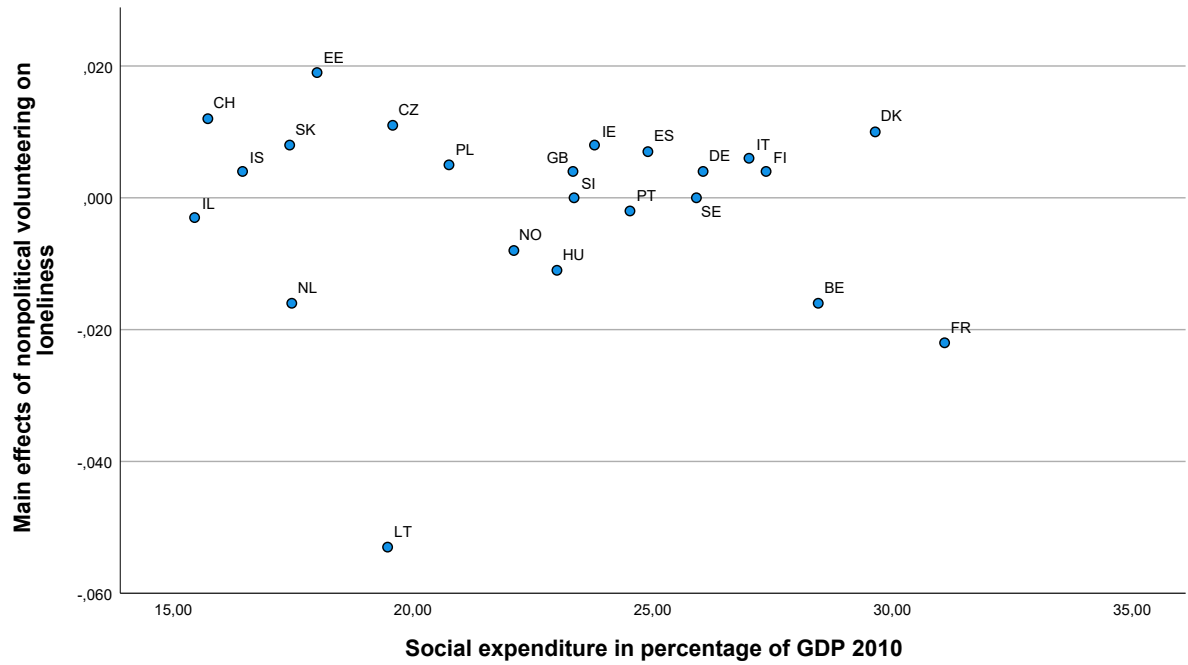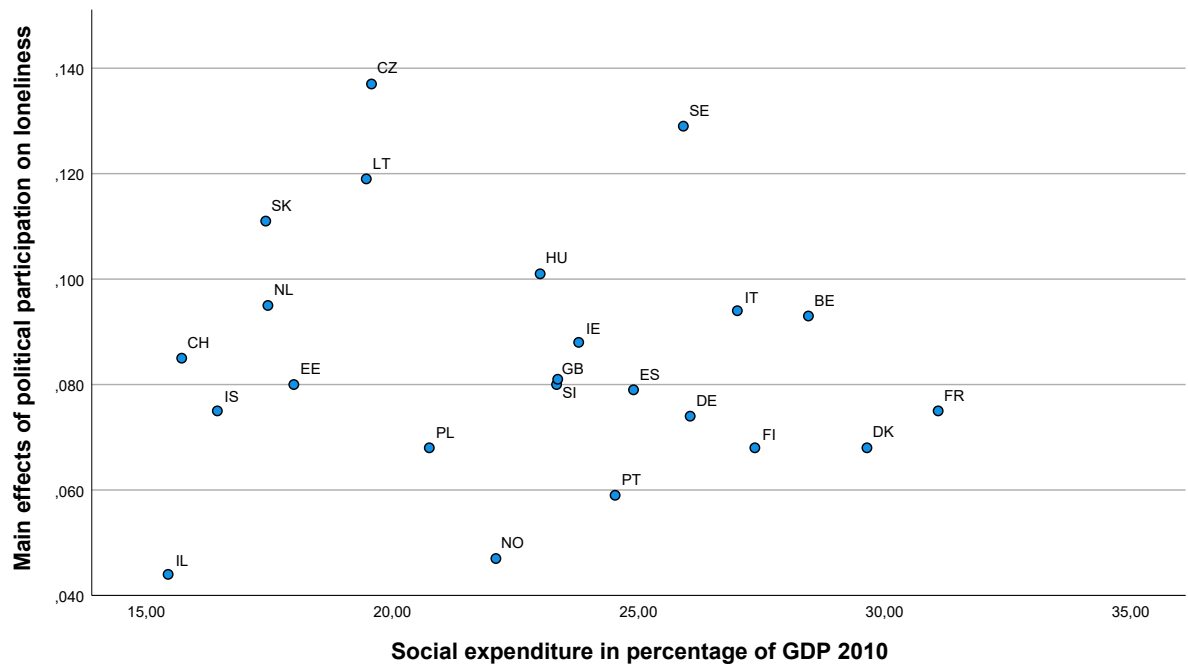

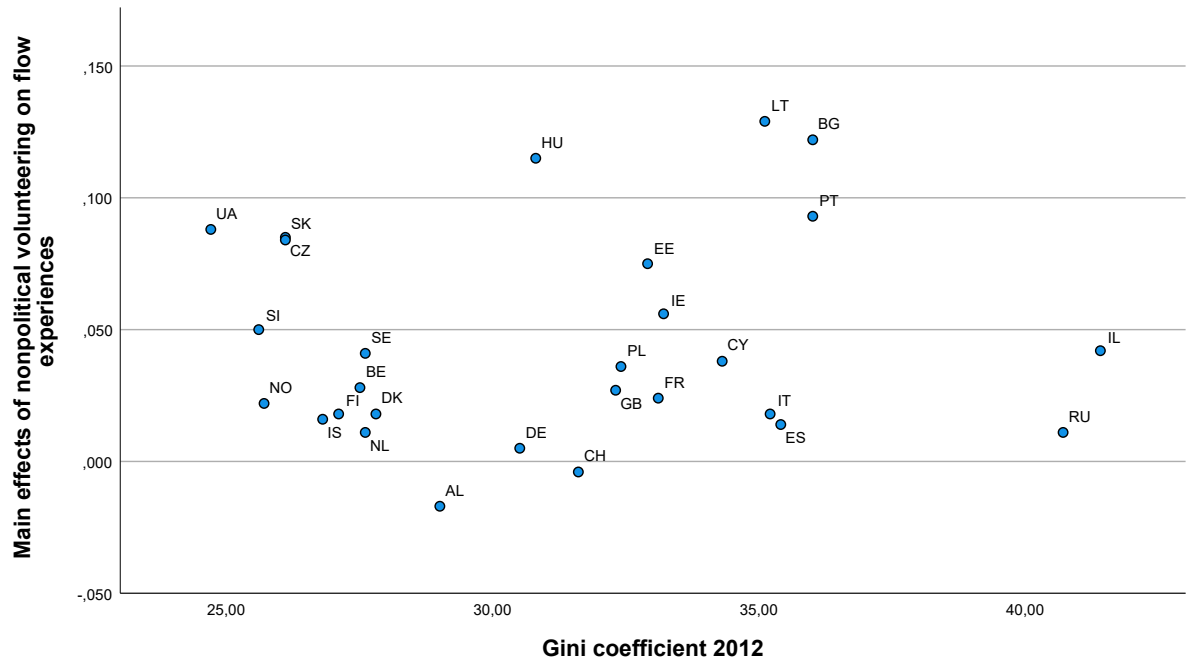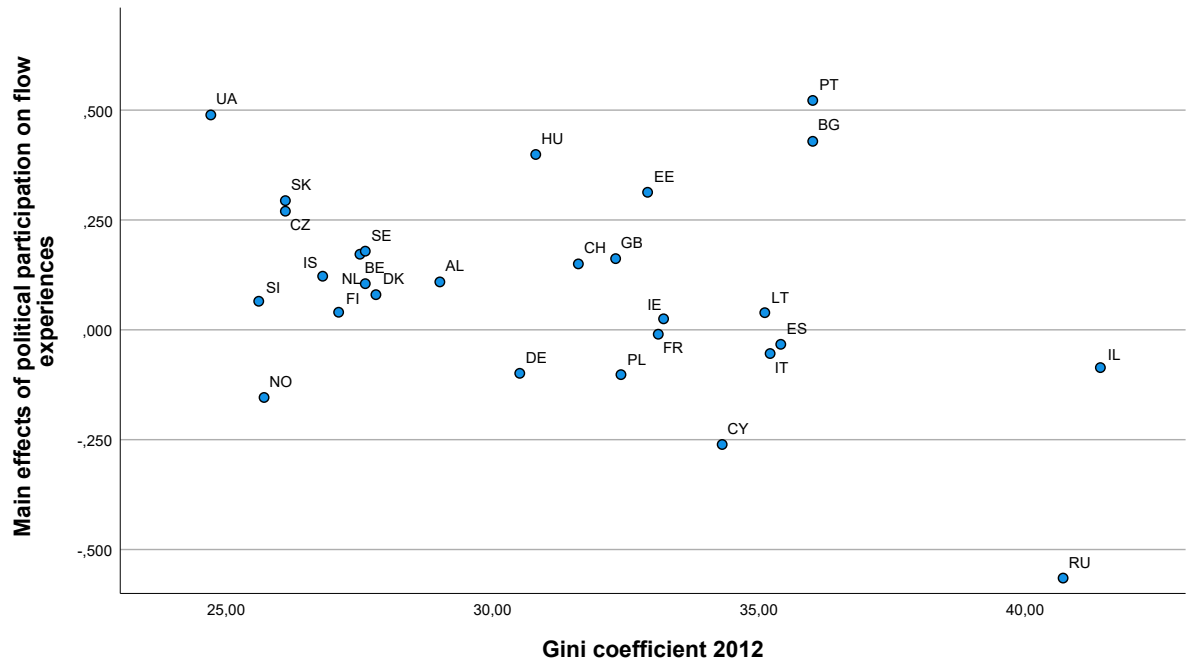

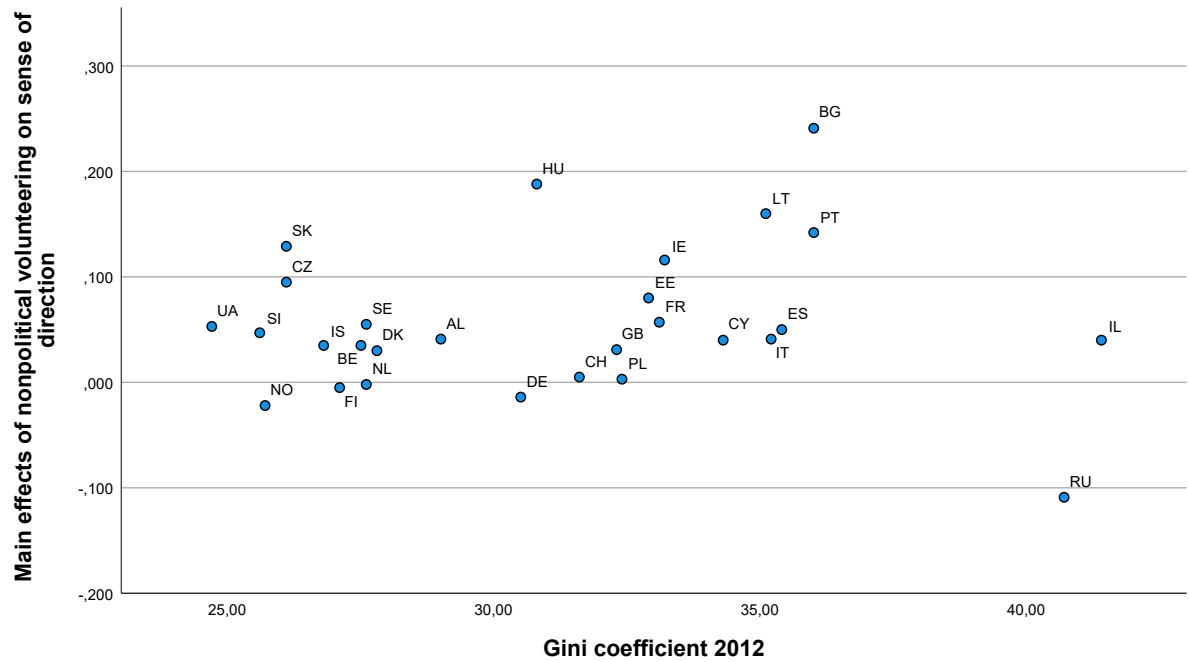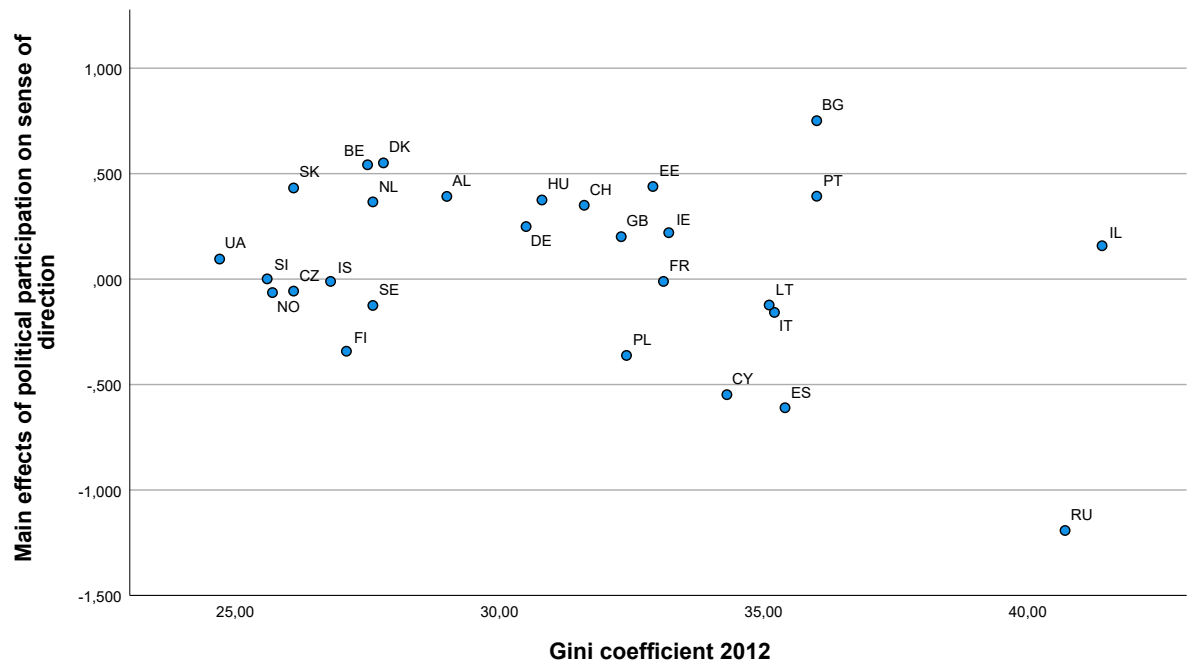

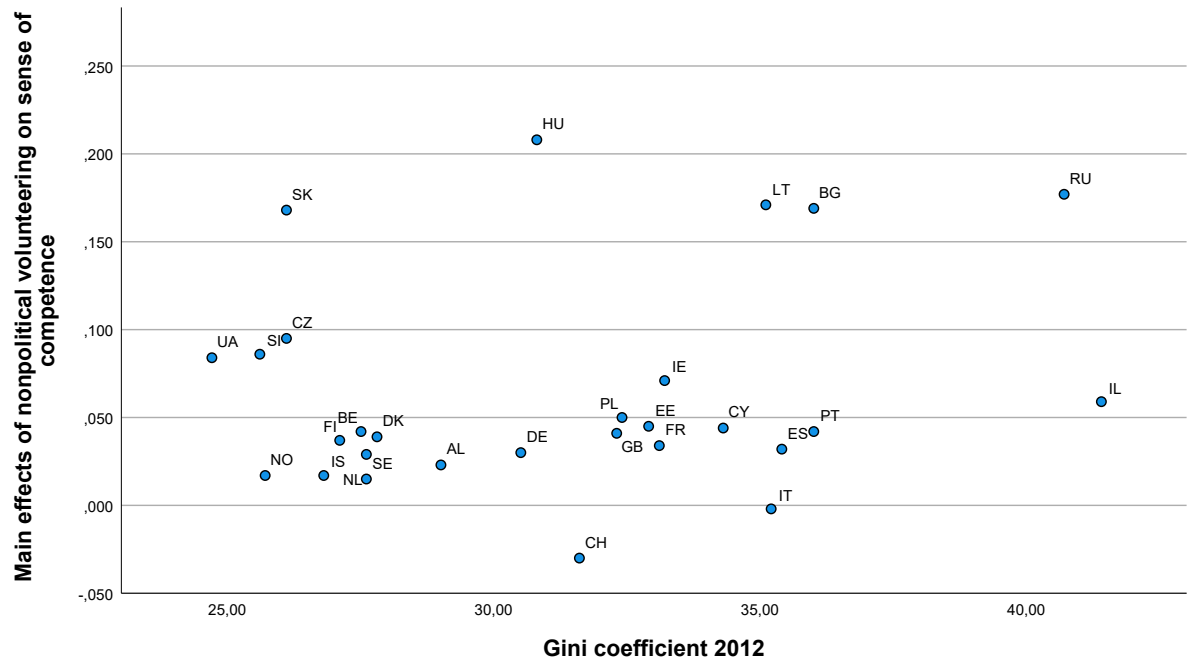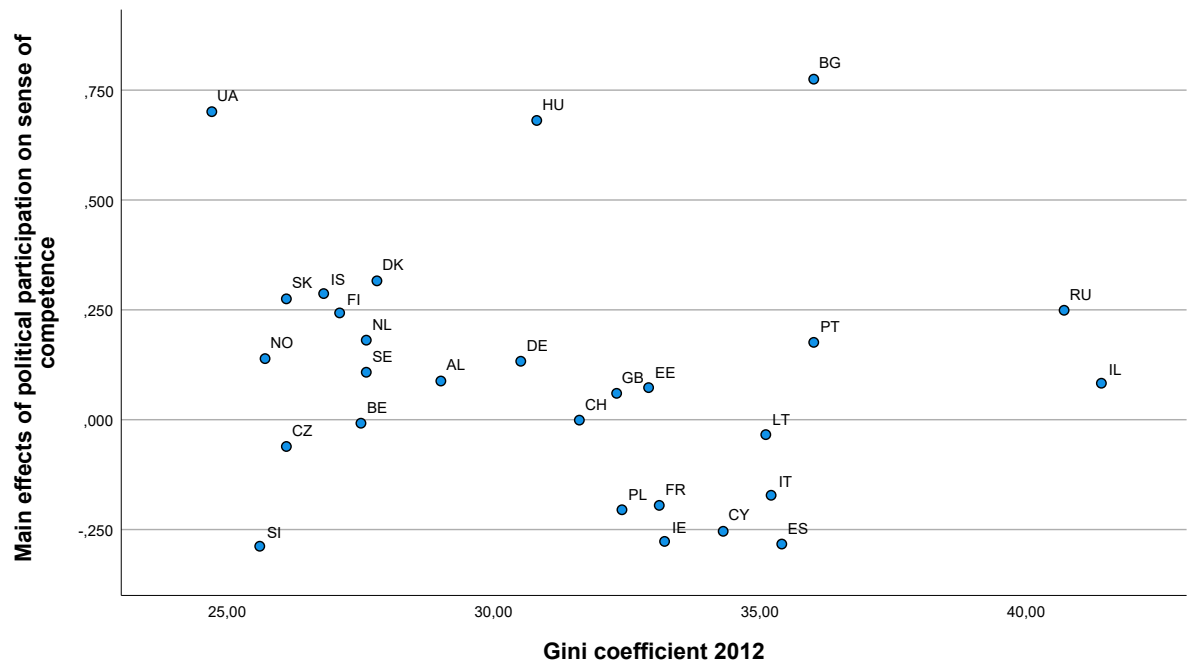

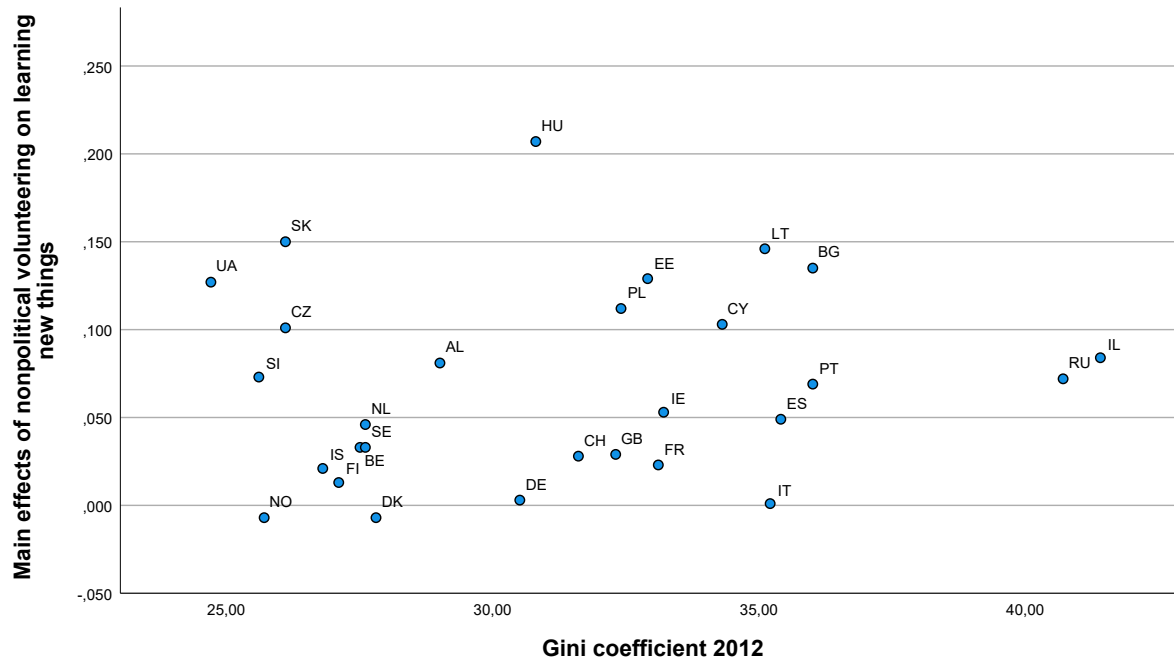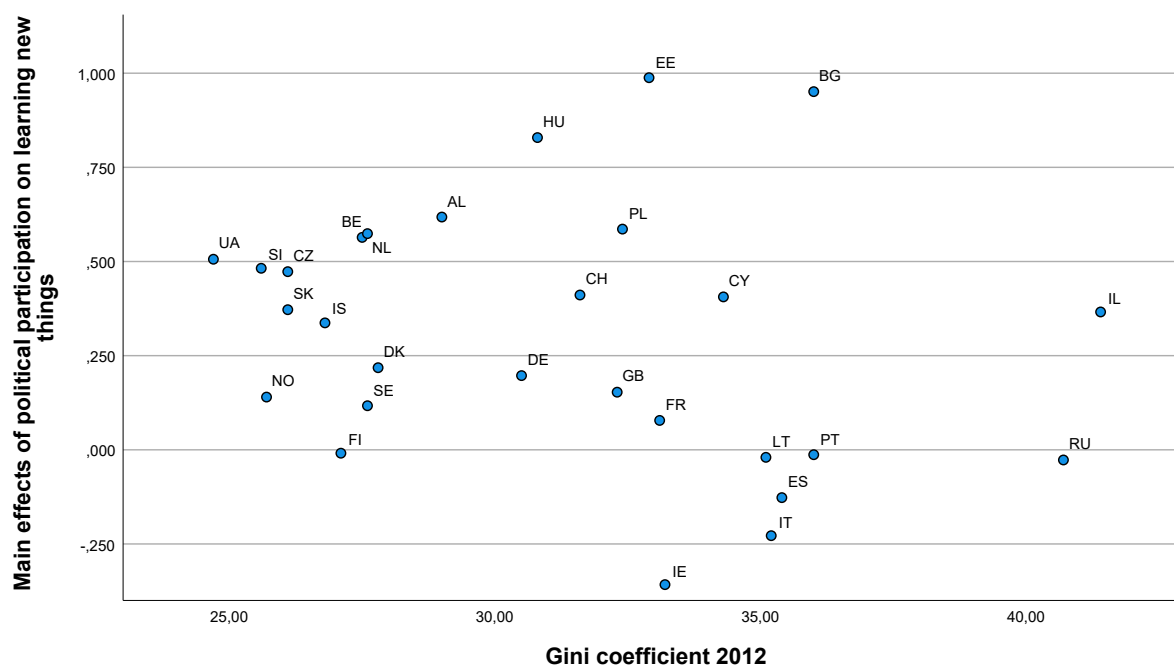

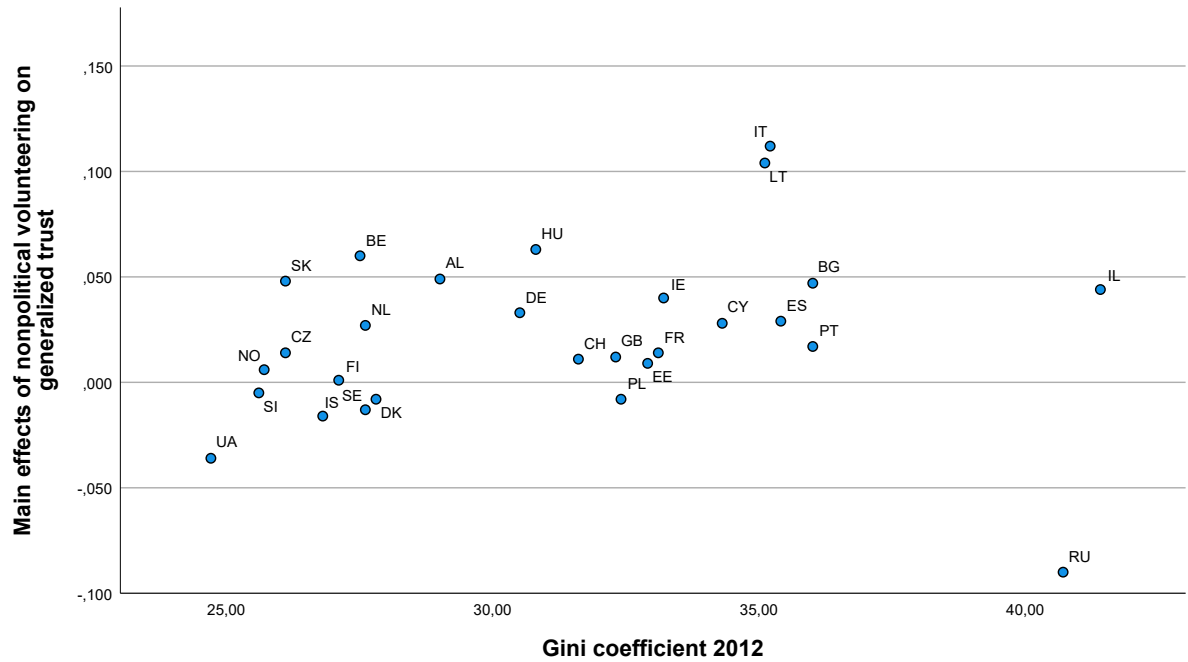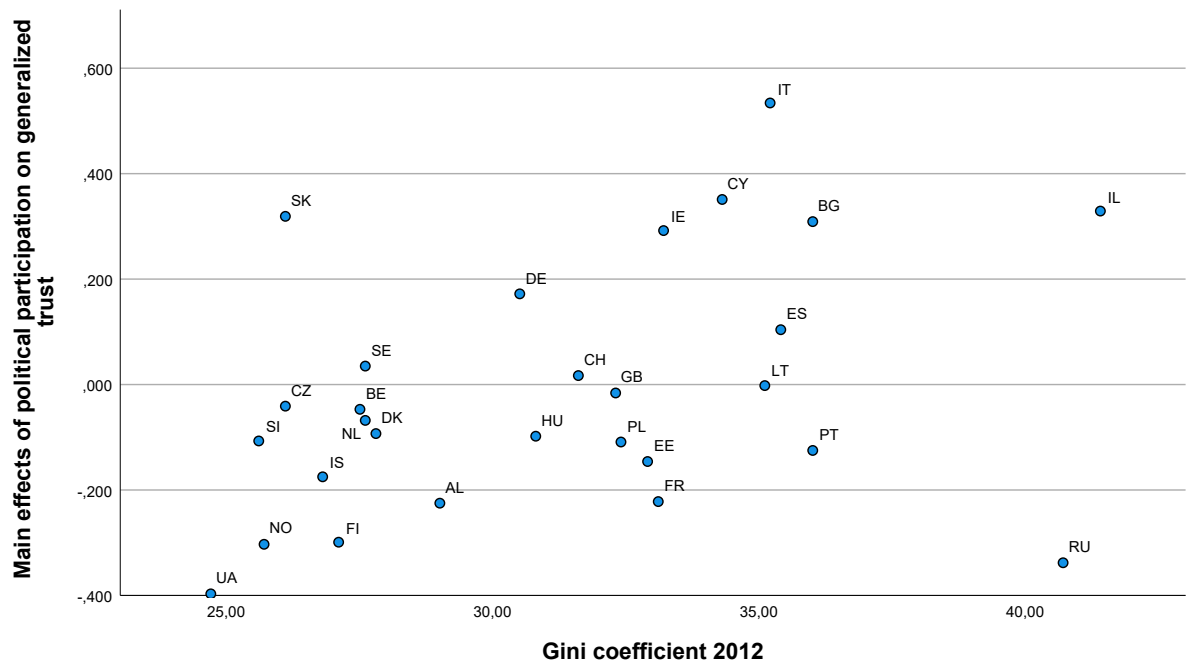

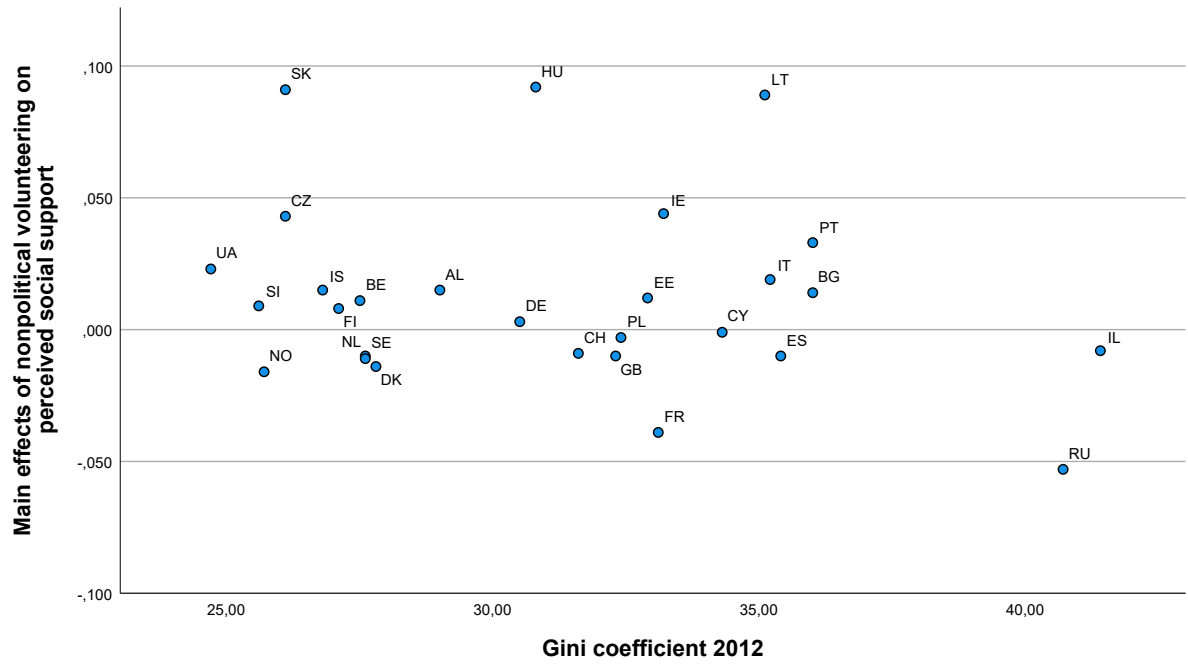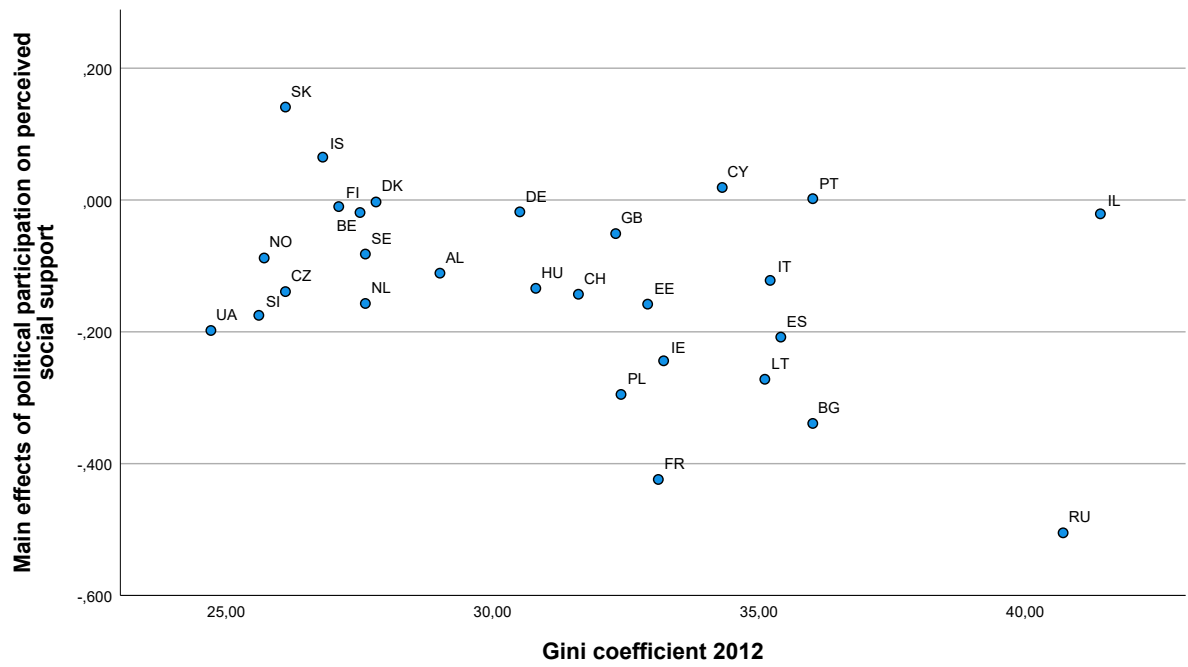

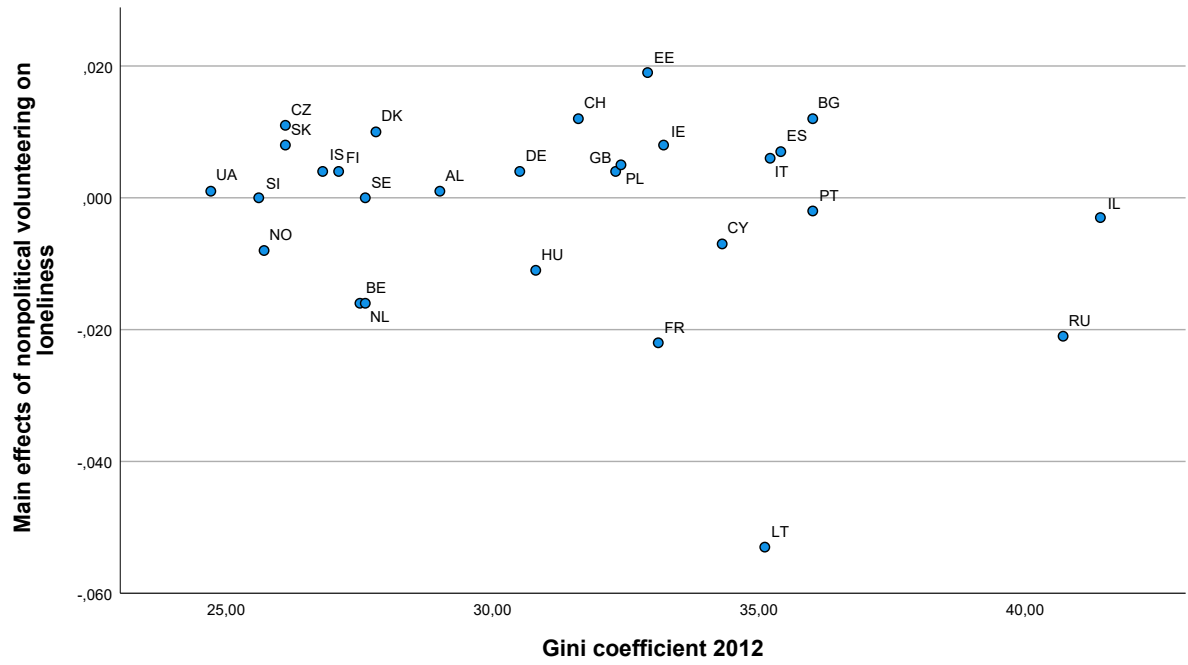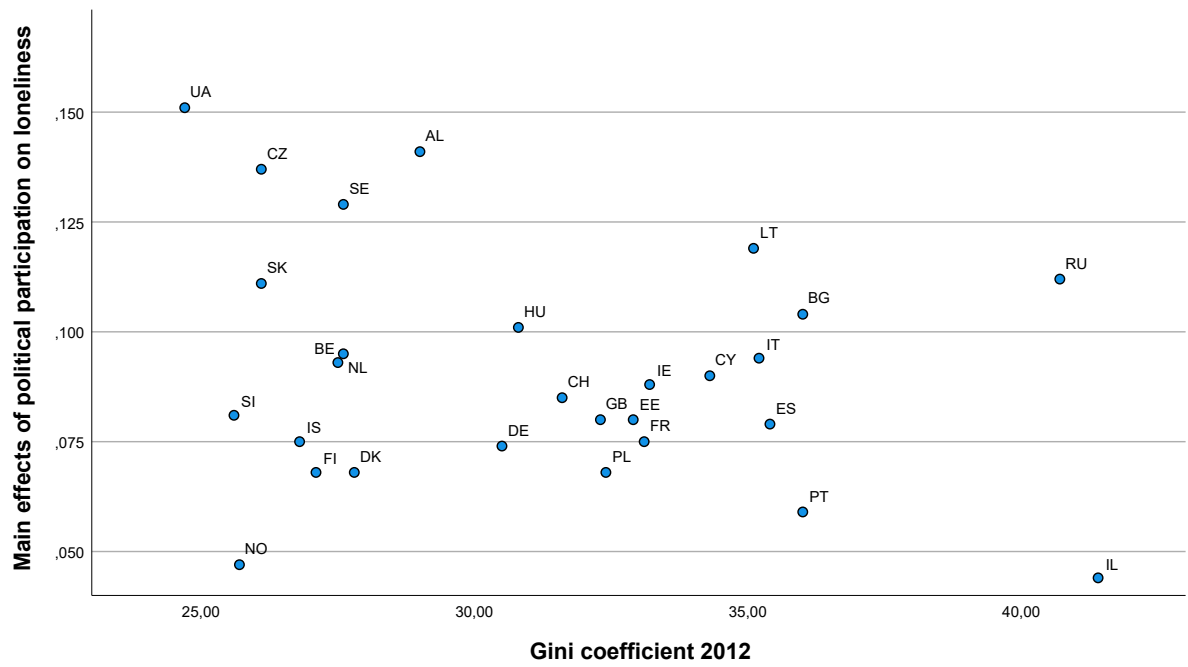

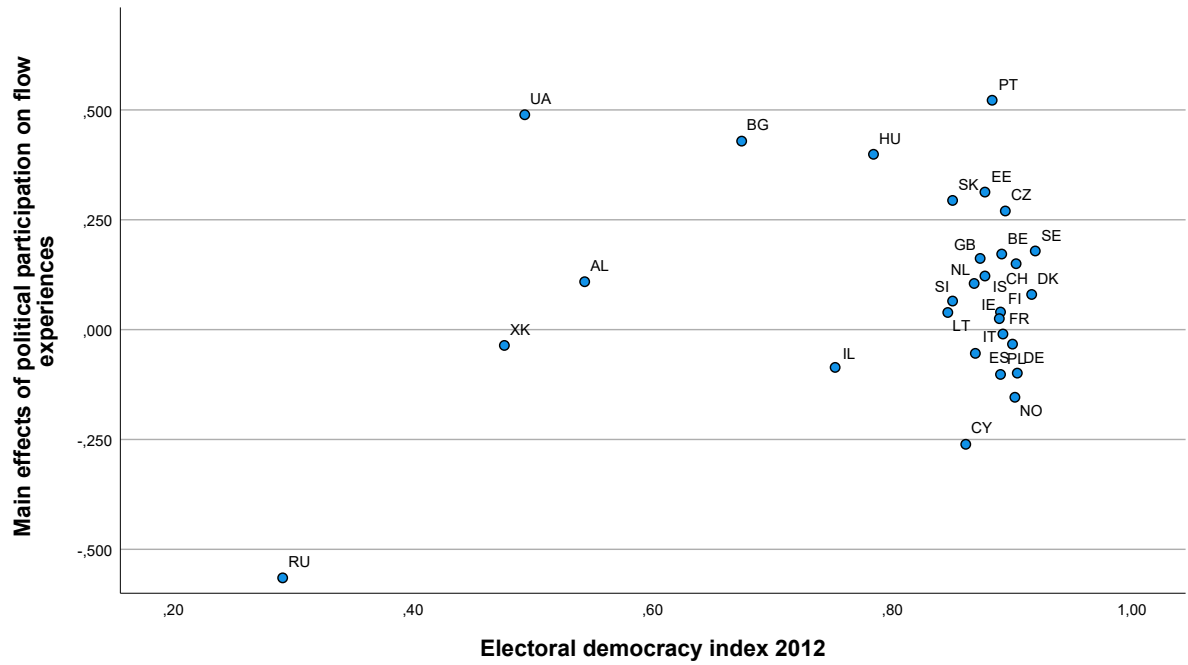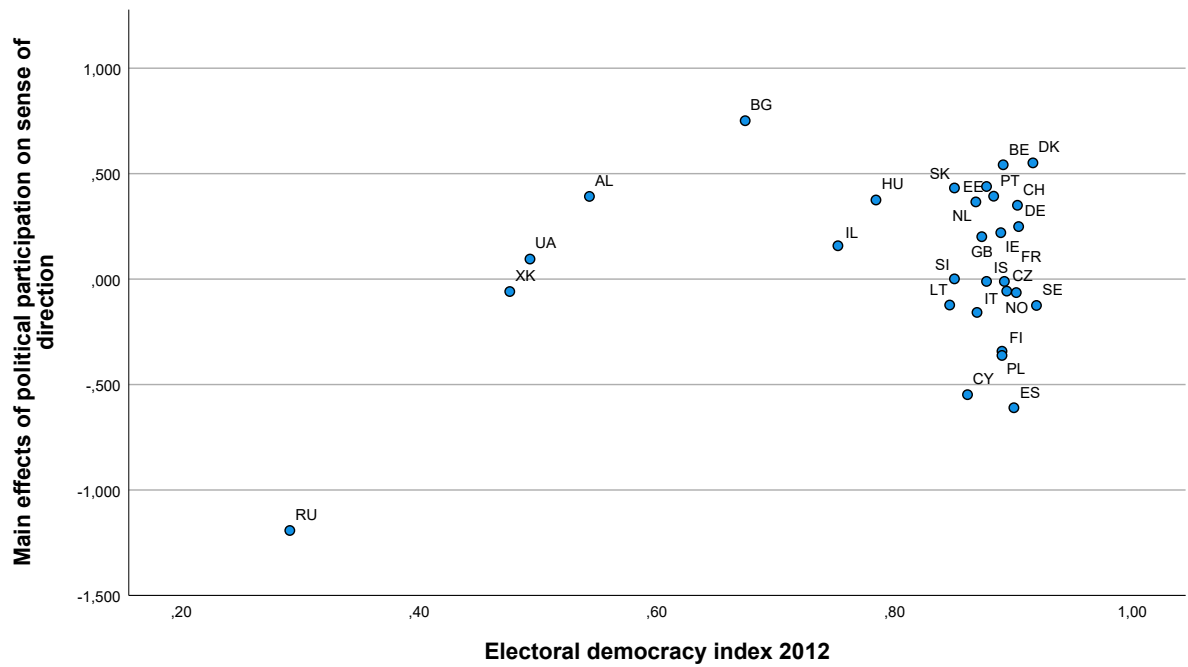

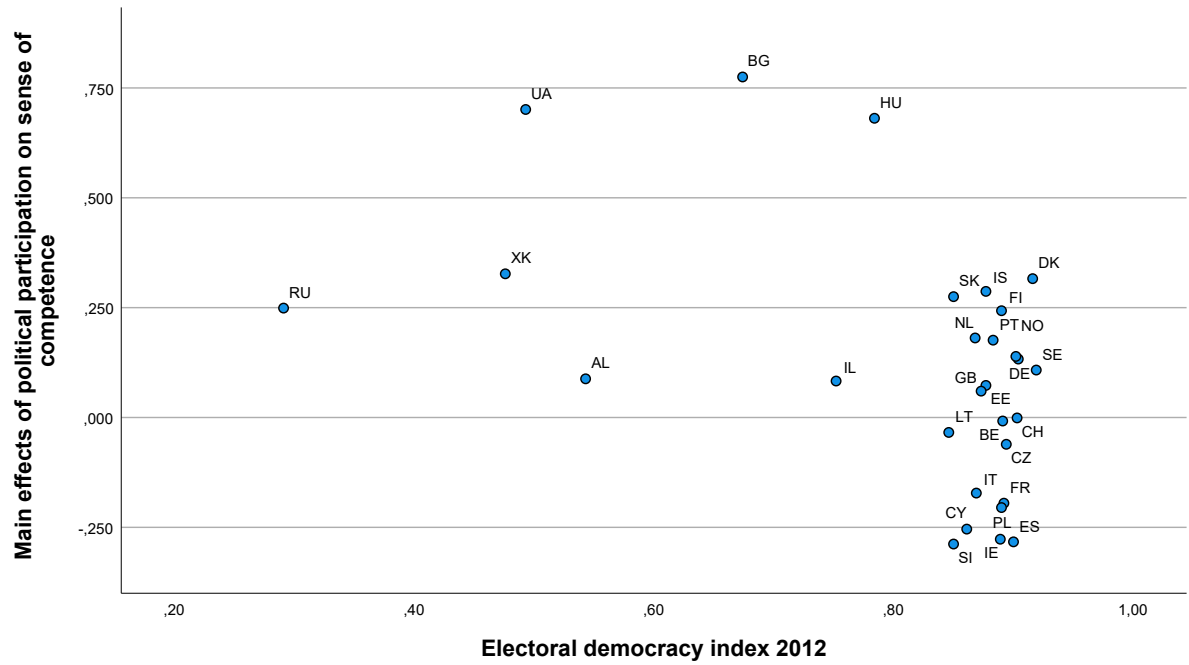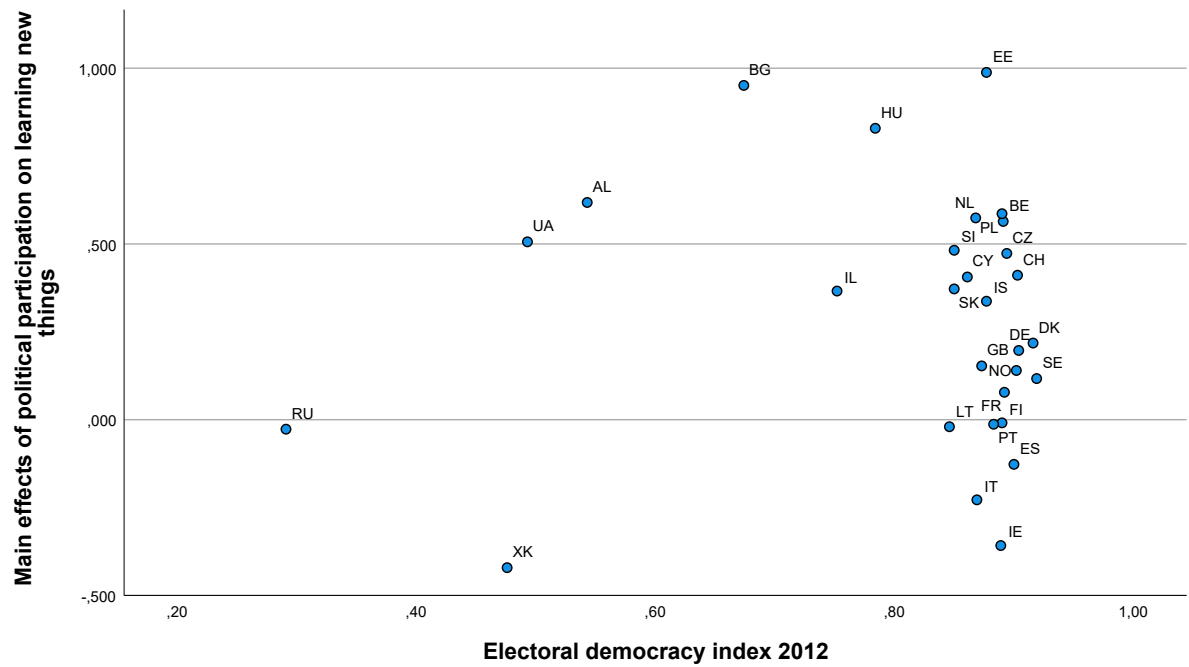

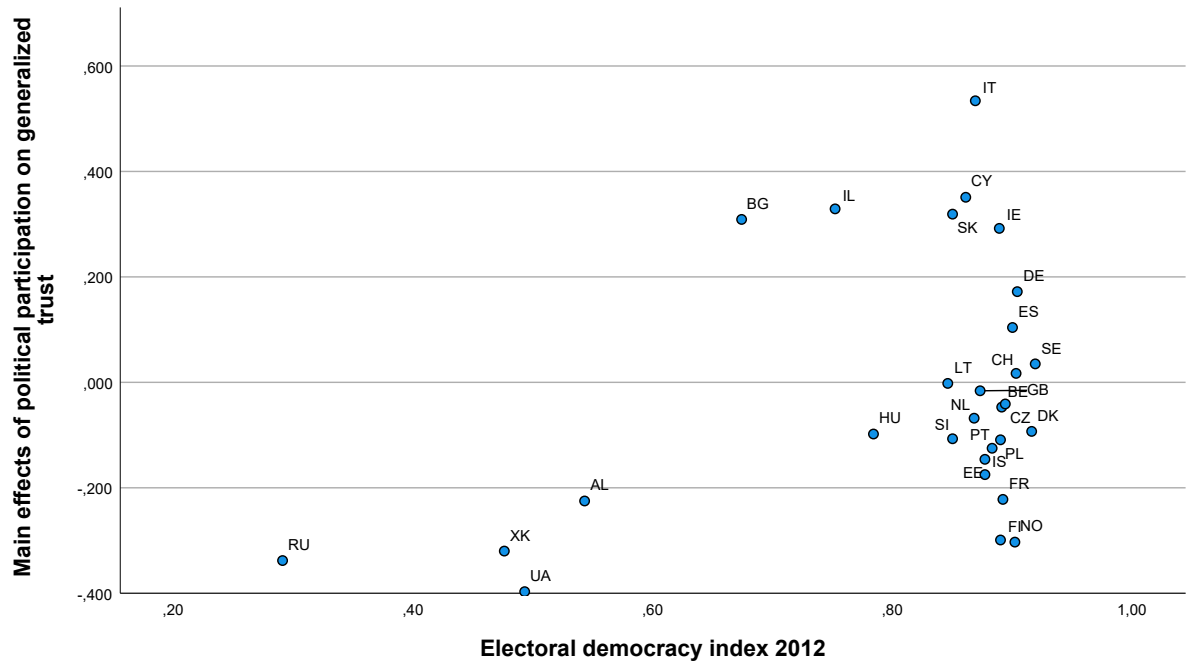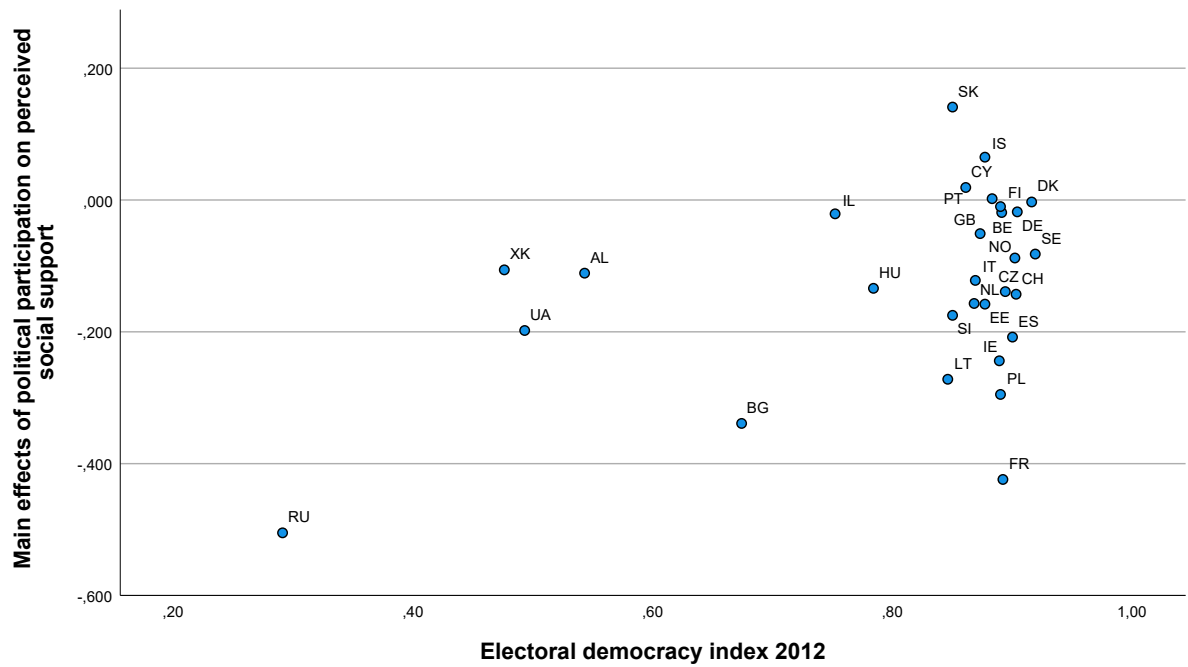

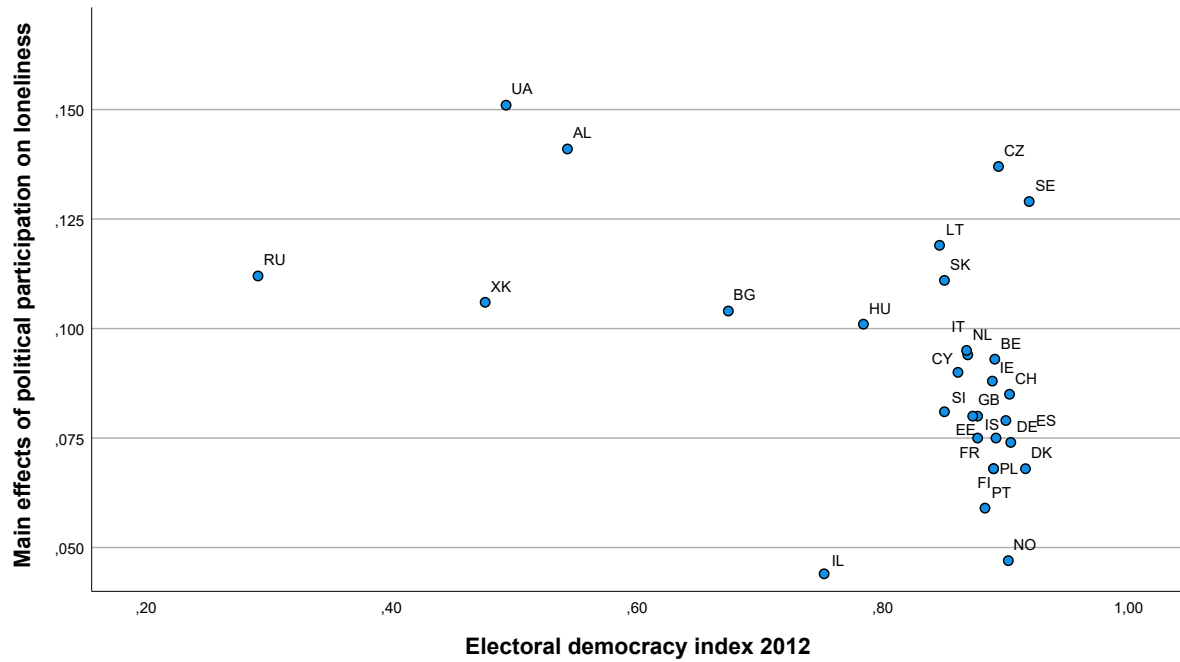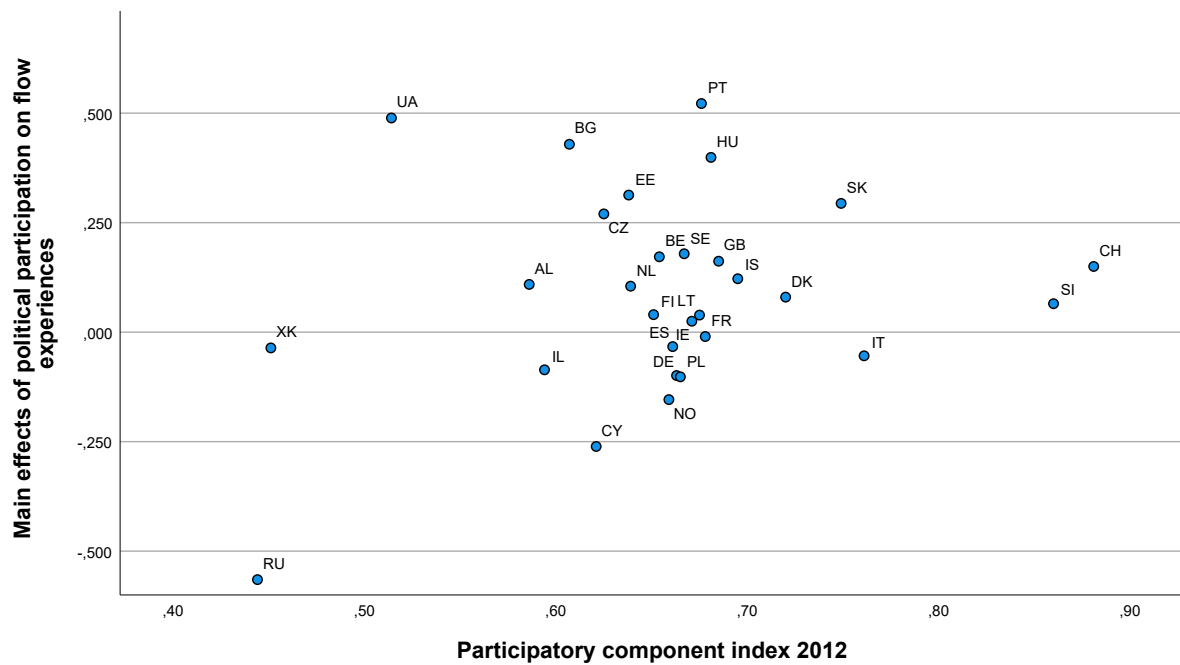

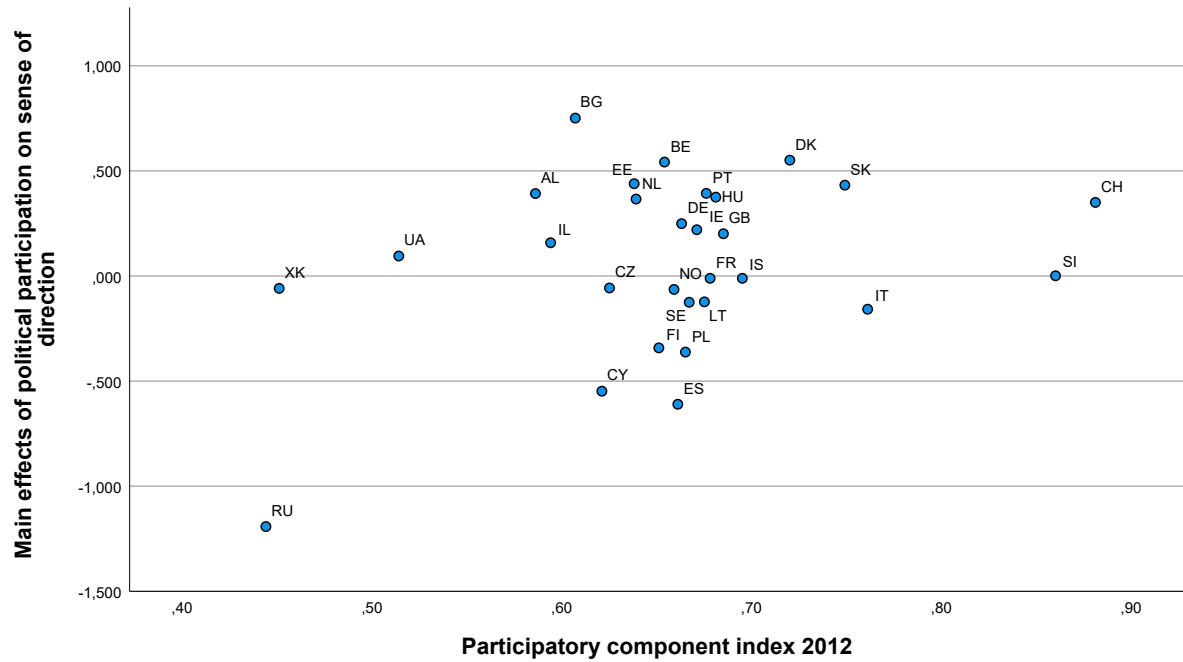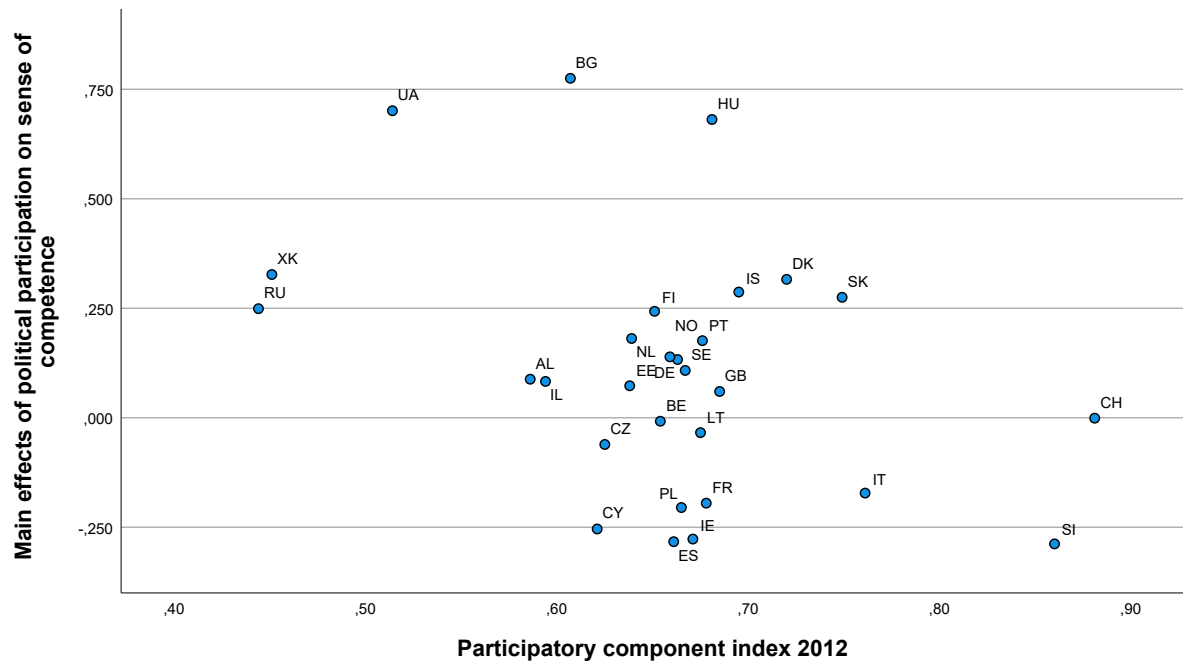

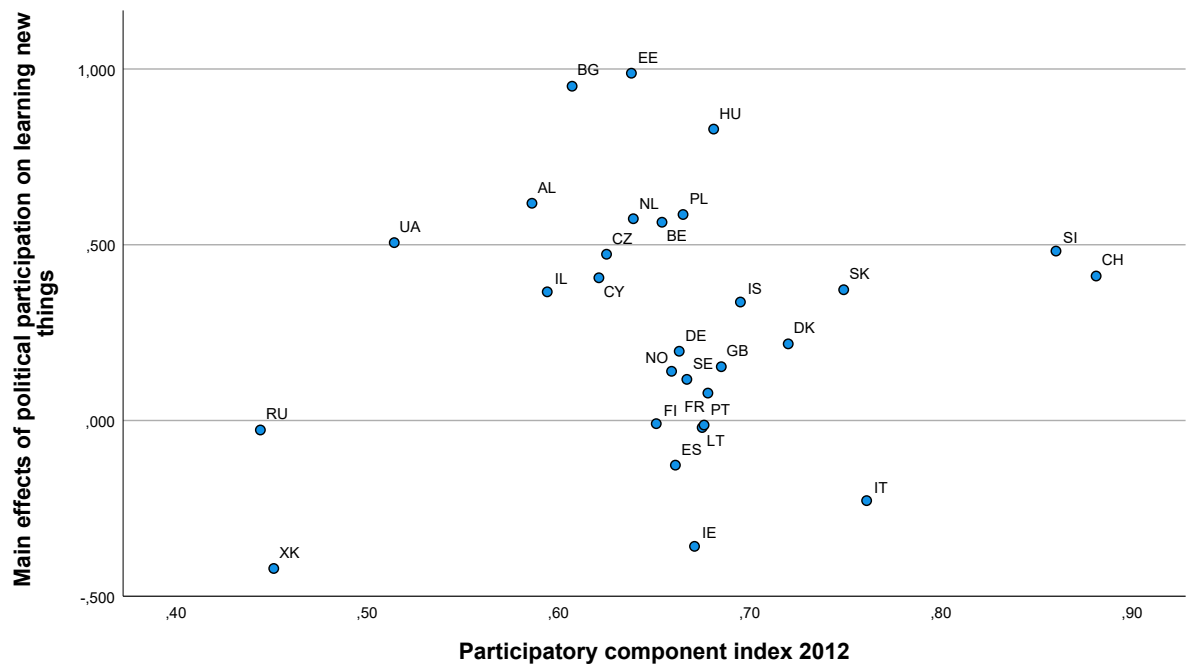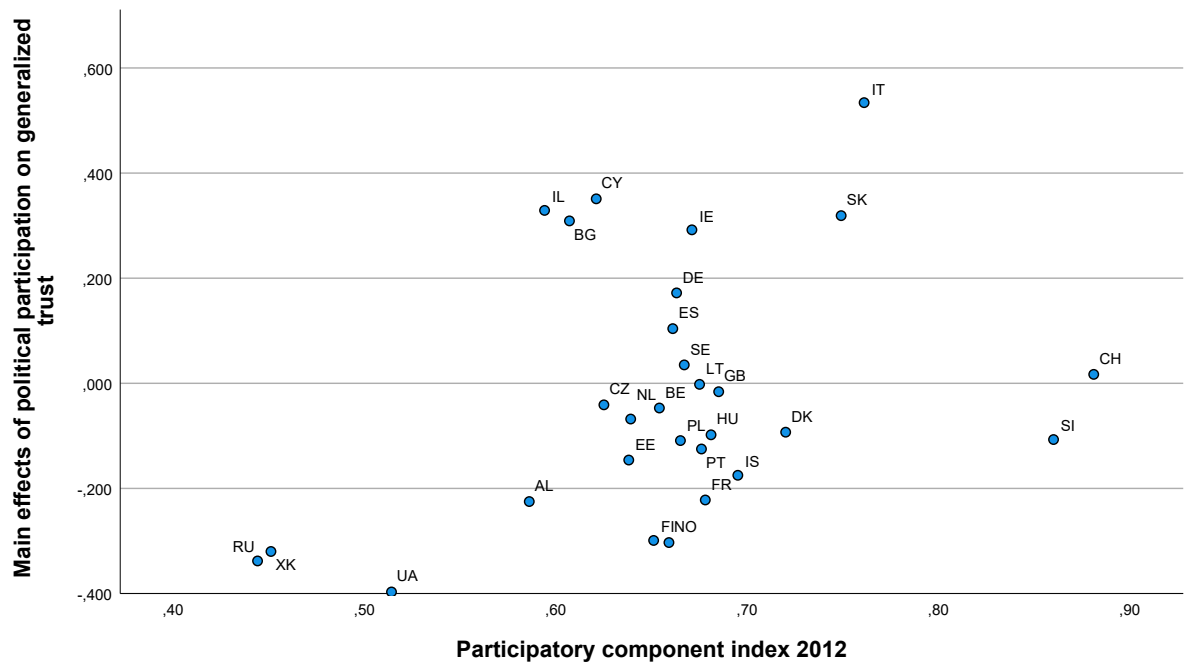

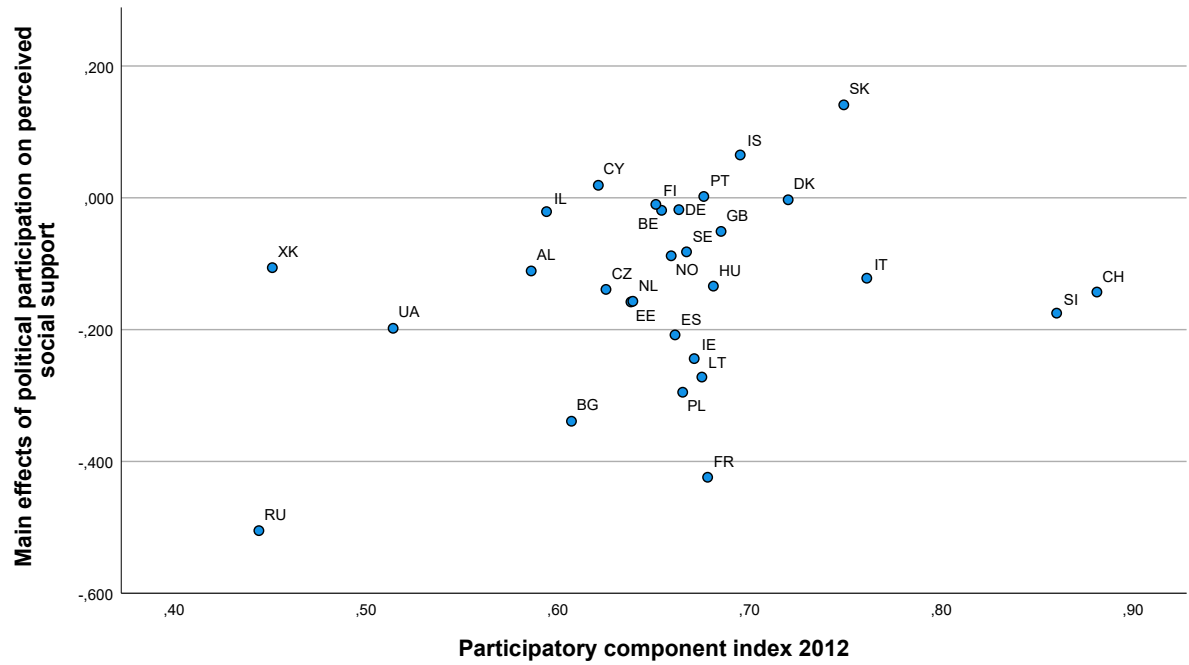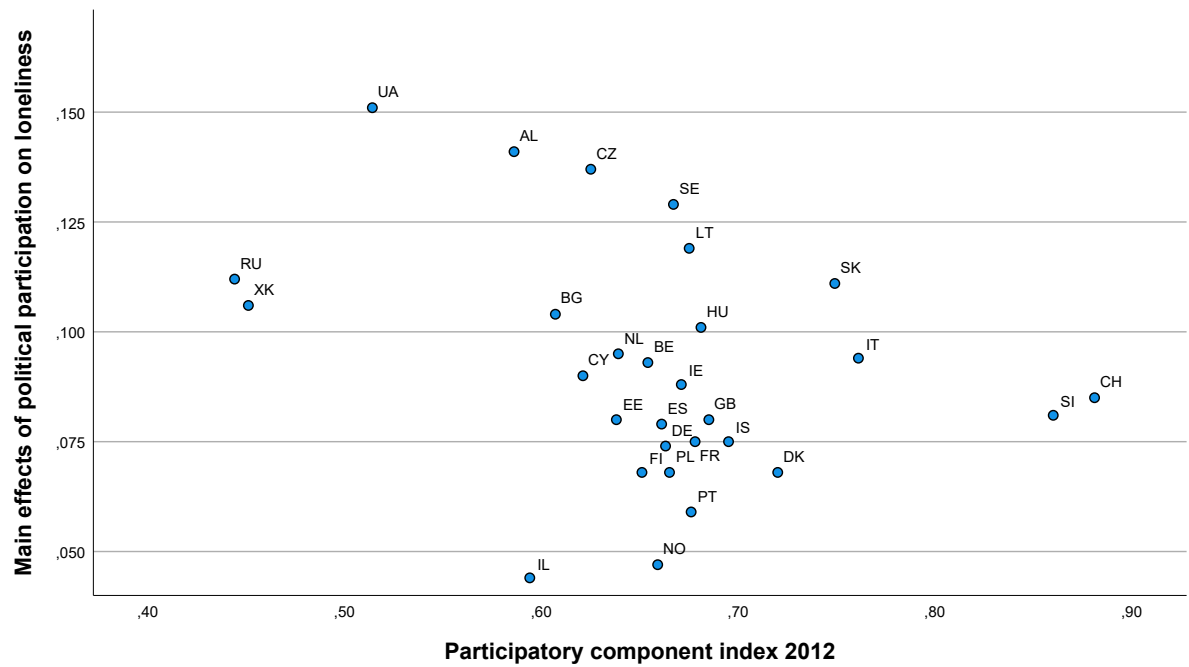

Supplement: S2 Fig — (PDF) [file pone.0281354.s005.pdf]

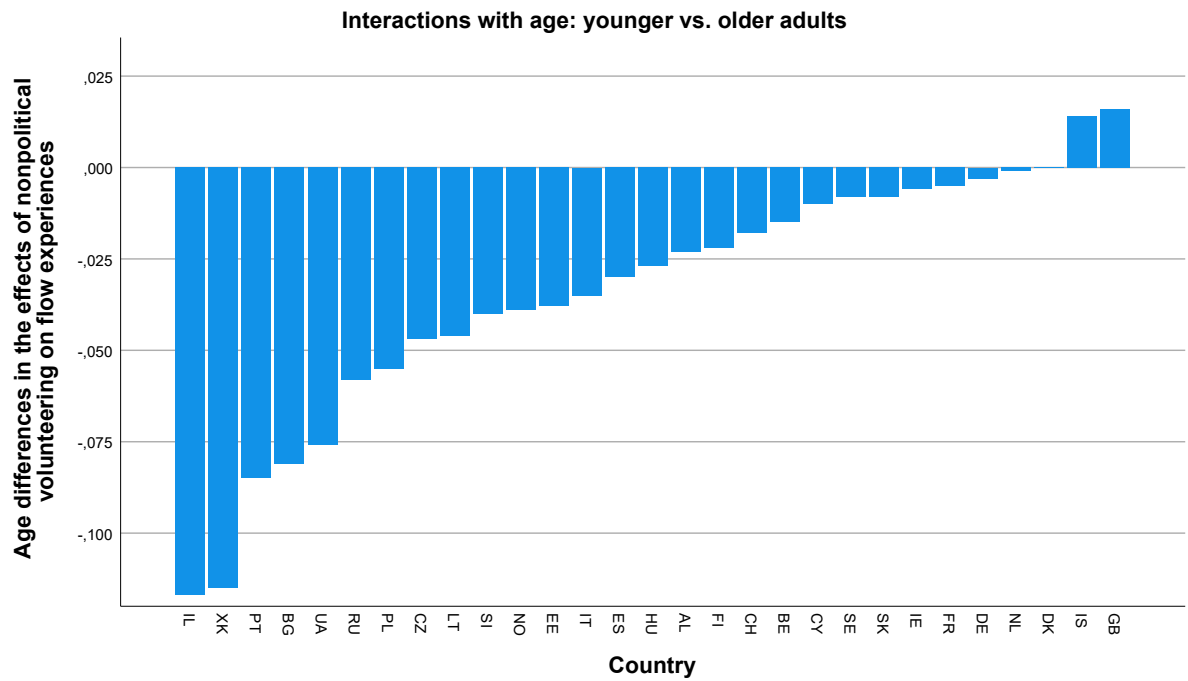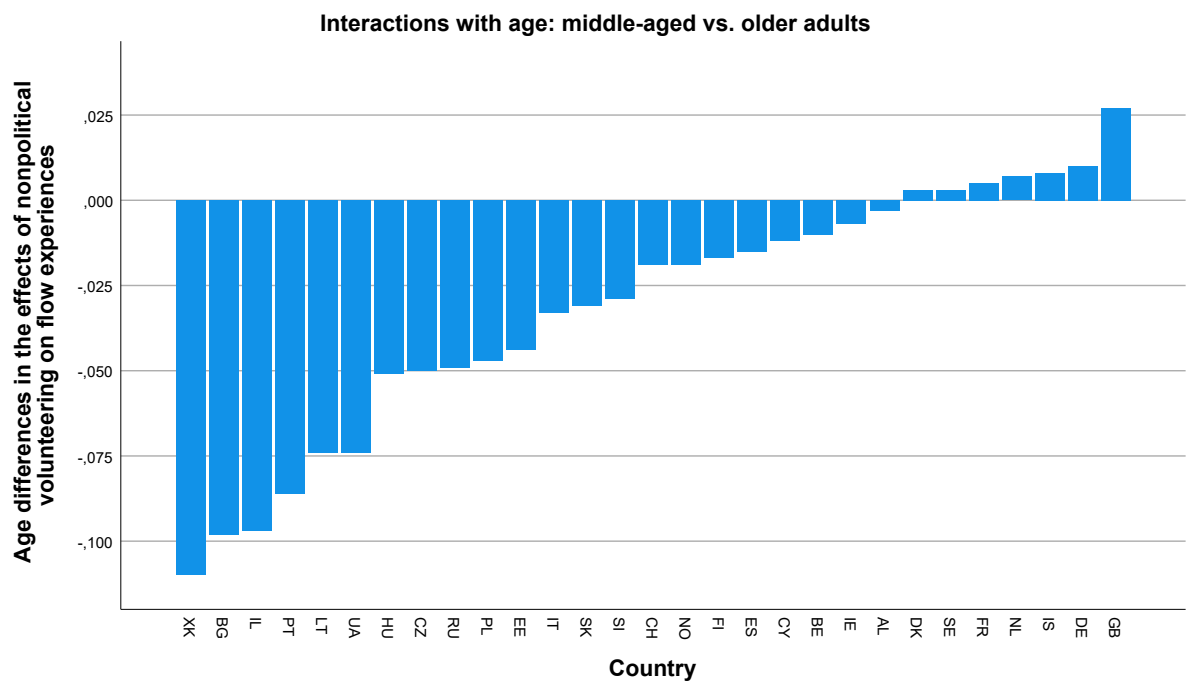

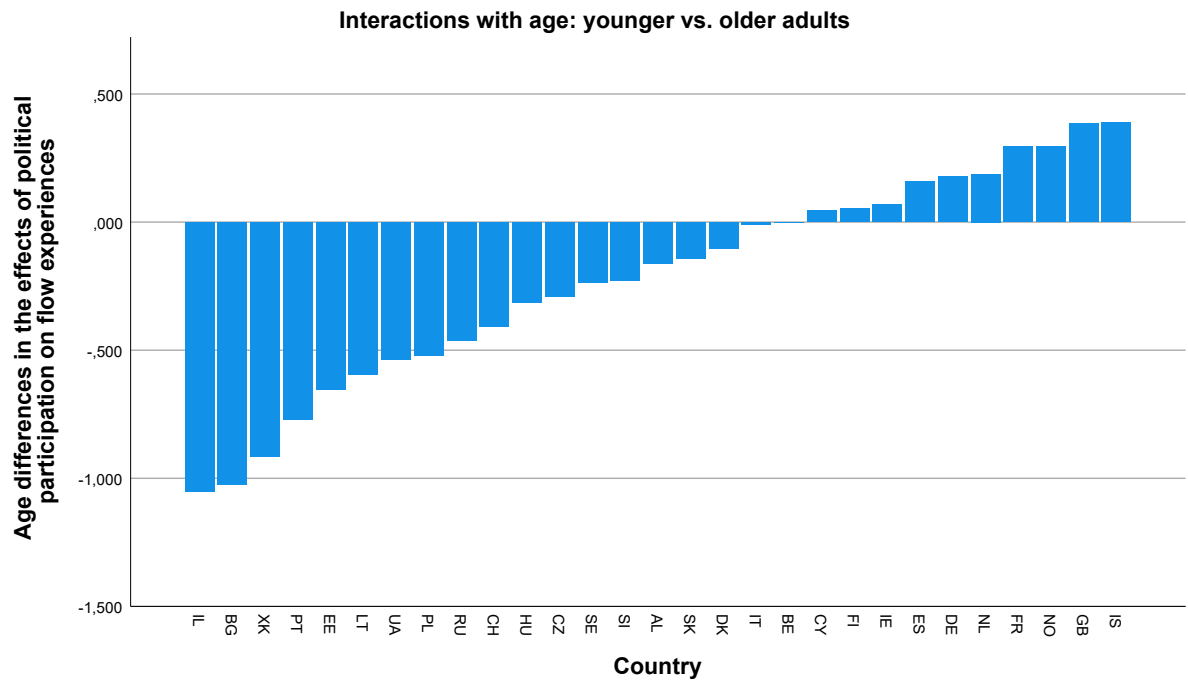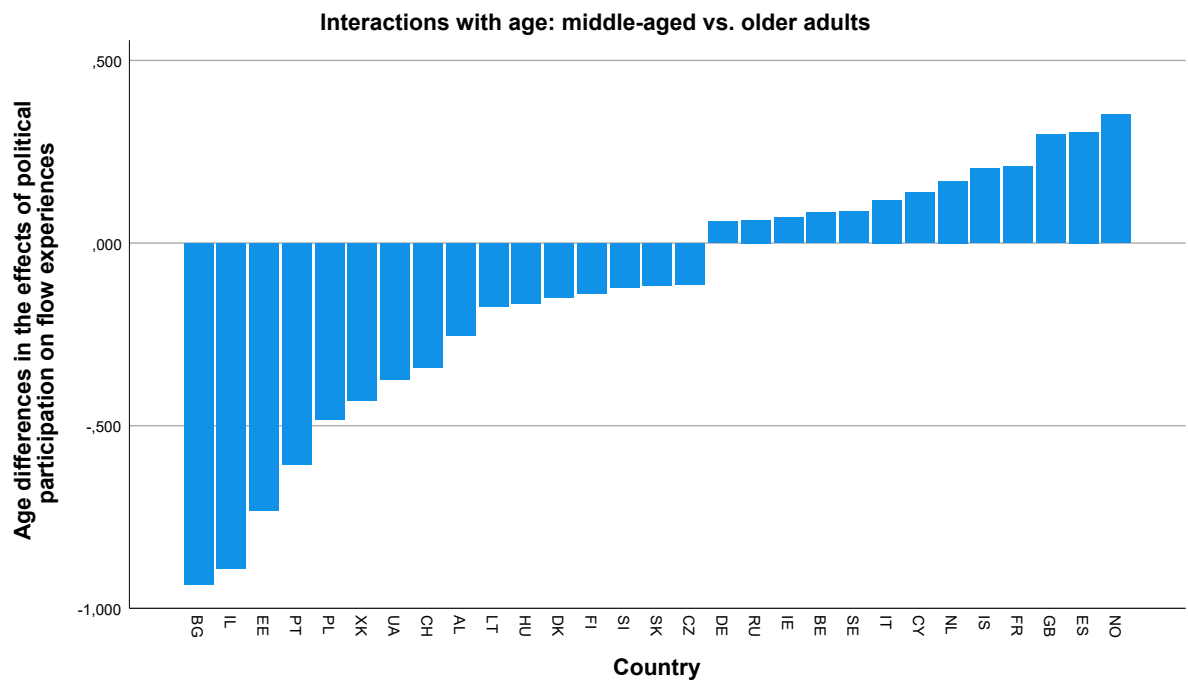

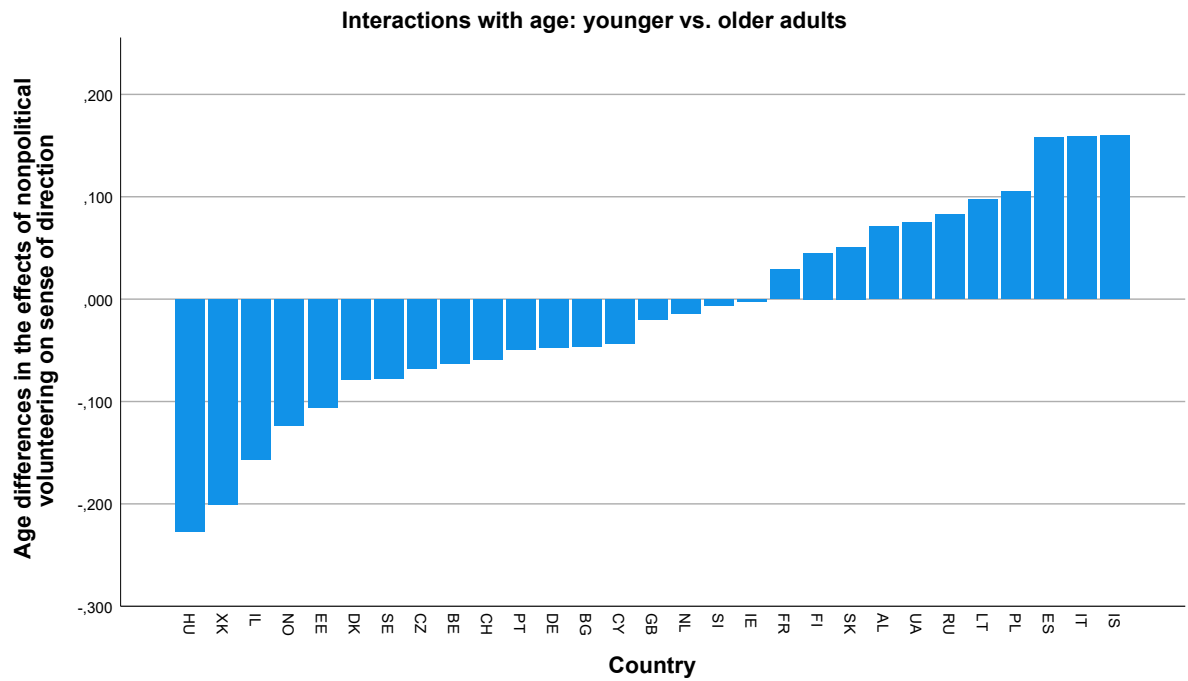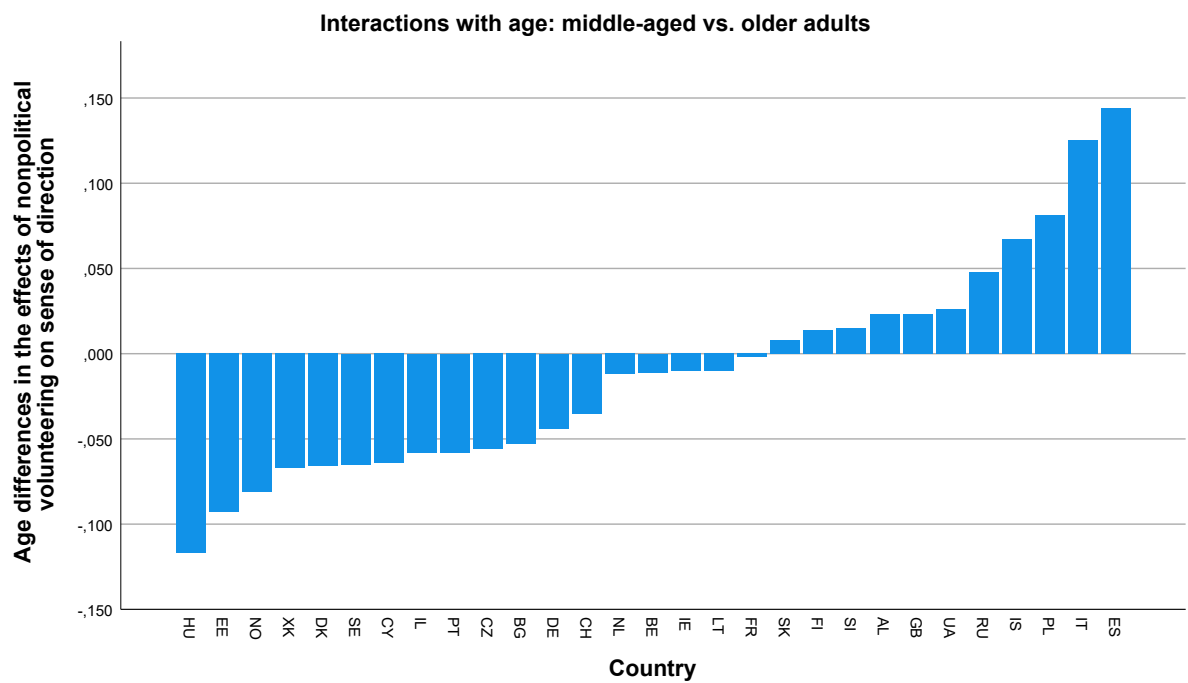

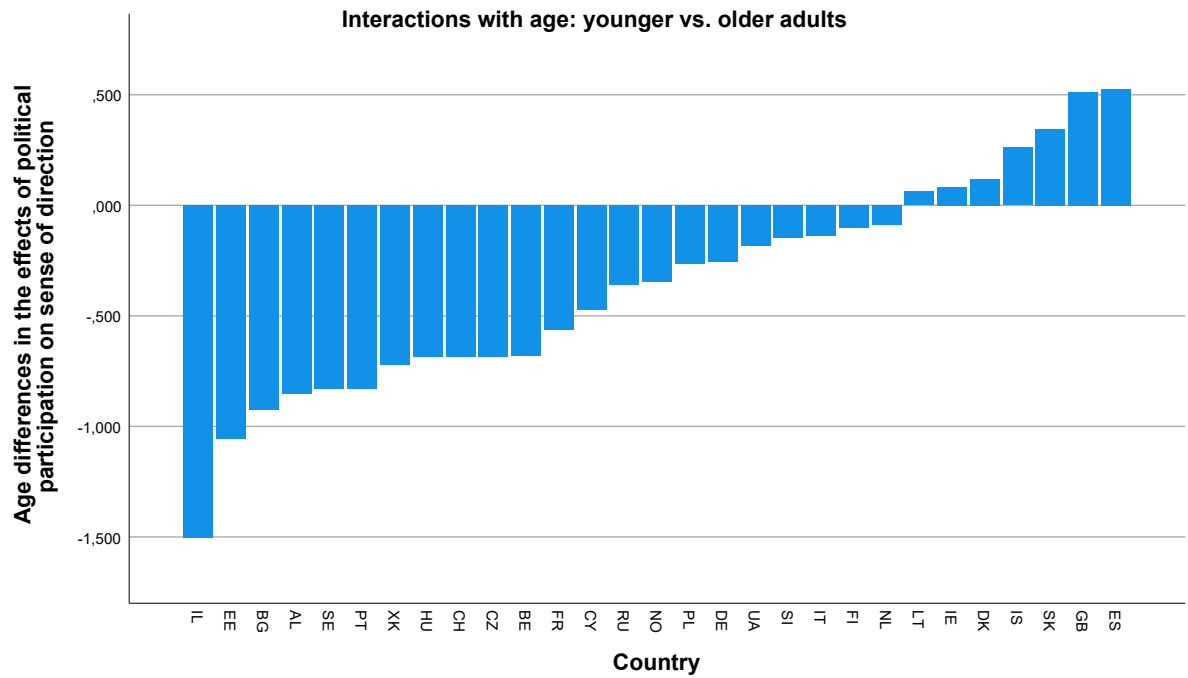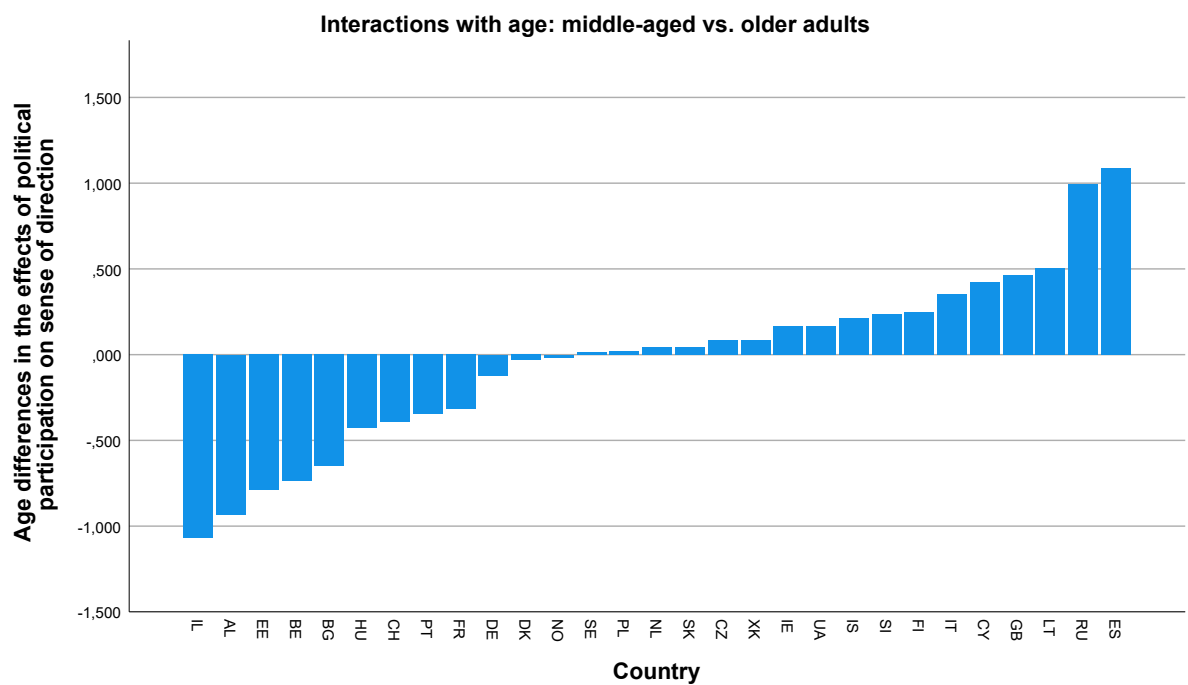

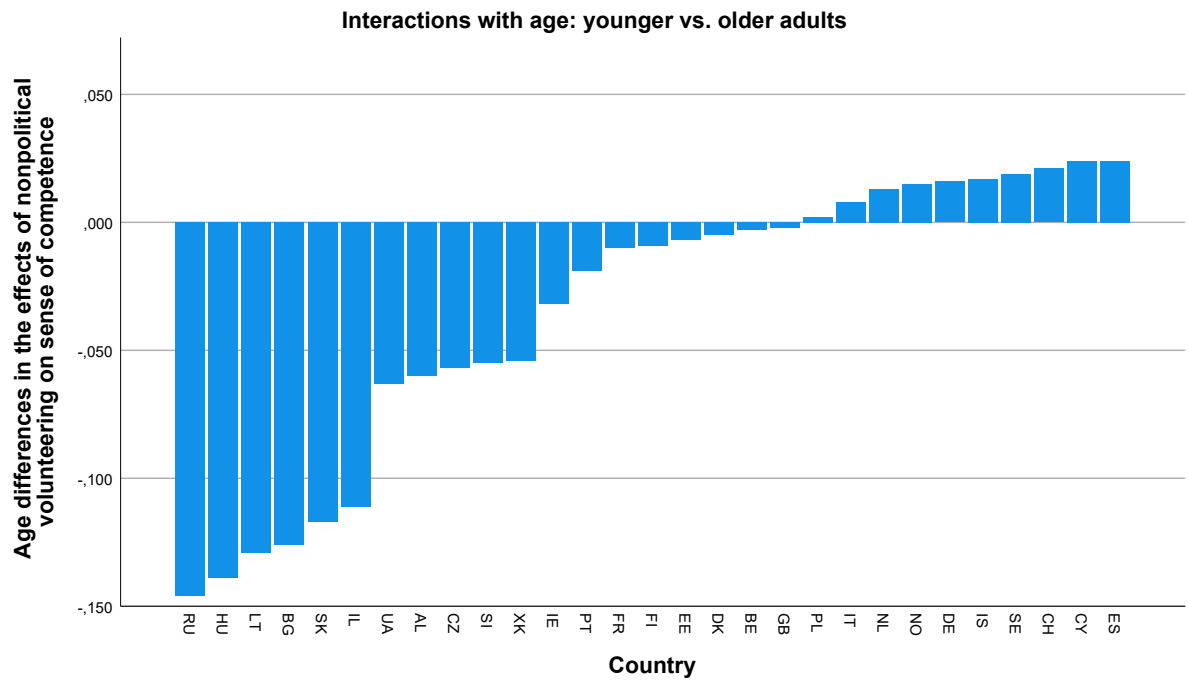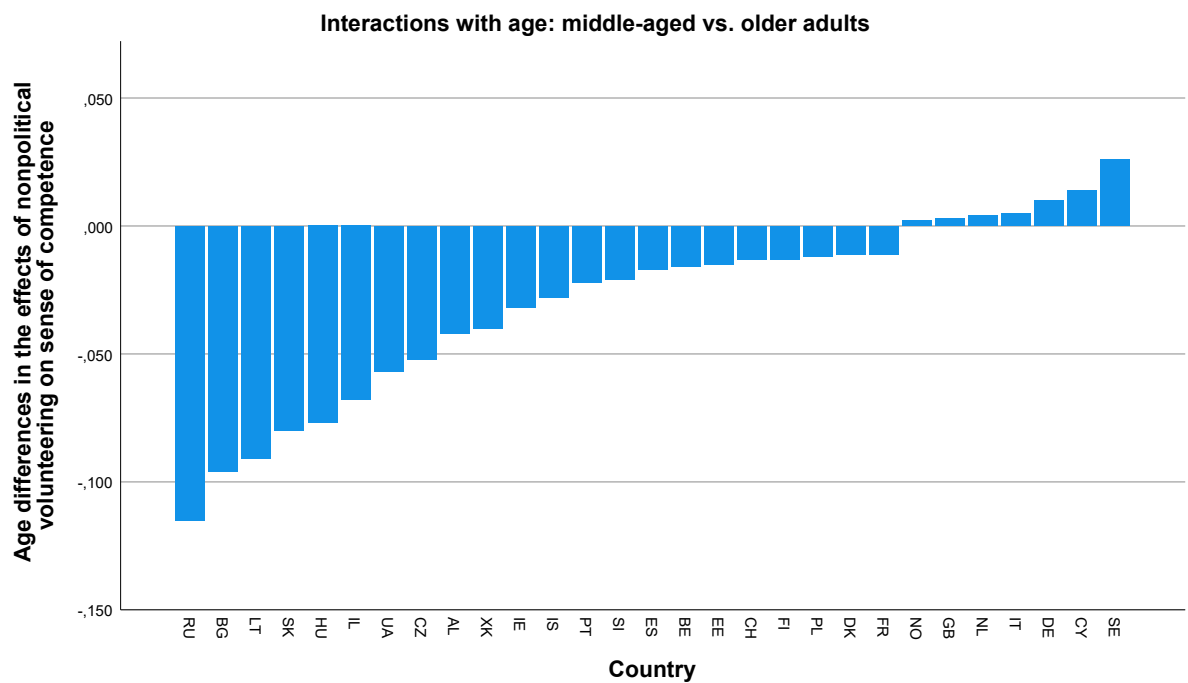

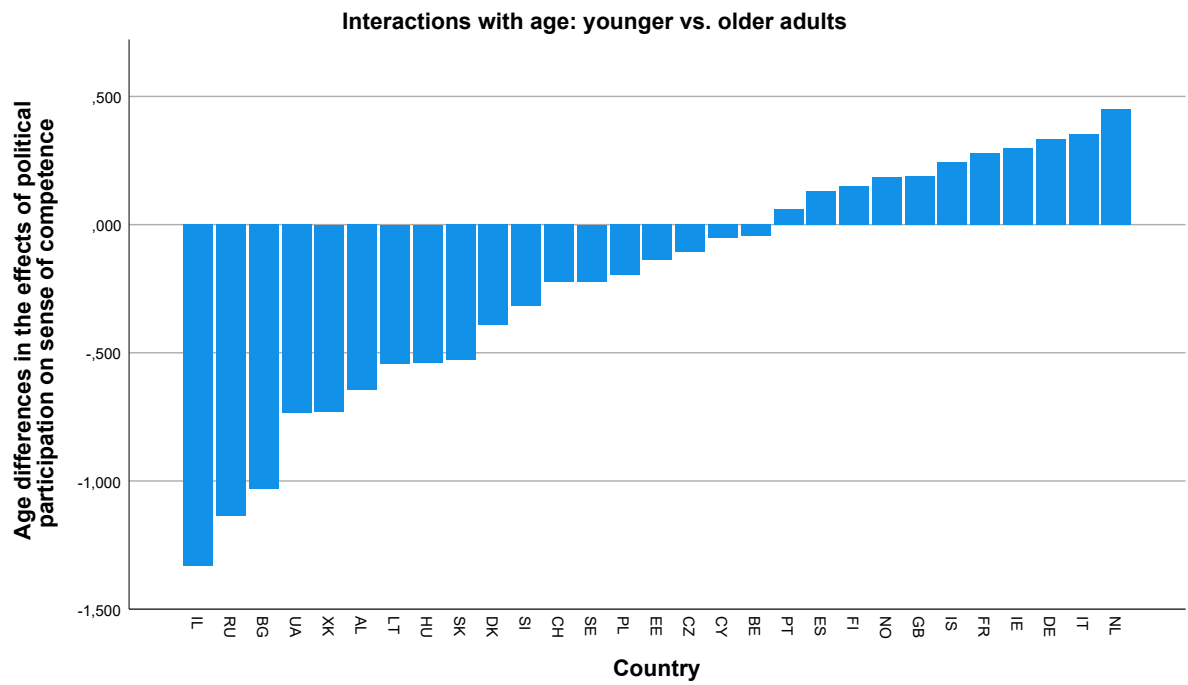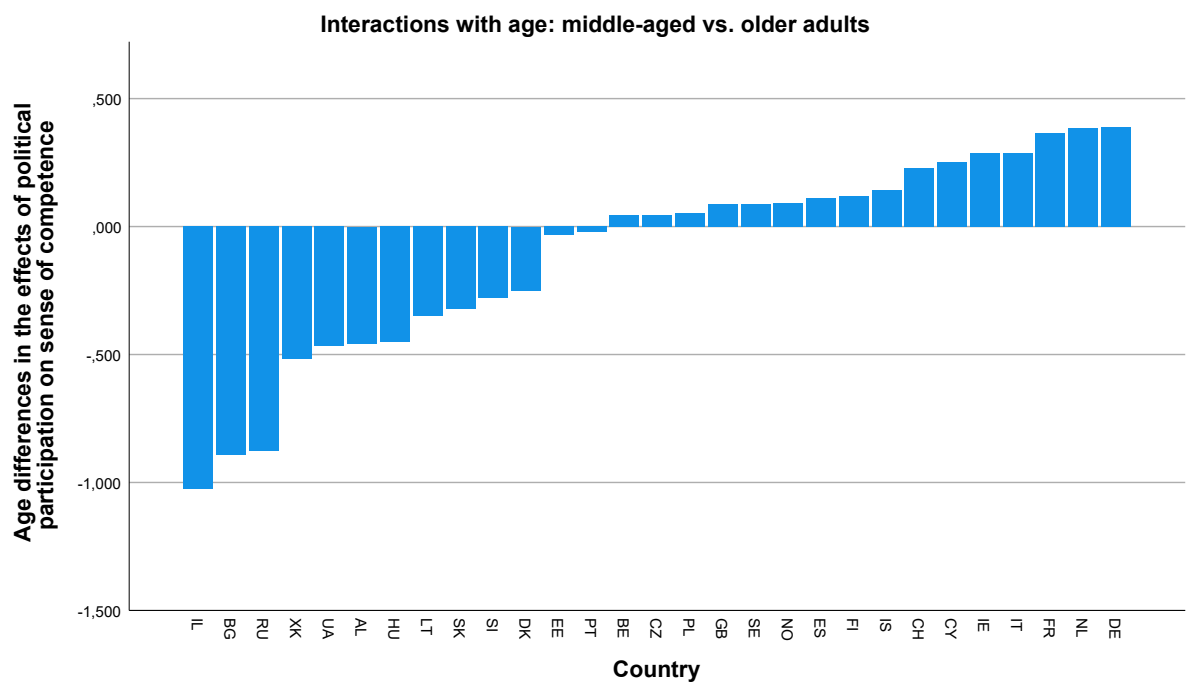

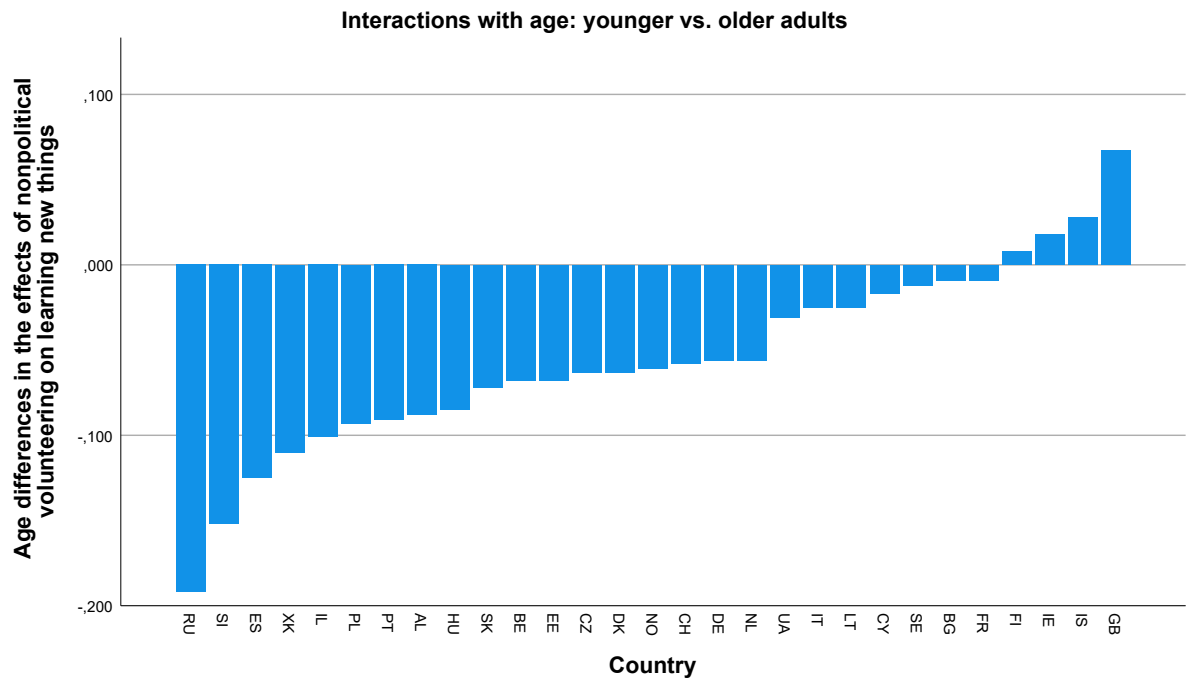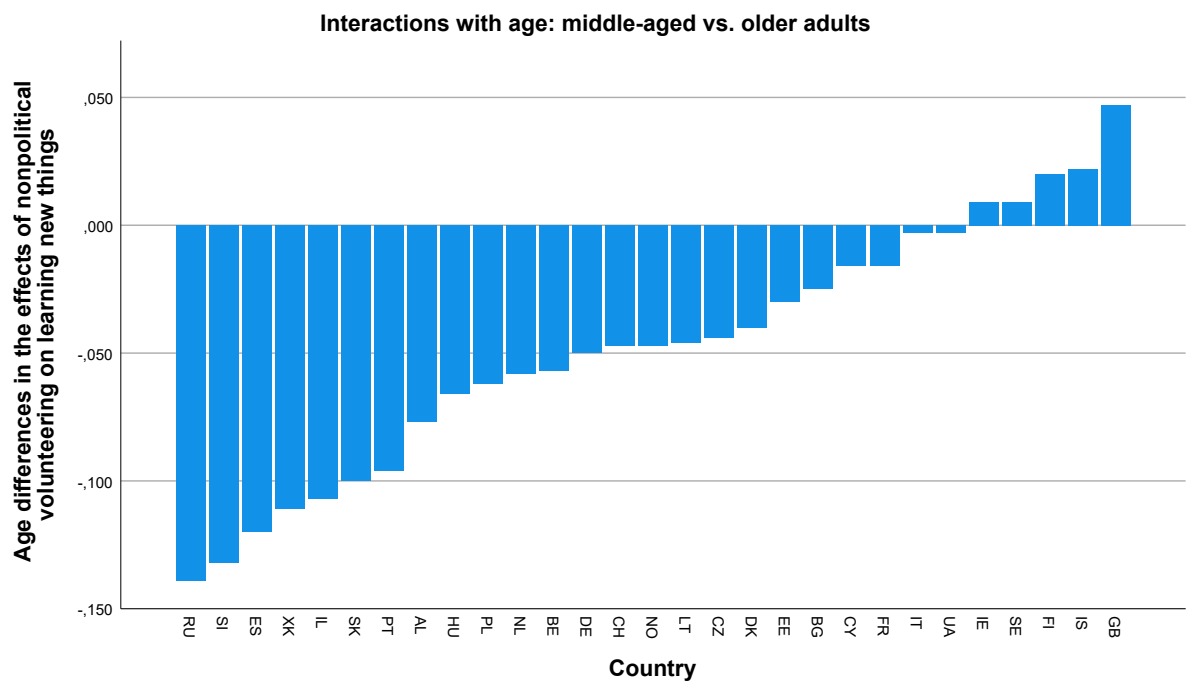

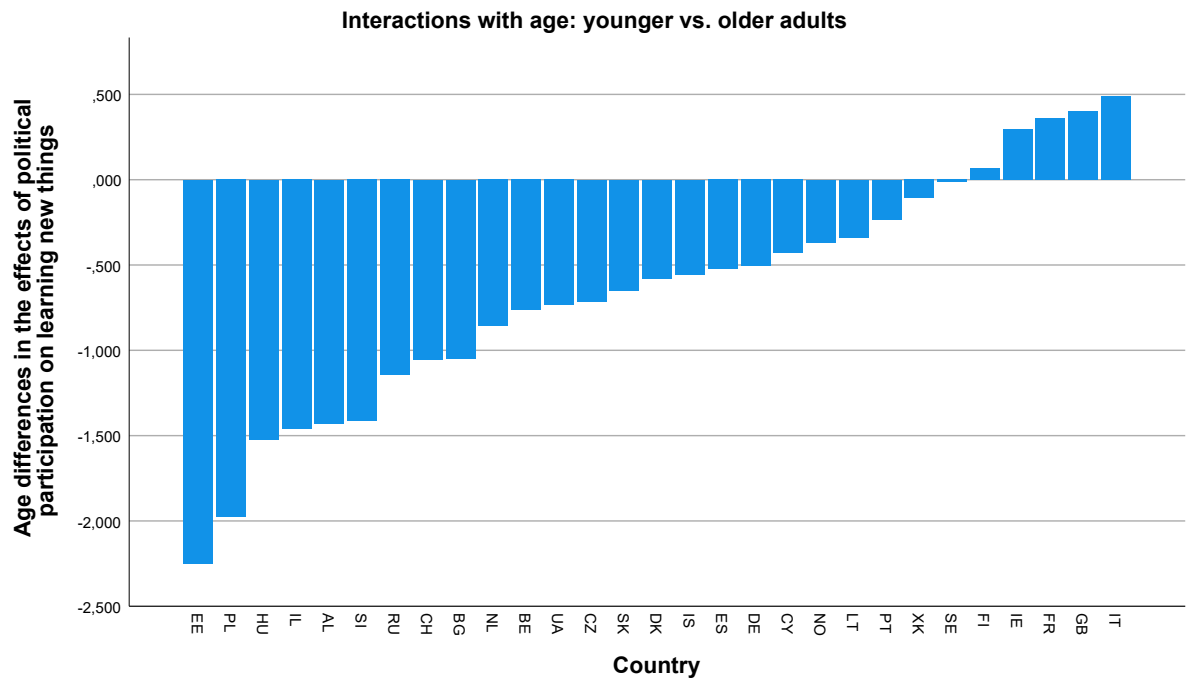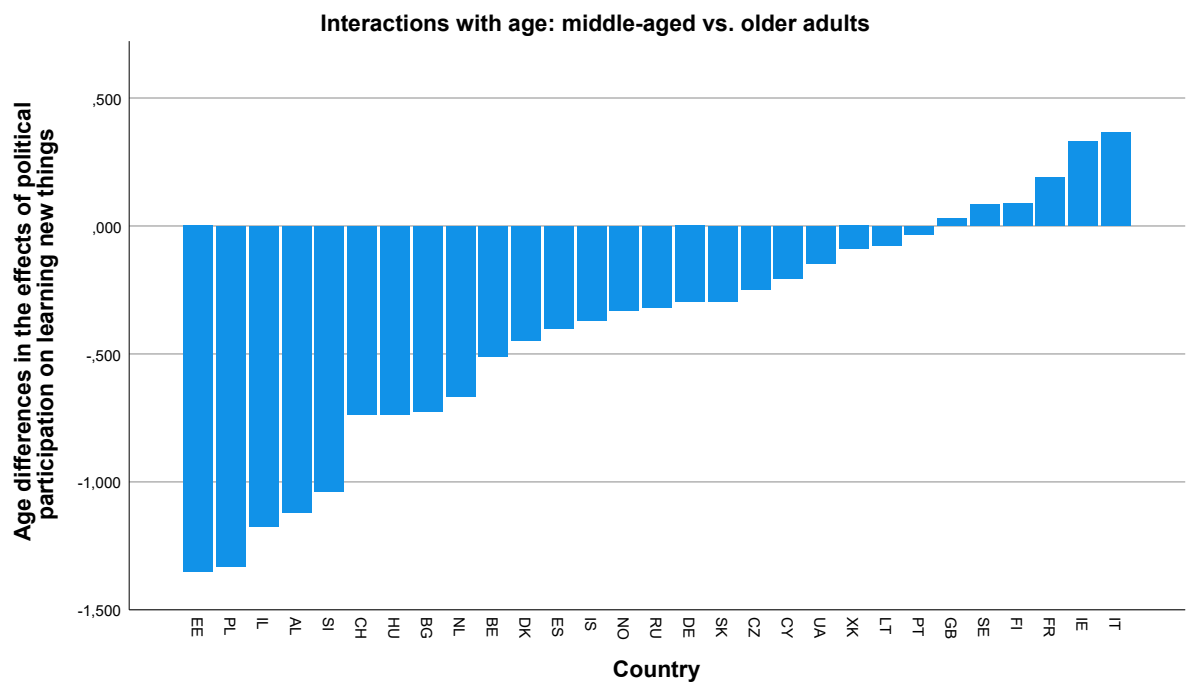

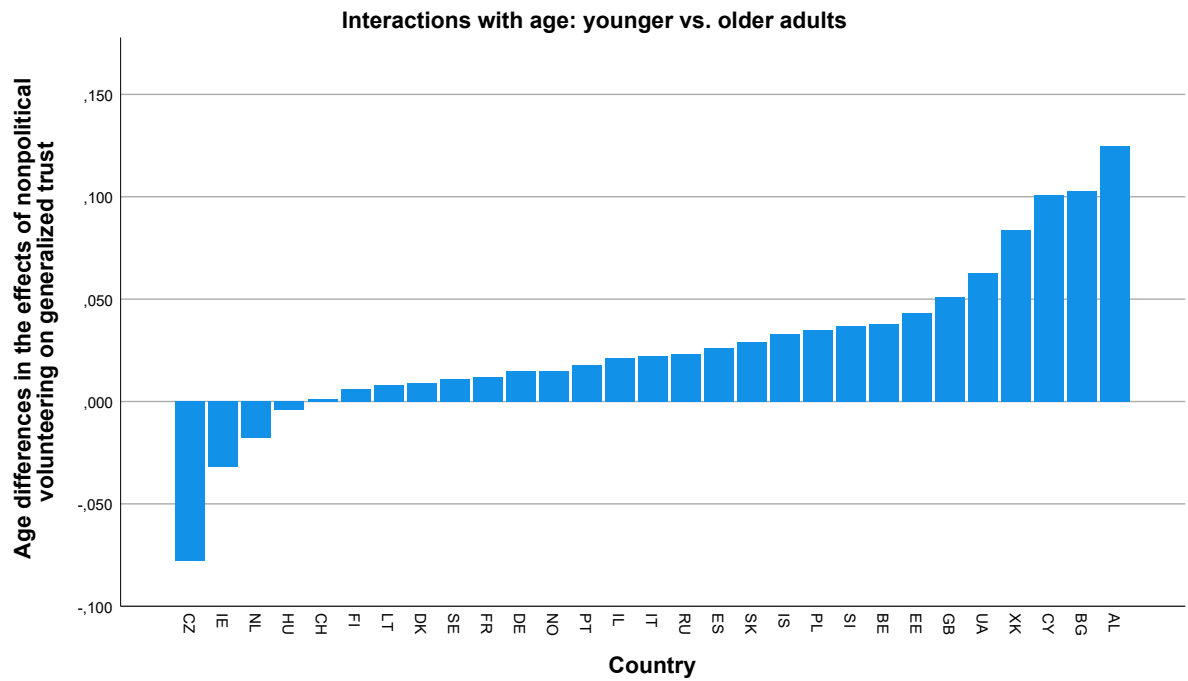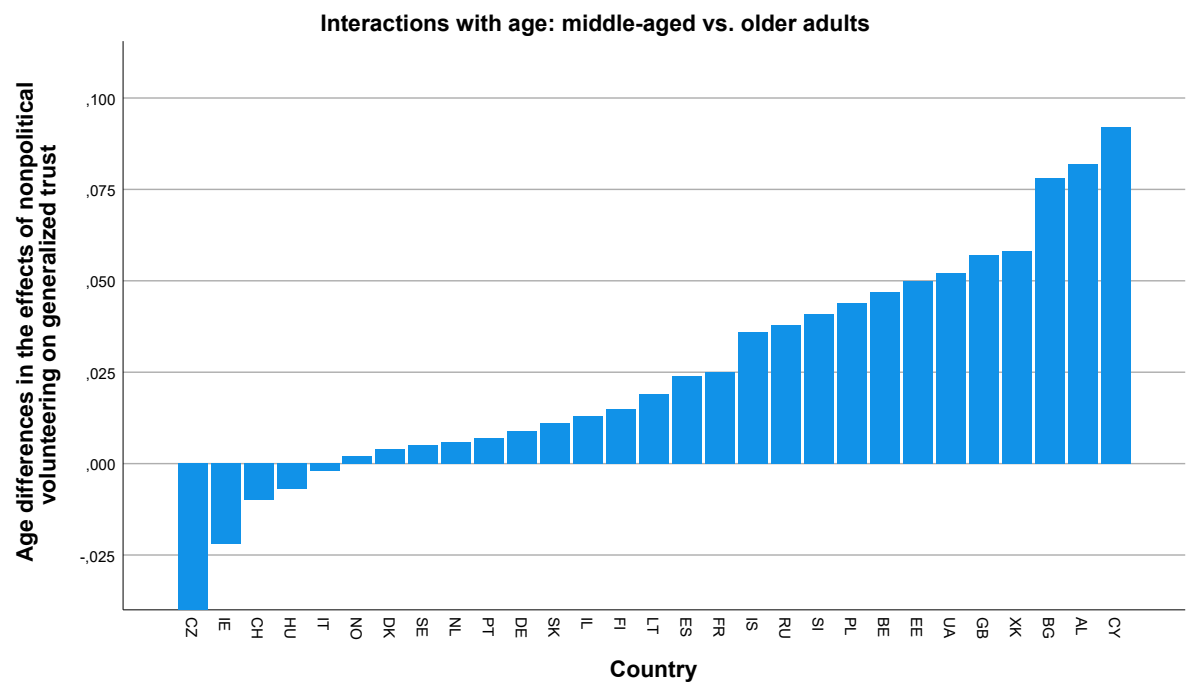

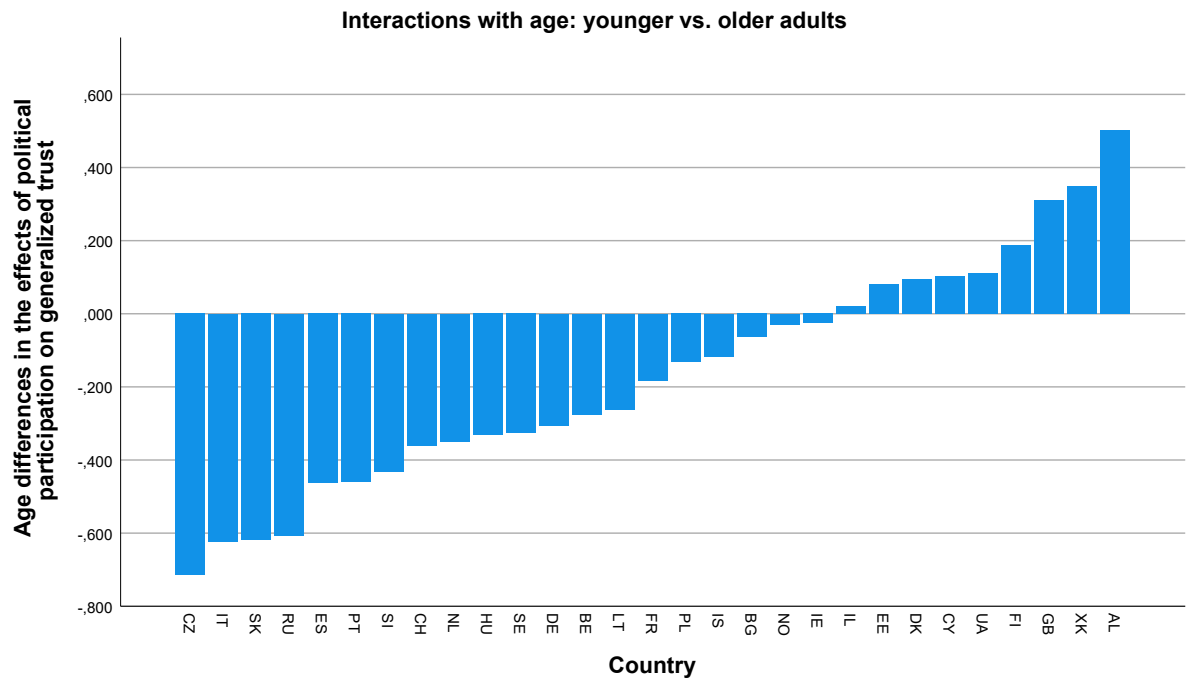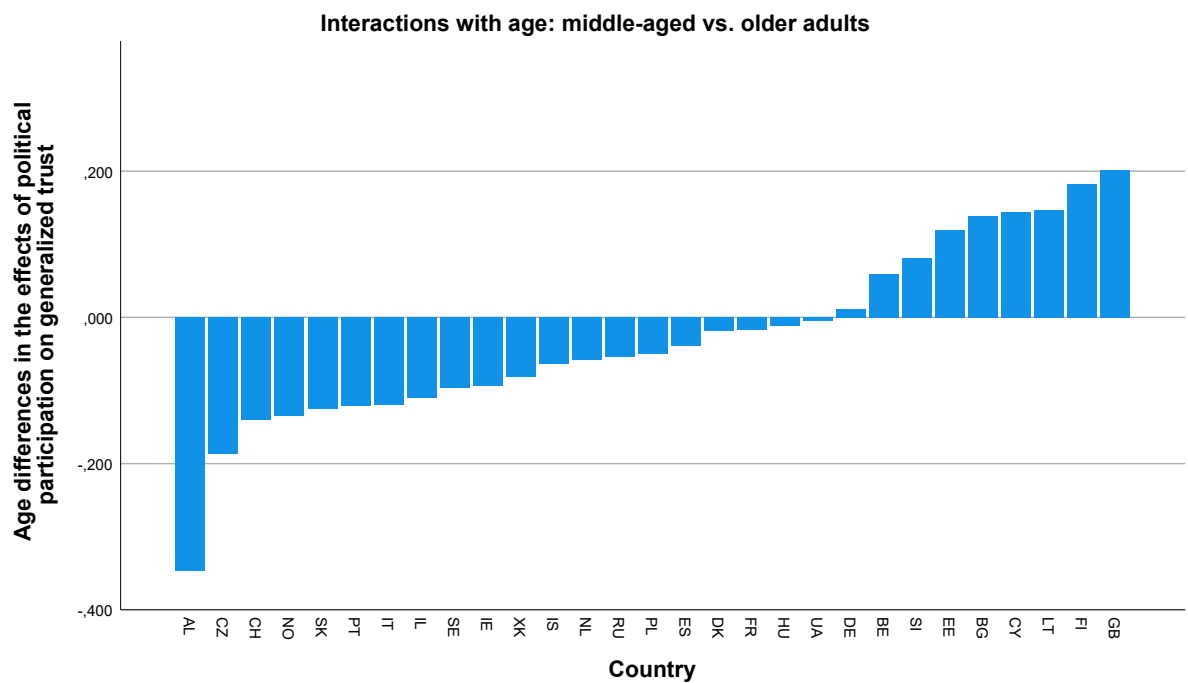

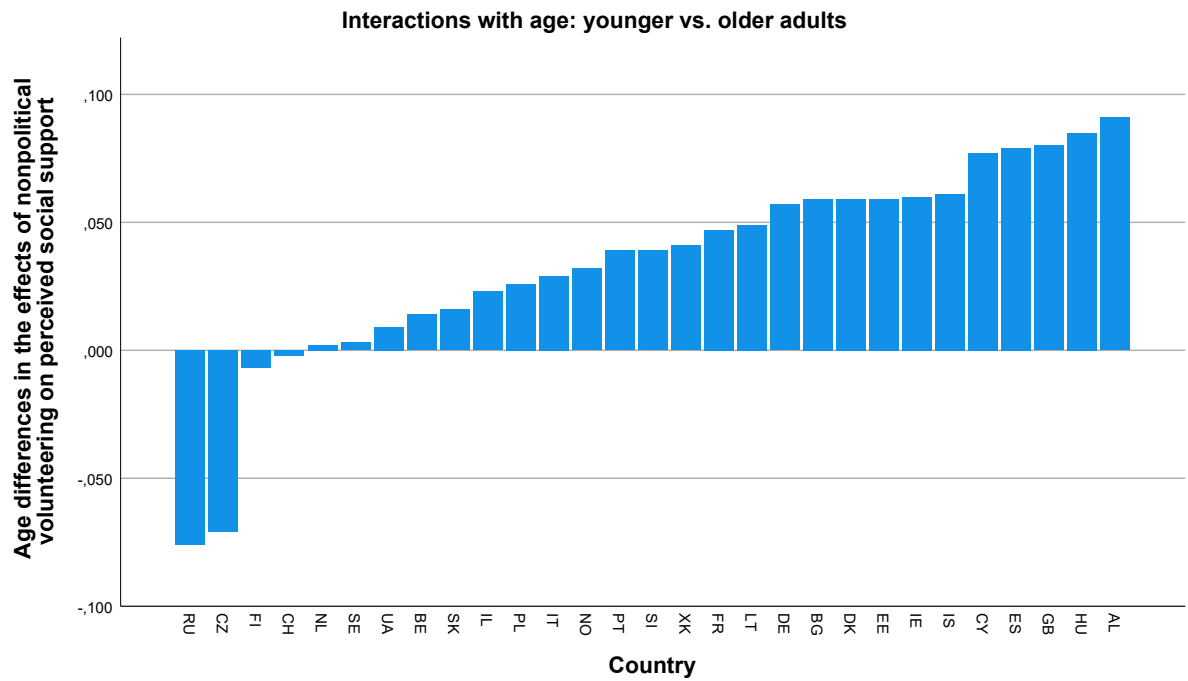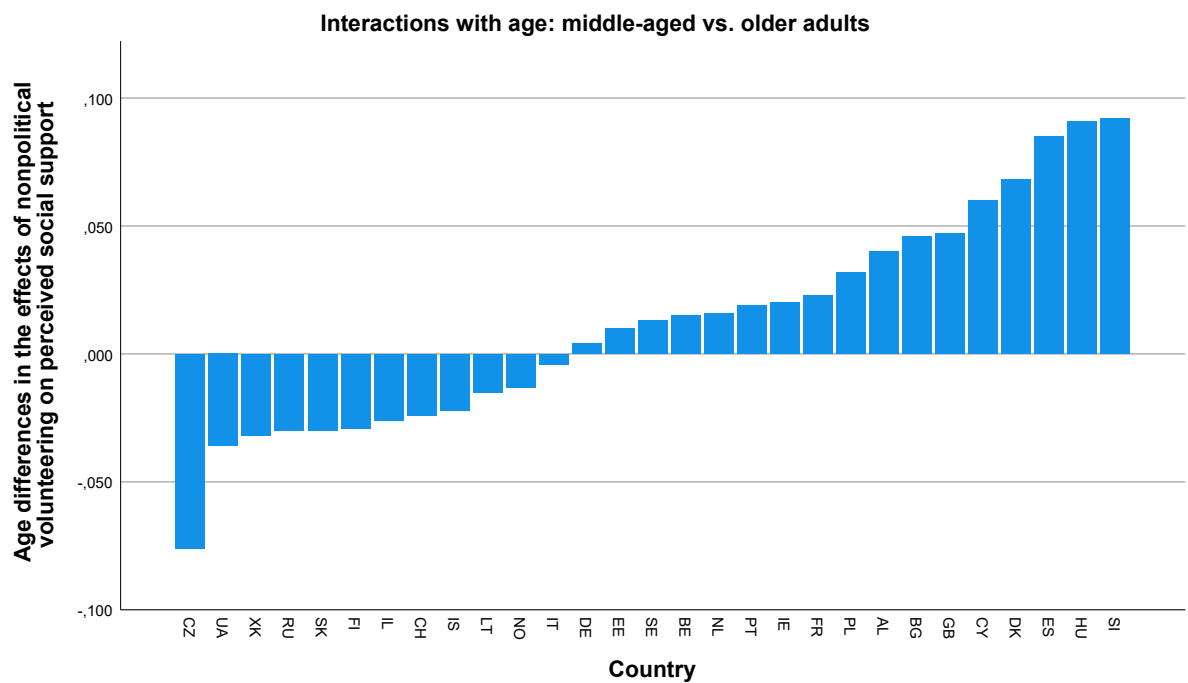

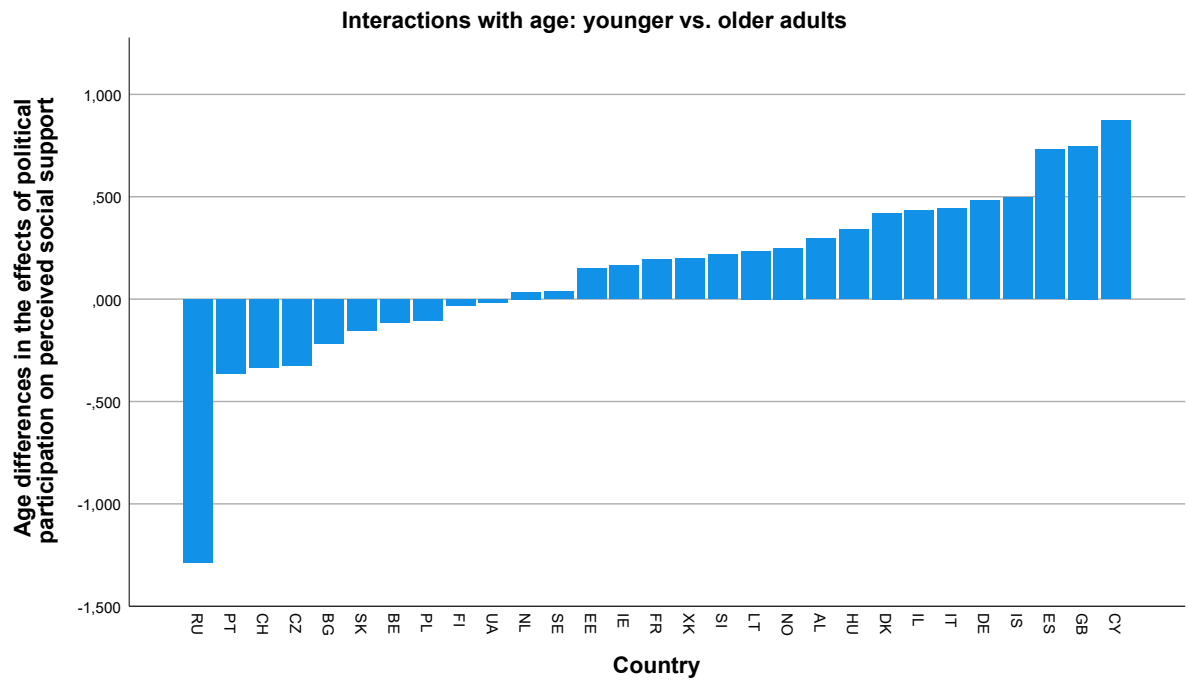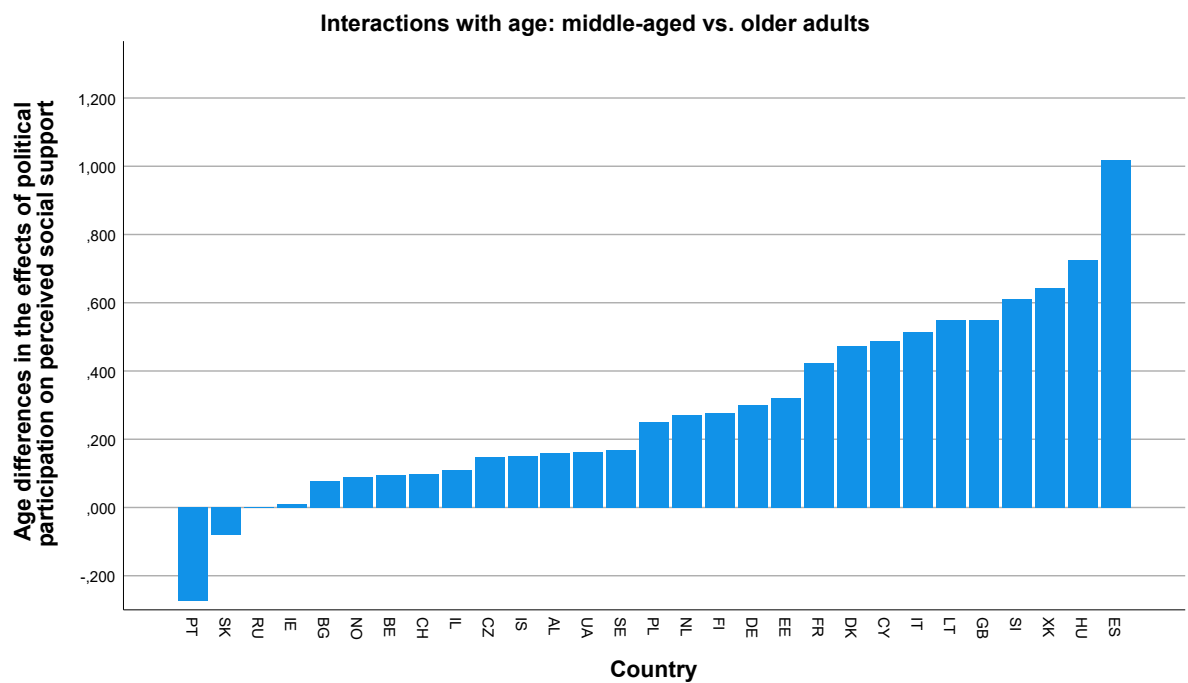

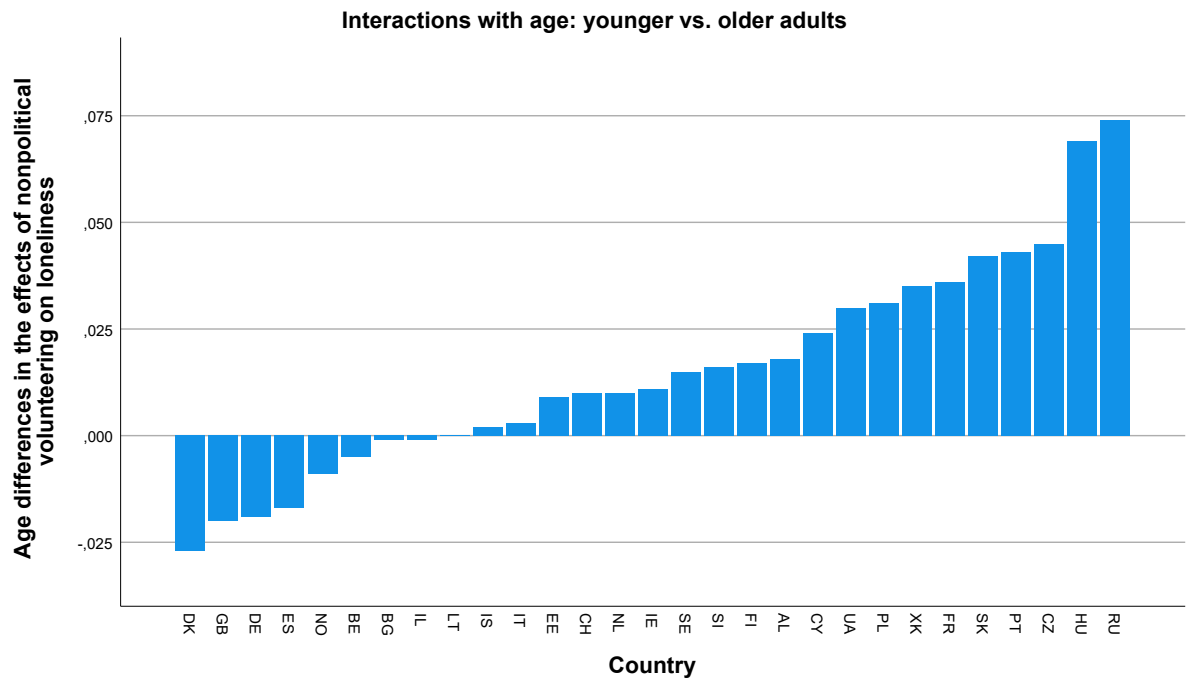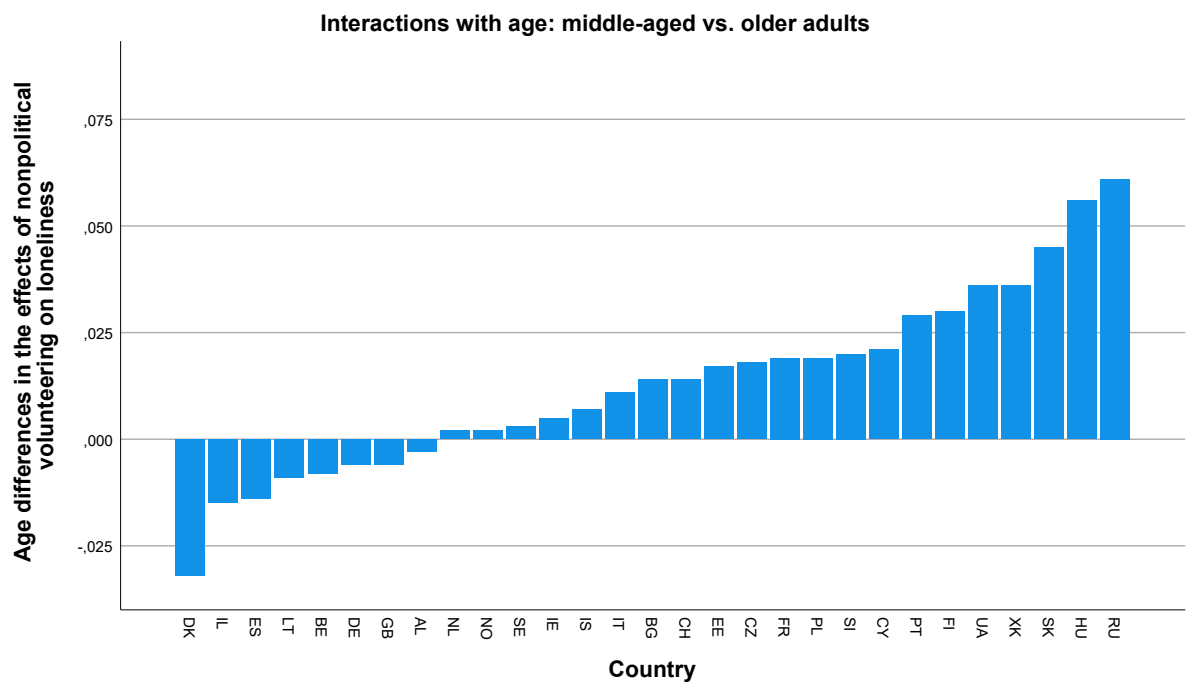

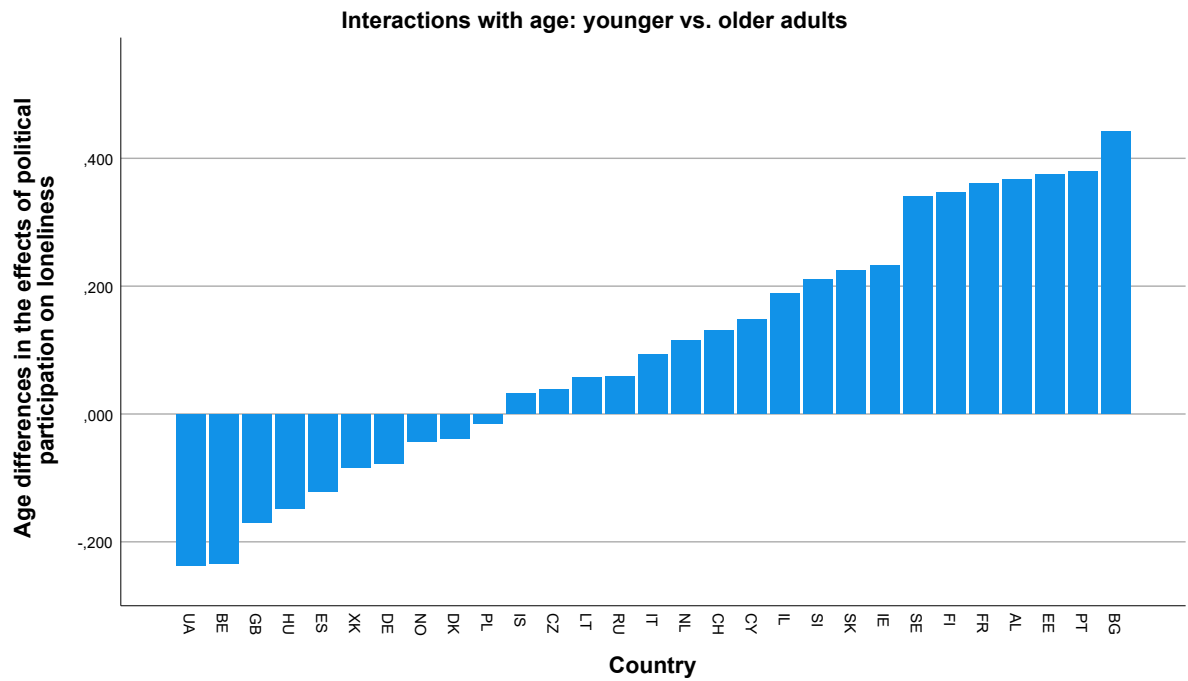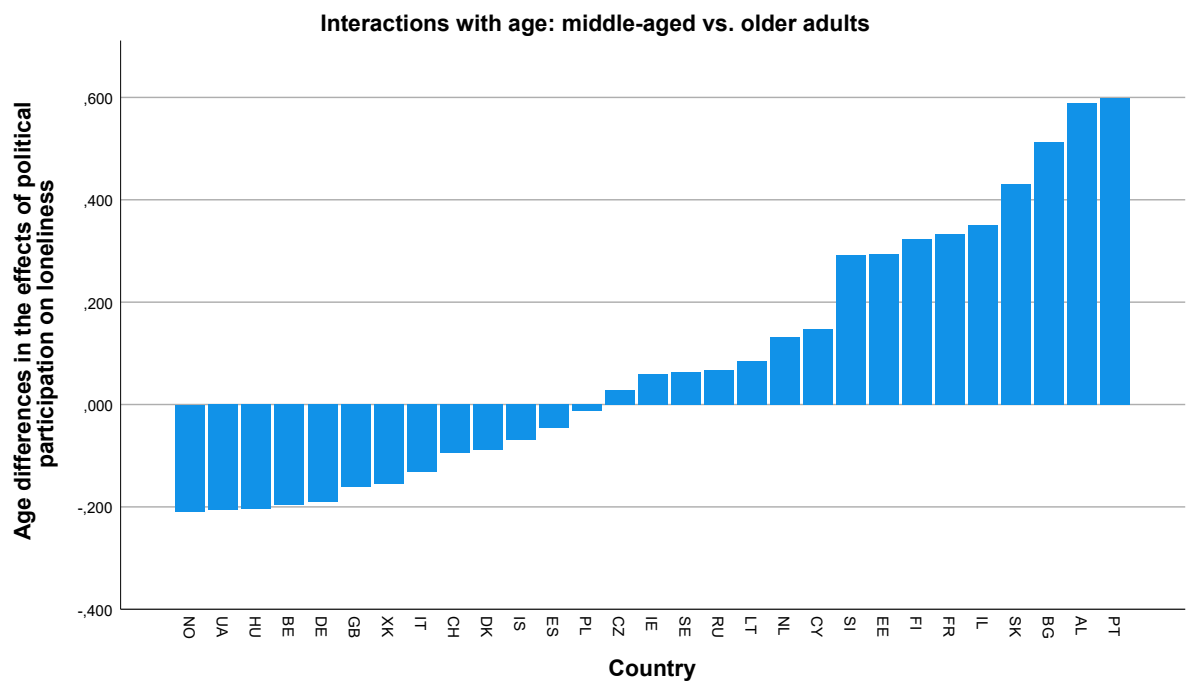

Supplement: S3 Fig — (PDF) [file pone.0281354.s006.pdf]

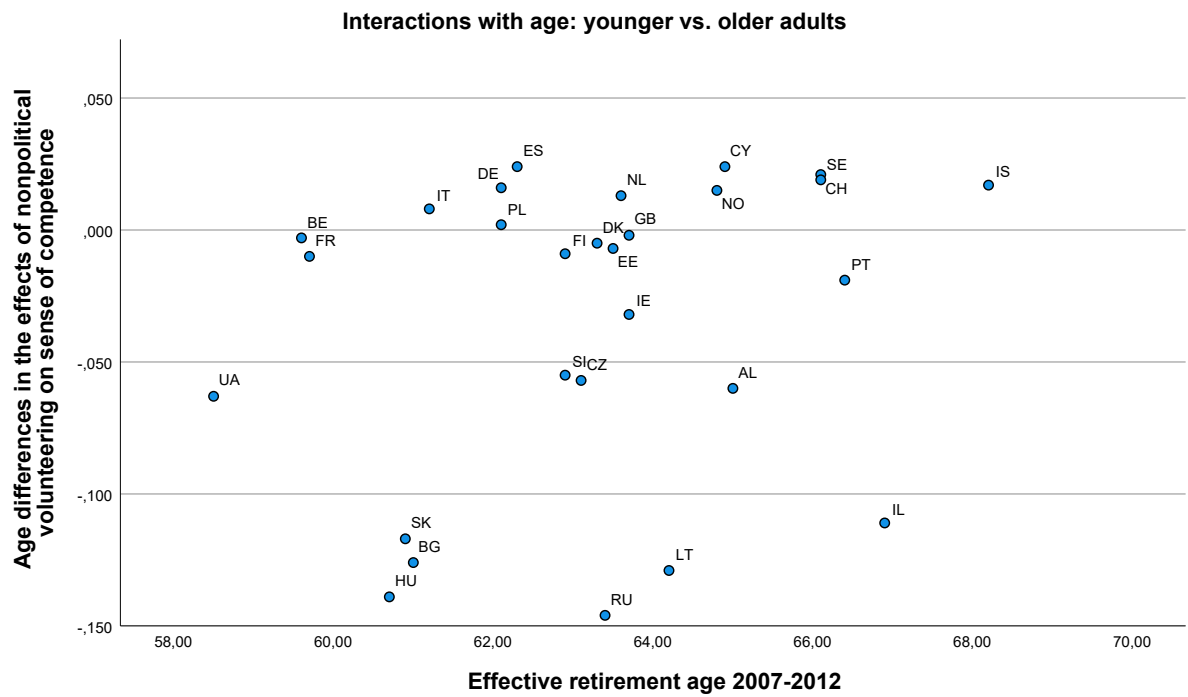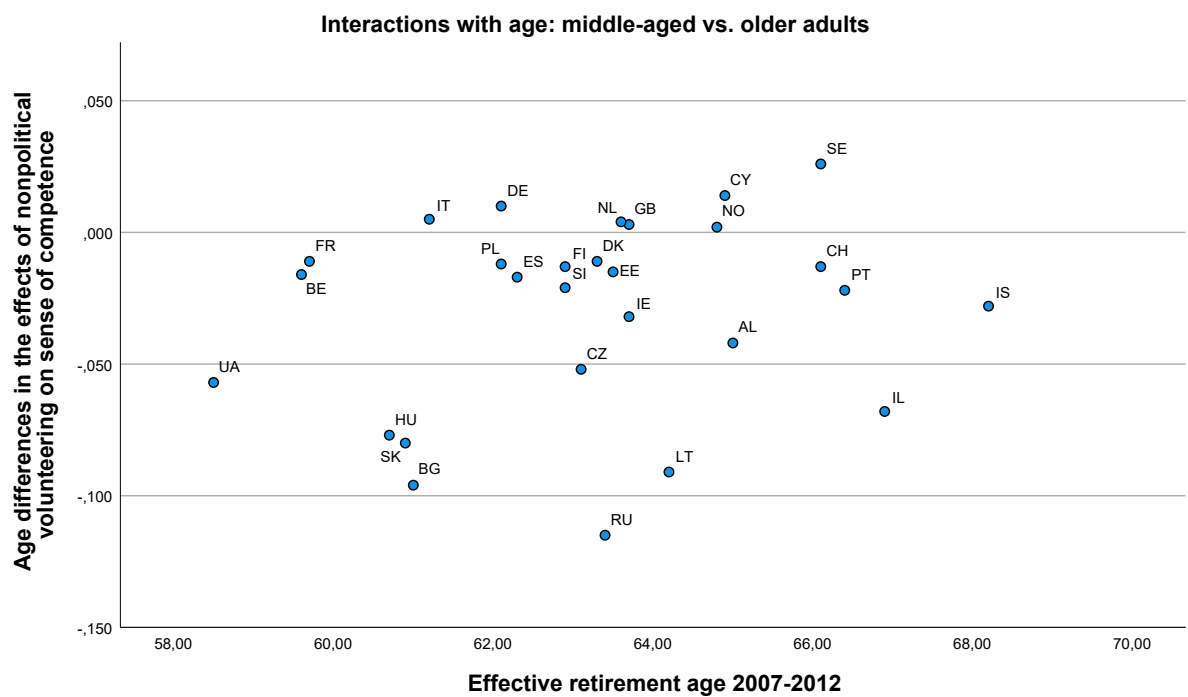

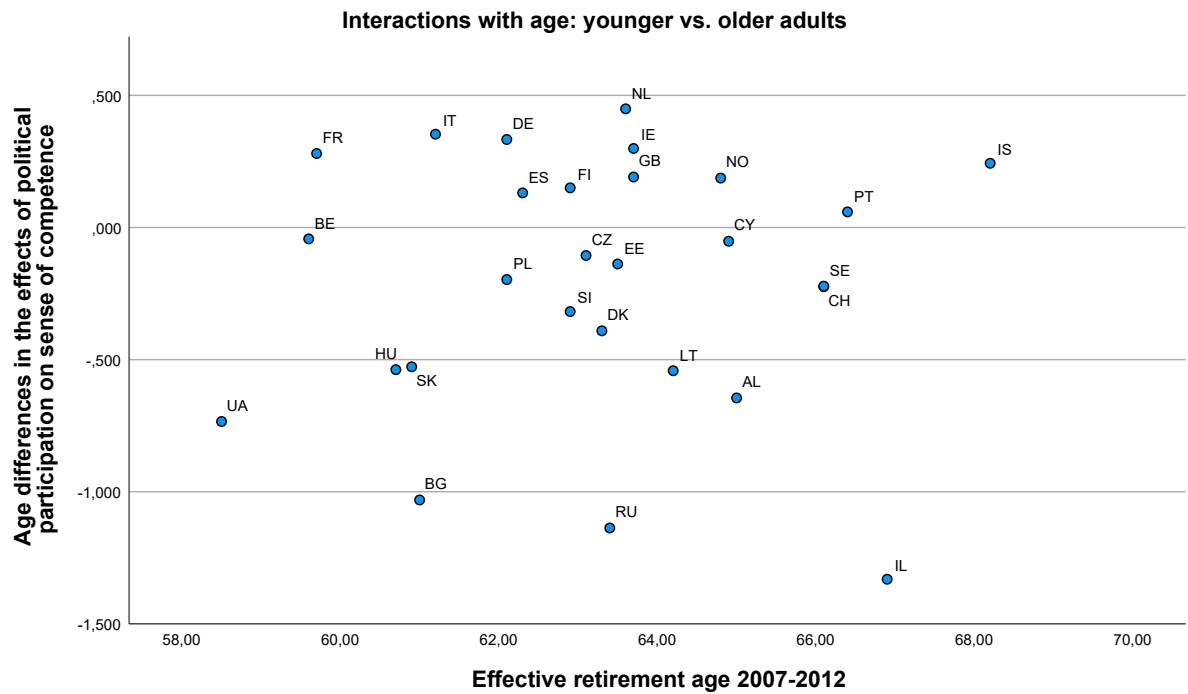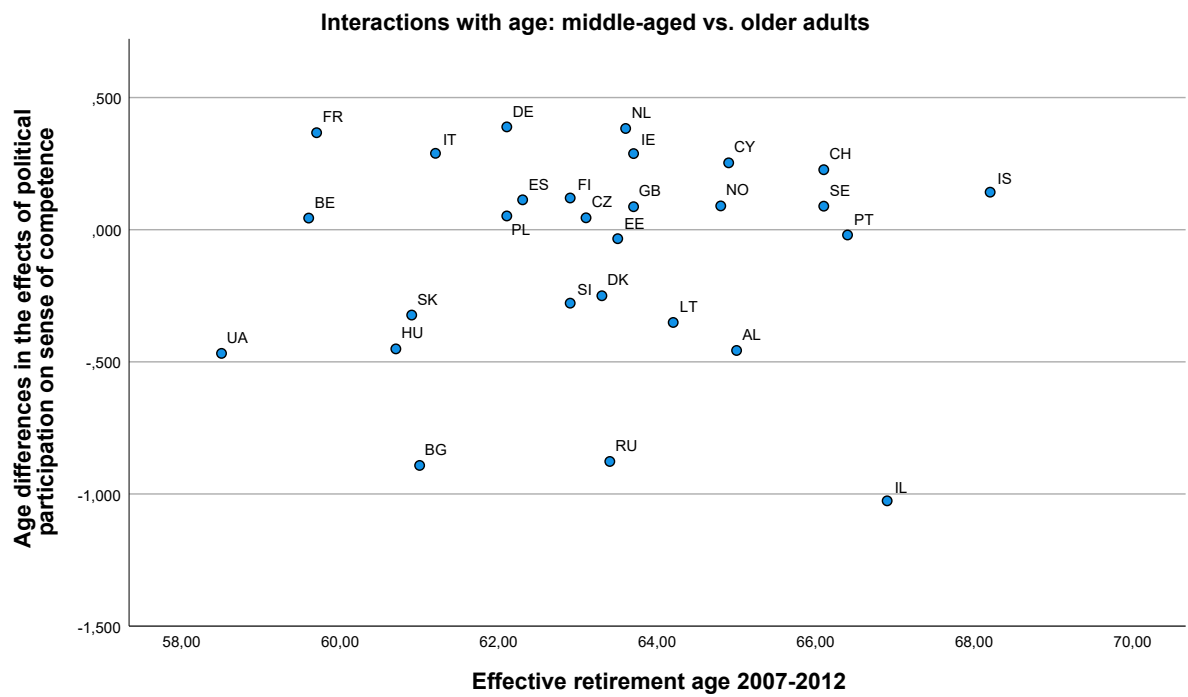

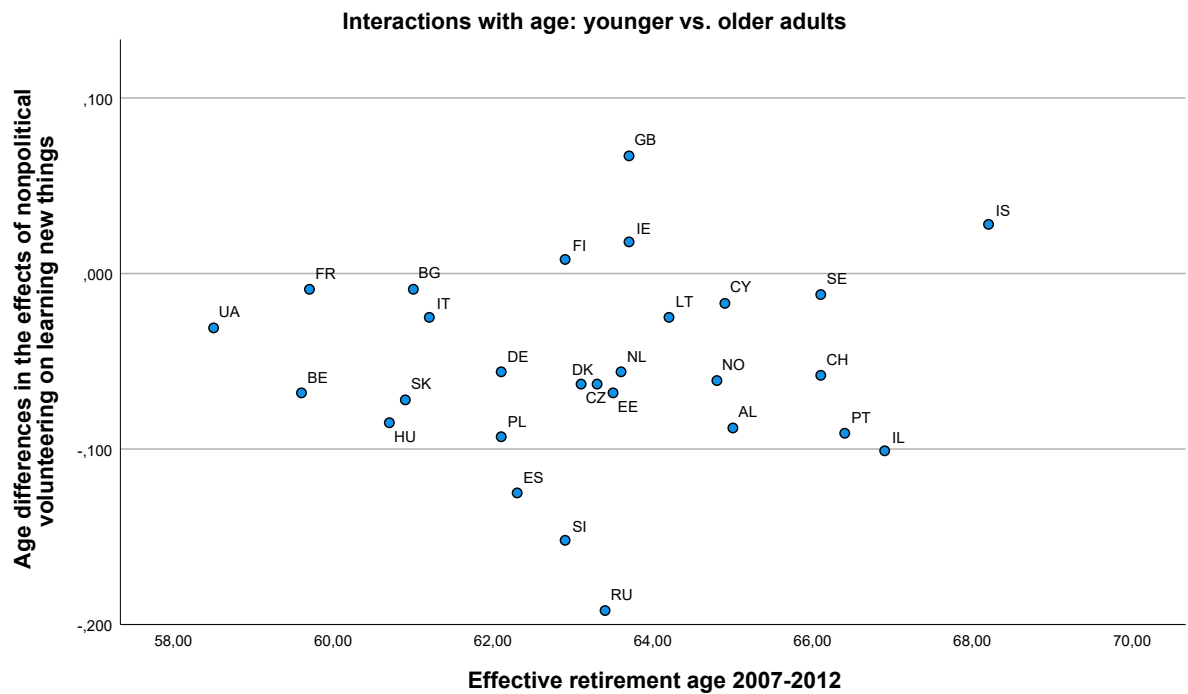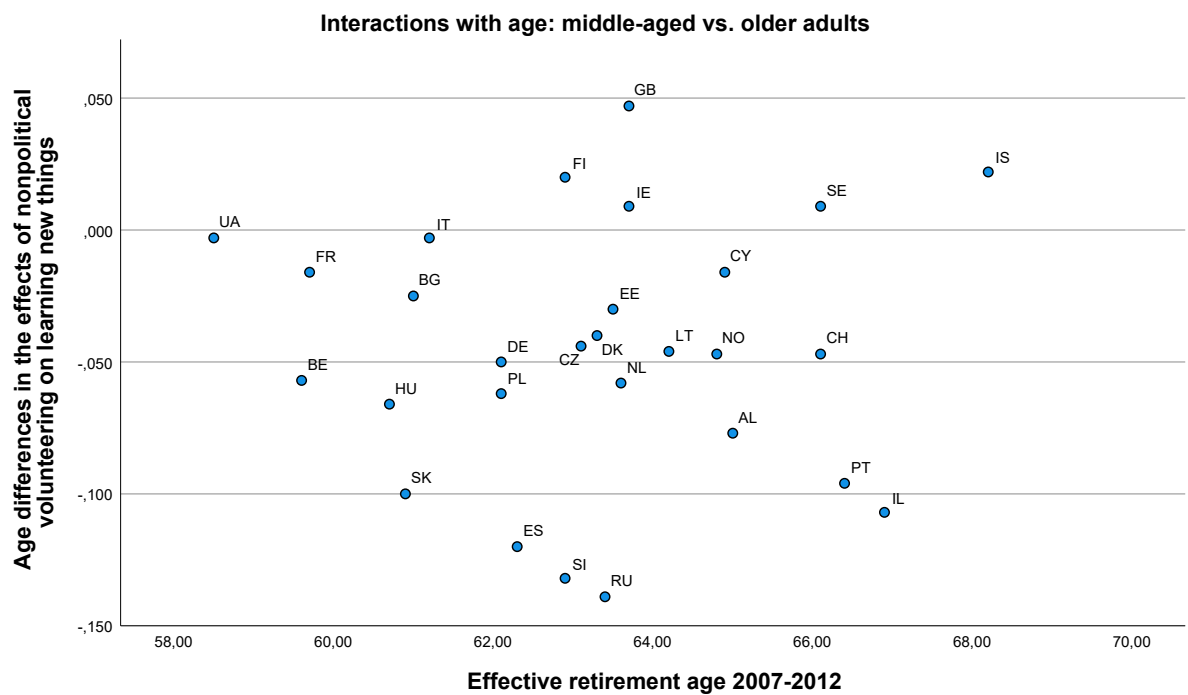

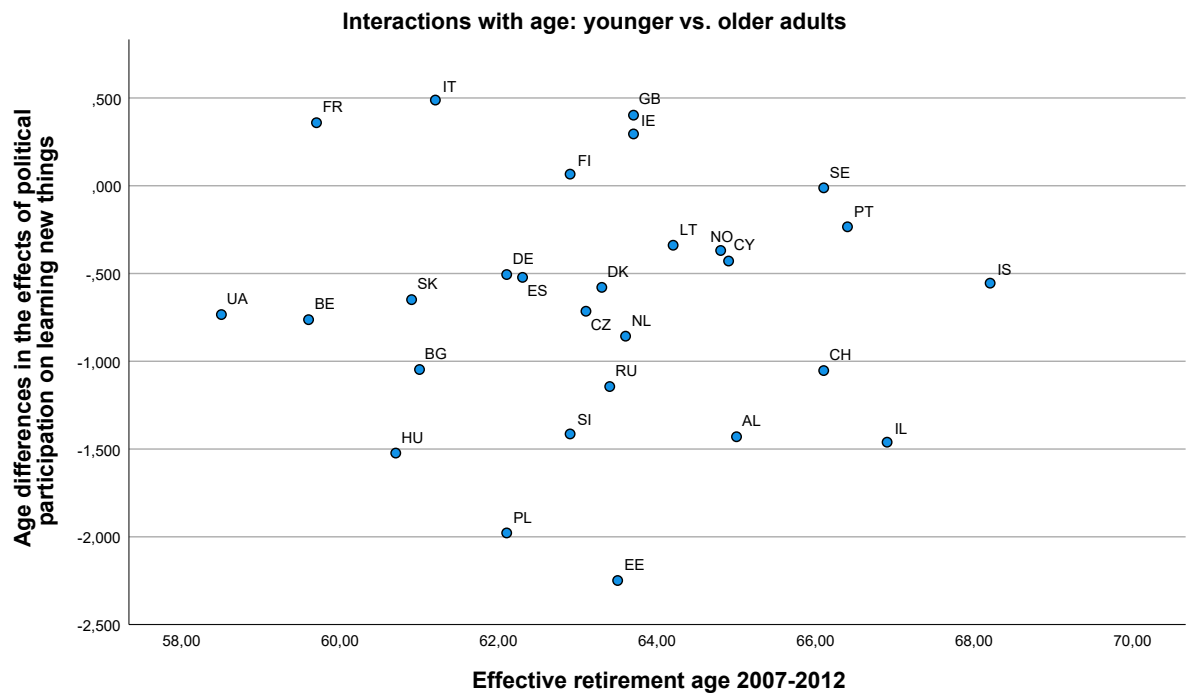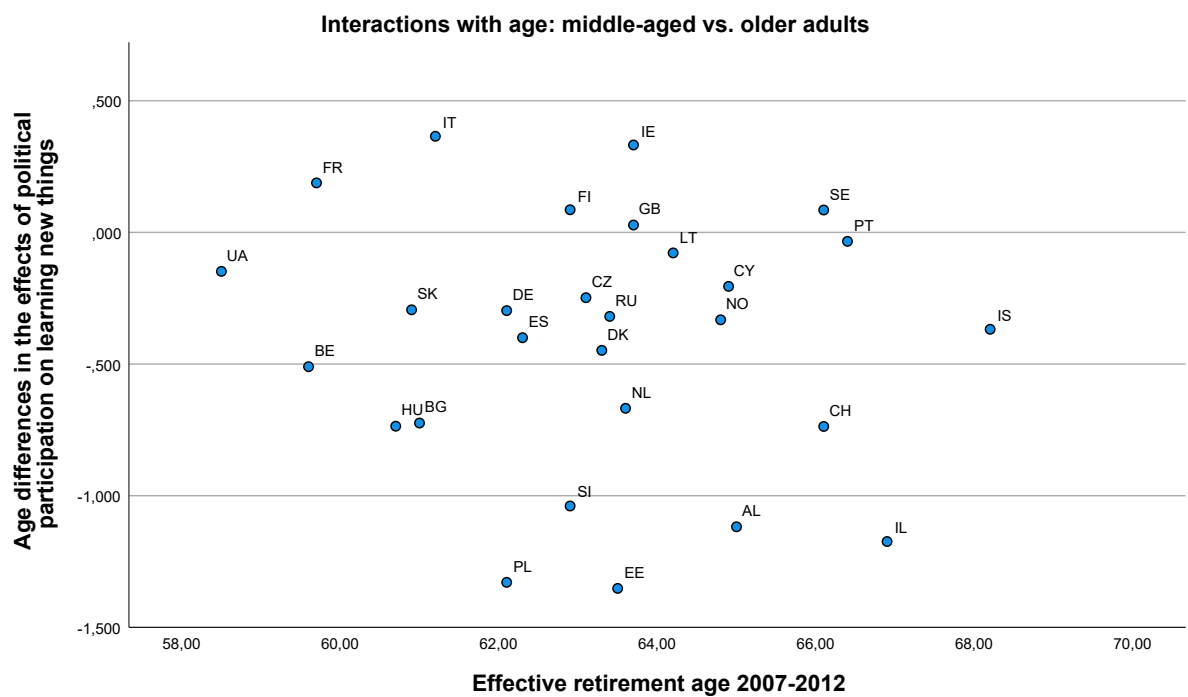

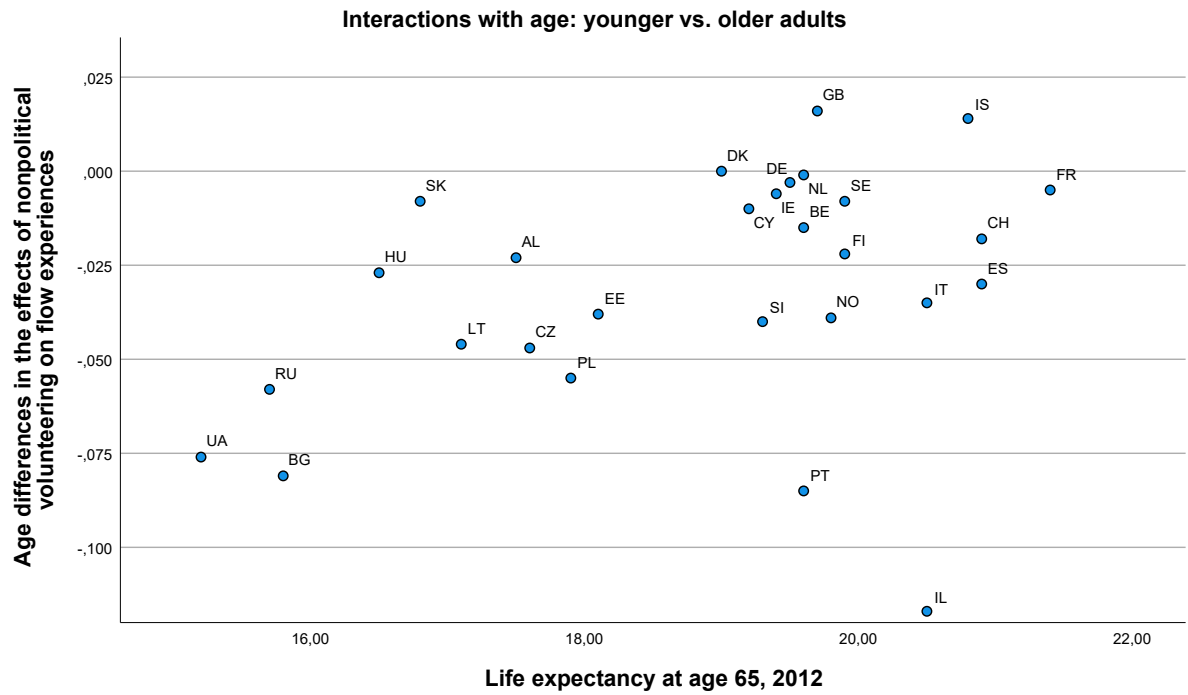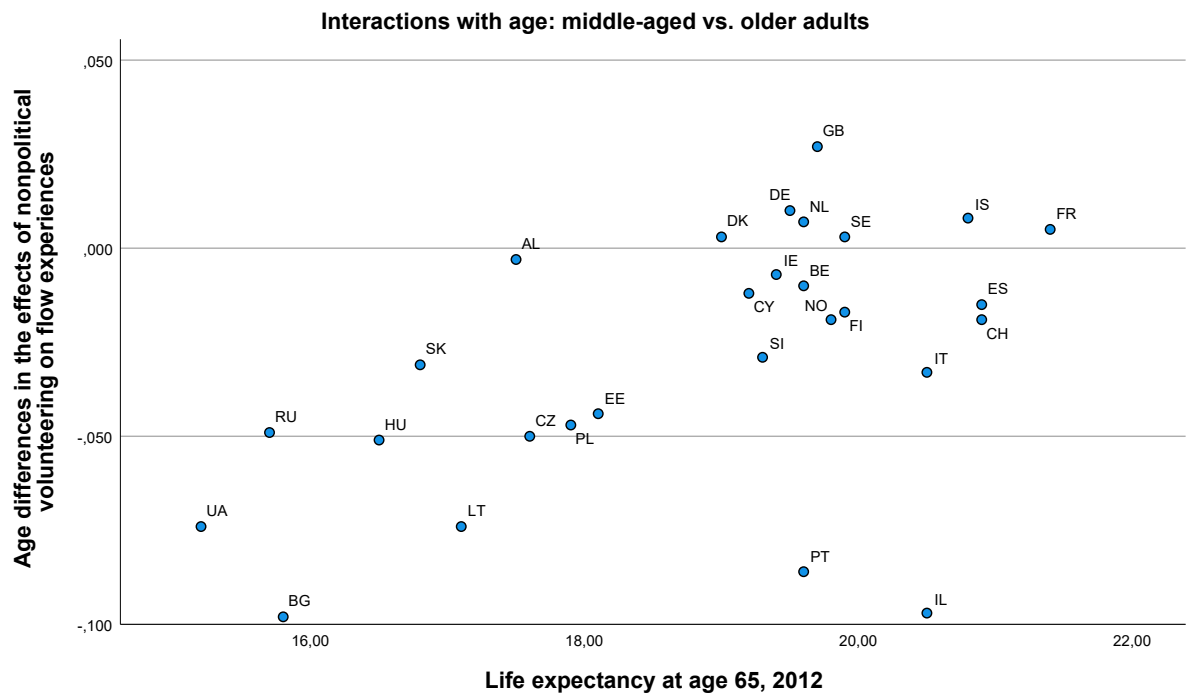

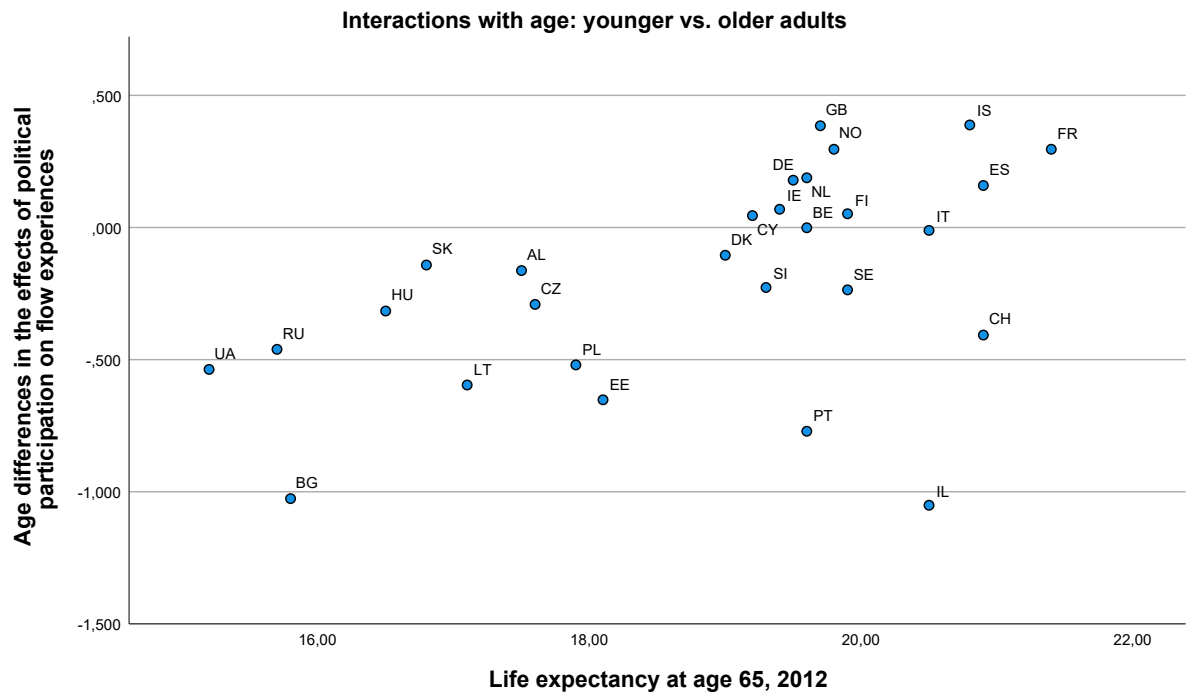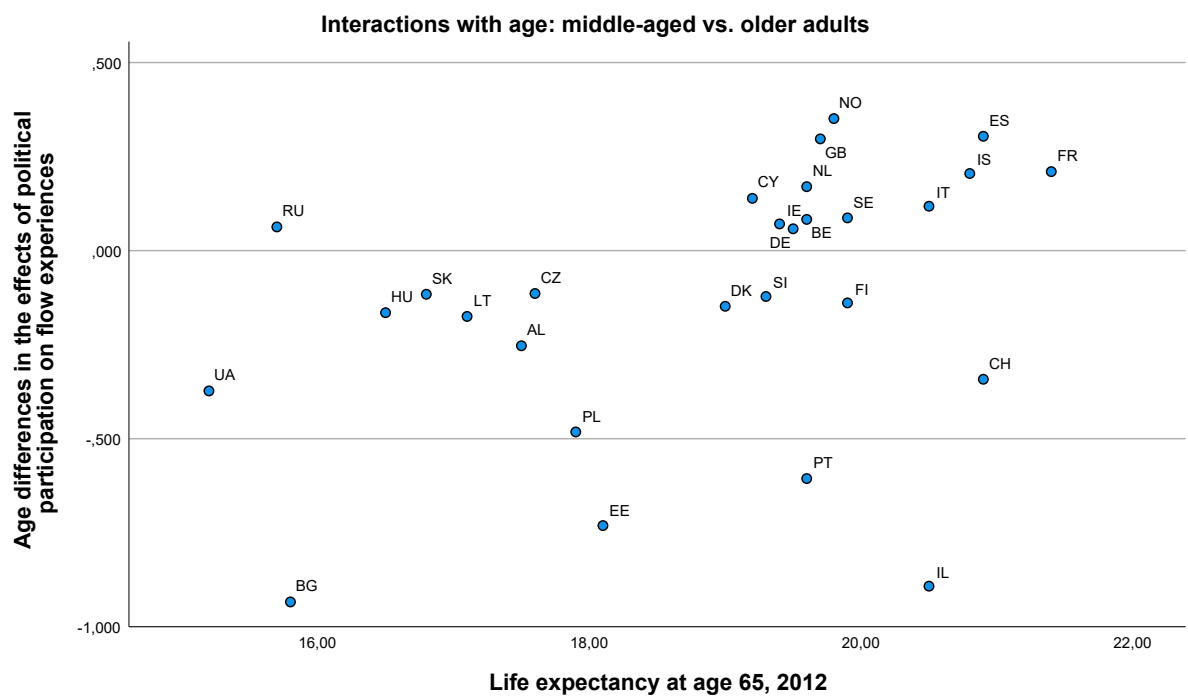

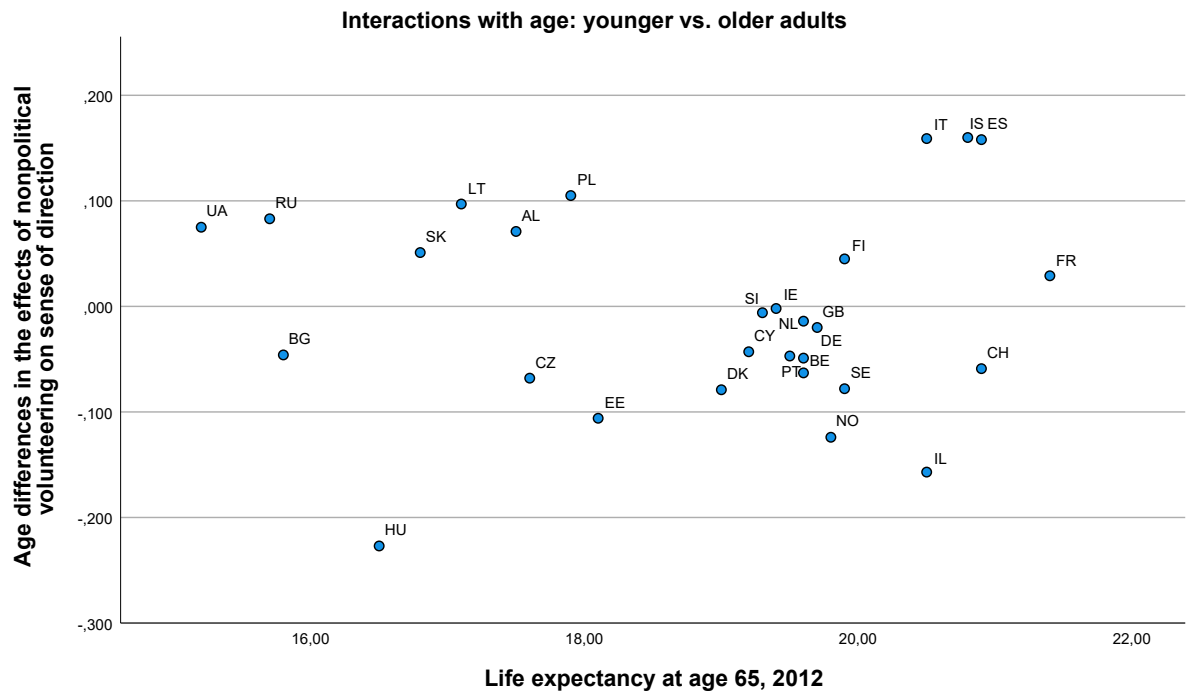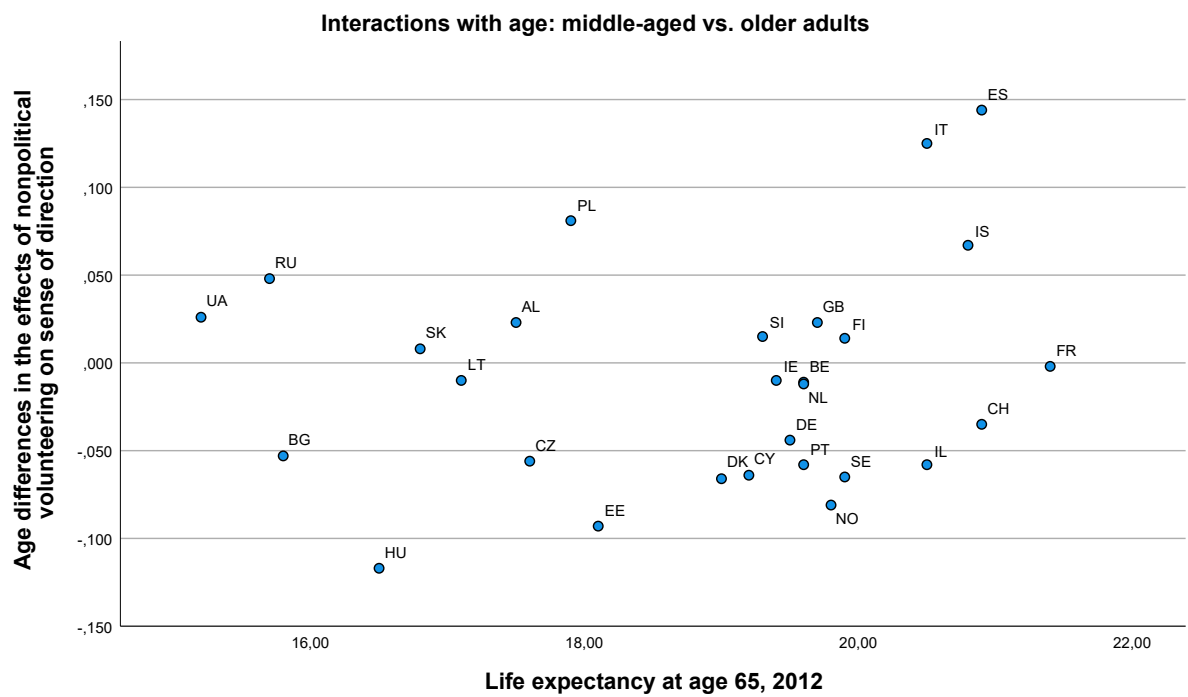

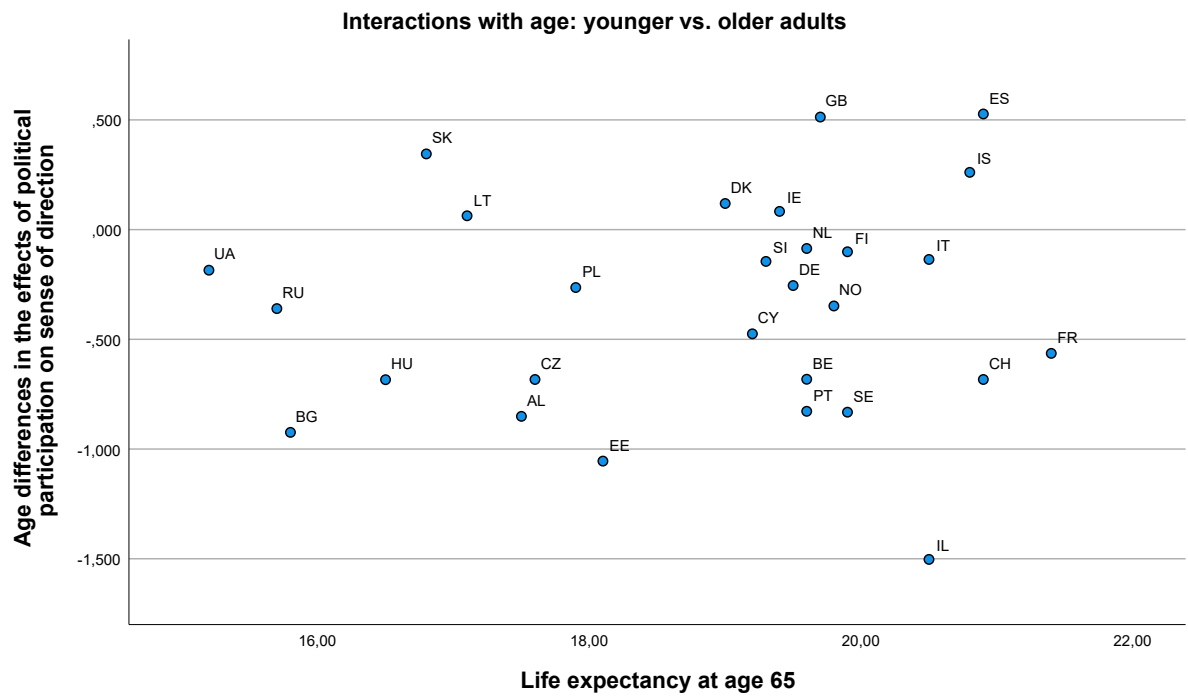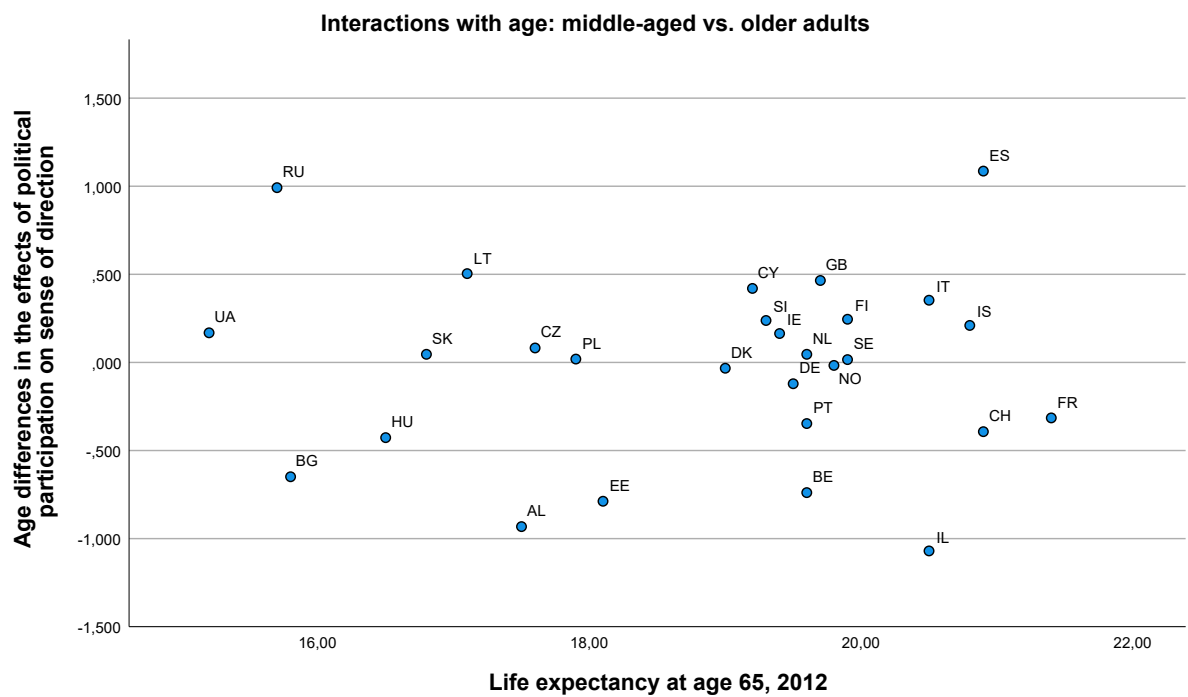

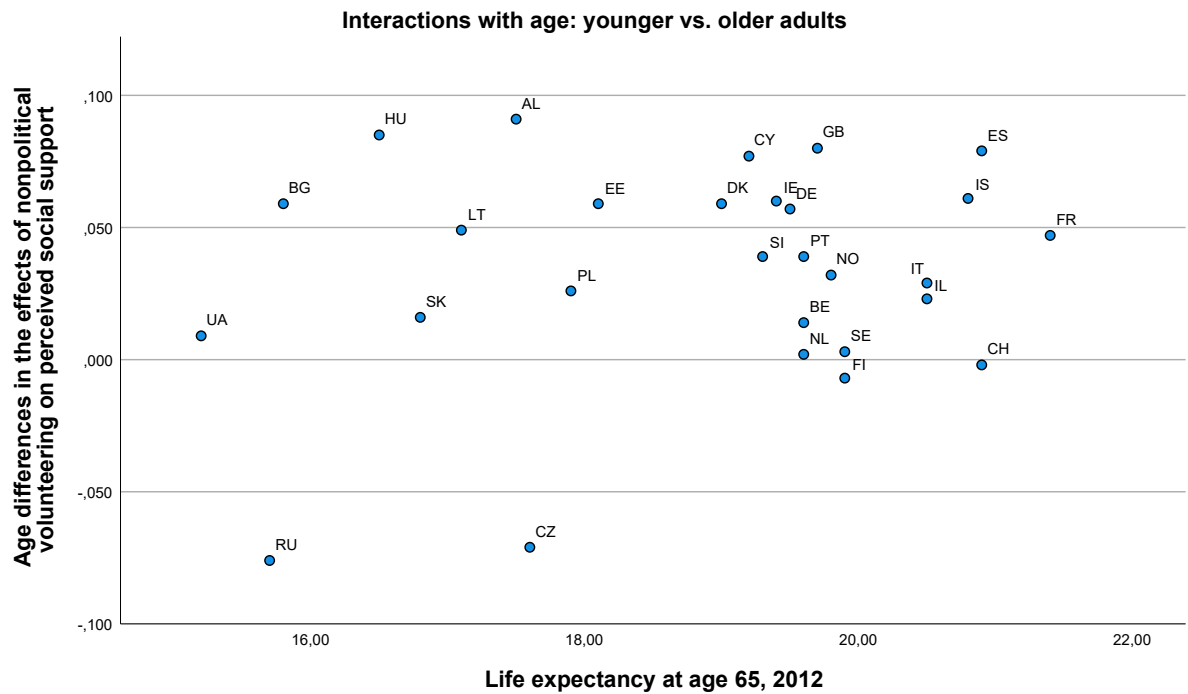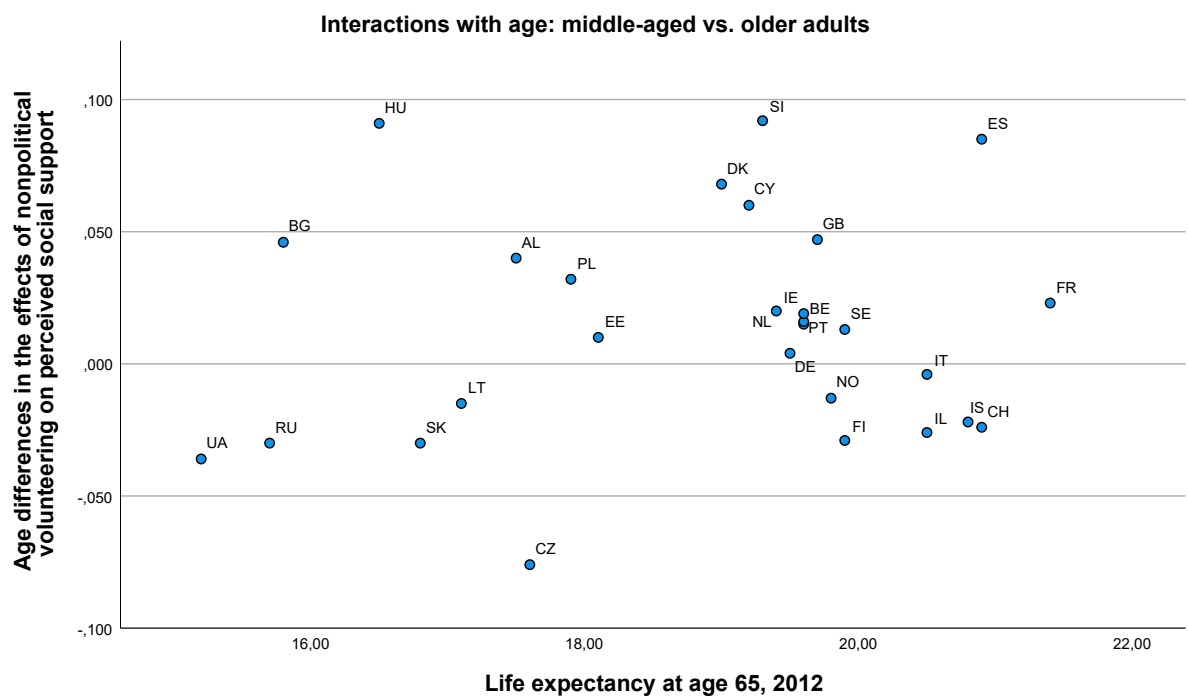

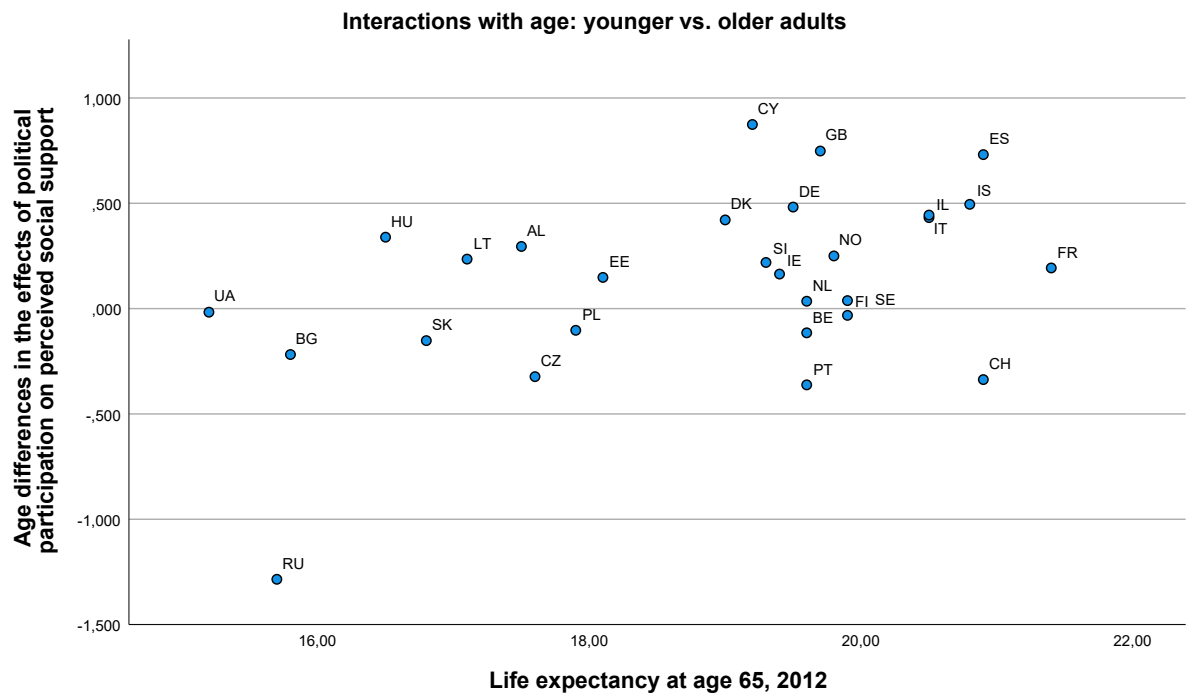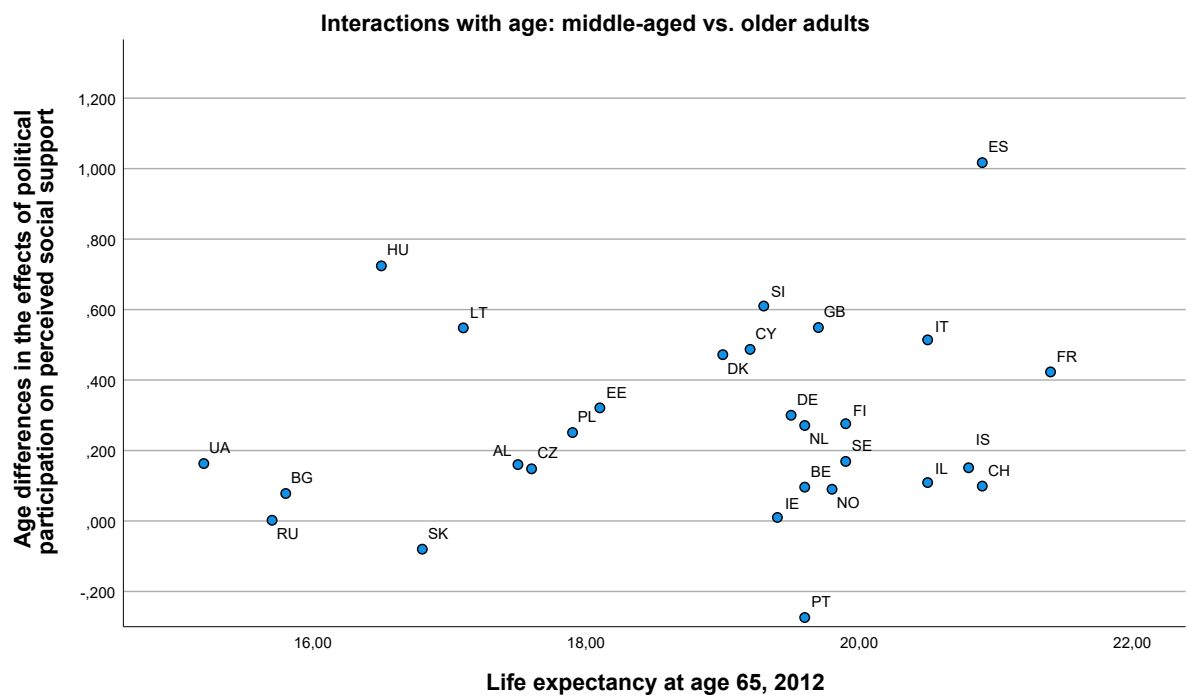

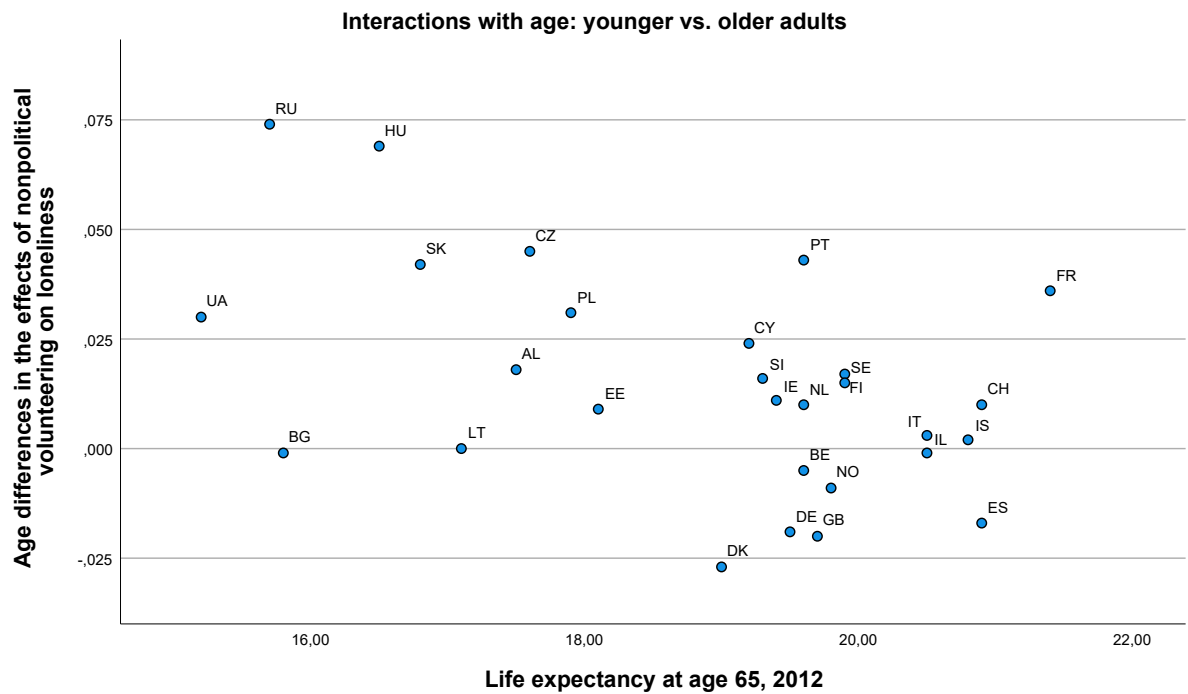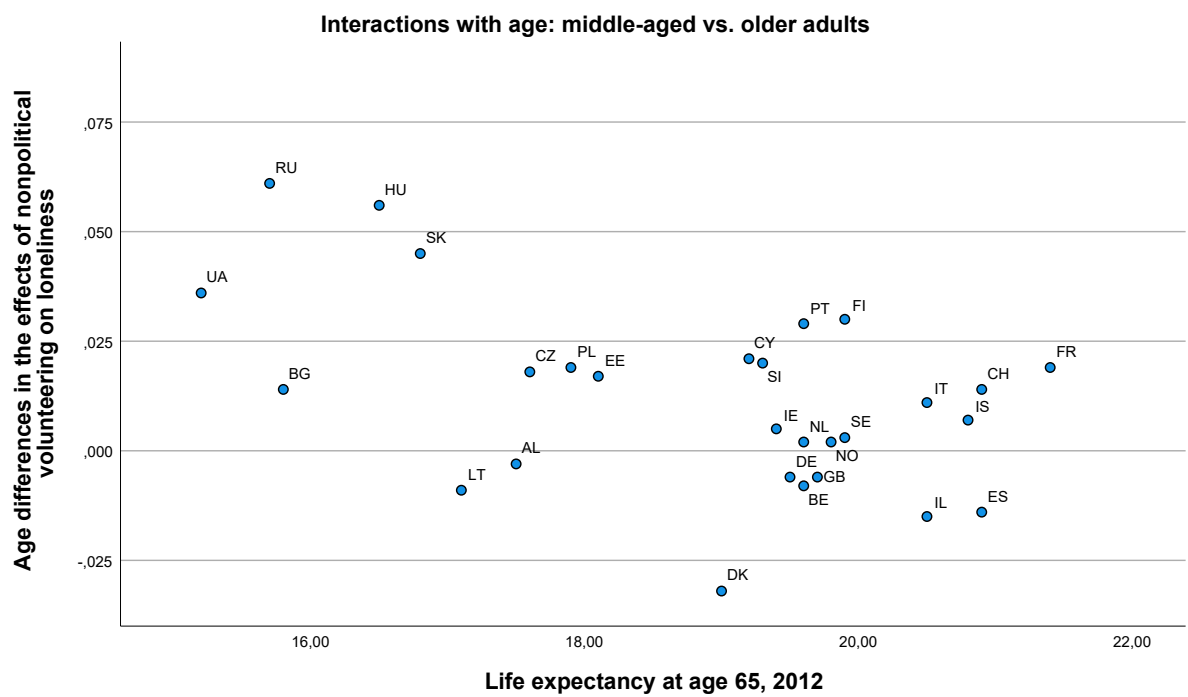

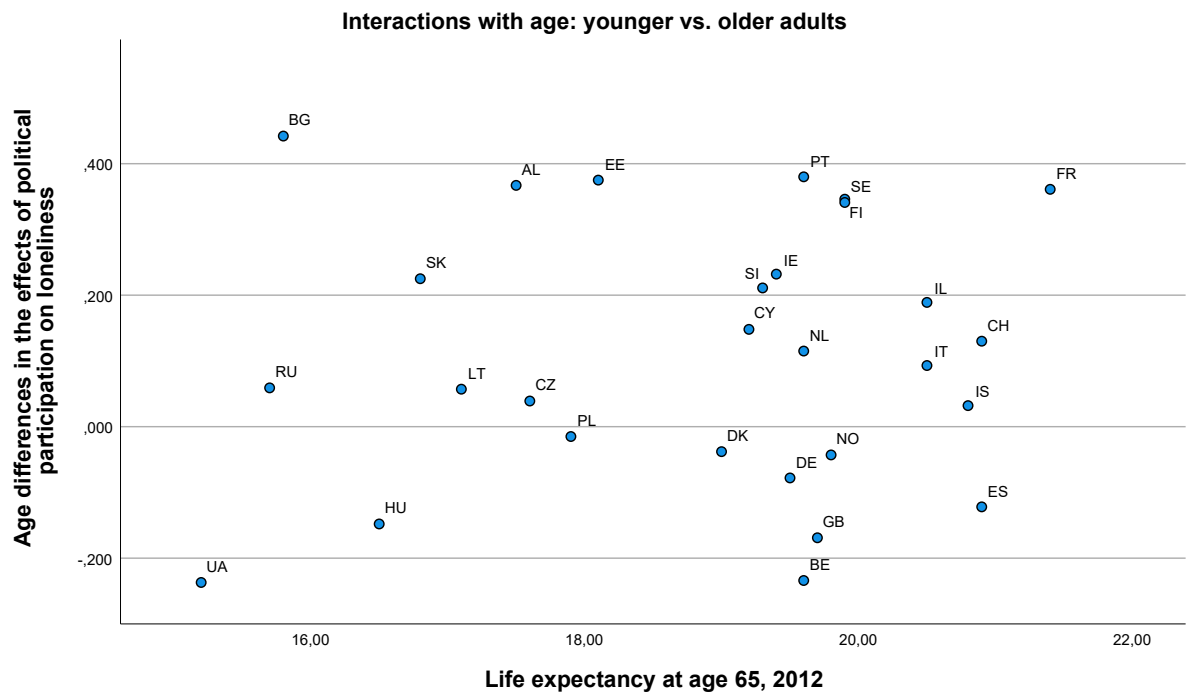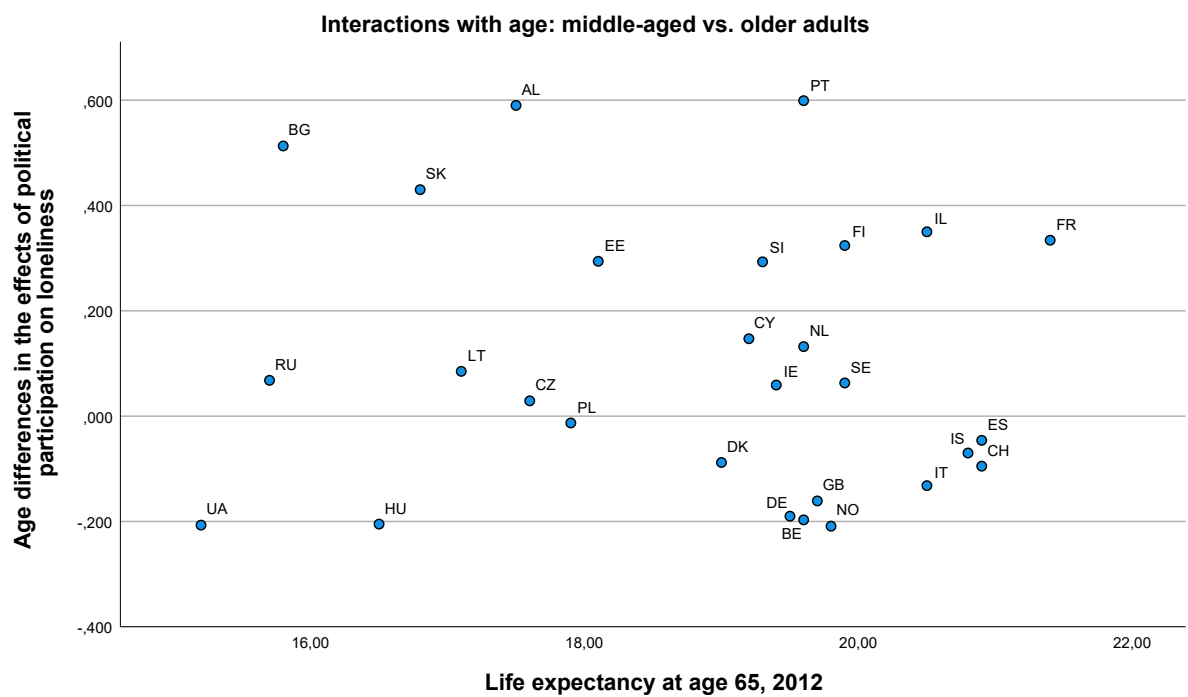

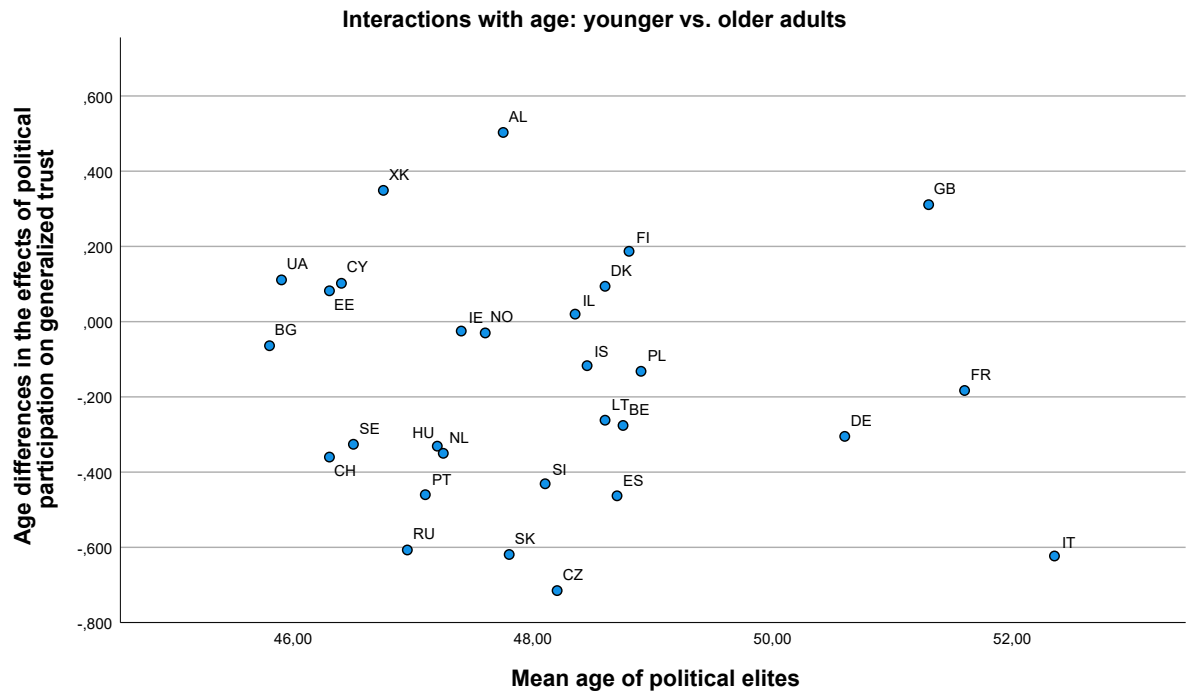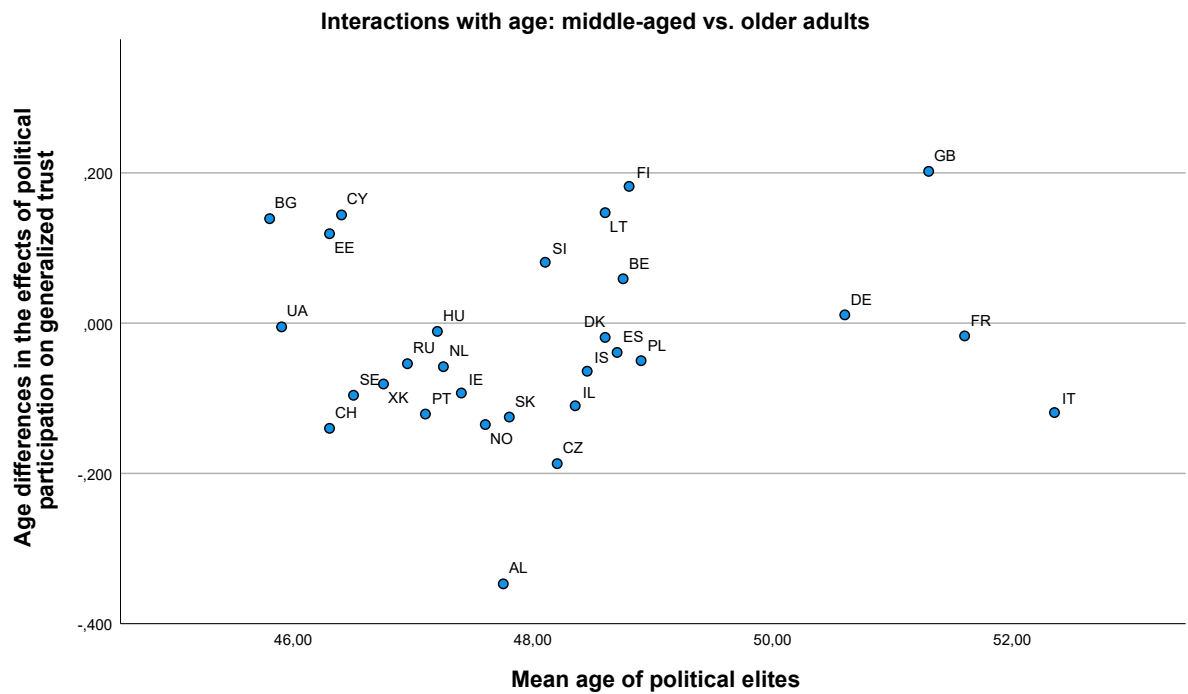

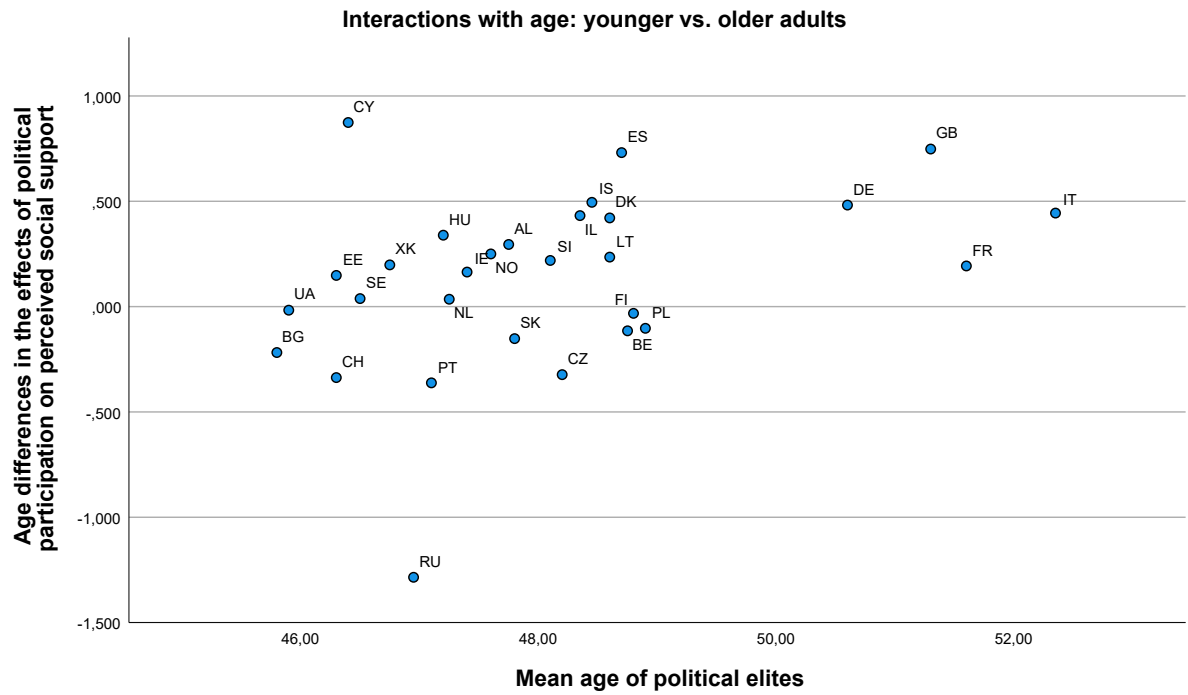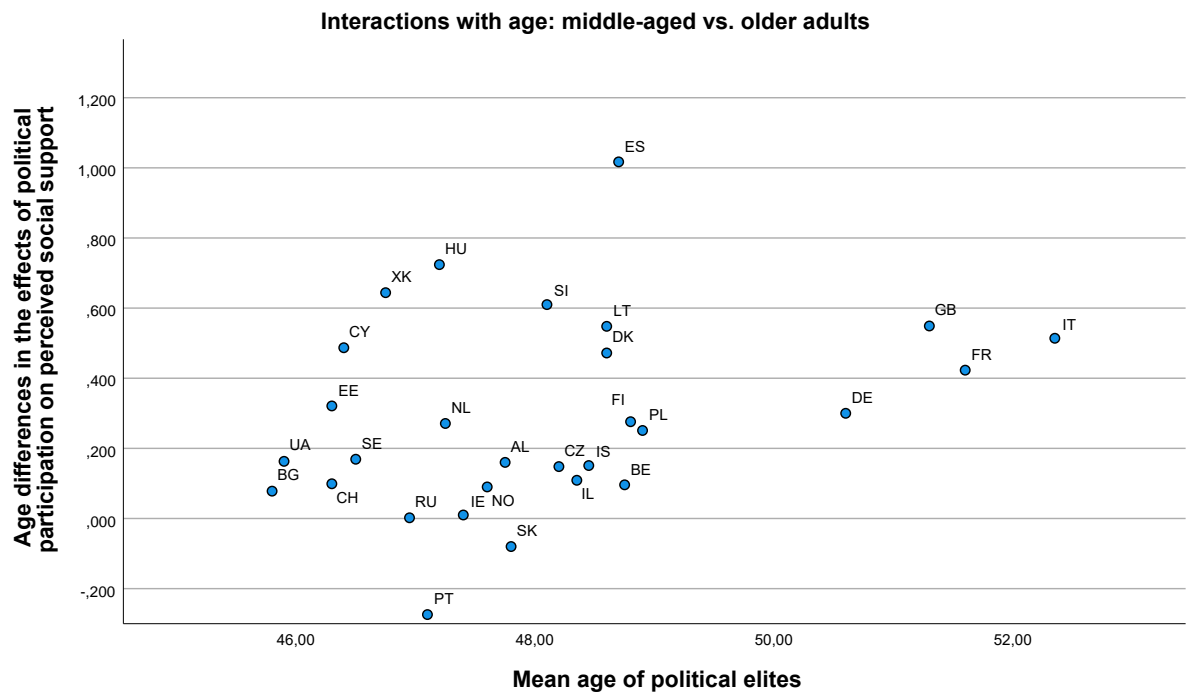

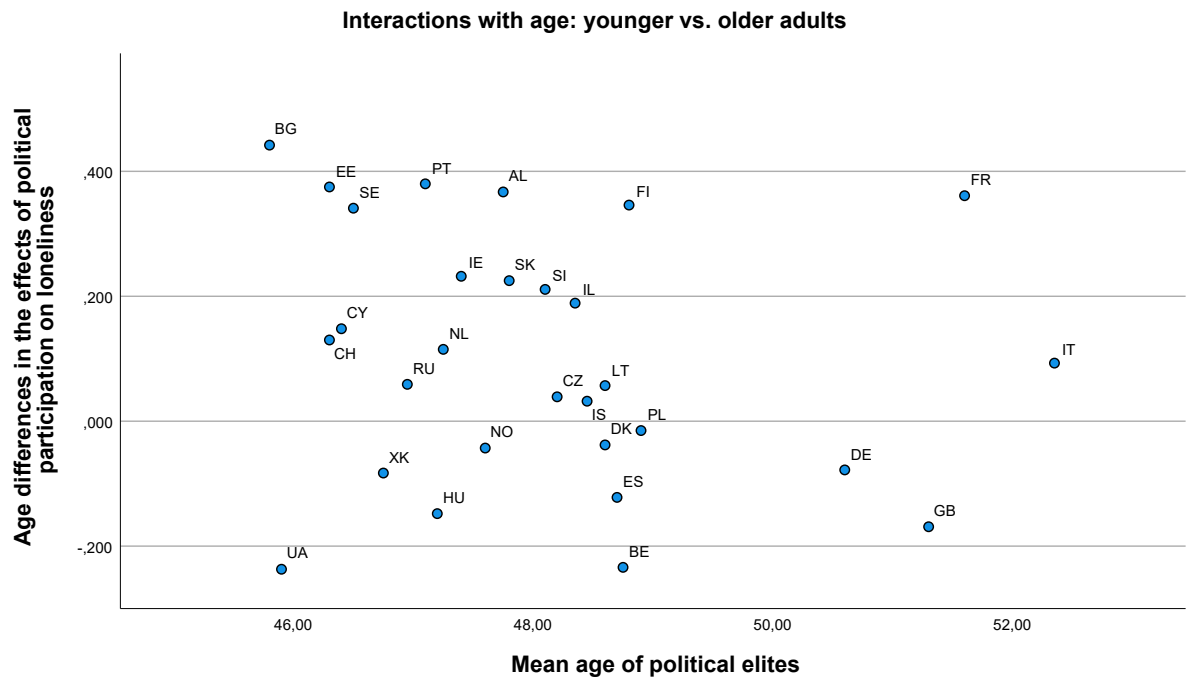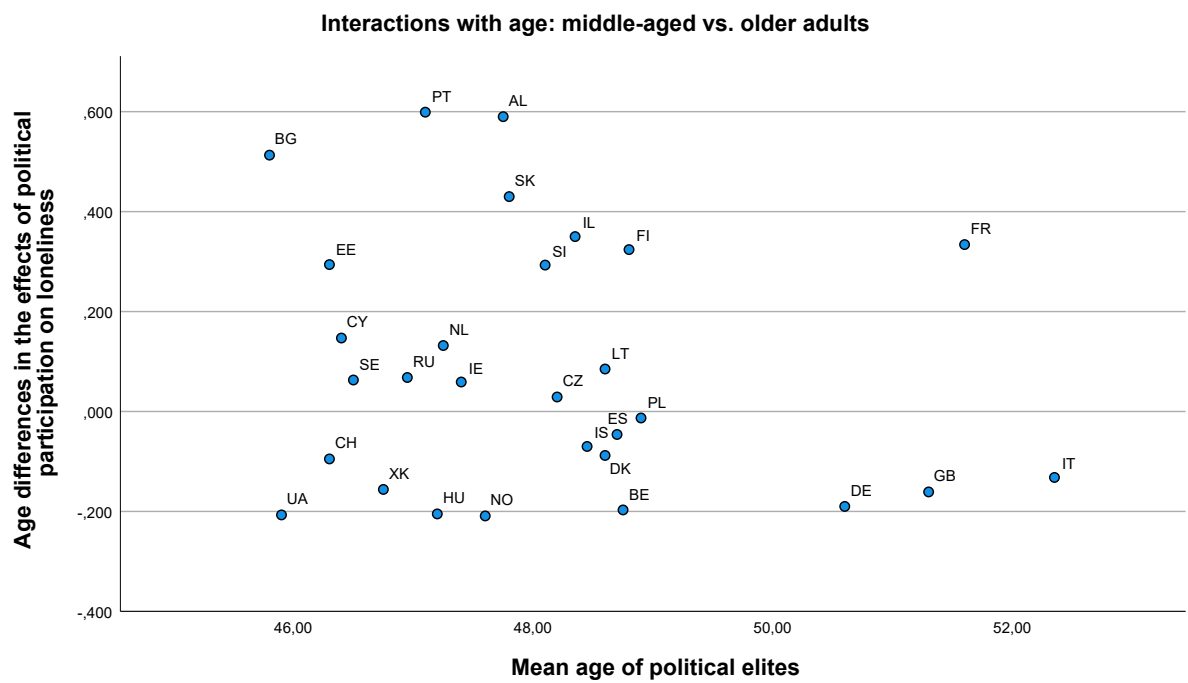

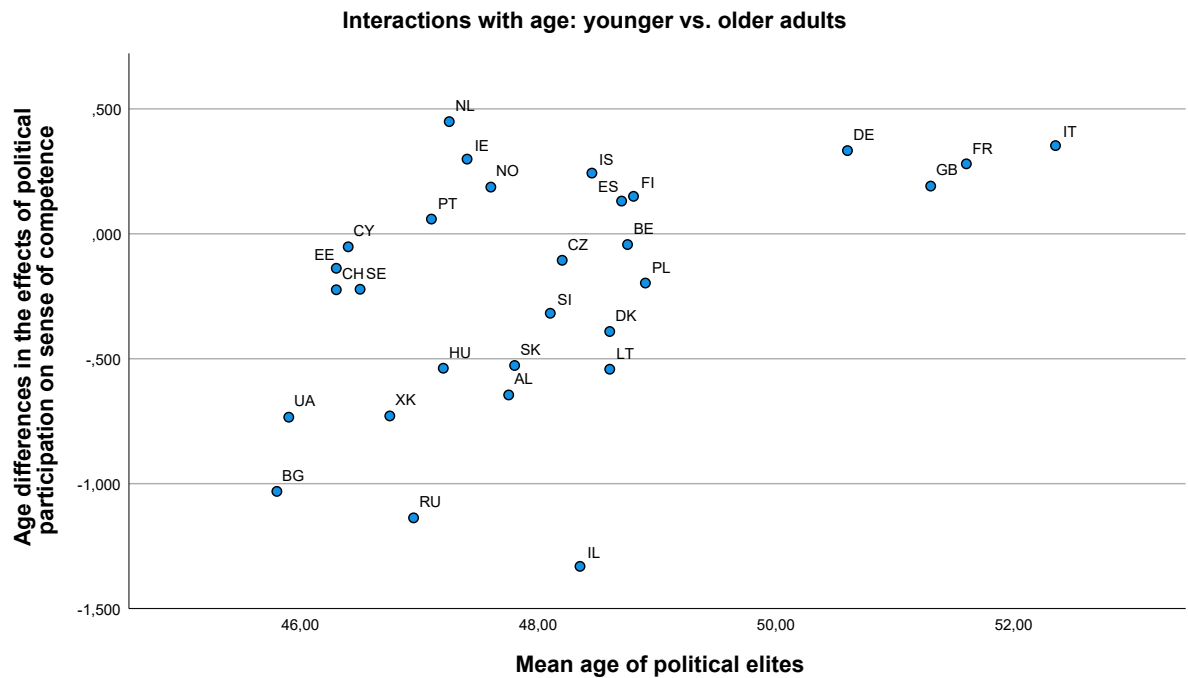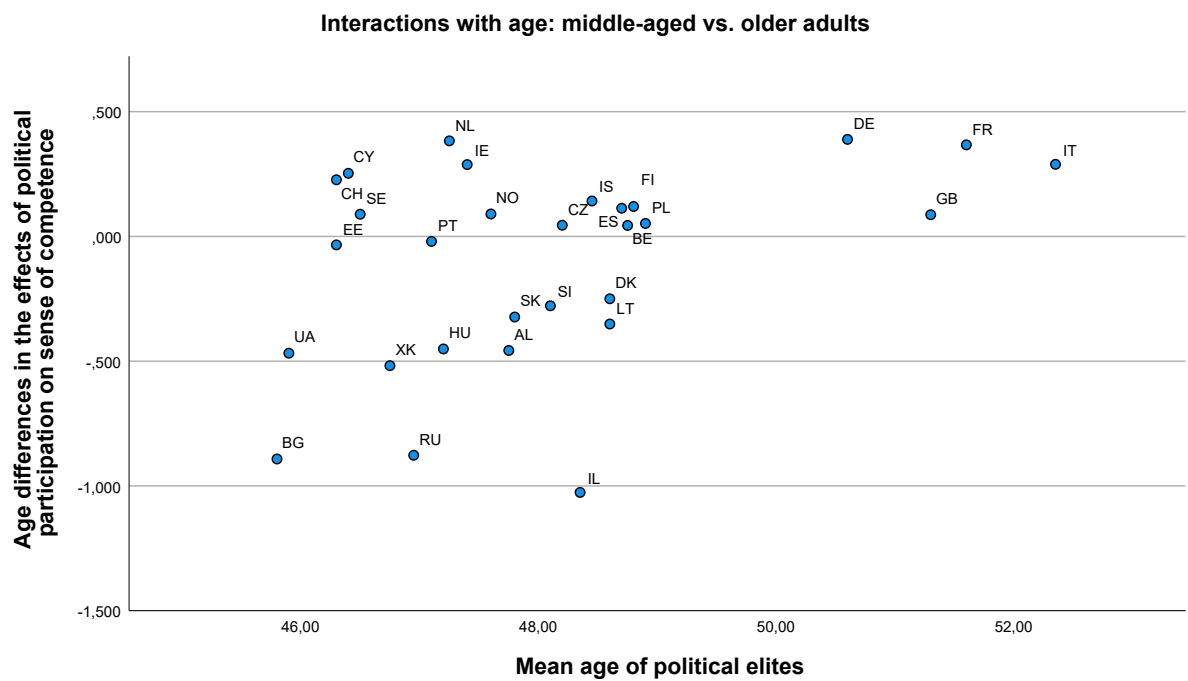

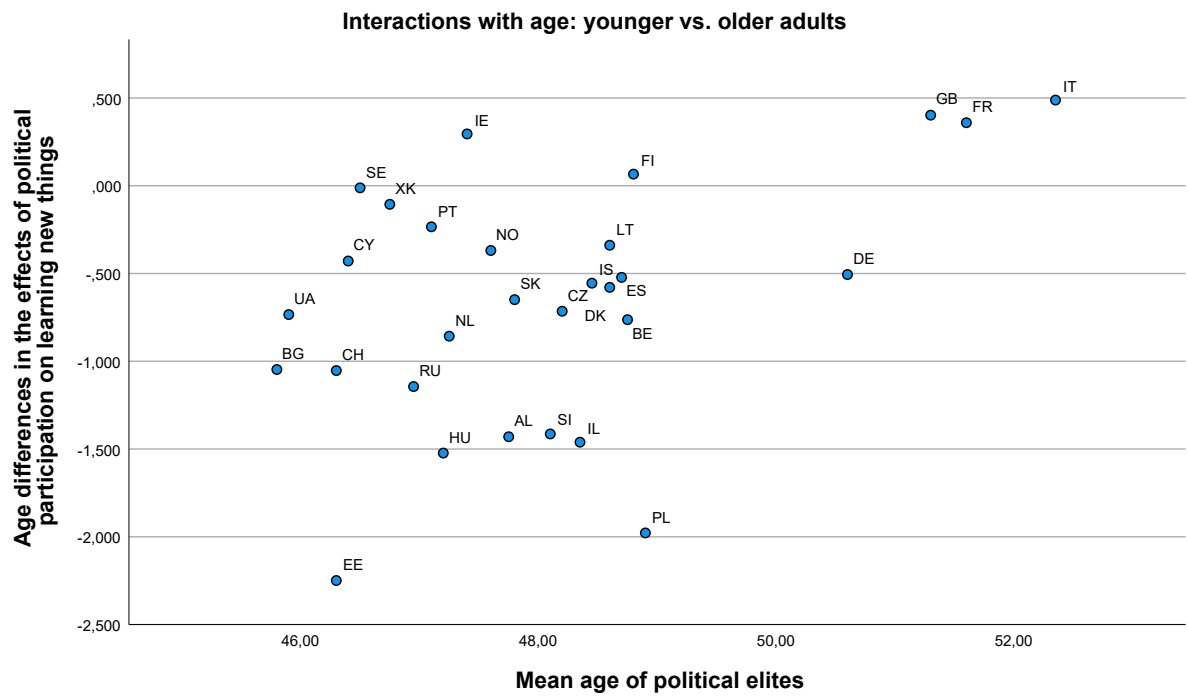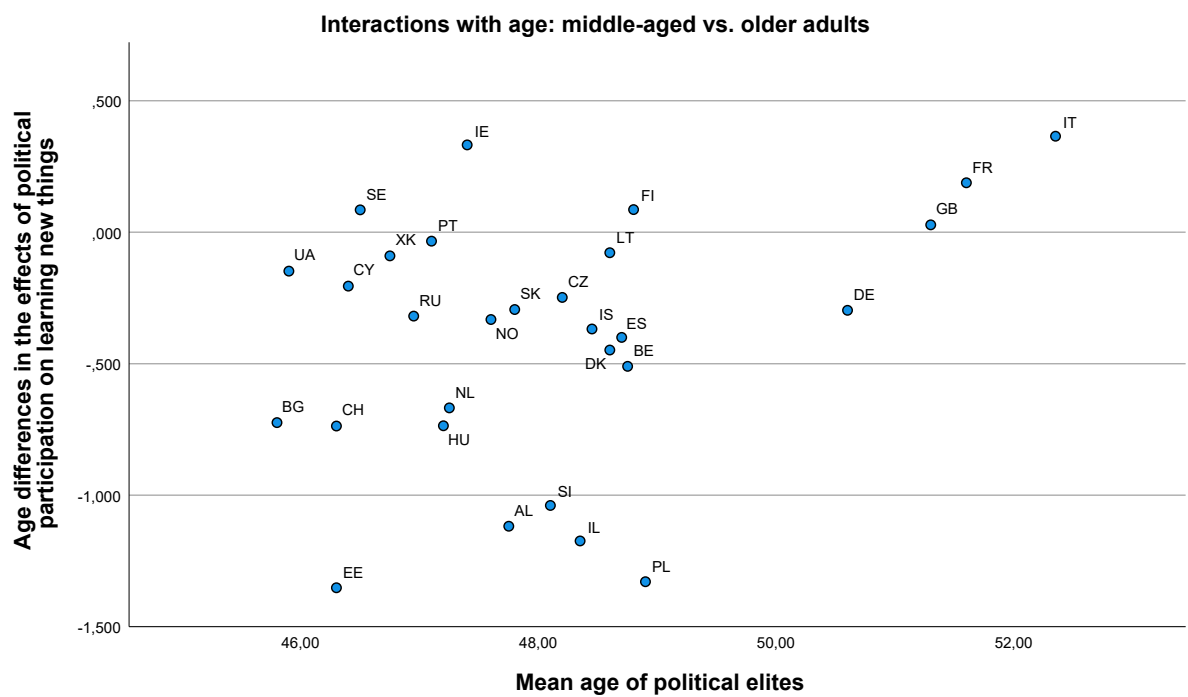

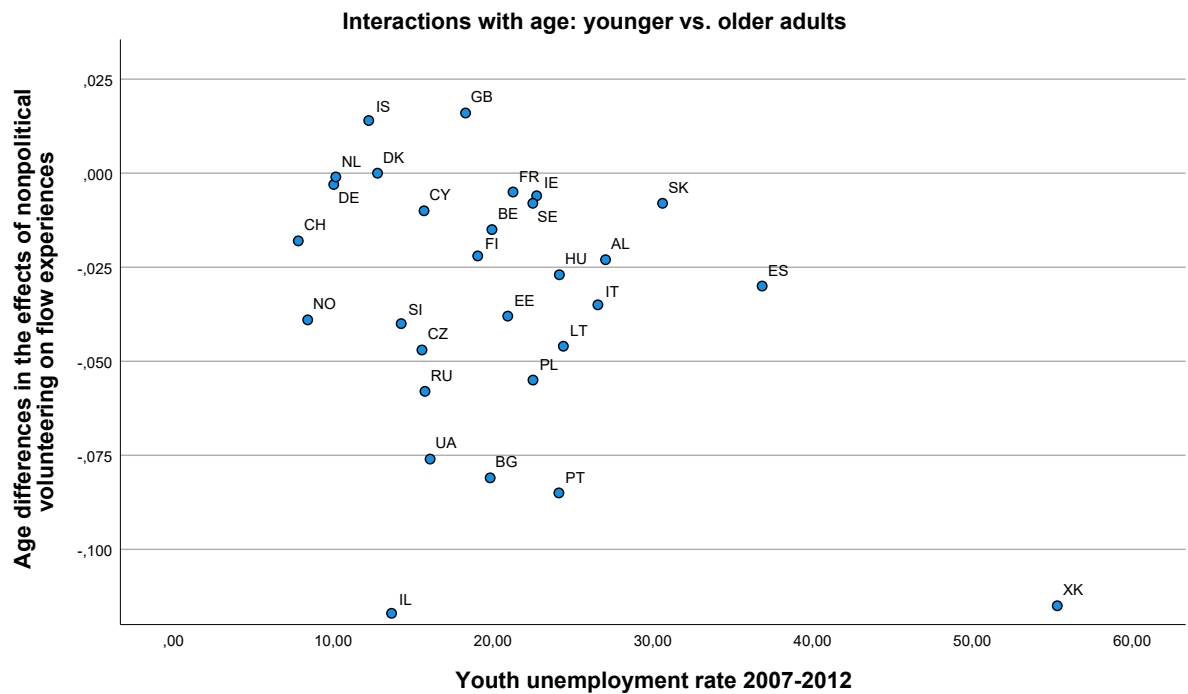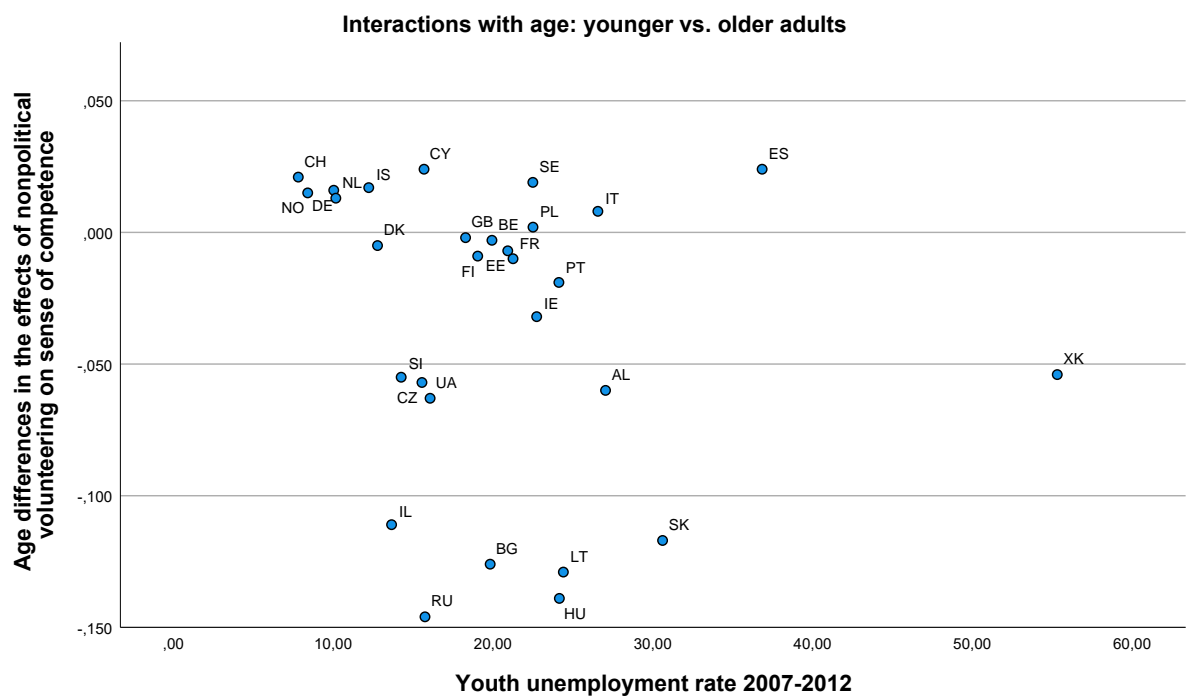

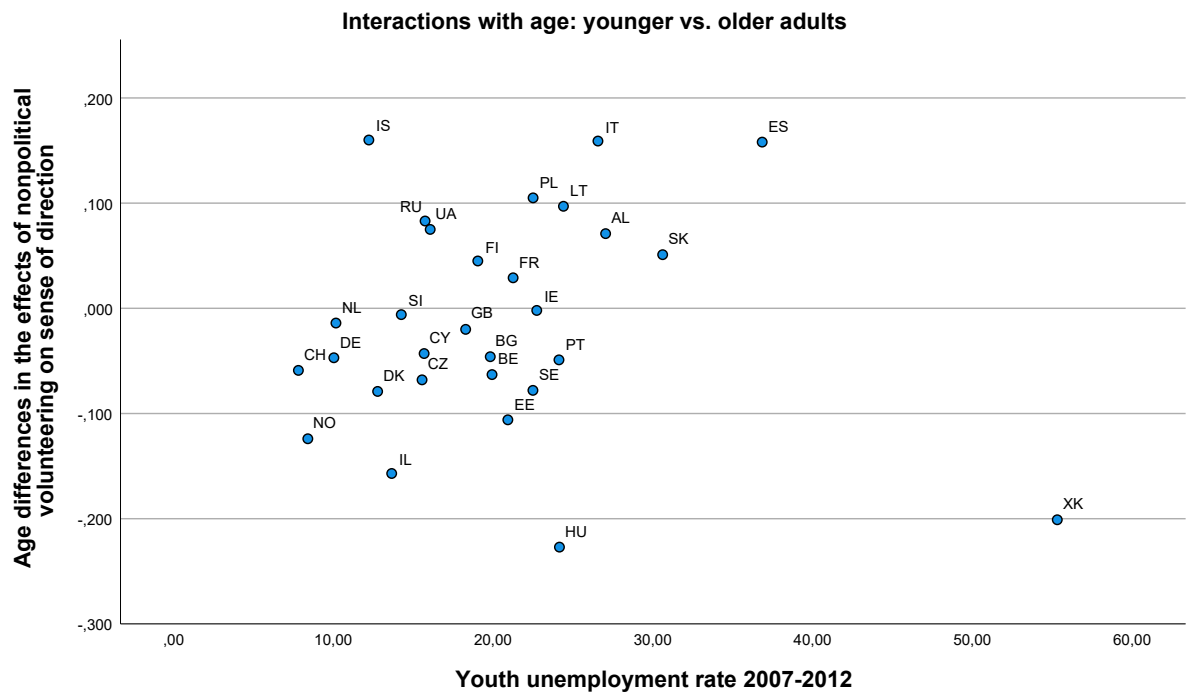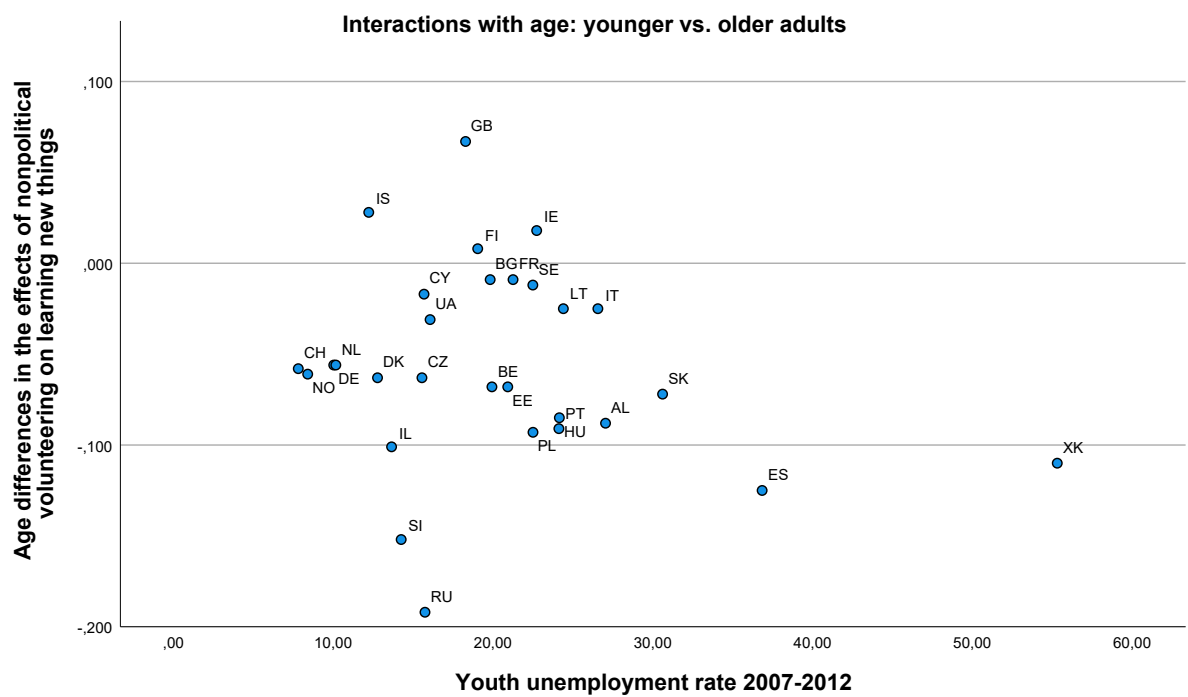

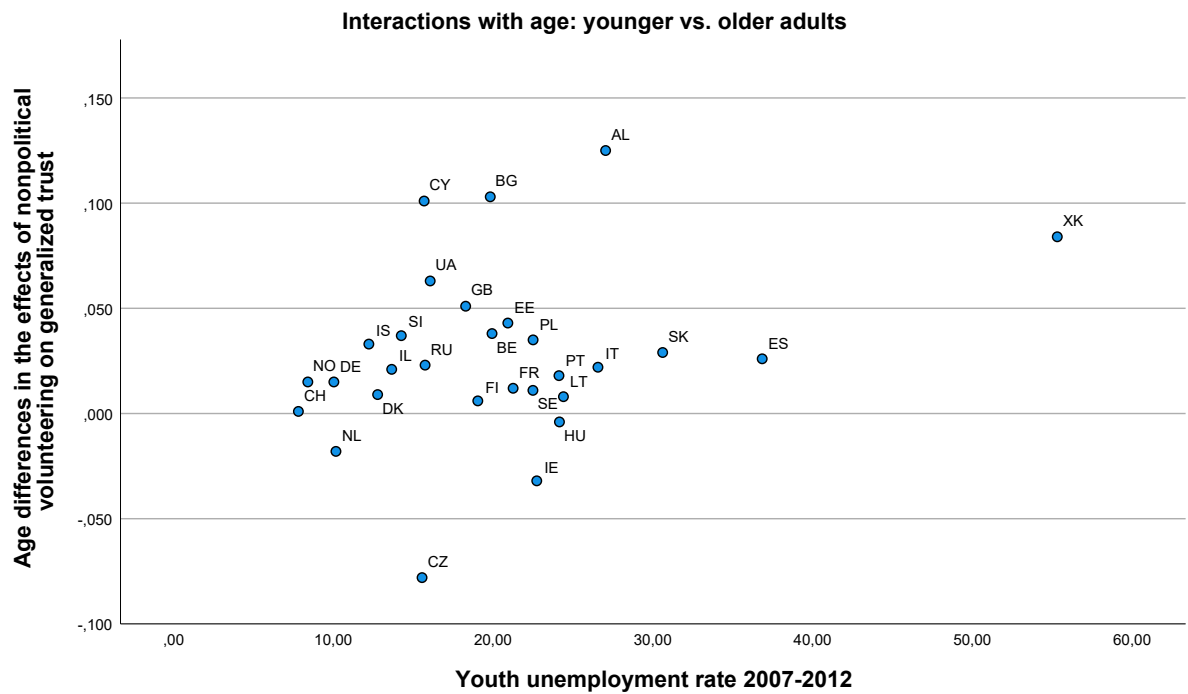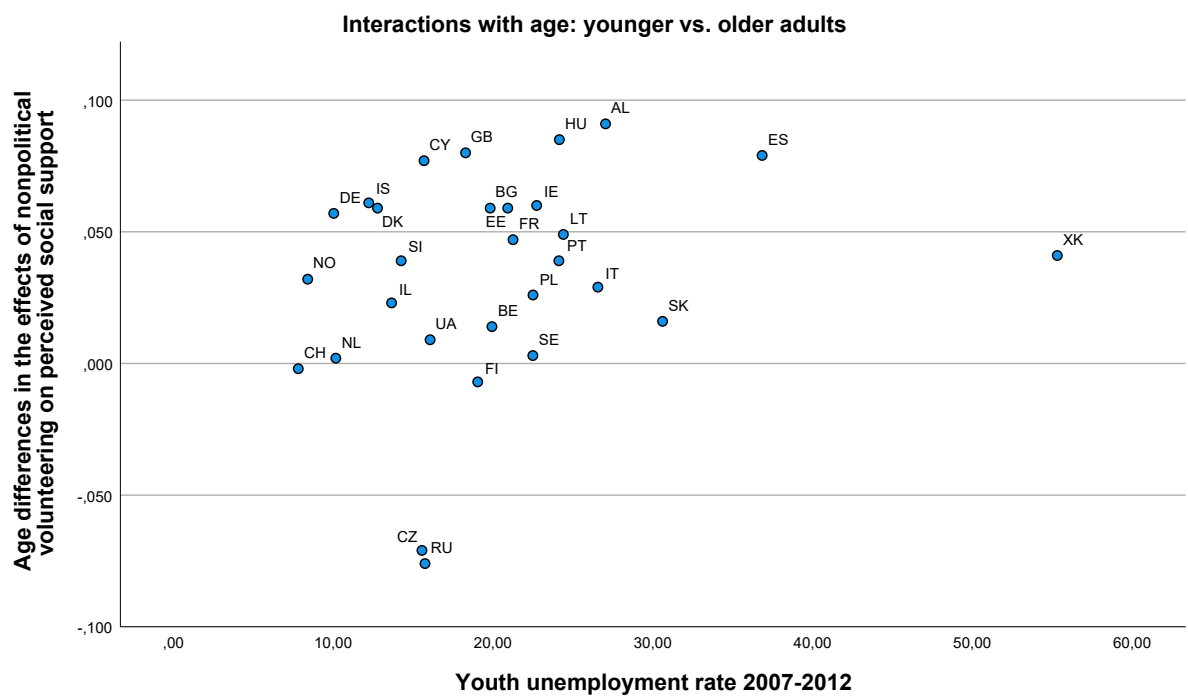

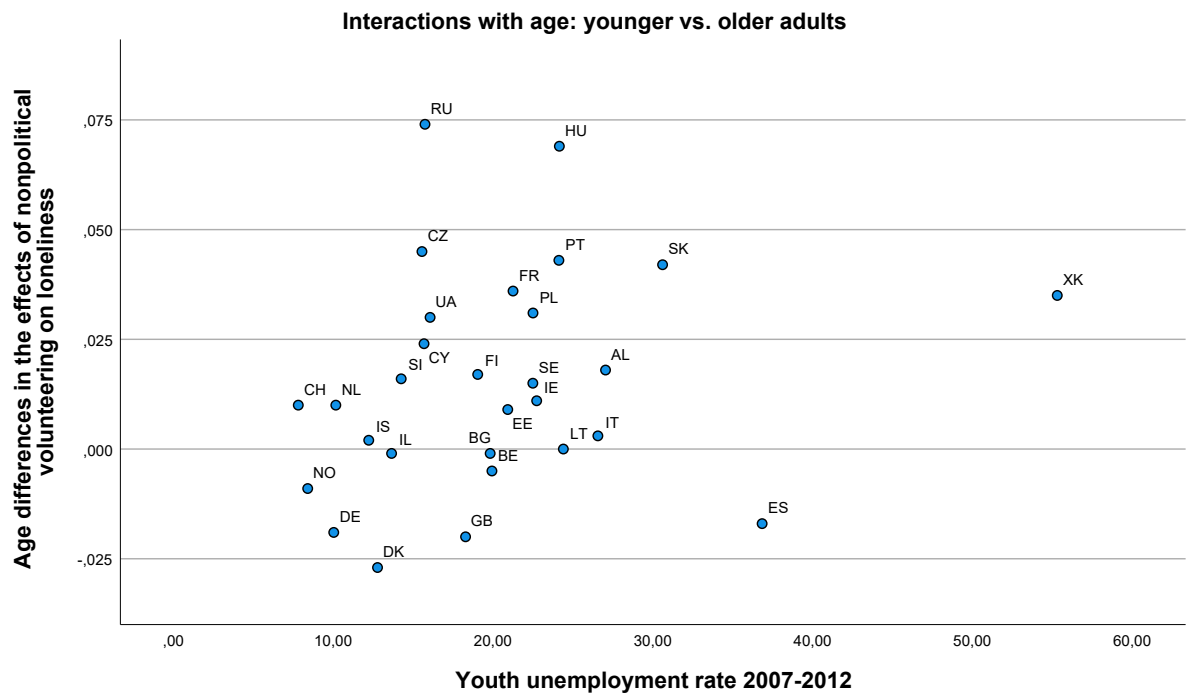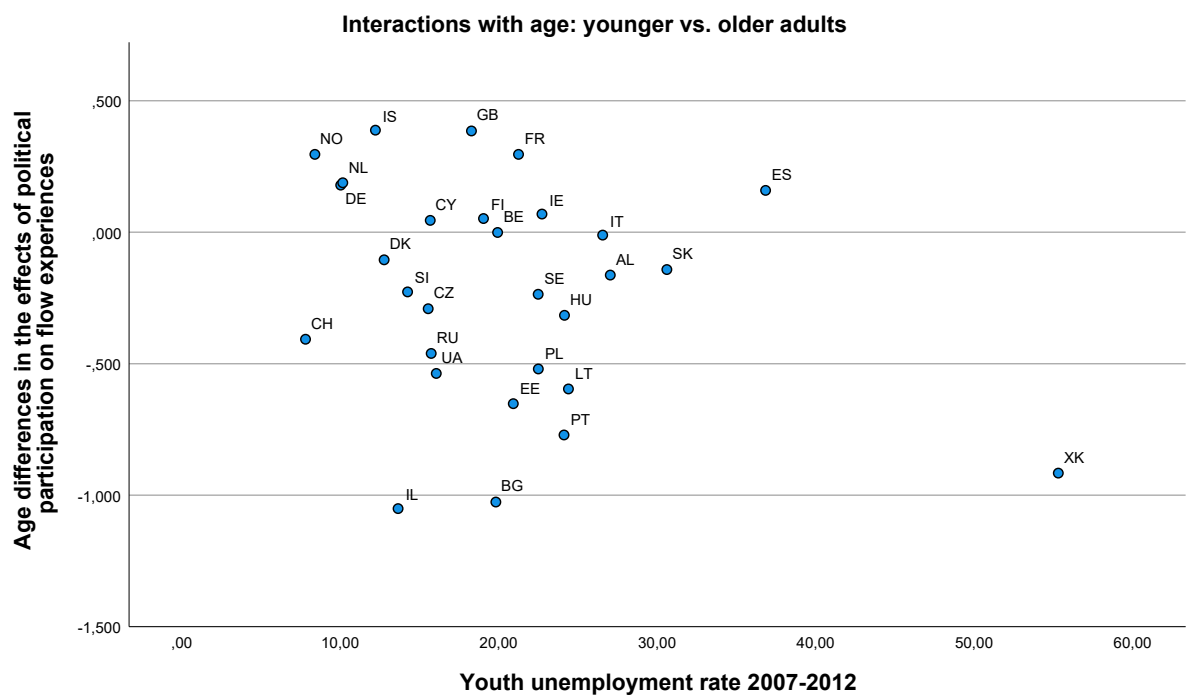

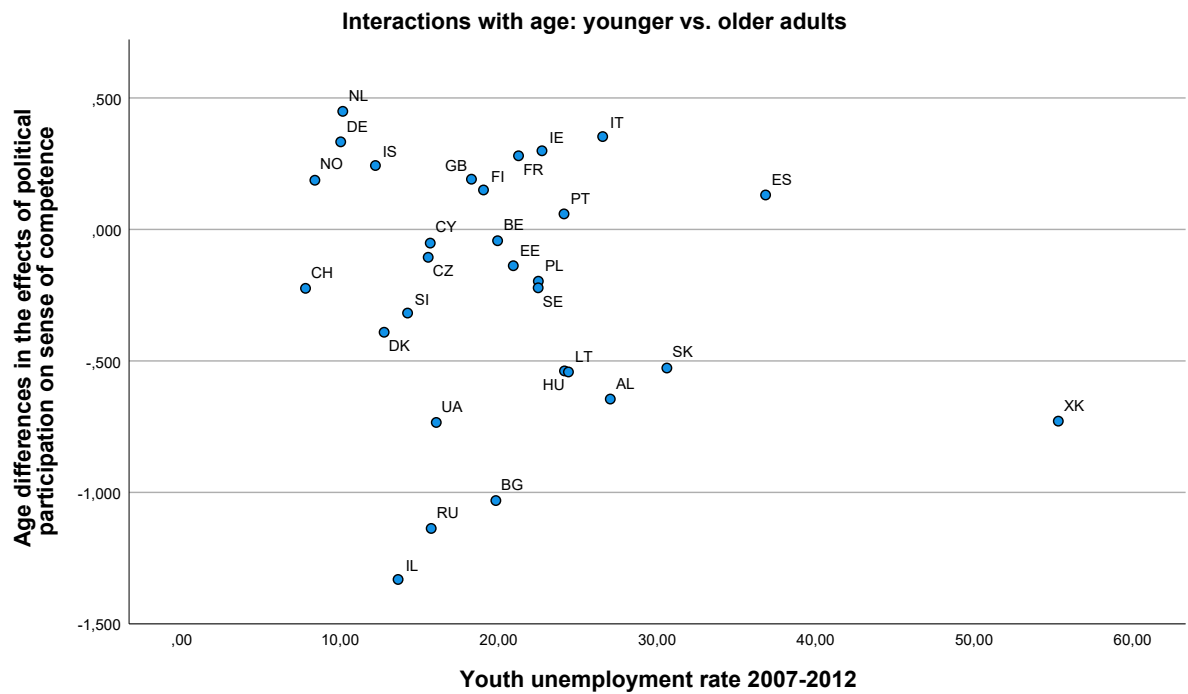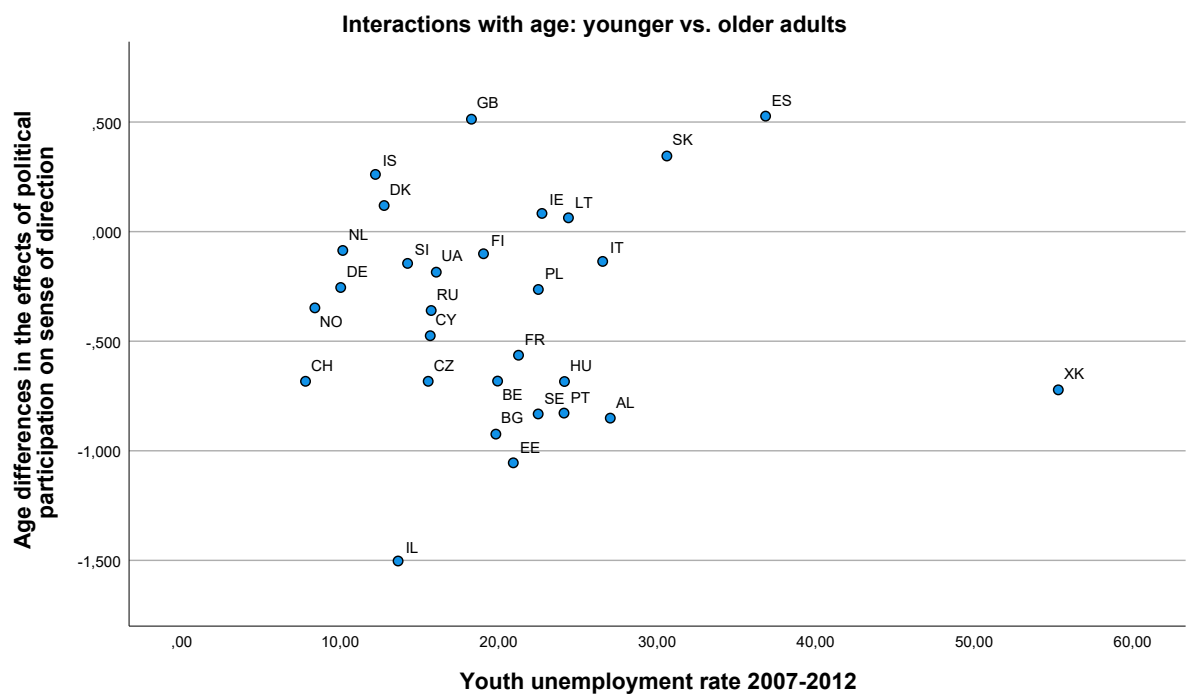

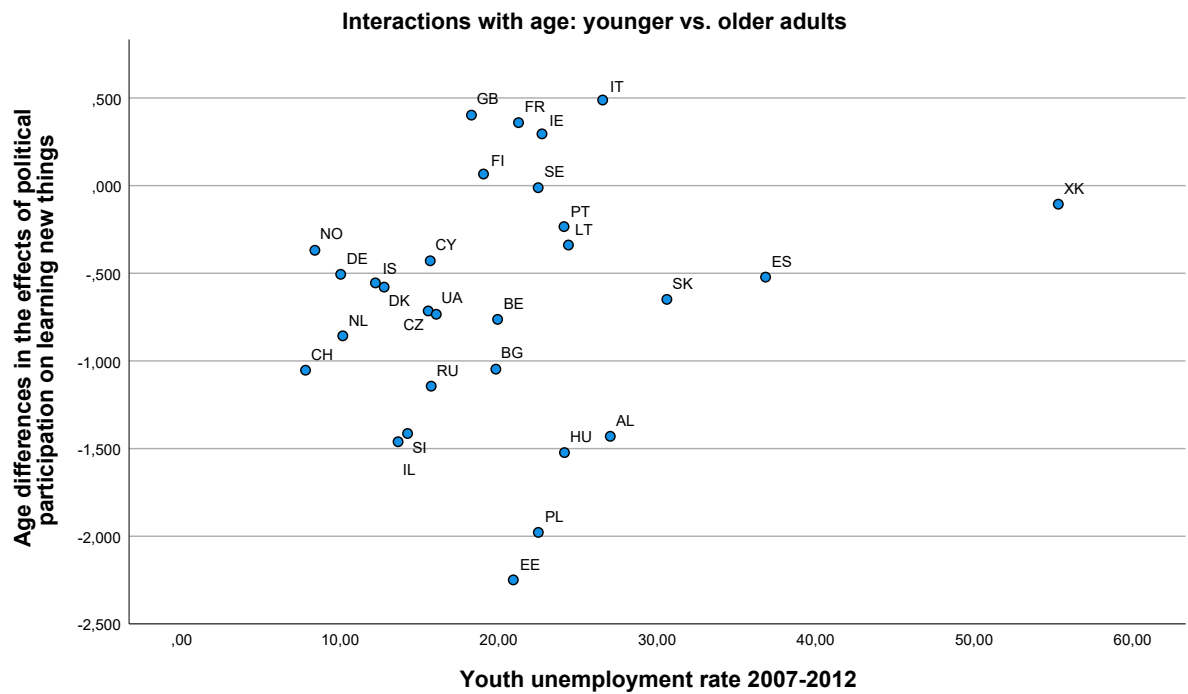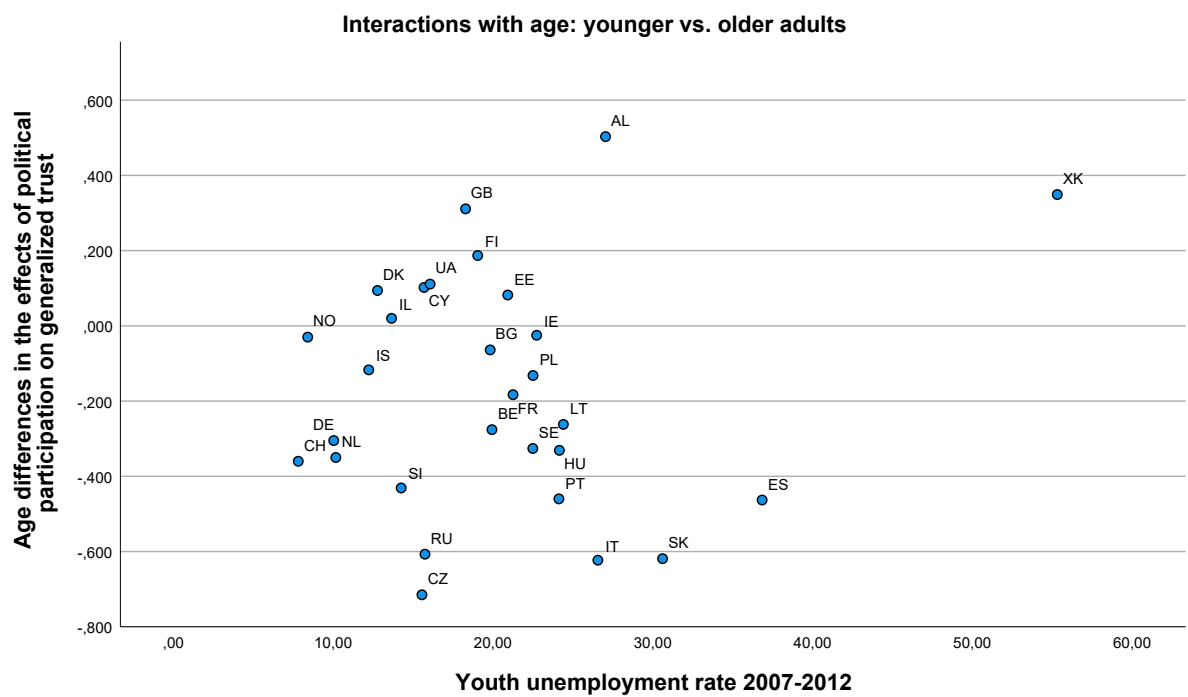

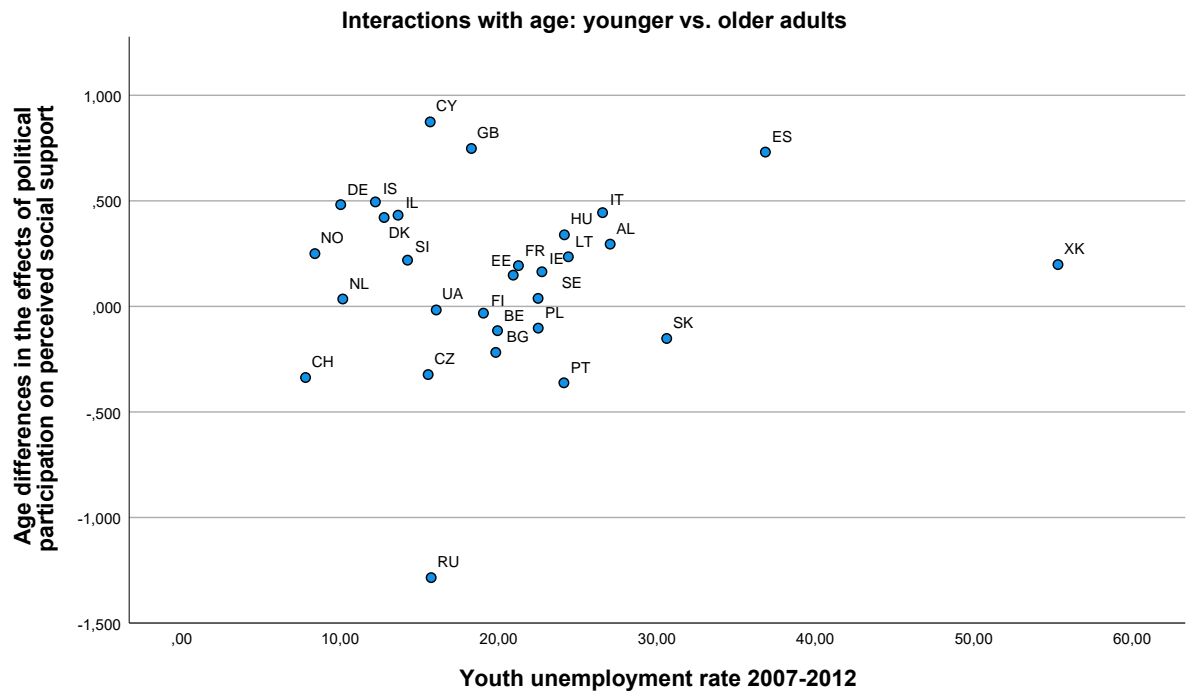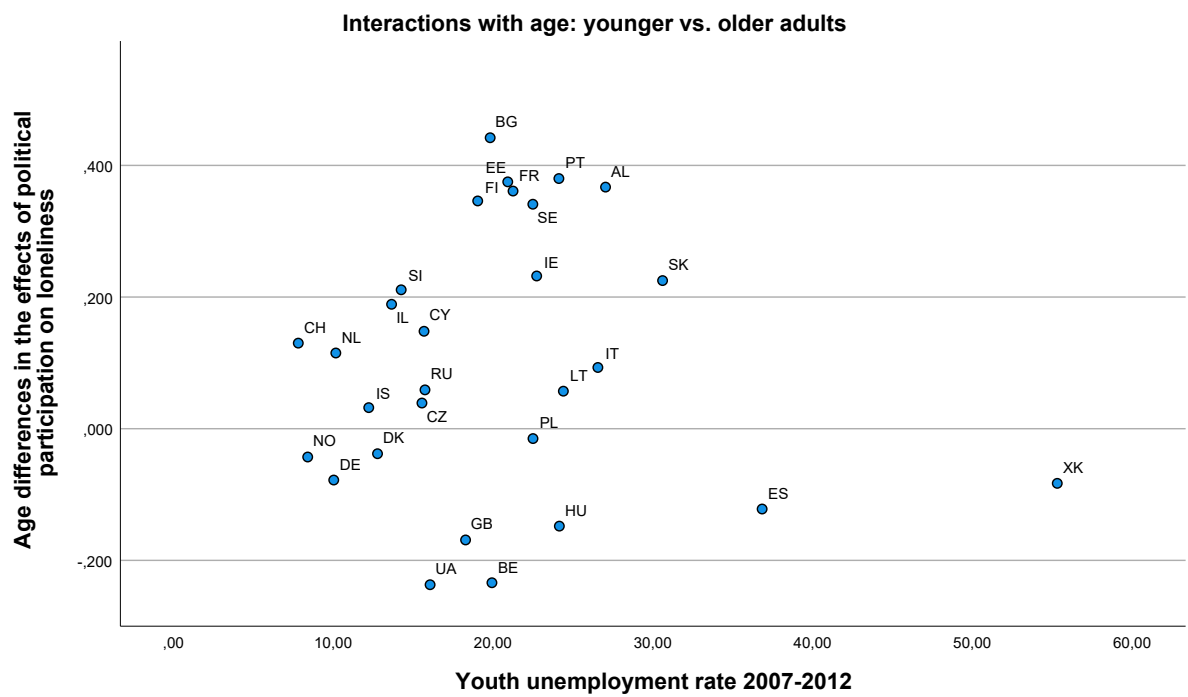

Supplement: S4 Fig — (PDF) [file pone.0281354.s007.pdf]

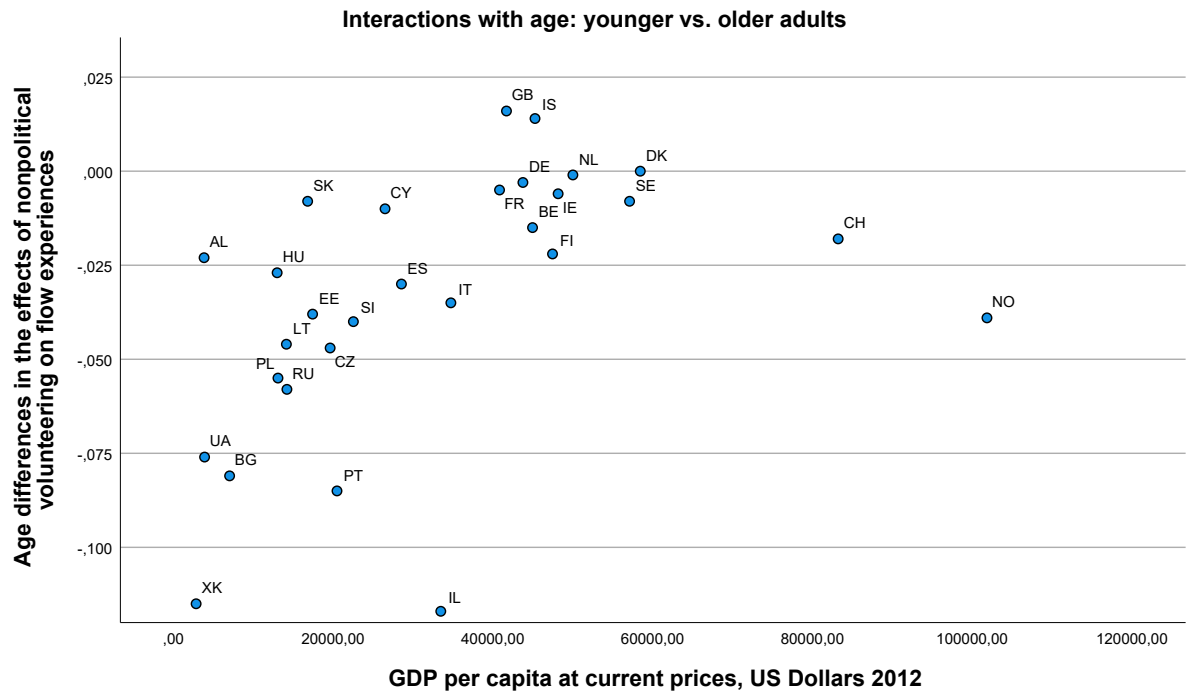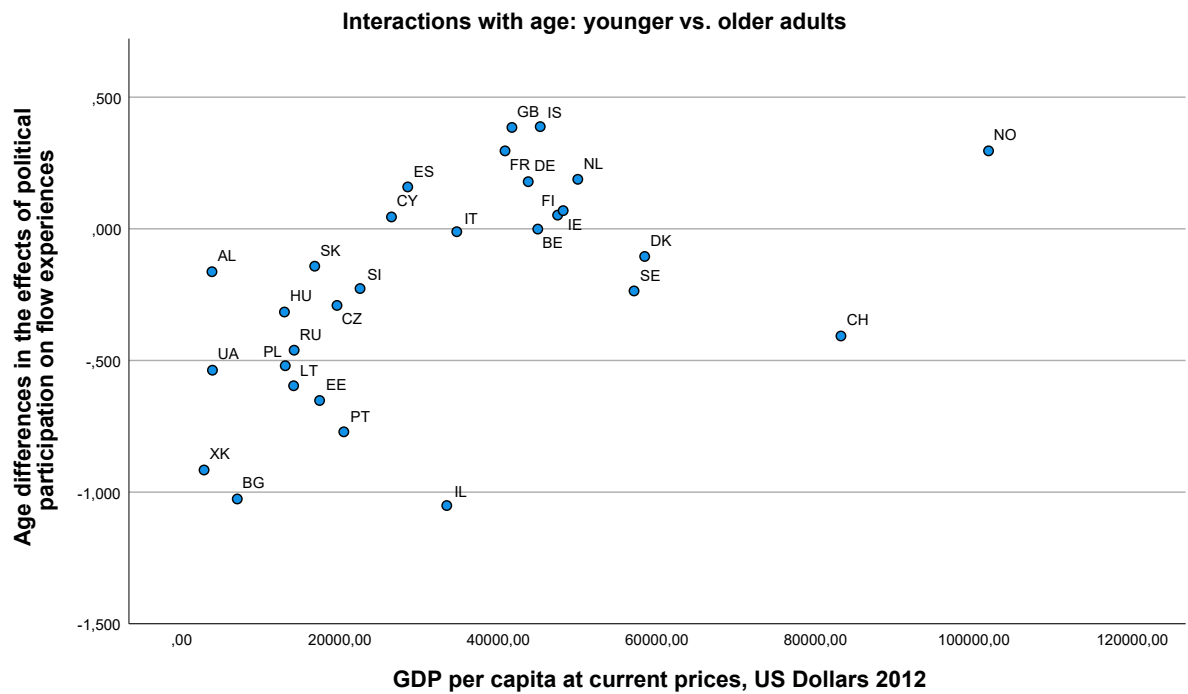

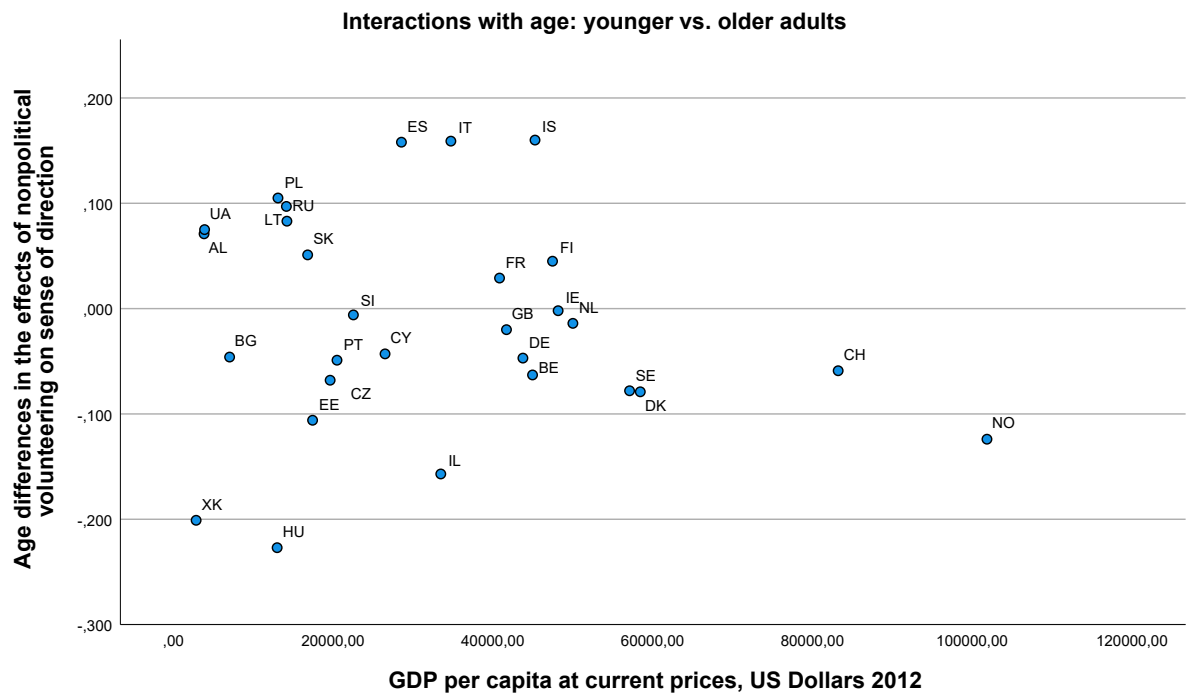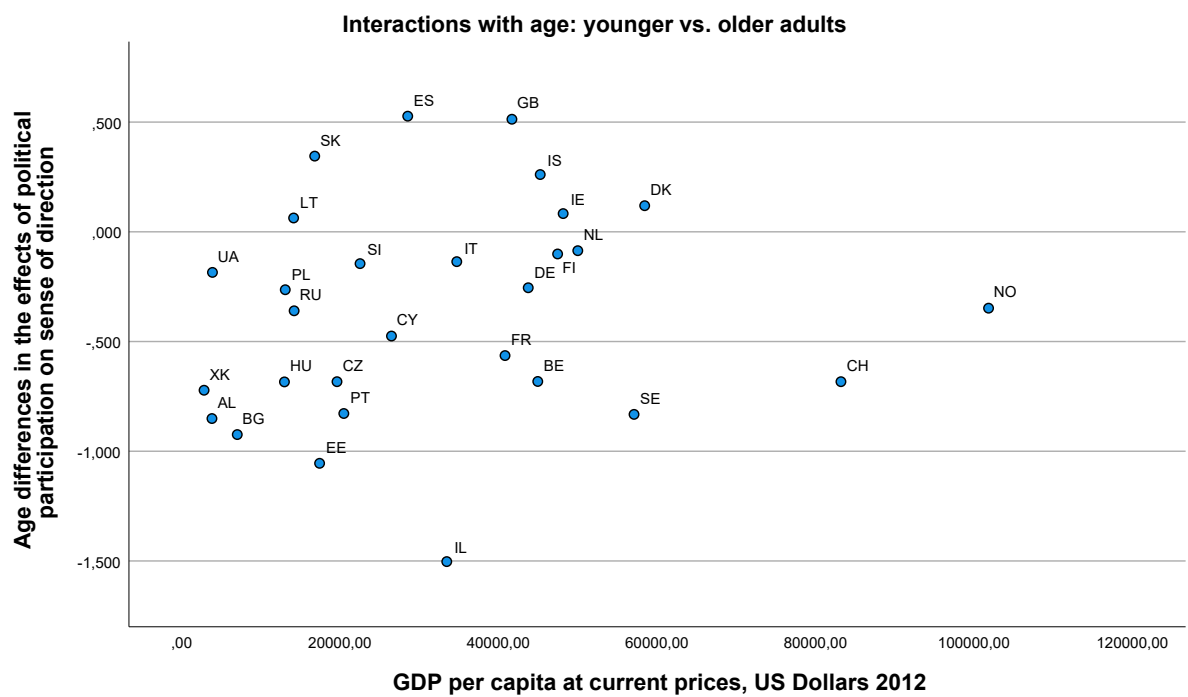

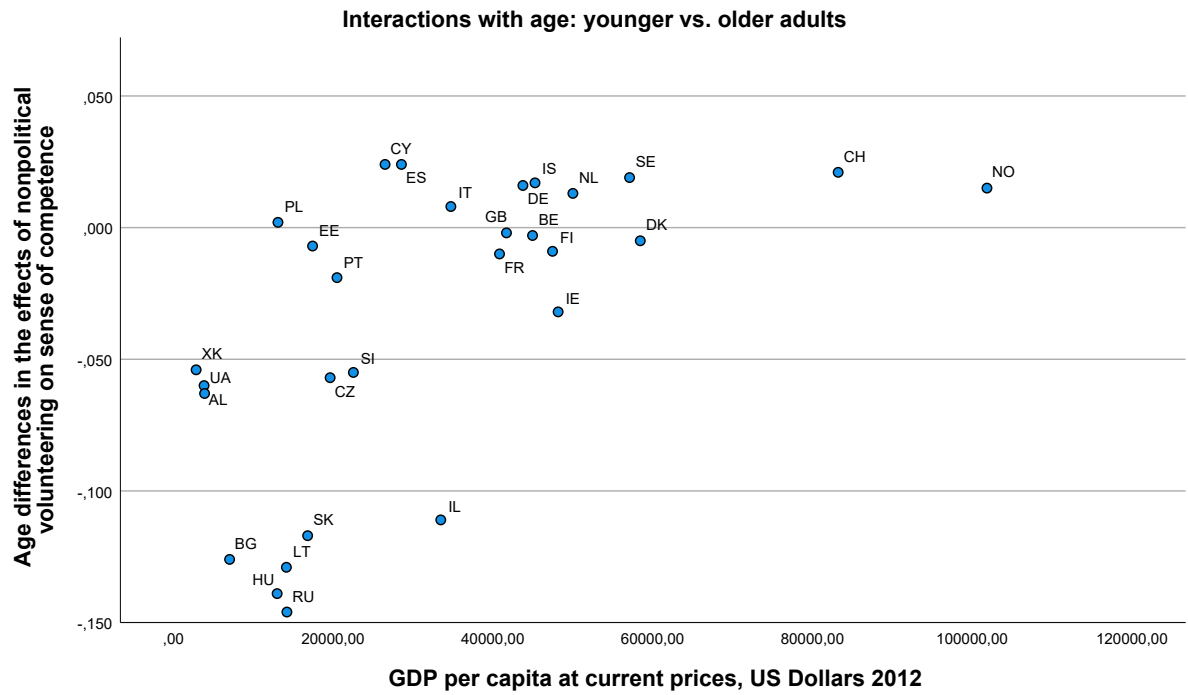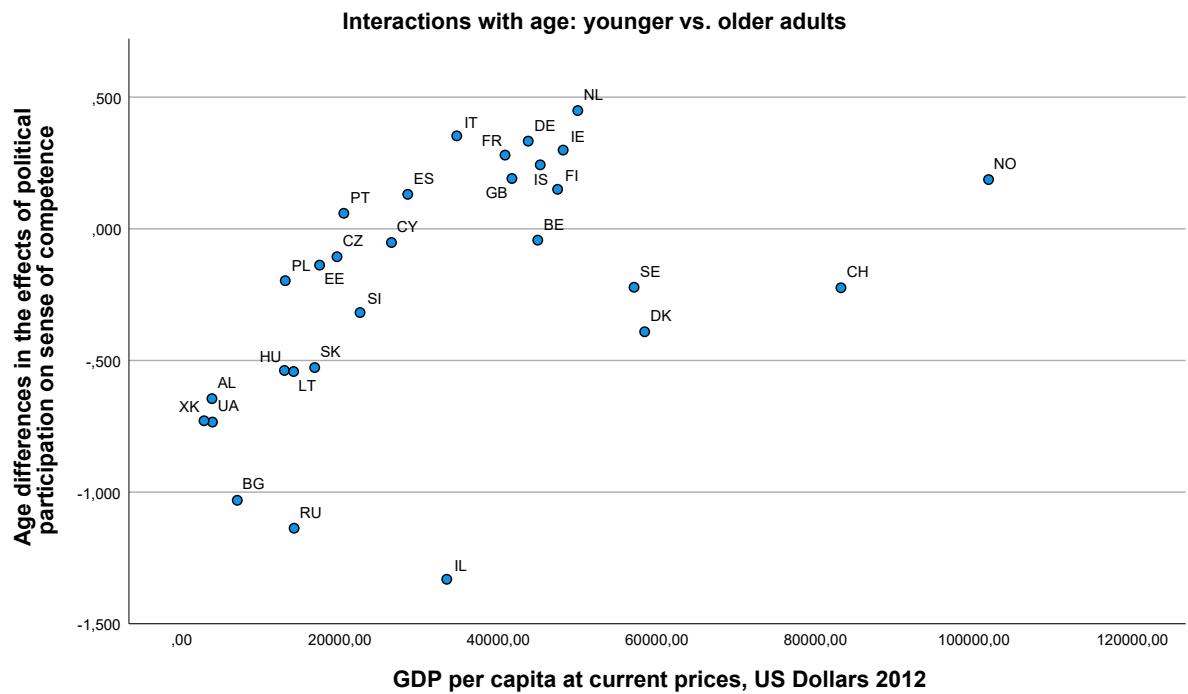

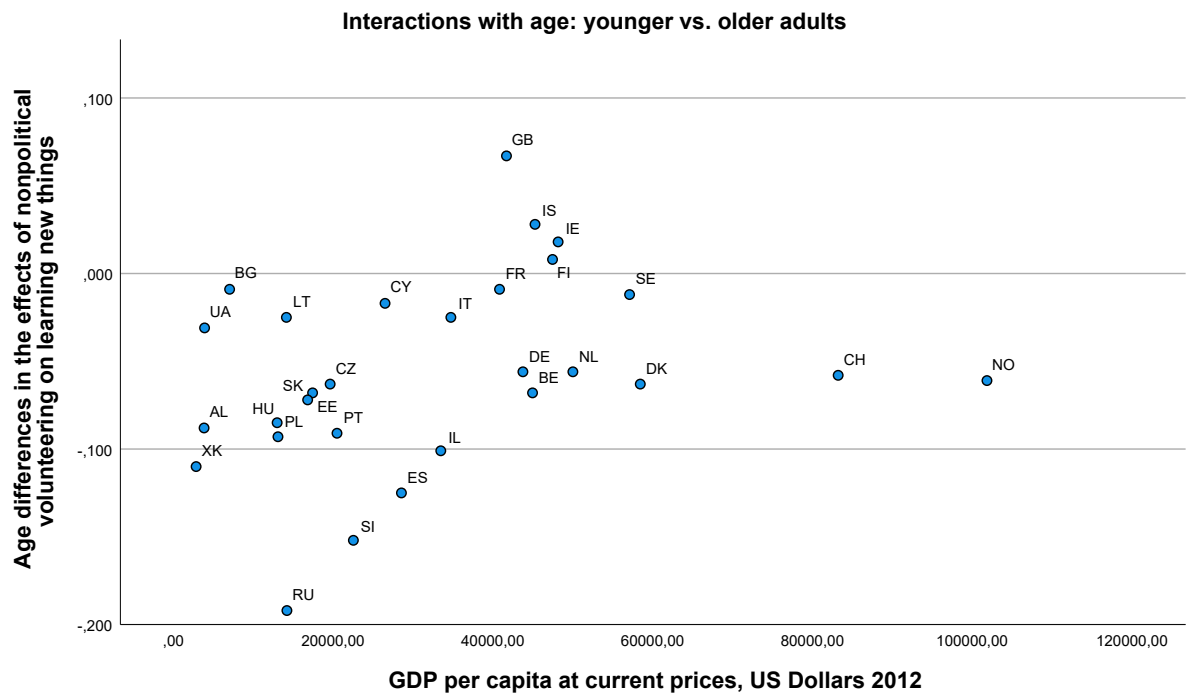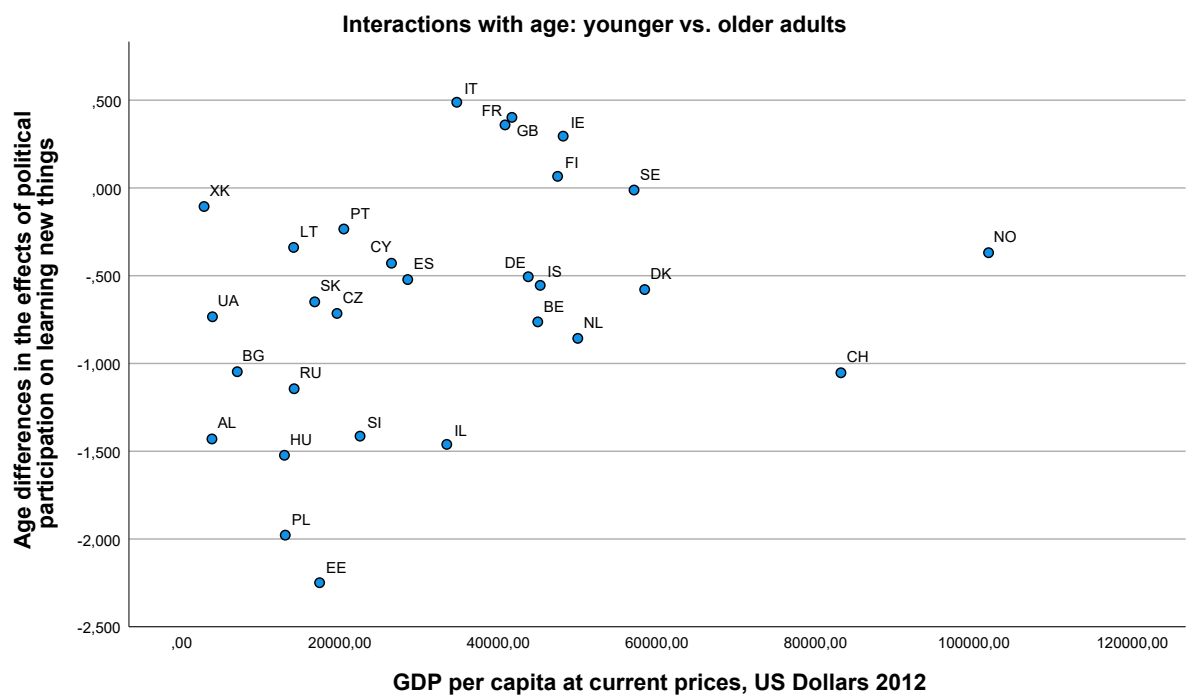

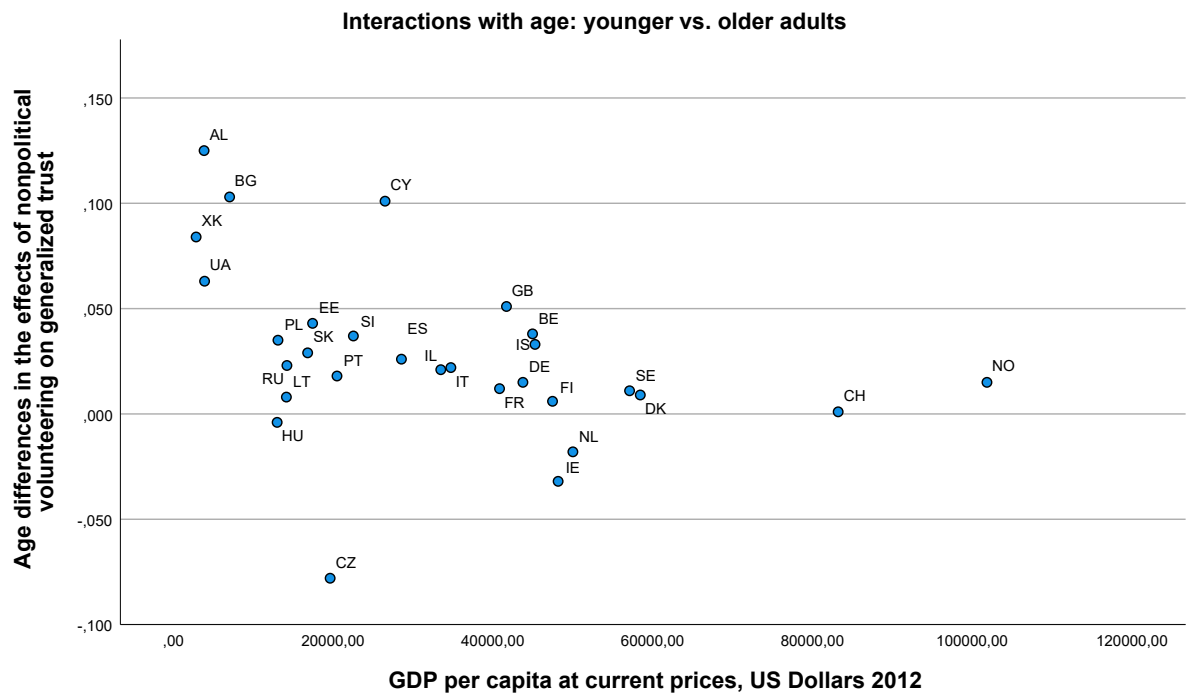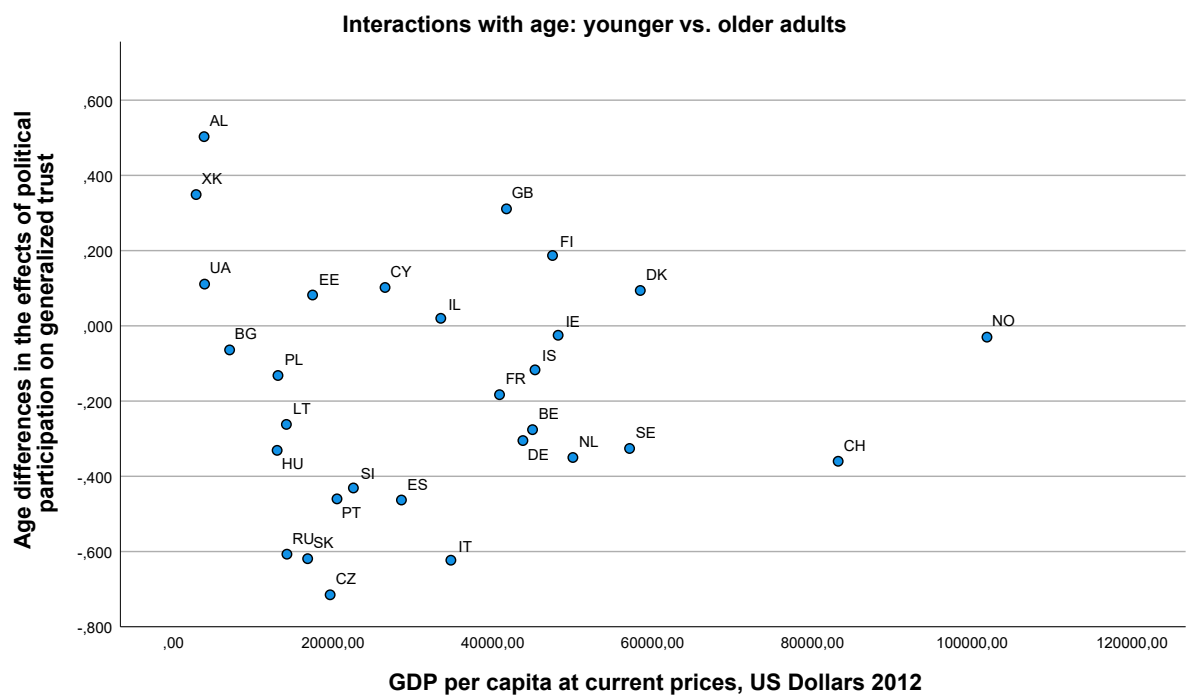

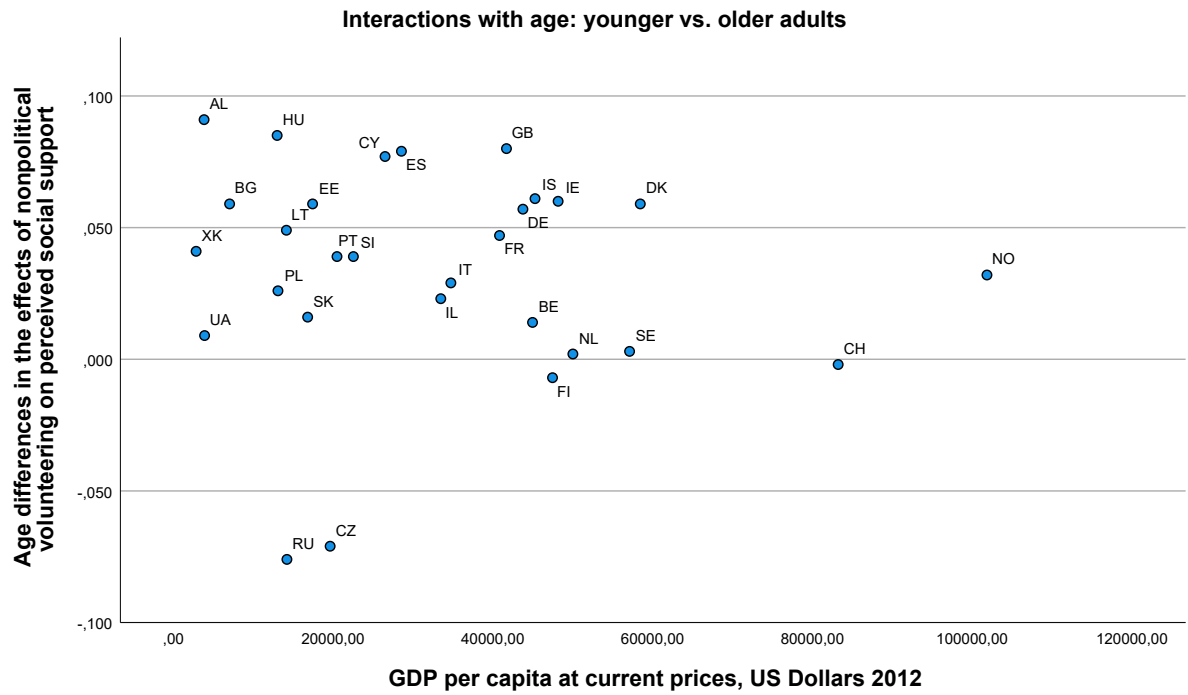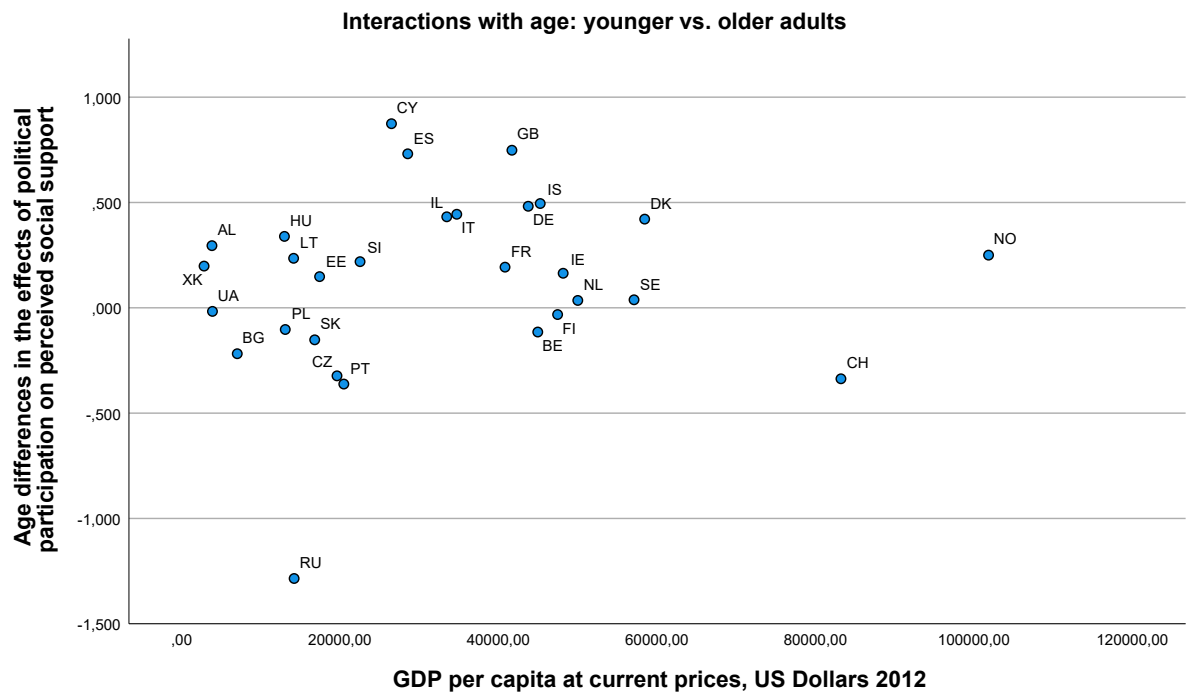

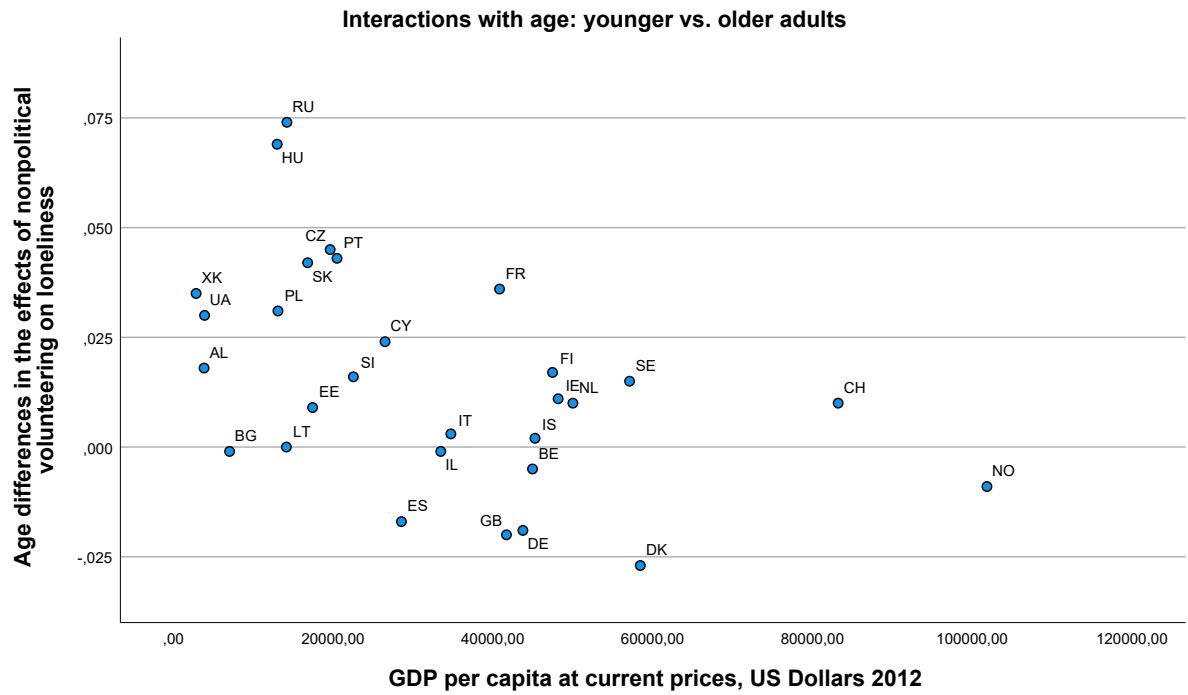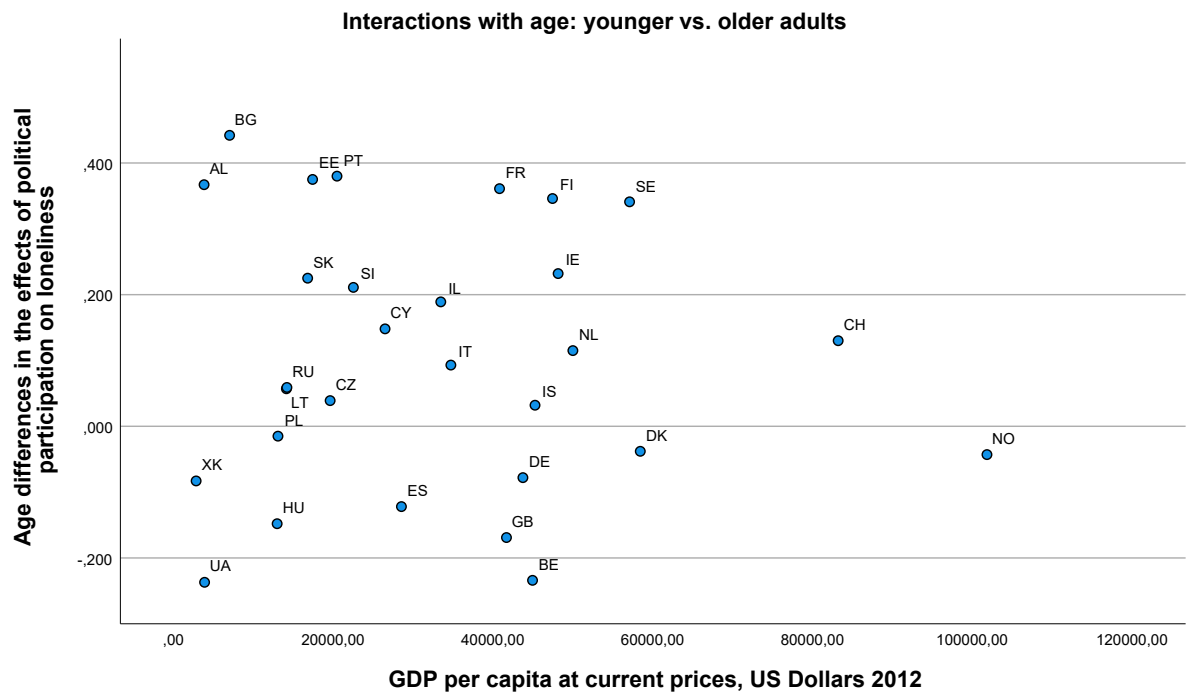

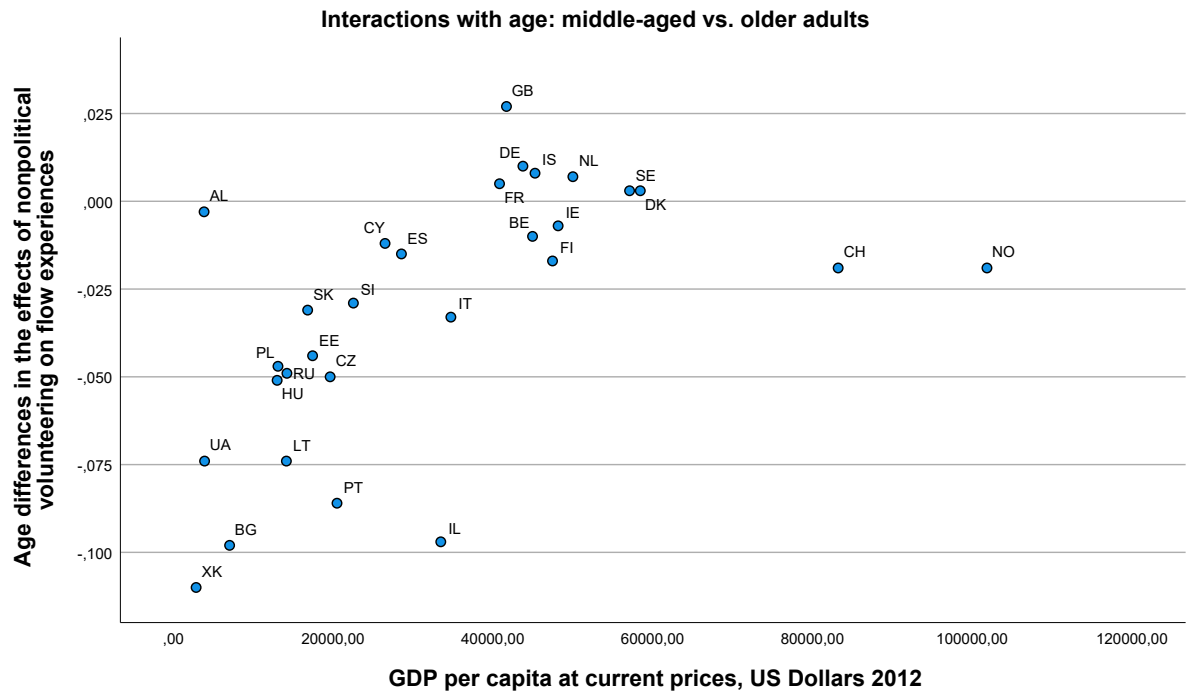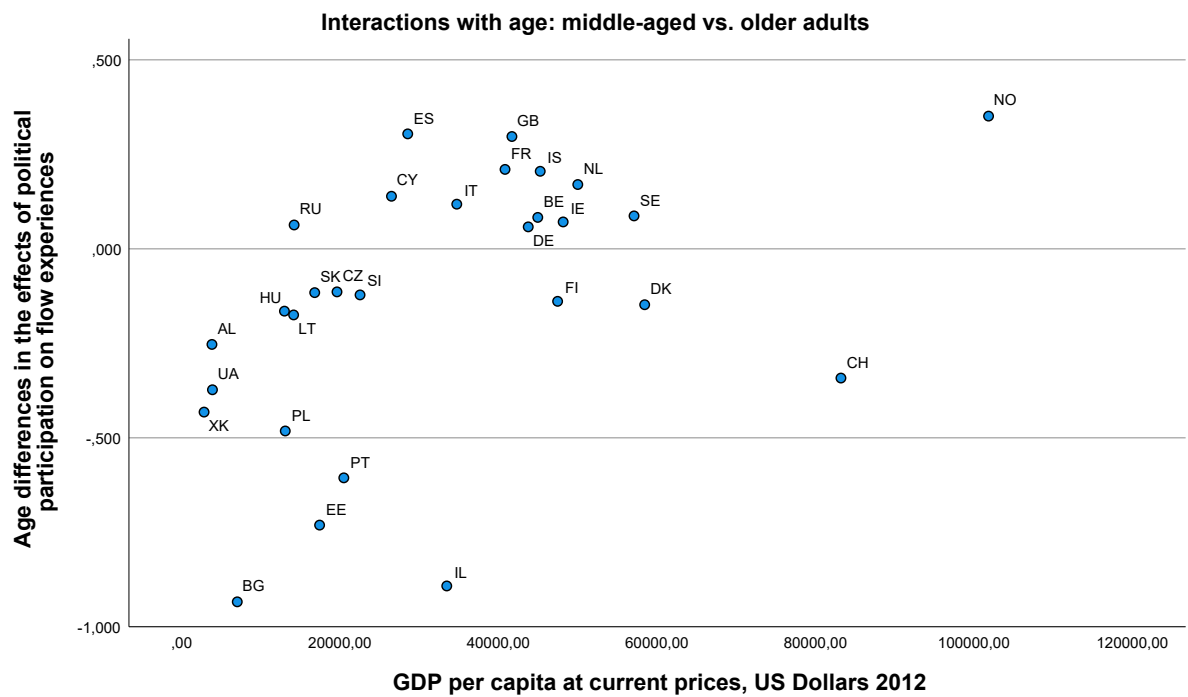

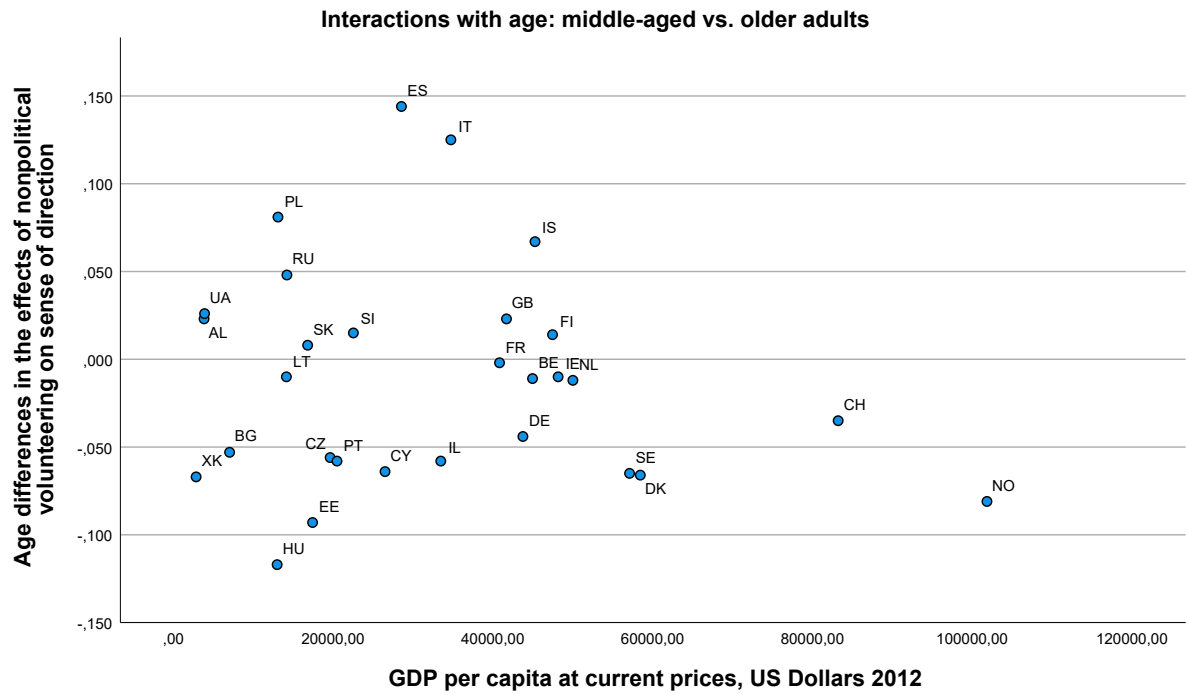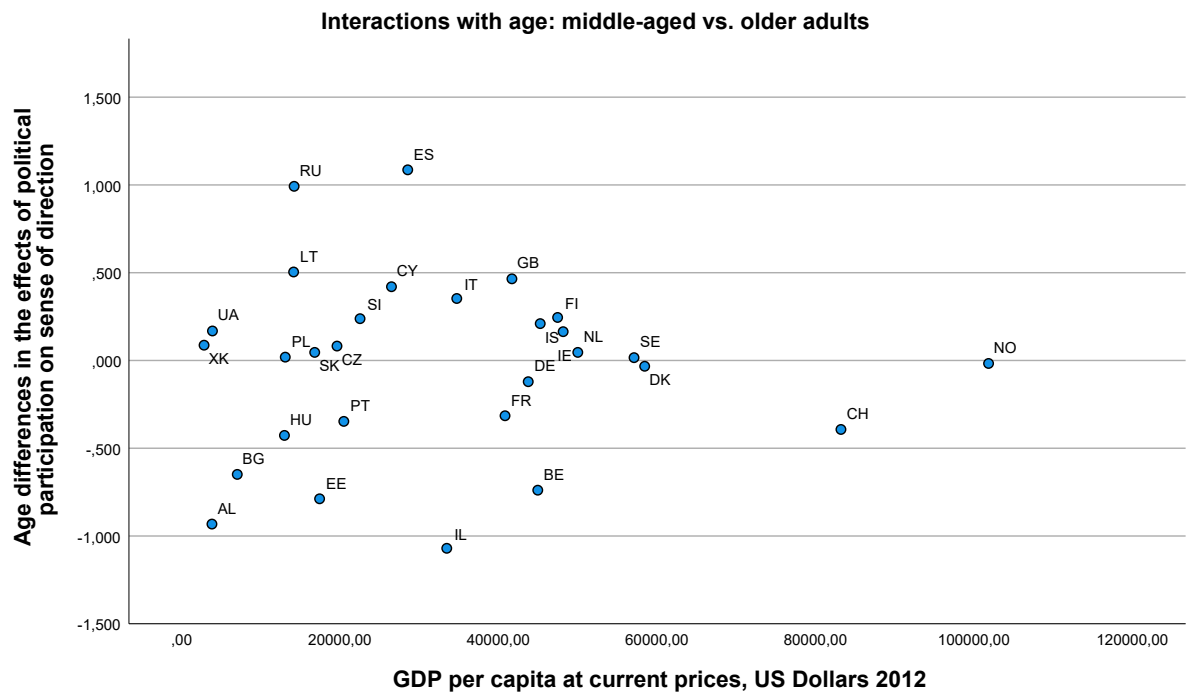

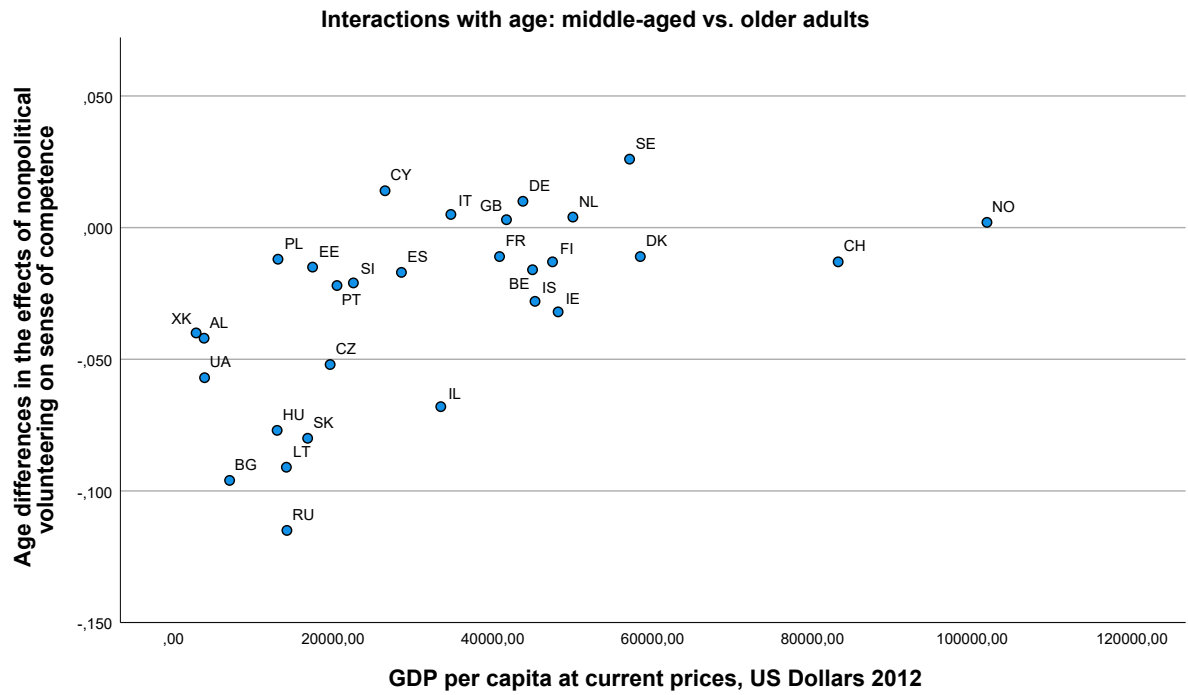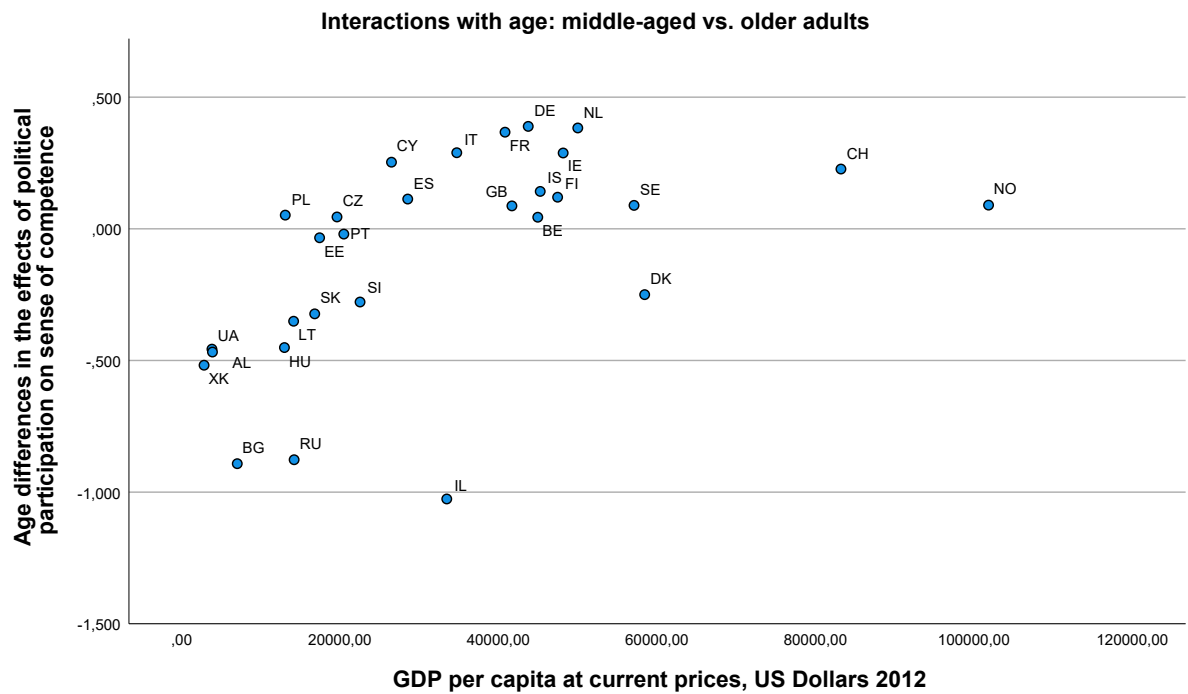

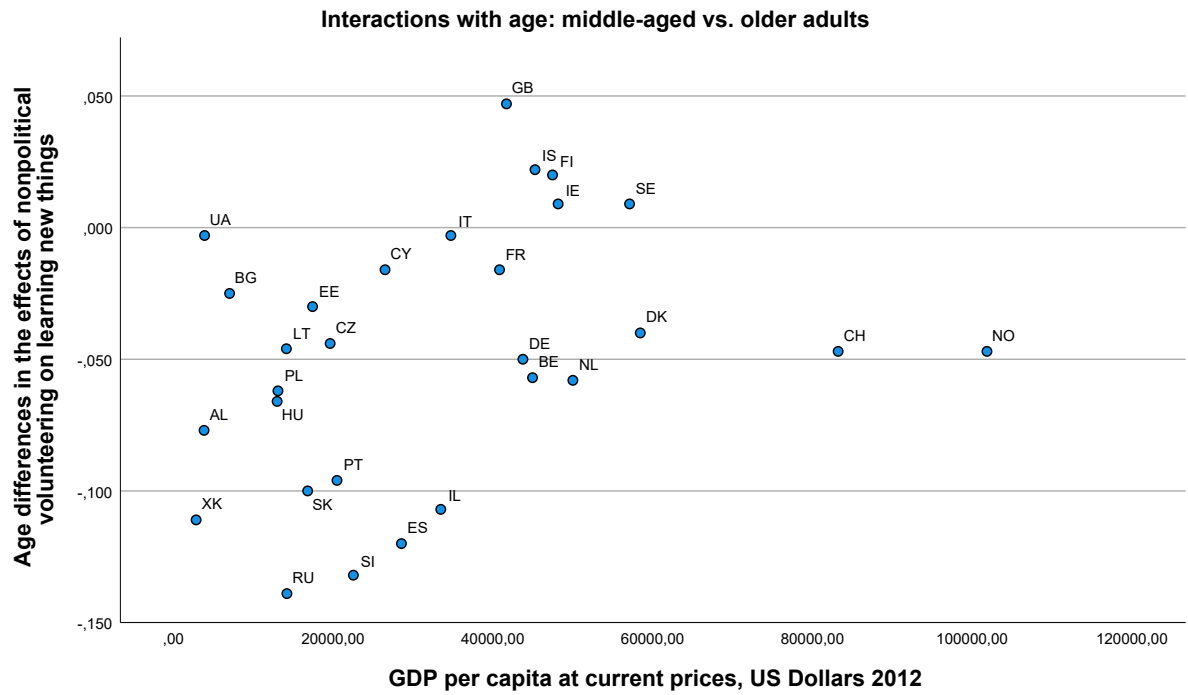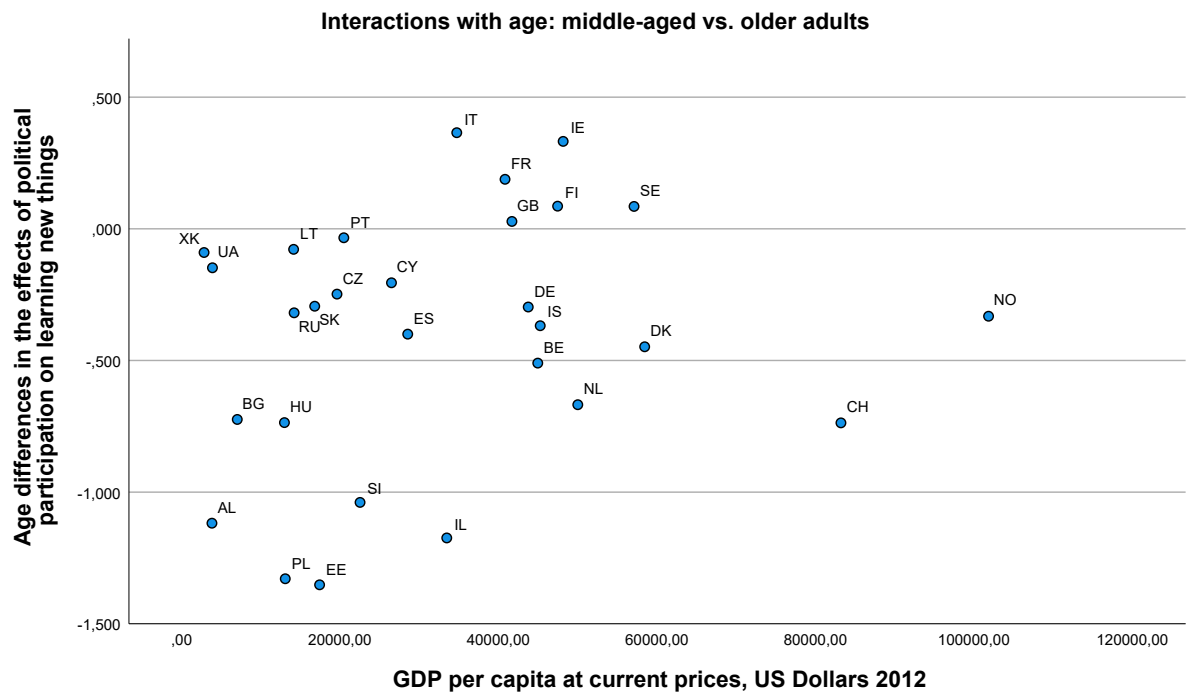

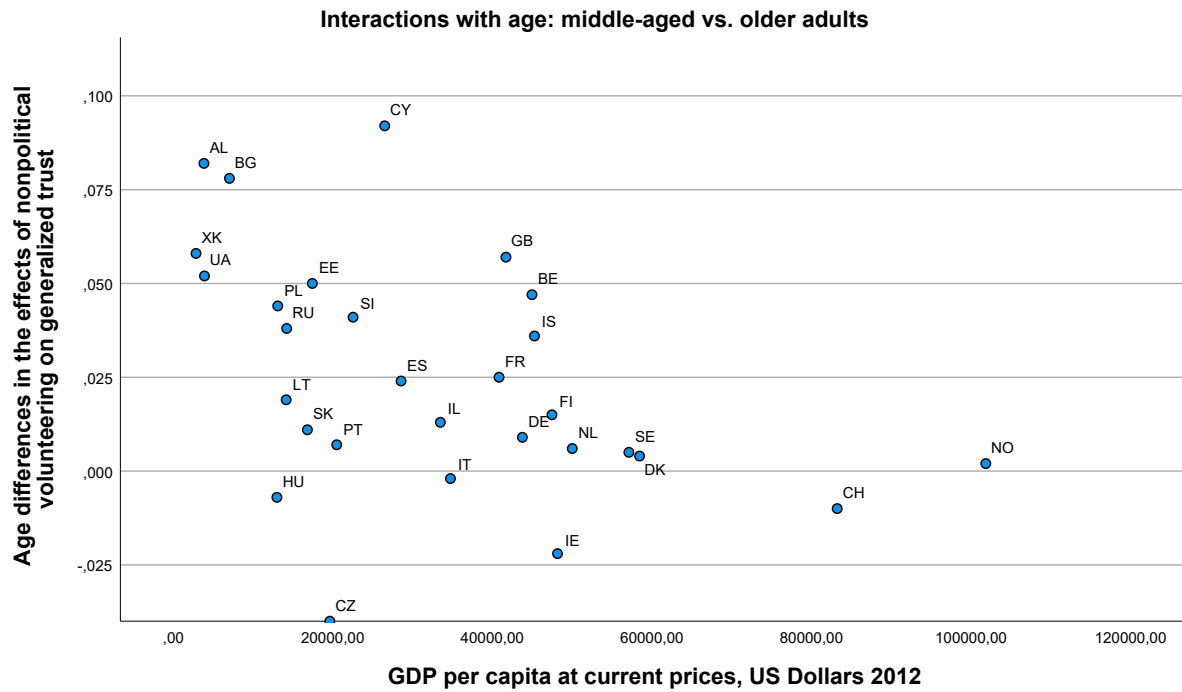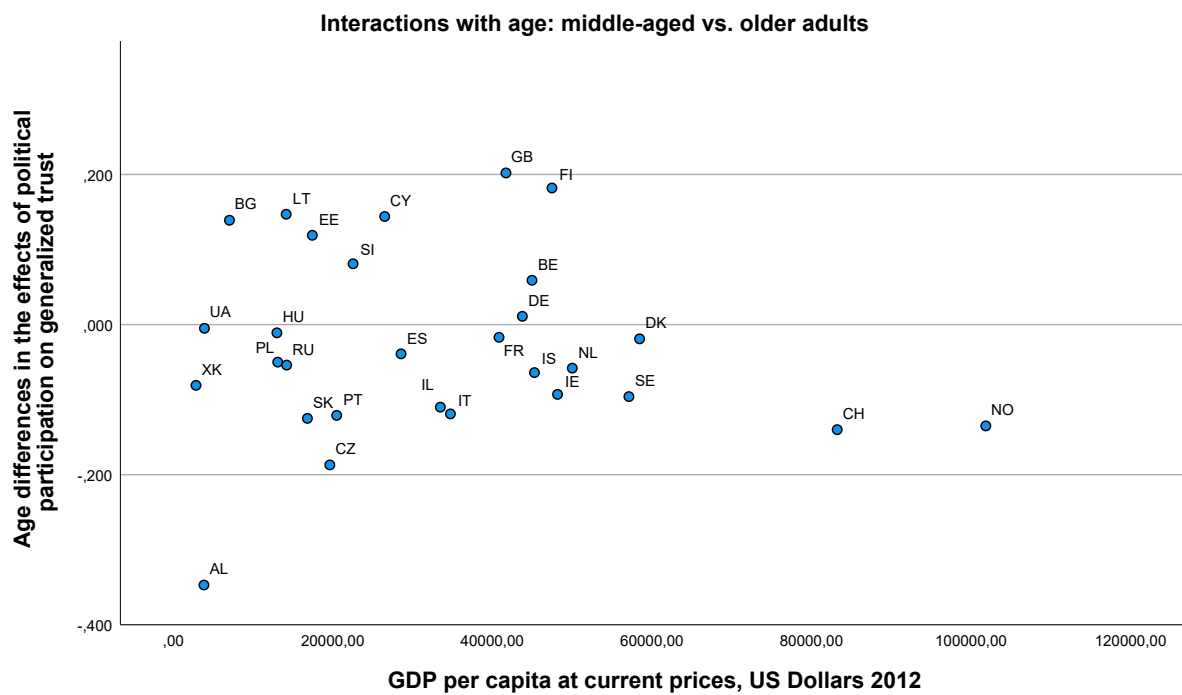

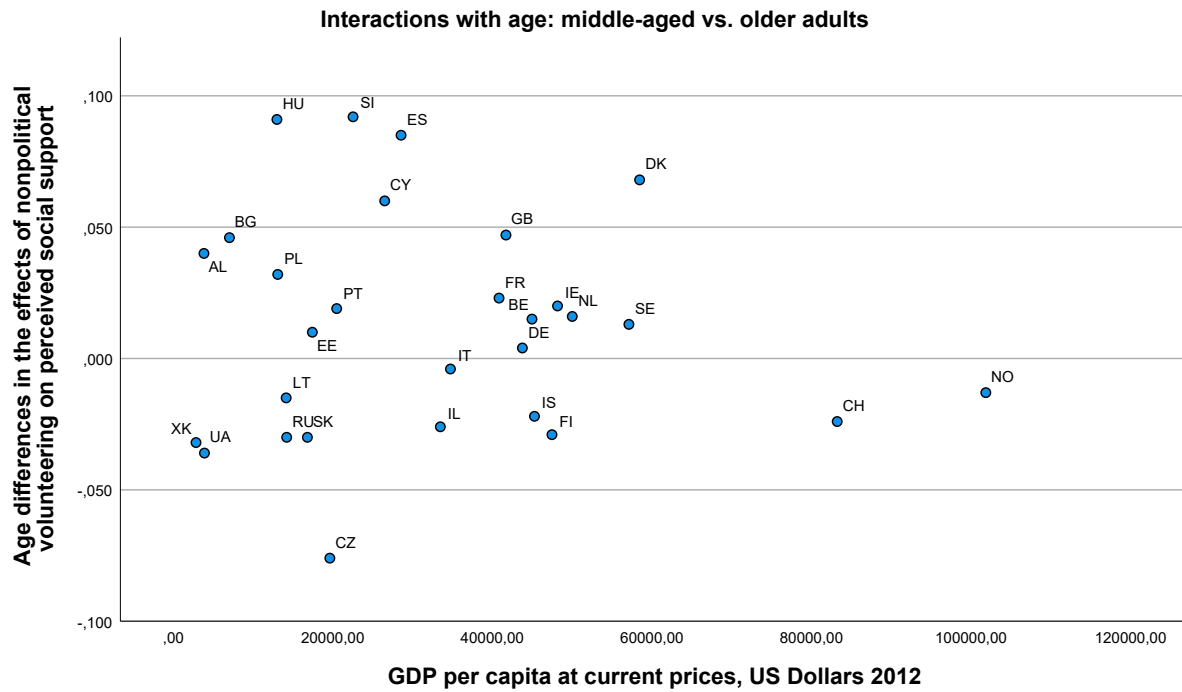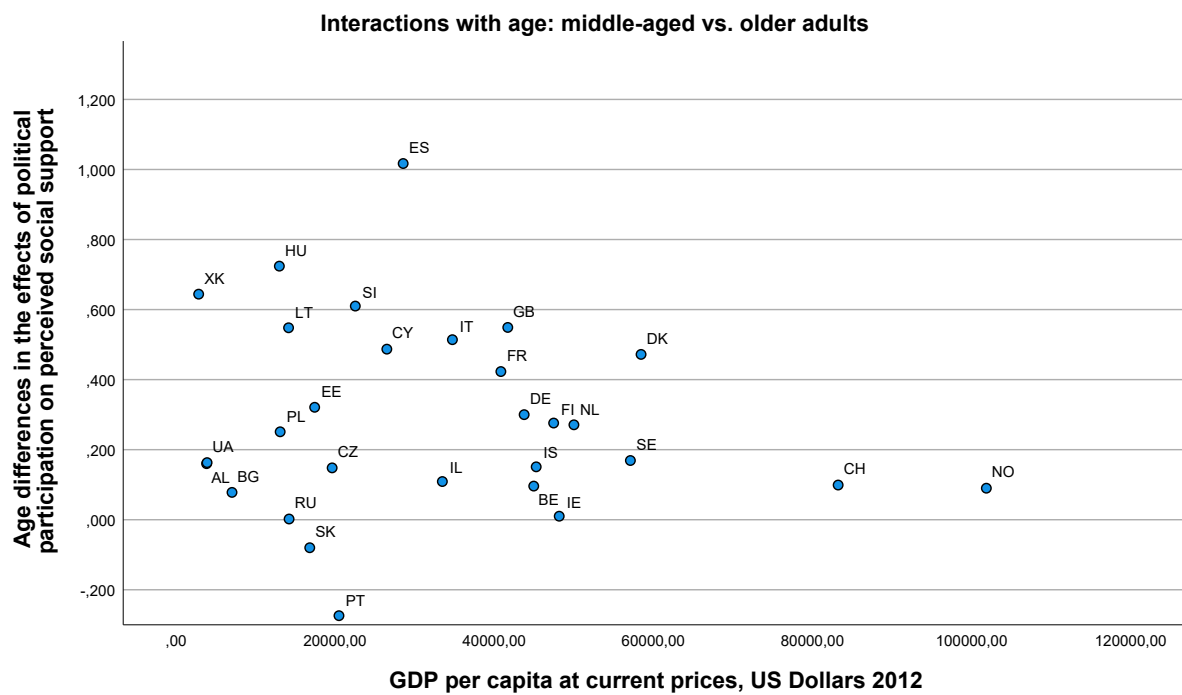

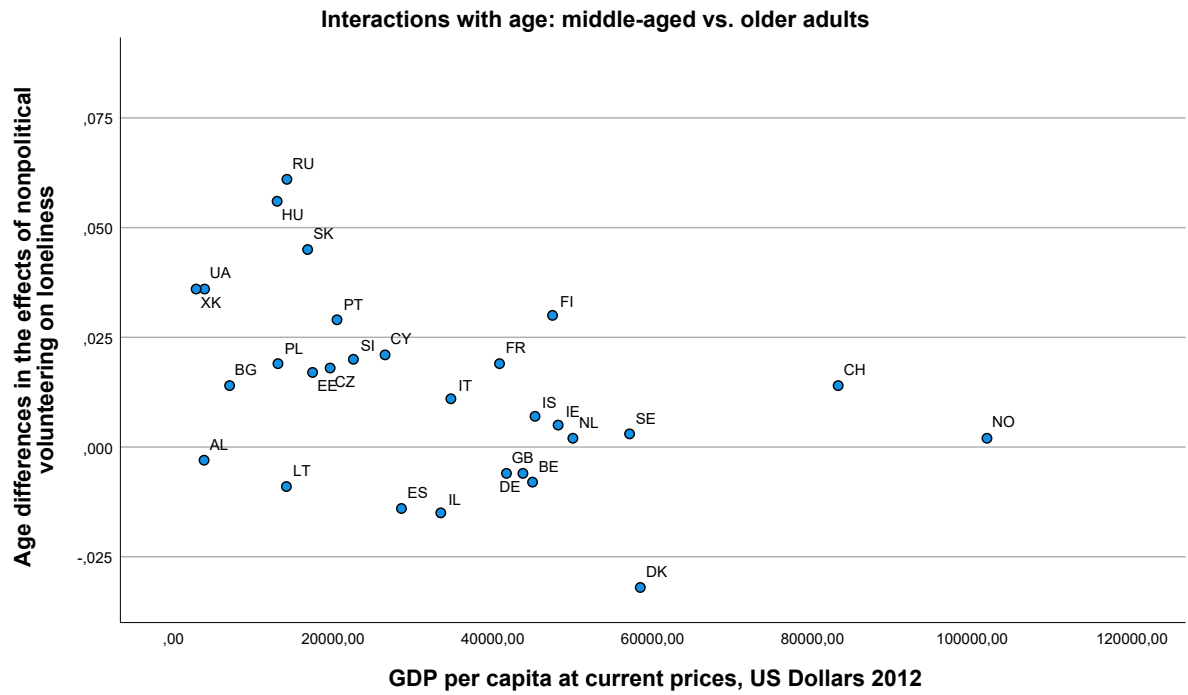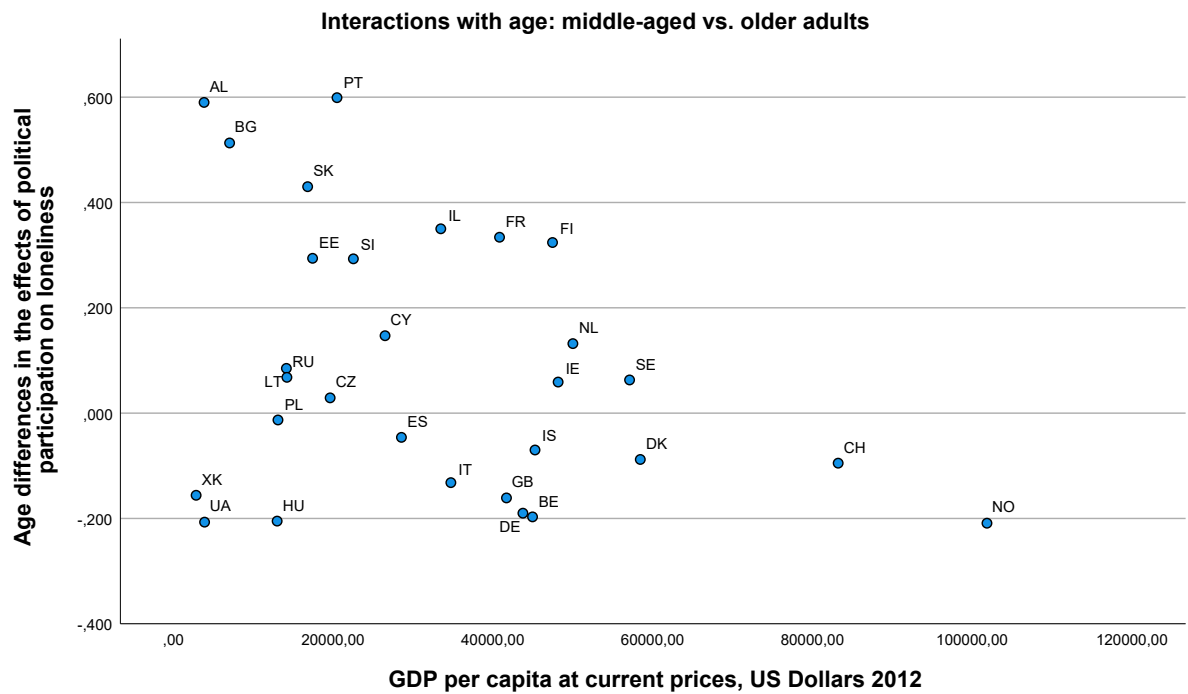

Supplement: S5 Fig — (PDF) [file pone.0281354.s008.pdf]
